# Synthesis and Reactivity of Bis-tris(pyrazolyl)borate Lanthanide/Aluminum Heterobimetallic Trihydride Complexes

**DOI:** 10.1021/acs.inorgchem.4c00824

**Published:** 2024-04-29

**Authors:** Tajrian Chowdhury, Fáinché Murphy, Alan R. Kennedy, Claire Wilson, Joy H. Farnaby, Catherine E. Weetman

**Affiliations:** † Joseph Black Building, School of Chemistry, University of Glasgow, Glasgow G12 8QQ, U.K.; ‡ Department of Pure and Applied Chemistry, University of Strathclyde, 295 Cathedral Street, Glasgow G1 1XL, U.K.

## Abstract

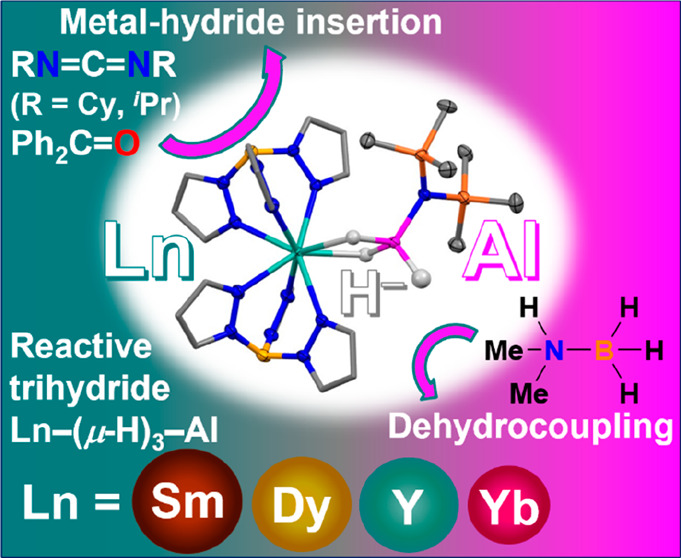

Molecular heterobimetallic hydride complexes of lanthanide (Ln) and main-group (MG) metals exhibit chemical properties unique from their monometallic counterparts and are highly reactive species, making their synthesis and isolation challenging. Herein, molecular Ln/Al heterobimetallic trihydrides [Ln(Tp)_2_(μ-H)_2_Al(H)(N″)] [**2-Ln**; Ln = Y, Sm, Dy, Yb; Tp = hydrotris(1-pyrazolyl)borate; N″ = N(SiMe_3_)_2_] have been synthesized by facile insertion of aminoalane [Me_3_N·AlH_3_] into the Ln–N amide bonds of [Ln(Tp)_2_(N″)] (**1-Ln**). Thus, this is a simple synthetic strategy to access a range of Ln/Al hydrides. Reactivity studies demonstrate that **2-Ln** is a heterobimetallic hydride, with evidence for the cooperative nature of **2-Ln** shown by the catalytic amine–borane dehydrocoupling under ambient conditions in contrast to its monomeric counterparts.

## Abstract 2

[Ln(Tp)_2_(μ-H)_2_Al(H)(N″)] [**2-Ln**; Ln = Y, Sm, Dy, Yb; Tp = hydrotris(1-pyrazolyl)borate; N″ = N(SiMe_3_)_2_] can be readily accessed via insertion chemistry. Reactivity studies show that **2-Ln** is a bimetallic hydride and can affect the catalytic dehydrocoupling of amine−boranes in line with Ln Lewis acidity.

Heterobimetallic hydride complexes are desirable synthetic targets because they are highly reactive species and exhibit unique chemical reactivity properties distinct from their monometallic counterparts.^1^ However, their high reactivity makes the synthesis and isolation of heterobimetallic hydrides challenging. This is particularly true in the case of lanthanide (Ln)/aluminum (Al) hydrides, and as such careful ligand design is required to stabilize the highly electrostatic Ln and Al cations, which are bridged by small hydrides. Bis-substituted cyclopentadienyl (Cp^R^) ligands have enabled the synthesis of Ln/Al heterobimetallic hydrides predominantly by salt metathesis routes, e.g., [{Ln(Cp^R^)_2_(X)}_*n*_] (X = halide) with LiAlH_4_.^1a^ However, the hydride and Ln/Al heterobimetallic hydride chemistry in Trofimenko’s isolable scorpionate nitrogen-donor tris(pyrazolyl)borate (Tp^R^) ligand environment is much less well established.^2^

Ln homometallic hydrides [{Ln(Tp^R^)(H)_2_}_*n*_] supported by a single (Tp^R^) ligand have been isolated, in which the size of the substituents in the 3 and 5 positions of the Tp ligand dictates the nuclearities of the complex (R = ^*i*^Pr, *n* = 3; R = Me, *n* = 4; R = H, *n* = 6).^2c^ Notably only for [{Ln(Tp^Me2^)(H)_2_}_4_] was Ln larger than Y, and for Tp^R^ = Tp^3-*t*Bu,5-Me^, no Ln(III) complexes were isolable. More importantly, Tp^3-*t*Bu,5-Me^ enabled the isolation of the first molecular ytterbium(II) hydride ([**I**], Figure 1). These Tp^R^-supported Ln hydrides are highly reactive; complex [**I**] undergoes facile activation chemistry at room temperature,^2a^ while [**II-Y**] mediated the hydrogenation and coupling of carbon monoxide (Figure 1).^2b^ The Tp^3-*t*Bu,5-Me^ ligand environment can also support monomeric Ln/Al heterobimetallic dihydrides [Ln(Tp^3-*t*Bu,5-Me^)(μ-HAlMe_3_)_2_], where Ln = Y and Lu ([**III**], Figure 1).^2i^ Complex [**III**] was synthesized by the reaction of [Ln(Tp^3-*t*Bu,5-Me^)(Me)_2_] with 2 equiv of HAlMe_2_ and shown to deprotonate amines to access a rare imide-bridged Ln–Al hydride ([**IV**], Figure 1).^2i,3^ The insertion of substituted alanes into Ln–Me σ-bonds has been shown to be a viable route to Ln/Al heterobimetallic dihydrides in the bis-Cp* (Cp* = C_5_Me_5_) ligand environment. The reaction of HAl(N″)_2_ (N″ = N(SiMe_3_)_2_) with [(Cp*)_2_Y(Me)(THF)] yielded [(Cp*)_2_Y(μ-H)_2_Al(N″)(Me)] ([**V**]), which was the first example of a Ln catalyst in the hydroalumination of 1-octene.^4^

**Figure 1:**
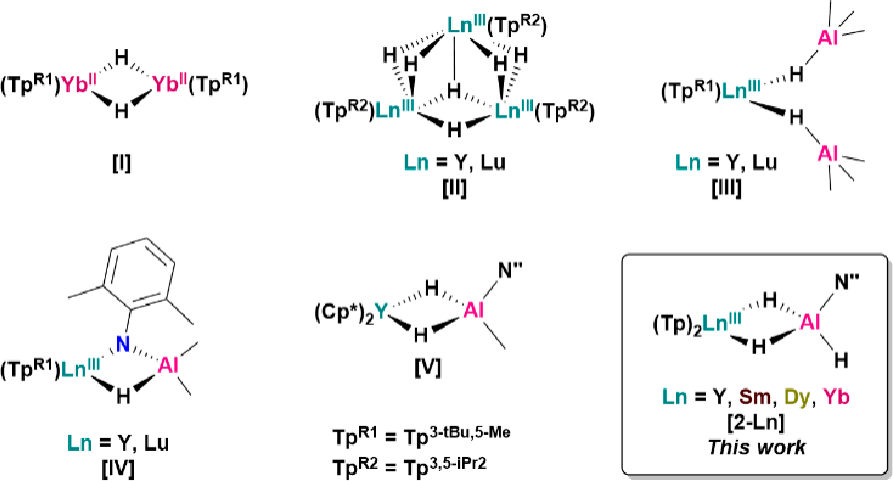
Reactive Ln hydride and Ln/Al heterobimetallic hydride complexes supported by Tp^R^ and Cp^R^ ligands.

Using Tp^R^, the Ln scope is limited to Y and Lu, and there are no bis-Tp-stabilized heterobimetallic Ln/Al complexes directly analogous to the isolable bis-Cp^R^ complexes. Recently, monomeric bis-Tp complexes [Ln(Tp)_2_(X)] (Ln = Y, Dy, Yb; X = triflate)^5^ have shown themselves to be a versatile tool for accessing new Ln-element bonds (E = O,^5a^ N^5b^) because the anion can be readily exchanged via metathesis or protonolysis. Herein, we report the facile synthesis of molecular Ln/Al heterobimetallic trihydrides in the bis-Tp ligand environment, which is applicable to a wide range of Ln.

We began by extending the scope of the [Ln(Tp)_2_(X)] complexes to include Sm(III), allowing for a representative range of Ln(III) ions to be studied. Following the same method as previously reported,^5^ [Sm(Tp)_2_(N″)] (**1-Sm**) was obtained by metathesis between the heteroleptic samarium (III) triflate [Sm(Tp)_2_(OTf)] (OTf = CF_3_SO_3_) with K(N″) in toluene. To target Ln/Al hydride species, we opted to use Lewis base-stabilized alanes, [L·AlH_3_] (L = NMe_3_, N-heterocyclic carbene) because these should undergo insertion chemistry into the Ln–N″ bond akin to the formation of [**III**] and [**V**].^2i,4^ The reactions of IDippAlH_3_ [IDipp = 1,3-bis(2,6-diisopropylphenyl)imidazol-2-ylidene] with **1-Ln** (Ln = Y, Dy, Yb) were slow and resulted in complicated mixtures. Major products were identified as [Ln(Tp)_3_]^5b,6^ and [(IDipp)Al(N″)(H)_2_] (**Al-IDipp**; sections A4.1 and B4.4).^7^ In contrast, the reactions of **1-Ln** (Ln = Y, Sm, Dy, Yb) with aminoalane [Me_3_N·AlH_3_] in toluene resulted in the evolution of NMe_3_ gas and the formation of Ln/Al heterobimetallic trihydride complexes [Ln(Tp)_2_(μ-H)_2_Al(H)(N″)] (**2-Ln**; Ln = Y, 95%; Sm, 50%; Dy, 92%; Yb, 94%; Scheme 1a).

**Scheme 1:**
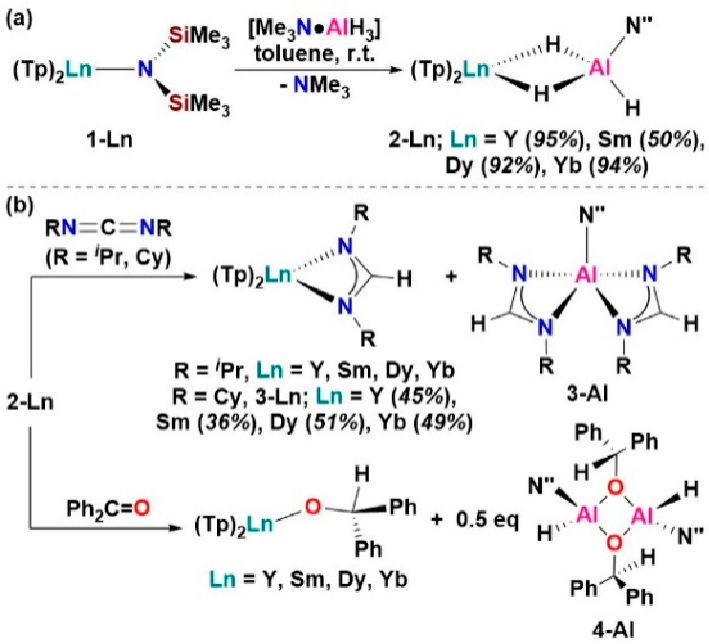
(a) Synthesis of Ln/Al Heterobimetallic Trihydride Complexes **2-Ln** and (b) Substrate Insertion Reactivity of **2-Ln** with Carbodiimides and Benzophenone

Multinuclear NMR data for the diamagnetic **2-Y** and paramagnetic **2-Ln** (Ln = Sm, Dy, Yb) complexes can be readily assigned by relative integration (Table S1) and are comparable to the literature.^5^ Amide (N″) transfer from Ln to Al is confirmed by ^1^H and ^29^Si{^1^H} INEPT NMR spectroscopy because the N″ shifts downfield in line with transfer to the more electronegative element [e.g., 0.13 and −11.96 ppm (**1-Y**) to 0.25 and −2.38 ppm (**2-Y**), respectively]. The metal hydrides of **2-Ln** are observed at 5.07 ppm (**2-Y**), 0.24 ppm (**2-Sm**), and −92.98 ppm (**2-Yb**) in the ^1^H NMR spectrum, with no hydride resonances observed for **2-Dy**. Variable-temperature (VT) NMR studies of **2-Y** (Figure S23) maintained the magnetically equivalent hydride environment across the temperature range of 220–353 K, suggesting fast exchange of the hydrides in solution. The Fourier transform infrared (FT-IR) spectra of **2-Ln** showed weak absorptions at 2450–2470 cm^–1^, which are characteristic of the Tp ligand borohydride stretching frequencies, and weak absorptions between 1800 and 1810 cm^–1^ and between 1730 and 1740 cm^–1^, consistent with the terminal and bridging hydrides, respectively, reported for Ln/Al heterobimetallic hydrides.^1a^

Colorless single crystals of **2-Ln** suitable for single-crystal X-ray diffraction (SCXRD) were grown from either saturated hexane solutions (Ln = Y, Dy) or saturated toluene solutions in the presence of hexane antisolvent (Ln = Yb) at −35 °C. The structures of **2-Ln** are all monomeric with an 8-coordinate Ln(III) ion bound to two κ^3^-coordinated Tp ligands and two bridging hydrides, which are arranged around the Ln(III) ion in a distorted square-antiprismatic geometry [**2-Ln** = Dy (Figure 2), Y, Yb, and Sm (Figures S110–S112); see section B4 for structural comparisons]. Two hydrides bridge the Ln(III) ion to a tetrahedral Al(III) ion, which is also bound to a terminal hydride and the N″ anion. Bridging and terminal hydrides were found and refined freely, with the Ln–H–Al bond distances comparable to previously reported Ln/Al heterobimetallic hydrides.^1a^ The Ln···Al separation is similar across the **2-Ln** series [**2-Dy** 3.1826(6), **2-Y** 3.1822(5), and **2-Yb** 3.1439(9) Å]. The close Ln–Al separation (approximately the sum of the Ln and Al covalent radii) is indicative of strong Ln–(μ-H)–Al bonding, consistent with previous bridging hydrides and three-center, two-electron bonds.^8^ The closest structural comparator of **2-Ln** is [**V**], which exhibits the same μ^2^ binding mode of the bridging hydrides.^4^ Notably, **2-Dy** is the first crystallographically characterized example of the Dy–(μ-H)–Al unit. The Ln–N(κ^3^-Tp) bond distances in **2-Ln** are consistent with those of **1-Ln** and [Ln(Tp)_2_]^+^ in the literature.^5^ The Al–N(N″) bond distances of 1.8430(19)–1.844(3) Å in **2-Ln** are similar to those in [**V**] [1.837(2) Å],^4^ TM/Al heterobimetallic hydrides containing TM–(μ-H)_2_–Al(N″)_*n*_,^9^ and the homoleptic complex [Al(N″)_3_] [1.813(3) Å].^10^

**Figure 2:**
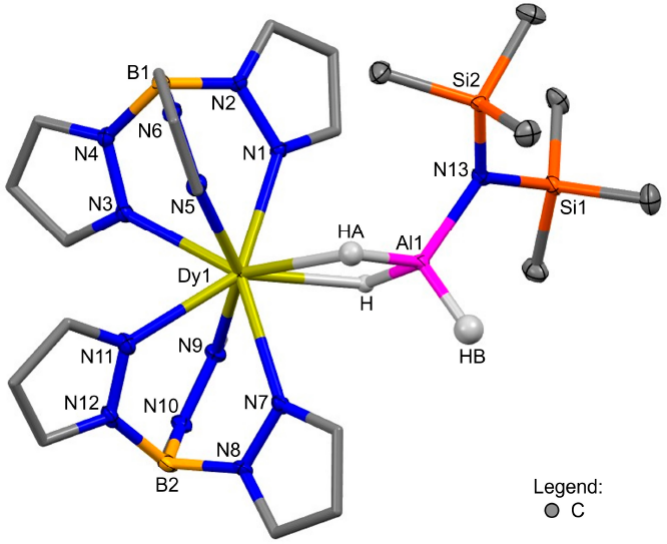
Molecular structure of **2-Dy**. Hydrogen atoms, except for H, HA, and HB, are omitted for clarity, and the pyrazolyl carbon atoms of Tp are displayed in wireframe. Displacement ellipsoids are drawn at 50% probability.

The reactivity scope of **2-Ln** was investigated to understand the nature of the heterobimetallic hydride. The reaction of **2-Ln** with 1 equiv of IDipp again led to the formation of **Al-IDipp**(7) and multiple products. Importantly, no discrete Ln homometallic hydrides, putative “[{Ln(Tp)_2_(μ-H)}_2_]”, were observed spectroscopically (section B2.2). Previously, the reaction chemistry of Ln(L)_2_(μ-X)_2_AlX_2_ (L = ligand; X = monoanionic substituent, e.g., H, CH_3_) showed that these complexes are best described as an ion pair, [Ln(L)_2_]^+^[AlX_4_]^−^, because they undergo facile metathesis reactions.^11^ As such, we examined the potential ion-pair character of **2-Ln**. The reaction of **2-Ln** with K(N″) should yield the starting material **1-Ln**; however, this resulted in complicated reaction mixtures containing **1-Ln**, [Ln(Tp)_3_],^5b,6^ [{K(μ-N″)(μ-H)Al(H)_2_}_*n*_],^12^ HN″, and H_2_ gas and, in addition for **2-Yb**, reduction to [Yb(Tp)_2_]^13^ (sections B2.3–B2.7). Complexes **2-Ln** do not undergo facile ion exchange, indicating that the strong Ln–(μ-H)–Al interaction persists in solution (consistent with the VT NMR data). Thus, **2-Ln** should be considered a bimetallic hydride.

To prove this hypothesis, we studied the insertion chemistry of **2-Ln** toward unsaturated substrates. Amides **1-Ln** do not react with carbodiimides to yield the putative guanidinates “[Ln(Tp)_2_{κ^2^-(R)NC(N″)N(R)}]”. The reaction of RN=C=NR (R = ^*i*^Pr or Cy) with **2-Ln** in benzene-*d*_6_ (Scheme 1b) resulted in formation of the formamidinate complexes [Ln(Tp)_2_{κ^2^-(R)NCHN(R)}] (**3-Ln**) and [Al{κ^2^-(R)NCHN(R)}_2_(N″)] (**3-Al**) (sections B2.10–B2.13). The ^1^H NMR resonances of **3-Al** are comparable to those of [Al{κ^2^-(R)NCHN(R)}_2_(X)] (X = hydride, halide) in the literature.^14^ Preparative-scale reactions allowed for the separation of **3-Al**, and complexes **3-Ln** (R = Cy) were isolated in moderate yields (Y, 45%; Sm, 36%; Dy, 51%; Yb, 49%; Scheme 1b). Multinuclear NMR data for diamagnetic **3-Y** and paramagnetic **3-Ln** (Ln = Sm, Dy, or Yb) complexes can be readily assigned by relative integration. The FT-IR spectra of **3-Ln** (Ln = Dy, Yb) confirm the transfer of the hydride to the carbodiimide because strong absorptions between 1556 and 1559 cm^–1^ are assigned to the N–C(H)–N linkage stretching frequencies (ν_NCN_) in **3-Ln**. ν_NCN_ in **3-Ln** is lower in wavenumber compared to ν_NCN_, 1650–1670 cm^–1^, as observed for homoleptic [Ln(*N*,*N*′-R_2_-formamidinate)_3_]^15^ and heteroleptic [Ln(*N*,*N*′-R_2_-formamidinate)_2_(X)] complexes, with X monoanions and aromatic R groups.^16^

Yellow crystals of **3-Ln** (Ln = Dy, Yb) suitable for SCXRD were grown from saturated toluene solutions in the presence of a hexane antisolvent at −35 °C. Both **3-Yb** (Figure 3) and **3-Dy** (Figure S113) structures are monomeric, containing an 8-coordinate Ln(III) ion bound to two κ^3^-coordinated Tp ligands and a κ^2^-coordinated bidentate (Cy)NCHN(Cy) anion, arranged around the Ln(III) ion in a distorted square-antiprismatic geometry. The reduction of the carbodiimide was confirmed by the presence of H10 on the sp^2^ carbon (C10) in the now notably bent formamidinate fragment [N7–C10–N7′ 118.6(15)° (**3-Dy**) and 119.8(6)° (**3-Yb**)]. A comparison of the C–N bond lengths also confirms the reduced nature of the formamidinate fragment because C10–N7 falls between the typical values for C–N single and double bonds [C10–N7 1.326(14) Å (**3-Dy**) and 1.315(5) Å (**3-Yb**) vs single (∼1.47 Å) and double (∼1.15 Å) C–N bonds], consistent with delocalization of the N–C–N fragment in **3-Ln**.^17^ The Ln–N(κ^3^-Tp) bond distances in **3-Ln** are consistent with those of **1-Ln**, **2-Ln**, and [Ln(Tp)_2_(X)] in the literature.^5b,6^ The Ln–N7 bond distances in **3-Ln** [Ln = Dy, 2.376(8) Å; Yb, 2.373(3) Å] are consistent with the Ln–N bond distances in related literature complexes.^15,16,18^

**Figure 3:**
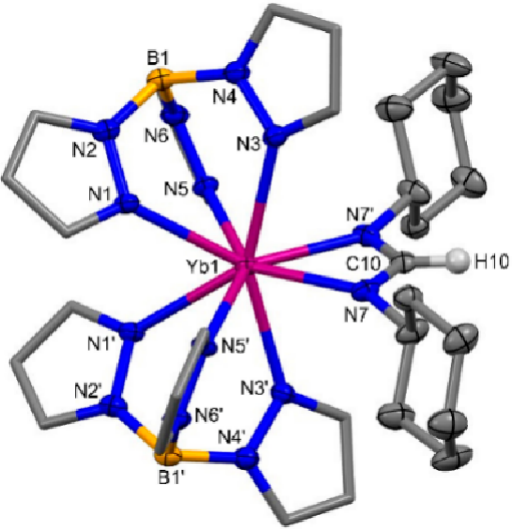
Molecular structure of **3-Yb**. Hydrogen atoms except H10 are omitted for clarity, and the pyrazolyl carbon atoms of Tp are displayed in wireframe. Displacement ellipsoids are drawn at 50% probability.

Notably, **2-Ln** behaves differently from the TM/Al heterobimetallic trihydride complex [Ti(Cp)_2_(μ-H)_2_Al(H){C(SiMe_3_)_3_}] because reaction with excess ^*i*^PrN=C=N^*i*^Pr resulted in a formamidinate-bridged Ti/Al complex, [Ti(Cp)_2_(μ-H){μ-(^i^Pr)N(CH)N(^*i*^Pr)}Al(H){C(SiMe_3_)_3_}].^19^ Analogous to the insertion chemistry of carbodiimides, the insertion of sterically hindered carbonyls like ketones is common in both Ln–H and MG–H chemistry.^1b,1d,1h,12b^ NMR-scale reactions of **2-Ln** with 3 equiv or more of benzophenone in benzene-*d*_6_ yielded the major products [Ln(Tp)_2_(OCHPh_2_)] and dimeric [{Al(N″)(H)(μ-O(CHPh_2_))}_2_] (**4-Al**) (see sections B2.14–B2.17).

Inspired by the Ln/Al heterobimetallic hydride hydroalumination catalyst [**V**] (Figure 1),^4^ the applicability of **2-Ln** in catalysis was examined to establish the cooperative behavior between the two metal centers. The catalytic dehydrocoupling of [Me_2_HN·BH_3_] has attracted significant interest due to hydrogen storage applications and as precursors to boron nitride materials.^20^ Ln and Al hydrides have both been reported to catalyze the dehydrocoupling of [Me_2_HN·BH_3_] at elevated temperatures, thus providing a good basis for this preliminary study.^1e,21^ The dehydrocoupling of [Me_2_HN·BH_3_] by 10 mol % **2-Ln** (Ln = Y, Sm, Dy, Yb) proceeds at room temperature to yield [Me_2_N·BH_2_]_2_ as the major product (see section C). Notable differences were found across the **2-Ln** series, with consumption of amine–borane occurring most efficiently in the order Yb > Y ∼ Dy > Sm, in line with the relative Lewis acidity of the Ln(III) ion. It is of note that **1-Y** and [Me_3_N·AlH_3_] do not catalyze this reaction under these conditions; therefore, cooperativity between the two metal centers enables this transformation to occur at room temperature.

In conclusion, the facile synthesis and characterization of Ln/Al heterobimetallic trihydride complexes **2-Ln** (Ln = Y, Sm, Dy, Yb) are reported. The reactivity of **2-Ln** was investigated to understand the cooperative nature of the two different metal centers. Attempts to break apart **2-Ln** by the addition of Lewis bases or ion-exchange chemistry resulted in multiple products. However, upon reaction with unsaturated organic substrates, clean conversion to discrete Ln(III) and Al(III) hydride insertion products was obtained. Highlighting this is the fact that **2-Ln** is best described as a bimetallic hydride with a strong Ln–(μ-H)–Al interaction. Preliminary catalytic investigations of **2-Ln** also showed heterobimetallic cooperativity, enabling the dehydrocoupling of [Me_2_HN·BH_3_] at room temperature. Exploration of the reactivity and catalytic activity of these Ln/Al heterobimetallic trihydrides is ongoing to fully understand and utilize the synergistic metal–metal interaction.

## Data Availability

Additional data that support the findings of this study are available from the University of Strathclyde KnowledgeBase at 10.15129/f6a428f0-48e2-400e-81c2-465639e81845.

## Supporting Information Available

Data that support the findings of this study and additional references^22^ (PDF)
