# Supplementary material for: Synthesis and Reactivity of Bis-tris(pyrazolyl)borate Lanthanide/Aluminum Heterobimetallic Trihydride Complexes
Source: Inorg Chem. 2024 Apr 29;63(21):9390–4. doi: 10.1021/acs.inorgchem.4c00824 (PMC11134492; doi:10.1021/acs.inorgchem.4c00824)
Supplement: Supplementary file 1 — ic4c00824_si_001.pdf [file ic4c00824_si_001.pdf]

# Synthesis and reactivity of *bis*-tris(pyrazolyl)borate lanthanide-aluminium heterobimetallic trihydride complexes

Tajrian Chowdhury,[a] Fáinché Murphy,[b] Alan R. Kennedy,[b] Claire Wilson,[a] Joy H. Farnaby,*[a] Catherine E. Weetman*[b]

[a] School of Chemistry, Joseph Black Building, University of Glasgow, Glasgow, G12 8QQ, UK.

[b] Department of Pure and Applied Chemistry, University of Strathclyde, 295 Cathedral Street, Glasgow, G1 1XL, UK.

Corresponding author(s): * joy.farnaby@glasgow.ac.uk;
* catherine.weetman@strath.ac.uk.

## Supporting Information

# Contents

**Experimental** .......... **5**
General Experimental Considerations .......... 5
Physical Methods .......... 6
**A. Synthesis of complexes** .......... **7**
**A1 Synthesis of [Sm(Tp)$_2$(OTf)] Sm-OTf and [Sm(Tp)$_2$(N")] 1-Sm** .......... **7**
A1.1 Synthesis of Sm(OTf)$_3$ .......... 7
A1.2 Synthesis of [Sm(Tp)$_2$(OTf)] **Sm-OTf** .......... 7
A1.3 Synthesis of [Sm(Tp)$_2$(N")] **1-Sm** .......... 8
**A2 Synthesis of [Ln(Tp)$_2$($\mu$-H)$_2$Al(H)(N")] 2-Ln (Ln = Y, Sm, Dy, Yb)** .......... **9**
A2.1 Synthesis of [Y(Tp)$_2$($\mu$-H)$_2$Al(H)(N")] **2-Y** .......... 9
A2.2 Synthesis of [Sm(Tp)$_2$($\mu$-H)$_2$Al(H)(N")] **2-Sm** .......... 10
A2.3 Synthesis of [Dy(Tp)$_2$($\mu$-H)$_2$Al(H)(N")] **2-Dy** .......... 10
A2.4 Synthesis of [Yb(Tp)$_2$($\mu$-H)$_2$Al(H)(N")] **2-Yb** .......... 11
**A3 Heating [Y(Tp)$_2$($\mu$-H)$_2$Al(H)(N")] 2-Y under dynamic vacuum at 80 °C** .......... **12**
**A4 Reaction between [Yb(Tp)$_2$(N")] 1-Yb with [IDipp•AlH$_3$] and reaction between [Yb(Tp)$_2$($\mu$-H)$_2$Al(H)(N")] 2-Yb with IDipp (1,3-*bis*(2,6-diisopropylphenyl)imidazol-2-ylidene)** .......... **12**
A4.1 Isolation of crystals of [(IDipp)Al(N")(H)$_2$] **Al-IDipp** in the reaction between [Yb(Tp)$_2$(N")] **1-Yb** with [IDipp•AlH$_3$] .......... 12
A4.2 NMR-scale reaction between [Yb(Tp)$_2$($\mu$-H)$_2$Al(H)(N")] **2-Yb** with IDipp .......... 13
**A5 NMR-scale reaction between [Me$_3$N•AlH$_3$] with K(N") and NMR-scale reactions between [Ln(Tp)$_2$($\mu$-H)$_2$Al(H)(N")] 2-Ln (Ln = Y, Sm, Dy, Yb) with K(N")** .......... **13**
A5.1 NMR-scale reaction of between [Me$_3$N•AlH$_3$] with K(N") .......... 13
A5.2 NMR-scale reactions between [Ln(Tp)$_2$($\mu$-H)$_2$Al(H)(N")] **2-Ln** (Ln = Y, Sm, Dy, Yb) with K(N") .......... 13
**A6 Synthesis of [Ln(Tp)$_2${$\kappa^2$-(Cy)NCHN(Cy)}] 3-Ln (Ln = Dy, Yb)** .......... **14**
A6.1 Synthesis of [Dy(Tp)$_2${$\kappa^2$-(Cy)NCHN(Cy)}] **3-Dy** .......... 14
A6.2 Synthesis of [Yb(Tp)$_2${$\kappa^2$-(Cy)NCHN(Cy)}] **3-Yb** .......... 14
**A7 NMR-scale reactions between [Ln(Tp)$_2$($\mu$-H)$_2$Al(H)(N")] 2-Ln (Ln = Y, Sm) with three equivalents of CyN=C=NCy (Cy = $C_6H_{11}$, DCC)** .......... **15**
A7.1 NMR-scale reaction between [Y(Tp)$_2$($\mu$-H)$_2$Al(H)(N")] **2-Y** with three equivalents of CyN=C=NCy (DCC, Cy = $C_6H_{11}$) .......... 15
A7.2 NMR-scale reaction between [Sm(Tp)$_2$($\mu$-H)$_2$Al(H)(N")] **2-Sm** with three equivalents of CyN=C=NCy (DCC, Cy = $C_6H_{11}$) .......... 16
**A8 NMR-scale reactions between [Ln(Tp)$_2$($\mu$-H)$_2$Al(H)(N")] 2-Ln (Ln = Y, Sm, Dy, Yb) with excess $^i$PrN=C=N$^i$Pr (DIC)** .......... **16**

**A9 NMR-scale reactions between [Ln(Tp)$_2$($\mu$-H)$_2$Al(H)(N")] 2-Ln (Ln = Y, Sm, Dy, Yb) with benzophenone (Ph$_2$C=O) ......17**
A9.1 NMR-scale reactions between [Ln(Tp)$_2$($\mu$-H)$_2$Al(H)(N")] **2-Ln** (Ln = Y, Yb) with sequential addition of one, two, and three equivalents of benzophenone ......17
A9.2 NMR-scale reactions between [Ln(Tp)$_2$($\mu$-H)$_2$Al(H)(N")] **2-Ln** (Ln = Sm, Dy) with direct addition of three equivalents of benzophenone ......17
**A10 Isolation of single-crystals of [{Al(N")(H)($\mu$-O(CHPh$_2$))}$_2$] 4-Al from reactions between [Ln(Tp)$_2$($\mu$-H)$_2$Al(H)(N")] 2-Ln (Ln = Y, Sm, Dy, Yb) with benzophenone (Ph$_2$C=O) ......18**
**B. Spectroscopic Data for Complexes ......19**
**B1 Nuclear magnetic resonance (NMR) data for isolated compounds ......19**
B1.1 Sm(OTf)$_3$ ......19
B1.2 [Sm(Tp)$_2$(OTf)] **Sm-OTf** ......19
B1.3 [Sm(Tp)$_2$(N")] **1-Sm** ......23
B1.4 $^{29}$Si{$^1$H} INEPT NMR data for [Ln(Tp)$_2$(N")] **1-Ln** (Ln = Y, Sm, Dy, Yb) ......27
B1.5 [Y(Tp)$_2$($\mu$-H)$_2$Al(H)(N")] **2-Y** ......29
B1.6 [Sm(Tp)$_2$($\mu$-H)$_2$Al(H)(N")] **2-Sm** ......34
B1.7 [Dy(Tp)$_2$($\mu$-H)$_2$Al(H)(N")] **2-Dy** ......38
B1.8 [Yb(Tp)$_2$($\mu$-H)$_2$Al(H)(N")] **2-Yb** ......39
B1.9 [(IDipp)Al(N")(H)$_2$] **Al-IDipp** ......41
B1.10 [Dy(Tp)$_2${$\kappa^2$-(Cy)NCHN(Cy)}] **3-Dy** ......42
B1.11 [Yb(Tp)$_2${$\kappa^2$-(Cy)NCHN(Cy)}] **3-Yb** ......43
**B2 NMR data for NMR-scale reactions ......45**
B2.1 Heating [Y(Tp)$_2$($\mu$-H)$_2$Al(H)(N")] **2-Y** under dynamic vacuum at 80 °C ......45
B2.2 NMR-scale reaction between [Yb(Tp)$_2$($\mu$-H)$_2$Al(H)(N")] **2-Yb** with IDipp ......46
B2.3 NMR-scale reaction between [Me$_3$N•AlH$_3$] with K(N") ......47
B2.4 NMR-scale reaction between [Y(Tp)$_2$($\mu$-H)$_2$Al(H)(N")] **2-Y** with K(N") ......49
B2.5 NMR-scale reaction between [Sm(Tp)$_2$($\mu$-H)$_2$Al(H)(N")] **2-Sm** with K(N") ......50
B2.6 NMR-scale reaction between [Dy(Tp)$_2$($\mu$-H)$_2$Al(H)(N")] **2-Dy** with K(N") ......51
B2.7 NMR-scale reaction between [Yb(Tp)$_2$($\mu$-H)$_2$Al(H)(N")] **2-Yb** with K(N") ......52
B2.8 NMR-scale reaction between [Y(Tp)$_2$($\mu$-H)$_2$Al(H)(N")] **2-Y** with three equivalents of CyN=C=NCy (Cy = C$_6$H$_{11}$, DCC) and purified by washing [Al{$\kappa^2$-(Cy)NCHN(Cy)}$_2$(N")] **3-Al** away with hexane ......54
B2.9 NMR-scale reaction between [Sm(Tp)$_2$($\mu$-H)$_2$Al(H)(N")] **2-Sm** with three equivalents of CyN=C=NCy (Cy = C$_6$H$_{11}$, DCC) and purified by washing [Al{$\kappa^2$-(Cy)NCHN(Cy)}$_2$(N")] **3-Al** away with hexane ......56
B2.10 NMR-scale reaction between [Y(Tp)$_2$($\mu$-H)$_2$Al(H)(N")] **2-Y** with excess $^i$PrN=C=N$^i$Pr (DIC) ......58

B2.11 NMR-scale reaction between [Sm(Tp)$_2$($\mu$-H)$_2$Al(H)(N")] **2-Sm** with excess $^i$PrN=C=N$^i$Pr (DIC) ........ 59
B2.12 NMR-scale reaction between [Dy(Tp)$_2$($\mu$-H)$_2$Al(H)(N")] **2-Dy** with excess $^i$PrN=C=N$^i$Pr (DIC) ........ 61
B2.13 NMR-scale reaction between [Yb(Tp)$_2$($\mu$-H)$_2$Al(H)(N")] **2-Yb** with excess $^i$PrN=C=N$^i$Pr (DIC) ........ 62
B2.14 NMR-scale reaction between [Y(Tp)$_2$($\mu$-H)$_2$Al(H)(N")] **2-Y** with sequential addition of one, two, and three equivalents of benzophenone ........ 64
B2.15 NMR-scale reaction between [Yb(Tp)$_2$($\mu$-H)$_2$Al(H)(N")] **2-Yb** with sequential addition of one, two, and three equivalents of benzophenone ........ 66
B2.16 NMR-scale reaction between [Sm(Tp)$_2$($\mu$-H)$_2$Al(H)(N")] **2-Sm** with three equivalents of benzophenone ........ 68
B2.17 NMR-scale reaction between [Dy(Tp)$_2$($\mu$-H)$_2$Al(H)(N")] **2-Dy** with three equivalents of benzophenone ........ 69
**B3 Infrared (IR) data ........ 72**
B3.1 ATR-IR data of Sm(OTf)$_3$, [Sm(Tp)$_2$(OTf)] **Sm-OTf**, and [Sm(Tp)$_2$(N")] **1-Sm** ........ 72
B3.2 FTIR data of [Ln(Tp)$_2$($\mu$-H)$_2$Al(H)(N")] **2-Ln** (Ln = Y, Sm, Dy, Yb) ........ 73
B3.3 FTIR data of [Ln(Tp)$_2${$\kappa^2$-(Cy)NCHN(Cy)}] **3-Ln** (Ln = Dy, Yb) ........ 75
**B4 Single-crystal X-Ray diffraction data ........ 77**
B4.1 [Sm(Tp)$_2$(OTf)] **Sm-OTf** and [Sm(Tp)$_2$(N")] **1-Sm** ........ 80
B4.2 [Ln(Tp)$_2$($\mu$-H)$_2$Al(H)(N")] **2-Ln** (Ln = Y, Dy, Yb) ........ 81
B4.3 [Ln(Tp)$_2${$\kappa^2$-(Cy)NCHN(Cy)}] **3-Ln** (Ln = Dy, Yb) ........ 82
B4.4 [(IDipp)Al(N")(H)$_2$] **Al-IDipp** and [{Al(N")(H)($\mu$-O(CHPh$_2$))}$_2$] **4-Al** ........ 83
**C. Catalytic dehydrocoupling of dimethylaminoborane [Me$_2$HN•BH$_3$] by [Ln(Tp)$_2$($\mu$-H)$_2$Al(H)(N")] 2-Ln (Ln = Y, Sm, Dy, Yb) at ambient temperature ........ 84**
C1 Methodology for catalytic runs ........ 84
C2 Data for catalytic runs ........ 84
**D. References ........ 86**

# Experimental

## General Experimental Considerations

All air-sensitive manipulations were carried out in an MBraun glovebox (inert atmosphere of $N_2$, $O_2$ <0.1 ppm, $H_2O$ <0.1 ppm or inert atmosphere of argon, $O_2$ <10.0 ppm, $H_2O$ <0.5 ppm) or by using standard Schlenk techniques under $N_2$. All glassware was dried at 130-150 °C overnight, in a Binder ED53 Drying Oven/Hot Air Steriliser, prior to use. Filter cannulas were prepared using Whatman 25 mm glass microfiber filters and were pre-dried at 130-135 °C overnight. An Innovative Technology Inc. Pure Solv 400-5-MD solvent purification system (activated alumina columns) was used to obtain anhydrous toluene, tetrahydrofuran (THF), and acetonitrile (MeCN). Anhydrous hexane (95%) was purchased from Merck or Fisher Scientific UK Ltd. Anhydrous solvents were degassed, sparged with $N_2$, and stored in ampoules over activated 3.0 Å or 4.0 Å molecular sieves (25-35% weight by volume) under $N_2$/argon. Absence of water/levels of residual water in bulk solvents were confirmed by using a sodium benzophenone ketyl solution in THF after 24-48 hours. Deuterated benzene ($d_6$-benzene) was dried by directly transferring from sealed glass ampoules onto activated 3.0 Å or 4.0 Å molecular sieves and stored in ampoules in a $N_2$/argon atmosphere glovebox. Deuterated toluene ($d_8$-toluene) was degassed by three freeze-pump-thaw degassing cycles and dried by storing in ampoules over activated 3.0 Å molecular sieves in $N_2$/argon atmosphere glovebox. Deuterated acetonitrile was dried by refluxing over $CaH_2$, degassed by three freeze-pump-thaw degassing cycles, filtered across a frit coated with Celite® into a Büchner flask, to exclude excess $CaH_2$ and stored in ampoules over activated 3.0 Å molecular sieves in a $N_2$ atmosphere glovebox. Absence of water in deuterated solvents was confirmed by $^1H$ NMR after 48 hours. Potassium hydride (KH) in mineral oil was purchased from Merck under mineral oil, then washed with anhydrous hexanes, dried *in vacuo* over a frit and stored in a $N_2$ atmosphere glovebox. Potassium hydrotris(1-pyrazolyl)borate K(Tp),[1] K(N'') (N'' = $N(SiMe_3)_2$),[1] $Ln(OTf)_3$ (OTf = $CF_3SO_3$),[1-2] $[Ln(Tp)_2(X)]$ (X = OTf, N'' ; Ln = Y, Dy, Yb),[1-2] $[Me_3N{\bullet}AlH_3]$,[3] and $[IDipp{\bullet}AlH_3]$ (IDipp = 1,3-*bis*(2,6-diisopropylphenyl)imidazol-2-ylidene),[4] were synthesised according to literature procedures. The following starting materials were used without further purification: from Alfa Aesar, $Ln_2O_3$ (Ln = Y, Sm, Dy, Yb), pyrazole, and benzophenone (dried *in vacuo* prior to use); from Merck, trifluoromethanesulfonic acid, deuterated solvents, $LiAlH_4$, $CaH_2$, *N,N'*-dicyclohexylcarbodiimide or DCC (dried *in vacuo* prior to use), *N,N'*-diisopropylcarbodiimide or DIC (sparged with $N_2$ and dried over activated 4.0 Å molecular sieves), $[Me_2HN{\bullet}BH_3]$ (sublimed prior to use and stored at -24 °C subsequently in an $N_2$/argon atmosphere glovebox); and from Fluorochem, $KBH_4$. Bench solvents and ammonium chloride were purchased from Fisher Scientific UK Ltd.

**Physical Methods**

*NMR Spectroscopy:*

NMR data were recorded on an AVIII 400 MHz spectrometer (either with or without a cryoprobe) operating at frequencies 400.1 MHz ($^1$H), 100.6 MHz ($^{13}$C{$^1$H}), 376.5 MHz ($^{19}$F), 128.4 MHz ($^{11}$B and $^{11}$B{$^1$H}), 79.5 MHz ($^{29}$Si{$^1$H} Insensitive nuclei enhanced by polarization transfer or INEPT). Variable temperature (VT) $^1$H NMR data were recorded in $d_8$-toluene, with assistance from Craig Irving at the University of Strathclyde, on an AVI 400 MHz spectrometer. The NMR data were referenced internally to the appropriate residual proteo-solvent and reported relative to tetramethylsilane ($\delta$ = 0 ppm) for $^1$H, $^{13}$C{$^1$H} and $^{29}$Si{$^1$H} NMR. $^{19}$F NMR data were reported relative to $CFCl_3$ ($\delta$ = 0 ppm) and $^{11}$B and $^{11}$B{$^1$H} NMR data were reported relative to 15% $BF_3$•$OEt_2$ in $CDCl_3$ ($\delta$ = 0 ppm). All spectra were recorded at a constant temperature of 25-27 °C (298-300 K). Coupling constants (*J*) are reported in hertz (Hz). Standard abbreviations for multiplicity were used as follows: m = multiplet, t = triplet, d = doublet, s = singlet. For broad intensities, abbreviated as br, the full width at half-maximum intensity (FWHM) is provided in Hz.

*IR Spectroscopy:*

ATR-IR spectra were collected in air at ambient temperature using ThermoFisher Scientific Nicolet Summit LITE FTIR Spectrometer (containing a $LiTaO_3$ detector) equipped with Everest ATR. FTIR spectra were collected at ambient temperature using PerkinElmer Spectrum 100 FTIR Spectrometer by first preparing a paste of the compound in nujol mull between NaCl discs inside an argon glovebox. Abbreviations for the intensity of stretching frequencies were used as follows: s = strong, m = medium, w = weak.

*Single Crystal X-Ray Diffraction:*

Single-crystal X-ray diffraction data for **Sm-OTf**, **1-Sm**, and **Al-IDipp** were collected at 150 K using Mo-$K_\alpha$ radiation ($\lambda$ = 0.71073 Å) on a Bruker D8 VENTURE diffractometer equipped with a Photon II CPAD detector, with Oxford Cryosystems n-Helix device mounted on an IμS 3.0 (dual Cu and Mo) microfocus sealed tube generator. This data was processed using APEX3 and SAINT.[8] All other data was measured using a Rigaku Synergy-i instrument and Cu-$K_\alpha$ radiation ($\lambda$ = 1.54184 Å) and processed using CrysalisPro.[9] All structures were refined to convergence using all unique reflections and against $F^2$ using the program SHELXL-2018 as implemented within Olex2[10,11]. Structure **3-Dy** was treated as a twin and refined against an hklf 5 formatted reflection file. Here the individual twin contributions refined to 0.755(2) and 0.245(2). The hexane solvent of **Al-IDipp** was refined as disordered over two sites. Appropriate restraints and constraints were applied to this disordered group to ensure that its geometric and displacement parameters approximated to normal behaviour. Crystallographic figures are presented as **Figure S 108** to **Figure S 116** and selected geometric parameters are given in **Table S 2**. Selected crystallographic and refinement parameters are given in **Table S 3** and full details have been deposited with the CCDC crystallographic database

in cif format. CCDC numbers 2313687-2313695 contain the crystallographic information for this paper.

*Elemental Analysis:*

Elemental analyses were performed by Orla McCullough at the London Metropolitan University, using a Flash 2000 Organic Elemental Analyzer, Thermo Scientific analyser. The samples for the measurements were prepared using $V_2O_5$ (to ensure complete combustion of all complexes) in tin capsules inside an inert argon glovebox atmosphere.

# A. Synthesis of complexes

## A1 Synthesis of [Sm(Tp)$_2$(OTf)] Sm-OTf and [Sm(Tp)$_2$(N")] 1-Sm

### A1.1 Synthesis of Sm(OTf)$_3$

Sm(OTf)$_3$ was synthesised according to literature,[1-2] using the following materials and quantities: $Sm_2O_3$ (3.49 g, 10.0 mmol, 1.0 eq), HOTf (10 g, 66.6 mmol, 6.66 eq). Yield: 4.93 g, 8.25 mmol, 41%.

$^{19}$F NMR ($d_3$-MeCN): $\delta$ -79.15 (s, OTf-C**F$_3$**) ppm.

Anal. Calcd. for $C_3S_3F_9O_9Sm$: C, 6.03%; H, 0.00%; N, 0.00%. Found: C, 6.60%; H, 0.00%; N, 0.00%.

IR (ATR): 1345 (w), 1284 (s), 1233 (s), 1198 (s), 1161 (s), 1030 (s), 777 (w), 626 (s), 580 (m), 508 (s) cm$^{-1}$.

### A1.2 Synthesis of [Sm(Tp)$_2$(OTf)] Sm-OTf

[Sm(Tp)$_2$(OTf)] **Sm-OTf** was synthesised by an adaptation of literature procedure.[1] A 250 mL rotaflo-tap reaction ampoule was charged with a stirrer bar and the white powders Sm(OTf)$_3$ (0.831 g, 1.39 mmol, 1.0 eq) and K(Tp) (0.700 g, 2.78 mmol, 2.0 eq). THF (60 mL) was added, and the resultant white suspension was stirred at ambient temperature for 4 hours. THF was removed *in vacuo* and the white solids yielded were dried (10$^{-2}$ mbar, 2 h). Toluene (150 mL) was added, and the ampoule was partially evacuated, and the white suspension was refluxed (130 °C, 17 h). The hot suspension was allowed to settle and heated at 120 °C for 0.25 h, after which it was filtered across a frit coated with Celite®, to exclude K(OTf). Toluene was removed *in vacuo* from the filtrate and the white solids were dried (10$^{-2}$ mbar, 2 h). The solids were washed with hexane (3 x 15 mL), ground into a fine white powder and dried *in vacuo* (130 °C, 10$^{-2}$ mbar, 18 h) yielding [Sm(Tp)$_2$(OTf)] **Sm-OTf** (0.772 g, 1.06 mmol, 77%).

Compound **Sm-OTf** has excellent solubility in THF, moderate solubility in toluene, and poor solubility in hexane. Lath-shaped colourless single-crystals of the THF-adduct of **Sm-OTf** [Sm(Tp)$_2$(OTf)(THF)] suitable for X-ray diffraction were grown from a saturated THF solution with a hexane antisolvent at -35 °C over a week.

$^{1}$H NMR ($d_3$-MeCN): $\delta$ 4.47 (6H, d, $^{3}J_{H-H}$ = 1.4 Hz, Tp-C$^{3/5}$**H**) 6.05 (6H, t, $^{3}J_{H-H}$ = 1.9 Hz, Tp-C$^{4}$**H**) 7.22 (2H, very br m, FWHM = 322.0 Hz, Tp-B**H**) 8.88 (6H, d, $^{3}J_{H-H}$ = 2.2 Hz, Tp-C$^{3/5}$**H**) ppm; $^{13}$C{$^{1}$H} NMR ($d_3$-MeCN): $\delta$ 105.0 (s, Tp-**C$^{4}$**) 137.8 (s, Tp-**C$^{3/5}$**) 142.2 (s, Tp-**C$^{3/5}$**) ppm; $^{19}$F NMR ($d_3$-MeCN): $\delta$ -78.67 (s, OTf-C**F$_3$**) ppm; $^{11}$B NMR ($d_3$-MeCN): $\delta$ 2.86 (d, $^{1}J_{B-H}$ = 100.7 Hz, Tp-**B**) ppm; $^{11}$B{$^{1}$H} NMR ($d_3$-MeCN): $\delta$ 2.91 (s, Tp-**B**) ppm.

Anal. Calcd. for $C_{19}H_{20}B_2F_3N_{12}O_3SSm$: C, 31.46%; H, 2.78%; N, 23.17%. Found: C, 31.52%; H, 2.82%; N, 21.96%.

IR (ATR): 3131 (w, $\upsilon_{sp^2\text{-}CH}$), 2466 (w, $\upsilon_{BH}$), 1506 (m, $\upsilon_{C=C}$) cm$^{-1}$.

### A1.3 Synthesis of [Sm(Tp)$_2$(N'')] 1-Sm

In the glovebox, a 20 mL scintillation vial was charged with a stirrer bar, and then the white powder [Sm(Tp)$_2$(OTf)] (0.153 g, 0.211 mmol, 1.0 eq) was suspended in toluene (2 mL) and stirred. To this suspension, a colourless solution of KN'' (0.042 g, 0.212 mmol, 1.0 eq) in toluene (1 mL) was added by a pipette dropwise for two minutes with stirring at ambient temperature. This resulted in a cloudy suspension, which was stirred at ambient temperature for an hour after which the resultant pale suspension was filtered across a frit into a Büchner flask, to exclude K(OTf). Further product was extracted from the solids on the frit with toluene (2 mL), the colourless filtrates were combined, and toluene (5 mL) was removed *in vacuo*. Cold (-35 °C) hexane (0.5 mL) was added to precipitate powdery white solids and all solvents were removed *in vacuo* and the resultant white powder was ground up and dried *in vacuo* ($10^{-2}$ mbar, 1.5 h) yielding [Sm(Tp)$_2$(N'')] **1-Sm** (0.126 g, 0.171 mmol, 81%).

Compound **1-Sm** has moderate solubilities in toluene and hexane. Plate-shaped colourless single crystals of **1-Sm** suitable for X-ray diffraction were grown from a saturated hexane solution at -35 °C overnight.

$^{1}$H NMR ($d_6$-benzene): $\delta$ -1.20 (18H, s, FWHM = 5.0 Hz, N''-Si(C**H$_3$**)$_3$) 2.23 (6H, s, FWHM = 9.5 Hz, Tp-C$^{4}$**H**) 5.58 (6H, d, $^{3}J_{H-H}$ = 1.7 Hz, Tp-C$^{3/5}$**H**) 8.64 (2H, very br m, overlapped with the resonance at $\delta$ 9.09 ppm integrating to 6H, FWHM = 281.0 Hz, Tp-B**H**) 9.09 (6H, d, $^{3}J_{H-H}$ = 1.8 Hz, Tp-C$^{3/5}$**H**) ppm; $^{13}$C{$^{1}$H} NMR ($d_6$-benzene): $\delta$ 5.2 (s, N''-Si(**C**H$_3$)$_3$) 103.0 (s, Tp-**C$^{3/5}$**) 136.5 (s, Tp-**C$^{3/5}$**) 141.0 (s, Tp-**C$^{4}$**) ppm; $^{11}$B NMR ($d_6$-benzene): $\delta$ 6.25 (d, $^{1}J_{B-H}$ = 83.6 Hz, Tp-**B**) ppm; $^{11}$B{$^{1}$H} NMR ($d_6$-benzene): $\delta$ 6.26 (s, Tp-**B**) ppm; $^{29}$Si{$^{1}$H} NMR ($d_6$-benzene): $\delta$ -1.34 (s, N''-**Si**(CH$_3$)$_3$) ppm.

Anal. Calcd. for $C_{24}H_{38}B_2N_{13}Si_2Sm$: C, 39.12%; H, 5.20%; N, 24.71%. Found: C, 39.12%; H, 4.27%; N, 24.44%.

IR (ATR): 3143 (w, $\upsilon_{sp^2\text{-}CH}$), 2952 (w, $\upsilon_{sp^3\text{-}CH}$), 2896 (w, $\upsilon_{sp^3\text{-}CH}$), 2452 (w, $\upsilon_{BH}$), 2409 (w, $\upsilon_{BH}$), 2374 (w, $\upsilon_{BH}$), 1500 (m, $\upsilon_{C=C}$) cm$^{-1}$.

## A2 Synthesis of [Ln(Tp)$_2$($\mu$-H)$_2$Al(H)(N")] 2-Ln (Ln = Y, Sm, Dy, Yb)

### A2.1 Synthesis of [Y(Tp)$_2$($\mu$-H)$_2$Al(H)(N")] 2-Y

In the glovebox, a 15 mL scintillation vial was charged with a stirrer bar, and then the white powder [Y(Tp)$_2$(N")] (100.8 mg, 149.3 μmol, 1.0 eq) was dissolved in toluene (1.5 mL) and stirred. To this solution, a colourless solution of [Me$_3$N•AlH$_3$] (13.3 mg, 149.2 μmol, 1.0 eq) in toluene (0.5 mL) was added by a pipette dropwise for two minutes with stirring at ambient temperature. This resulted in a colourless solution and effervescence of colourless NMe$_3$ gas was immediate and the resultant solution was stirred at ambient temperature for 0.5 h and toluene (2 mL) was removed *in vacuo* within the reaction time of 0.5 h, yielding white glassy solids. Hexane (0.5 mL) was added to precipitate powdery white solids, which were washed with hexane (0.5 mL) and the washing subsequently decanted away to remove impurities. All solvents were removed *in vacuo* and the resultant white powder was ground up and dried *in vacuo* ($10^{-2}$ mbar, 1.5 h) yielding [Y(Tp)$_2$($\mu$-H)$_2$Al(H)(N")] **2-Y** (99.7 mg, 141.4 μmol, 95%).

Compound **2-Y** has excellent solubility in toluene and poor solubility in hexane. Block-shaped colourless single crystals of **2-Y** suitable for X-ray diffraction were grown from a saturated hexane solution at -35 °C overnight.

$^1$H NMR ($d_6$-benzene): $\delta$ 0.25 (18H, s, N"-Si(C**H$_3$**)$_3$) 4.70 (2H, very br m, overlapped with the resonance at $\delta$ 5.07 ppm integrating to 5H, FWHM = 416.6 Hz approximately, Tp-B**H**) 5.07 (3H, very br s, overlapped with the resonance at $\delta$ 4.70 ppm integrating to 5H, FWHM = 118.8 Hz, $\mu$-**H$_3$**) 5.78 (6H, t, $^3J_{H-H}$ = 2.1 Hz, Tp-C$^4$**H**) 7.32 (6H, d, $^3J_{H-H}$ = 1.9 Hz, Tp-C$^3$**H**) 7.39 (6H, d, $^3J_{H-H}$ = 2.1 Hz, Tp-C$^5$**H**) ppm; $^{13}$C{$^1$H} NMR ($d_6$-benzene): $\delta$ 4.4 (s, N"-Si(**C**H$_3$)$_3$) 104.9 (s, Tp-**C$^4$**) 135.8 (s, Tp-**C$^5$**) 142.8 (s, Tp-**C$^3$**) ppm; $^{11}$B NMR ($d_6$-benzene): $\delta$ -2.87 (d, $^1J_{B-H}$ = 65.0 Hz, Tp-**B**) ppm; $^{11}$B{$^1$H} NMR ($d_6$-benzene): $\delta$ -2.93 (s, Tp-**B**) ppm; $^{29}$Si{$^1$H} NMR ($d_6$-benzene): $\delta$ -2.38 (s, N"-**Si**(CH$_3$)$_3$) ppm.

Variable Temperature (VT) $^1$H NMR spectra of **2-Y** ($d_8$-toluene) (**Figure S 23**), shows that the terminal and bridging metal hydride resonances cannot be distinguished. Therefore, in solution-state NMR spectra of **2-Ln**, the respective resonance for the metal hydrides is denoted as $\mu$-**H$_3$**. For VT $^1$H NMR of **2-Y,** the JY NMR tube was cooled down in steps of 10 K from 300 K to 220 K and $^1$H NMR spectra recorded after *ca* 5 minutes of temperature equilibration. After the cooling VT NMR experiment, the JY NMR tube was warmed to ambient temperature and solution-stability of **2-Y** was confirmed by $^1$H NMR spectroscopy. Then the temperature was raised to 353 K and maintained for 2 h, obtaining $^1$H NMR spectra every two minutes (see topmost trace in **Figure S 23** for the spectrum at *t* = 2 h). After the VT NMR experiment, the JY NMR tube was cooled down to ambient temperature and the solution-stability of **2-Y** was confirmed by $^1$H NMR spectroscopy.

Anal. Calcd. for $C_{24}H_{41}AlB_2N_{13}Si_2Y$: C, 40.87%; H, 5.86%; N, 25.82%. Found: C, 41.36%; H, 5.61%; N, 24.24%.

IR (FTIR, nujol mull between NaCl discs): 2463 (w, $\upsilon_{BH}$), 1803 (w, $\upsilon_{MH}$), 1730 (w, $\upsilon_{MH}$), 1505 (m, $\upsilon_{C=C}$) $cm^{-1}$.

### A2.2 Synthesis of [Sm(Tp)$_2$($\mu$-H)$_2$Al(H)(N")] 2-Sm

In the glovebox, a 15 mL scintillation vial was charged with a stirrer bar, and then the white powder [Sm(Tp)$_2$(N")] (50.5 mg, 68.5 μmol, 1.0 eq) was dissolved in toluene (2 mL) and stirred. To this solution, a colourless solution of [$Me_3N$•$AlH_3$] (6.1 mg, 68.4 μmol, 1.0 eq) in toluene (1 mL) was added by a pipette dropwise for two minutes with stirring at ambient temperature. This resulted in a colourless solution, which was stirred at ambient temperature for 5 h resulting in the gradual formation of a turbid suspension and slow effervescence of colourless $NMe_3$ gas was observed throughout. Toluene (3 mL) was removed *in vacuo*, within the reaction time of 5 h, yielding white glassy solids. Hexane (6 mL) was added to precipitate powdery white solids, which were washed with hexane (6 mL) and the washing subsequently decanted away to remove unreacted [Sm(Tp)$_2$(N")]. All solvents were removed *in vacuo* and the resultant white powder was ground up and dried *in vacuo* ($10^{-2}$ mbar, 1 h) yielding [Sm(Tp)$_2$($\mu$-H)$_2$Al(H)(N")] **2-Sm** (26.2 mg, 34.2 μmol, 50%).

Compound **2-Sm** has moderate to poor solubilities in toluene and poor solubility in hexane. Microcrystalline **2-Sm** precluded structural characterisation by X-ray diffraction.

$^{1}$H NMR ($d_6$-benzene): $\delta$ 0.24 (3H, very br s, FWHM = 158.4 Hz, $\mu$-**H$_3$**) 0.74 (18H, s, N"-Si(C**H$_3$**)$_3$) 3.96 (6H, s, FWHM = 5.2 Hz, Tp-C$^{3/5}$**H**) 5.56 (6H, t, $^{3}J_{H\text{-}H}$ = 1.8 Hz approximately, Tp-C$^{4}$**H**) 7.25 (2H, very br m, FWHM = 307.9 Hz, Tp-B**H**) 8.42 (6H, d, $^{3}J_{H\text{-}H}$ = 2.0 Hz, Tp-C$^{3/5}$**H**) ppm; $^{13}$C{$^{1}$H} NMR ($d_6$-benzene): $\delta$ 4.9 (s, N"-Si(**C**H$_3$)$_3$) 104.1 (s, Tp-**C$^{4}$**) 136.3 (s, Tp-**C$^{3/5}$**) 141.7 (s, Tp-**C$^{3/5}$**) ppm; $^{11}$B NMR ($d_6$-benzene): $\delta$ 3.24 (d, $^{1}J_{B\text{-}H}$ = 54.3 Hz, Tp-**B**) ppm; $^{11}$B{$^{1}$H} NMR ($d_6$-benzene): $\delta$ 3.17 (s, Tp-**B**) ppm; $^{29}$Si{$^{1}$H} NMR ($d_6$-benzene): $\delta$ -1.54 (s, N"-**Si**(CH$_3$)$_3$) ppm.

Anal. Calcd. for $C_{24}H_{41}AlB_2N_{13}Si_2Sm$: C, 37.59%; H, 5.39%; N, 23.75%. Found: C, 37.22%; H, 5.02%; N, 22.99%.

IR (FTIR, nujol mull between NaCl discs): 2453 (w, $\upsilon_{BH}$), 2443 (w, $\upsilon_{BH}$), 2406 (w, $\upsilon_{BH}$), 1807 (w, $\upsilon_{MH}$), 1740 (w, $\upsilon_{MH}$), 1506 (m, $\upsilon_{C=C}$) $cm^{-1}$.

### A2.3 Synthesis of [Dy(Tp)$_2$($\mu$-H)$_2$Al(H)(N")] 2-Dy

In the glovebox, a 15 mL scintillation vial was charged with a stirrer bar, and then the white powder [Dy(Tp)$_2$(N")] (101.4 mg, 130.2 μmol, 1.0 eq) was dissolved in toluene (1.5 mL) and stirred. To this solution, a colourless solution of [$Me_3N$•$AlH_3$] (11.6 mg, 130.2 μmol, 1.0 eq) in toluene (0.5 mL) was added by a pipette dropwise for two minutes with stirring at ambient temperature. This resulted in a colourless solution and effervescence of colourless $NMe_3$ gas was immediate and the resultant solution was stirred at ambient temperature for 0.5 h and toluene (2 mL) was removed *in vacuo* within the reaction time of 0.5 h, yielding white glassy solids. Hexane (0.5 mL) was added to

precipitate powdery white solids. All solvents were removed *in vacuo* and the resultant white powder was ground up and dried *in vacuo* ($10^{-2}$ mbar, 1.5 h) yielding [Dy(Tp)$_2$($\mu$-H)$_2$Al(H)(N")] **2-Dy** (93.3 mg, 119.8 μmol, 92%).

Compound **2-Dy** has excellent solubility in toluene and moderate solubility in hexane. Block-shaped colourless single crystals of **2-Dy** suitable for X-ray diffraction were grown from a saturated hexane solution at -35 °C overnight.

$^1$H NMR ($d_6$-benzene): $\delta$ -128.53 (6H, very br s, FWHM = 1185.4 Hz, Tp-C**H**) -6.77 (6H, br s, FWHM = 81.1 Hz, Tp-C**H**) 10.25 (2H, 18H, br s, FWHM = 61.8 Hz, N"-Si(C**H$_3$**)$_3$) 40.71 (6H, br s, FWHM = 113.8 Hz, Tp-C**H**) 104.88 (2H, very br s, FWHM = 362.6 Hz, Tp-B**H**) ppm (Note: The $\mu$-**H$_3$** resonance could not be observed owing to the quadrupolar moment of aluminium and the paramagnetic dysprosium); $^{11}$B NMR ($d_6$-benzene): $\delta$ -27.08 (s, Tp-**B**) ppm; $^{11}$B{$^1$H} NMR ($d_6$-benzene): $\delta$ -27.81 (s, Tp-**B**) ppm. Compound **2-Dy** does not exhibit any $^{29}$Si NMR resonances.

Anal. Calcd. for $C_{24}H_{41}AlB_2N_{13}Si_2Dy$: C, 37.01%; H, 5.31%; N, 23.38%. Found: C, 37.52%; H, 5.00%; N, 21.88%.

IR (FTIR, nujol mull between NaCl discs): 2461 (w, $\upsilon_{BH}$), 1812 (w, $\upsilon_{MH}$), 1739 (w, $\upsilon_{MH}$), 1505 (m, $\upsilon_{C=C}$) cm$^{-1}$.

### A2.4 Synthesis of [Yb(Tp)$_2$($\mu$-H)$_2$Al(H)(N")] 2-Yb

In the glovebox, a 15 mL scintillation vial was charged with a stirrer bar, and then the yellow powder [Yb(Tp)$_2$(N")] (127.4 mg, 167.7 μmol, 1.0 eq) was dissolved in toluene (2 mL) and stirred. To this yellow solution, a colourless solution of [Me$_3$N•AlH$_3$] (14.9 mg, 167.2 μmol, 1.0 eq) in toluene (1 mL) was added by a pipette dropwise for two minutes with stirring at ambient temperature. This resulted in a yellow solution and effervescence of colourless NMe$_3$ gas was immediate and the resultant solution stirred at ambient temperature for 0.5 h. Throughout the course of the reaction, the yellow colour of the solution faded to colourless and gradually to a final pale pink colour. Toluene (3 mL) was removed *in vacuo* within the reaction time of 0.5 h, yielding pale pink solids. Hexane (1 mL) was added to precipitate powdery pale pink solids. All solvents were removed *in vacuo* and the resultant pale pink powder was ground up and dried *in vacuo* ($10^{-2}$ mbar, 4.5 h) yielding [Yb(Tp)$_2$($\mu$-H)$_2$Al(H)(N")] **2-Yb** (124.0 mg, 157.1 μmol, 94%).

Compound **2-Yb** has excellent solubility in toluene and moderate solubility in hexane. Block-shaped colourless single crystals of **2-Yb** suitable for X-ray diffraction were grown from a partially saturated toluene solution with a hexane antisolvent at -35 °C over 3 days.

$^1$H NMR ($d_6$-benzene): $\delta$ -92.98 (3H, very br s, FWHM = 579.7 Hz, $\mu$-**H$_3$**) -14.16 (2H, very br m, FWHM = 302.1 Hz, Tp-B**H**) -5.97 (18H, s, N"-Si(C**H$_3$**)$_3$) -1.41 (6H, s, Tp-C**H**) 7.60 (6H, s, Tp-C**H**) 40.38 (6H, very br s, FWHM = 203.8 Hz, Tp-C**H**) ppm; $^{11}$B NMR ($d_6$-benzene): $\delta$ -48.64 (s, Tp-**B**)

ppm; $^{11}B\{^1H\}$ NMR ($d_6$-benzene): $\delta$ -48.27 (s, Tp-**B**) ppm; $^{29}Si\{^1H\}$ NMR ($d_6$-benzene): $\delta$ -14.67 (s, N"-**Si**($CH_3$)$_3$) ppm.

Anal. Calcd. for $C_{24}H_{41}AlB_2N_{13}Si_2Yb$: C, 36.51%; H, 5.23%; N, 23.06%. Found: C, 35.52%; H, 4.68%; N, 21.04%.

IR (FTIR, nujol mull between NaCl discs): 2464 (w, $\upsilon_{BH}$), 1802 (w, $\upsilon_{MH}$), 1729 (w, $\upsilon_{MH}$), 1506 (m, $\upsilon_{C=C}$) cm$^{-1}$.

## A3 Heating [Y(Tp)$_2$($\mu$-H)$_2$Al(H)(N")] 2-Y under dynamic vacuum at 80 °C

A JY NMR tube was charged with the white powder [Y(Tp)$_2$($\mu$-H)$_2$Al(H)(N")] **2-Y** (2.3 mg, 3.3 μmol) and evacuated *in vacuo*. Under application of dynamic vacuum, the JY NMR tube was heated at 80 °C for 2 h. To the resultant white solids, $d_6$-benzene (0.5 mL) was added, and the resultant colourless solution was analysed *via* multinuclear NMR (see **Figure S 53** and **Figure S 54**).

## A4 Reaction between [Yb(Tp)$_2$(N")] 1-Yb with [IDipp•AlH$_3$] and reaction between [Yb(Tp)$_2$($\mu$-H)$_2$Al(H)(N")] 2-Yb with IDipp (1,3-*bis*(2,6-diisopropylphenyl)imidazol-2-ylidene)

### A4.1 Isolation of crystals of [(IDipp)Al(N")(H)$_2$] Al-IDipp in the reaction between [Yb(Tp)$_2$(N")] 1-Yb with [IDipp•AlH$_3$]

In the glovebox, a 20 mL scintillation vial was charged with a stirrer bar, and then the yellow powder [Yb(Tp)$_2$(N")] (31.7 mg, 41.7 μmol, 1.0 eq) was dissolved in toluene (1 mL) and stirred. To this yellow solution, a colourless solution of white crystalline [IDipp•AlH$_3$] (IDipp = 1,3-*bis*(2,6-diisopropylphenyl)imidazol-2-ylidene) (17.5 mg, 41.8 μmol, 1.0 eq) in toluene (1 mL) was added by a pipette dropwise for two minutes with stirring at ambient temperature. This resulted in a beige solution, which was stirred at ambient temperature overnight for 23 h. Toluene (2 mL) was removed *in vacuo*, yielding an orange oil, which was dried *in vacuo* for 0.5 h. Cold (-35 °C) hexane (1 mL) was added to precipitate powdery peach solids. The pale beige supernatant was decanted away and filtered through a pipette containing a Kimwipe. The beige solution was cooled down to -35 °C and the product crystallised overnight as colourless blocks, which were isolated and dried *in vacuo* ($10^{-2}$ mbar, 2 h) yielding [(IDipp)Al(N")(H)$_2$] **Al-IDipp** (7.1 mg, 12.3 μmol, 29%).

Compound **Al-IDipp** has excellent solubility in toluene and hexane. Block-shaped colourless single crystals of **Al-IDipp** suitable for X-ray diffraction were grown from a saturated hexane solution at -35 °C overnight.

$^1H$ NMR ($d_6$-benzene): $\delta$ 0.20 (18H, s, N"-Si(C**H$_3$**)$_3$) 0.96 (12H, d, $^3J_{H-H}$ = 6.7 Hz, CH(C**H$_3$**)$_2$) 1.46 (12H, d, $^3J_{H-H}$ = 6.7 Hz, CH(C**H$_3$**)$_2$) 2.81 (4H, septet, $^3J_{H-H}$ = 6.8 Hz, C**H**(CH$_3$)$_2$) 3.98 (2H, very br s, FWHM = 240.4 Hz, Al**H$_2$**) 6.40 (2H, s, NC**H**) 7.12 (4H, d, $^3J_{H-H}$ = 7.7 Hz, *meta*-Ph-C**H**) 7.23 (2H, t, $^3J_{H-H}$ = 7.7 Hz, *para*-Ph-C**H**) ppm.

### A4.2 NMR-scale reaction between [Yb(Tp)$_2$($\mu$-H)$_2$Al(H)(N")] 2-Yb with IDipp

A JY NMR tube was charged with a dry mixture of the pale pink powder [Yb(Tp)$_2$($\mu$-H)$_2$Al(H)(N")] (3.3 mg, 4.2 μmol, 1.0 eq) and white crystalline IDipp (IDipp = 1,3-*bis*(2,6-diisopropylphenyl)imidazol-2-ylidene) (2.0 mg, 5.1 μmol, 1.2 eq), to which $d_6$-benzene (0.5 mL) was added and the resultant peach solution was analysed *via* multinuclear NMR at ambient temperature up to 24 h. Then the JY NMR tube containing the peach solution was heated at 60 °C for 8 h and analysed *via* multinuclear NMR (see **Figure S 55** to **Figure S 57**).

## A5 NMR-scale reaction between [Me$_3$N•AlH$_3$] with K(N") and NMR-scale reactions between [Ln(Tp)$_2$($\mu$-H)$_2$Al(H)(N")] 2-Ln (Ln = Y, Sm, Dy, Yb) with K(N")

### A5.1 NMR-scale reaction of between [Me$_3$N•AlH$_3$] with K(N")

To a colourless $d_6$-benzene (0.5 mL) solution of the white powder KN" (10.3 mg, 51.6 μmol, 1.0 eq) in a JY NMR tube, the white crystalline [Me$_3$N•AlH$_3$] (4.6 mg, 51.6 μmol, 1.0 eq) was added by a spatula and the resultant white suspension was analysed *via* multinuclear NMR (see **Figure S 58** to **Figure S 60**).

### A5.2 NMR-scale reactions between [Ln(Tp)$_2$($\mu$-H)$_2$Al(H)(N")] 2-Ln (Ln = Y, Sm, Dy, Yb) with K(N")

Ln = Y: A JY NMR tube was charged with a dry mixture of the white powders [Y(Tp)$_2$($\mu$-H)$_2$Al(H)(N")] (6.2 mg, 8.8 μmol, 1.0 eq) and KN" (2.0 mg, 10.0 μmol, 1.1 eq), to which $d_6$-benzene (0.5 mL) was added and the resultant pale white suspension was analysed *via* multinuclear NMR (see **Figure S 61** to **Figure S 63**).

Ln = Sm: A JY NMR tube was charged with a dry mixture of the white powders [Sm(Tp)$_2$($\mu$-H)$_2$Al(H)(N")] (2.0 mg, 2.6 μmol, 1.0 eq) and KN" (1.0 mg, 5.0 μmol, 1.9 eq), to which $d_6$-benzene (0.5 mL) was added and the resultant white suspension was analysed *via* multinuclear NMR (see **Figure S 64** and **Figure S 65**). Note: No $^{29}$Si resonances were observed.

Ln = Dy: To a colourless $d_6$-benzene (0.5 mL) solution of [Dy(Tp)$_2$($\mu$-H)$_2$Al(H)(N")] (11.6 mg, 14.9 μmol, 1.0 eq) in a JY NMR tube, the white powder KN" (3.0 mg, 15.0 μmol, 1.0 eq) was added by a spatula and the resultant colourless solution was analysed *via* multinuclear NMR (see **Figure S 66**). Note: No real $^{11}$B or $^{29}$Si resonances were observed.

Ln = Yb: To a pale pink $d_6$-benzene (0.5 mL) solution of [Yb(Tp)$_2$($\mu$-H)$_2$Al(H)(N")] (7.3 mg, 9.2 μmol, 1.0 eq) in a JY NMR tube, the white powder KN" (2.0 mg, 10.0 μmol, 1.1 eq) was added by a spatula and the resultant peach orange solution was analysed *via* multinuclear NMR (see **Figure S 67** to **Figure S 69** for data points t = 0.25 h and t = 20 h for comparison). With gradual course of the

reaction, the colour of the reaction mixture darkened, resulting a final dark red colour at t = 20 h, arising from a high concentration of the Yb(II) complex $[Yb(Tp)_2]$[5] formed in the reaction.

# A6 Synthesis of $[Ln(Tp)_2\{\kappa^2\text{-(Cy)NCHN(Cy)}\}]$ 3-Ln (Ln = Dy, Yb)

## A6.1 Synthesis of $[Dy(Tp)_2\{\kappa^2\text{-(Cy)NCHN(Cy)}\}]$ 3-Dy

In the glovebox, a 15 mL scintillation vial was charged with a stirrer bar, and then the white powder $[Dy(Tp)_2(\mu\text{-H})_2Al(H)(N'')]$ (29.0 mg, 37.2 μmol, 1.0 eq) was dissolved in toluene (1 mL) and stirred. To this colourless solution, a colourless solution of CyN=C=NCy (Cy = $C_6H_{11}$) (23.0 mg, 111.5 μmol, 3.0 eq) in toluene (0.5 mL) was added by a pipette dropwise for two minutes with stirring at ambient temperature. The resultant colourless solution was stirred at ambient temperature for 1 h. Toluene (1.5 mL) was removed *in vacuo* within the reaction time of 1 h, yielding pastel white solids. Hexane (5 mL) was added to precipitate powdery white solids, which were washed with hexane (5 mL) and the washing subsequently decanted away to remove $[Al\{\kappa^2\text{-(Cy)NCHN(Cy)}\}_2(N'')]$. All solvents were removed *in vacuo* and the resultant white powder was ground up and dried *in vacuo* ($10^{-2}$ mbar, 0.5 h) yielding $[Dy(Tp)_2\{\kappa^2\text{-(Cy)NCHN(Cy)}\}]$ **3-Dy** (15.0 mg, 18.8 μmol, 51%).

Compound **3-Dy** has excellent solubility in toluene and moderate solubility in hexane. Block-shaped yellow single crystals of **3-Dy** suitable for X-ray diffraction were grown from a saturated toluene solution with a hexane antisolvent at -35 °C over 1 week.

$^1$H NMR ($d_6$-benzene): $\delta$ -35.51 (6H, very br s, FWHM = 410.8 Hz, Tp-C**H**) 13.02 (4H, br s, FWHM = 70.8 Hz, Cy-**H**) 16.42 (7H, very br s, FWHM = 779.0 Hz, Cy-**H**) 17.70 (6H, br s, FWHM = 37.4 Hz, Tp-C**H**) 43.51 (11H, br s, FWHM = 277.7 Hz, Cy-C**H**) 176.22 (6H, very br s, FWHM = 673.3 Hz, Tp-C**H**) ppm (Note: The Tp-B**H** and (Cy)NC**H**N(Cy) resonances could not be observed); $^{11}$B NMR ($d_6$-benzene): $\delta$ -123.73 (s, Tp-**B**) ppm; $^{11}$B{$^1$H} NMR ($d_6$-benzene): $\delta$ -123.61 (s, Tp-**B**) ppm.

Anal. Calcd. for $C_{31}H_{43}B_2N_{14}Dy$: C, 46.78%; H, 5.45%; N, 24.64%. Found: C, 47.94%; H, 5.60%; N, 22.98%. Discrepancies in the experimentally observed CHN values compared to calculated values may be the result of minor $[Al\{\kappa^2\text{-(Cy)NCHN(Cy)}\}_2(N'')]$ impurity in the sample. However, the observed CH ratio $C_{31}H_{43}$ matches the calculated.

IR (FTIR, nujol mull between NaCl discs): 2458 (m, $\upsilon_{BH}$), 2413 (w, $\upsilon_{BH}$), 2373 (w, $\upsilon_{BH}$), 1556 (s, $\upsilon_{NCN}$), 1504 (m, $\upsilon_{C=C}$) cm$^{-1}$.

## A6.2 Synthesis of $[Yb(Tp)_2\{\kappa^2\text{-(Cy)NCHN(Cy)}\}]$ 3-Yb

In the glovebox, a 15 mL scintillation vial was charged with a stirrer bar, and then the pale pink powder $[Yb(Tp)_2(\mu\text{-H})_2Al(H)(N'')]$ (31.0 mg, 39.3 μmol, 1.0 eq) was dissolved in toluene (1 mL) and stirred. To this pale pink solution, a colourless solution of CyN=C=NCy (Cy = $C_6H_{11}$) (24.5 mg, 118.7 μmol, 3.0 eq) in toluene (0.5 mL) was added by a pipette dropwise for two minutes with stirring at ambient temperature. This resulted in a yellow solution, which was stirred at ambient temperature

for 1 h. Toluene (1.5 mL) was removed *in vacuo* within the reaction time of 1 h, yielding yellow solids. Hexane (4 mL) was added to precipitate powdery yellow solids, which were washed with hexane (4 mL) and the washing subsequently decanted away to remove [Al{$\kappa^2$-(Cy)NCHN(Cy)}$_2$(N")]. All solvents were removed *in vacuo* and the resultant yellow powder was ground up and dried *in vacuo* ($10^{-2}$ mbar, 0.5 h) yielding [Yb(Tp)$_2${$\kappa^2$-(Cy)NCHN(Cy)}] **3-Yb** (15.4 mg, 19.1 $\mu$mol, 49%).

Compound **3-Yb** has excellent solubility in toluene and moderate solubility in hexane. Block-shaped yellow single crystals of **3-Yb** suitable for X-ray diffraction were grown from a saturated toluene solution with a hexane antisolvent at -35 °C over 3 days.

$^1$H NMR (*d$_6$*-benzene): $\delta$ -74.71 (2H, br s, FWHM = 44.0 Hz, Cy-**H**) -44.81 (4H, br s, FWHM = 48.2 Hz, Cy-**H**) -23.98 (6H, very br s, FWHM = 954.6 Hz, Tp-C**H**) -11.65 (8H, br s, FWHM = 35.5 Hz, Cy-**H**) -10.43 (4H, approx. q, $^3J_{H\text{-}H}$ = 10.8 Hz, Cy-**H**) -7.21 (6H, very br s, FWHM = 216.9 Hz, Tp-C**H**) -6.35 (4H, d, $^3J_{H\text{-}H}$ = 9.1 Hz, Cy-**H**) 0.79 (2H, very br m, FWHM = 298.6 Hz, Tp-B**H**) 70.85 (6H, very br s, FWHM = 4004.0 Hz, Tp-C**H**) ppm (Note: The (Cy)NC**H**N(Cy) resonance could not be observed); $^{11}$B NMR (*d$_6$*-benzene): $\delta$ -11.38 (s, Tp-**B**) ppm; $^{11}$B{$^1$H} NMR (*d$_6$*-benzene): $\delta$ -11.22 (s, Tp-**B**) ppm.

Anal. Calcd. for $C_{31}H_{43}B_2N_{14}Yb$: C, 46.17%; H, 5.37%; N, 24.32%. Found: C, 48.90%; H, 5.54%; N, 21.89%. Discrepancies in the experimentally observed CHN values compared to calculated values may be the result of minor [Al{$\kappa^2$-(Cy)NCHN(Cy)}$_2$(N")] impurity in the sample. However, the observed CH ratio $C_{31}H_{43}$ closely matches the calculated.

IR (FTIR, nujol mull between NaCl discs): 2458 (m, $\upsilon_{BH}$), 2413 (w, $\upsilon_{BH}$), 2374 (w, $\upsilon_{BH}$), 1559 (s, $\upsilon_{NCN}$), 1504 (m, $\upsilon_{C=C}$) cm$^{-1}$.

# A7 NMR-scale reactions between [Ln(Tp)$_2$($\mu$-H)$_2$Al(H)(N")] 2-Ln (Ln = Y, Sm) with three equivalents of CyN=C=NCy (Cy = $C_6H_{11}$, DCC)

## A7.1 NMR-scale reaction between [Y(Tp)$_2$($\mu$-H)$_2$Al(H)(N")] 2-Y with three equivalents of CyN=C=NCy (DCC, Cy = $C_6H_{11}$)

To a colourless *d$_6$*-benzene (0.5 mL) solution of [Y(Tp)$_2$($\mu$-H)$_2$Al(H)(N")] (9.8 mg, 13.9 $\mu$mol, 1.0 eq) in a JY NMR tube, the white crystalline CyN=C=NCy (Cy = $C_6H_{11}$) (8.5 mg, 41.2 $\mu$mol, 3.0 eq) was added by a spatula and the resultant colourless solution was analysed *via* multinuclear NMR (see **Figure S 70** to **Figure S 72**). Solvent was subsequently removed *in vacuo* from the JY NMR tube and the resultant white solids were washed with hexane (1 mL) and the washing decanted away to remove [Al{$\kappa^2$-(Cy)NCHN(Cy)}$_2$(N")]. All solvents were removed *in vacuo* and the resultant white powder was ground up and dried *in vacuo* ($10^{-2}$ mbar, 0.5 h) yielding [Y(Tp)$_2${$\kappa^2$-(Cy)NCHN(Cy)}] **3-Y** (4.5 mg, 6.2 $\mu$mol, 45%) (see **Figure S 70(b)**).

Compound **3-Y** has excellent solubility in toluene and moderate solubility in hexane.

$^1H$ NMR ($d_6$-benzene): $\delta$ 0.63-1.68 (20H, m, Cy-**H**) 2.76 (2H, tt, $^3J_{Hax-Hax}$ = 11.3 Hz, $^3J_{Hax-Heq}$ = 3.2 Hz, Cy-**H**) 4.83 (2H, very br m, FWHM = 282.9 Hz approximately, Tp-B**H**) 5.84 (6H, t, $^3J_{H-H}$ = 1.9 Hz, Tp-C$^4$**H**) 7.26 (6H, br s, FWHM = 28.1 Hz, Tp-C$^3$**H**) 7.54 (6H, dd, $^3J_{H-H}$ = 2.1 Hz, $^4J_{H-H}$ = 0.4 Hz, Tp-C$^5$**H**) 8.28 (1H, d, $^4J_{H-H}$ = 2.8 Hz ppm, (Cy)NC**H**N(Cy)) ppm; $^{11}B$ NMR ($d_6$-benzene): $\delta$ -2.80 (s, Tp-**B**) ppm.

### A7.2 NMR-scale reaction between [Sm(Tp)$_2$($\mu$-H)$_2$Al(H)(N")] 2-Sm with three equivalents of CyN=C=NCy (DCC, Cy = $C_6H_{11}$)

To a turbid $d_6$-benzene (0.5 mL) suspension of [Sm(Tp)$_2$($\mu$-H)$_2$Al(H)(N")] (25.0 mg, 32.6 μmol, 1.0 eq) in a JY NMR tube, the white crystalline CyN=C=NCy (Cy = $C_6H_{11}$) (20.2 mg, 97.9 μmol, 3.0 eq) was added by a spatula and the resultant turbid suspension was analysed *via* multinuclear NMR (see **Figure S 73** to **Figure S 75**). Solvent was subsequently removed *in vacuo* from the JY NMR tube and the resultant white solids were washed with hexane (5 mL) and the washing decanted away to remove [Al{$\kappa^2$-(Cy)NCHN(Cy)}$_2$(N")]. All solvents were removed *in vacuo* and the resultant white powder was ground up and dried *in vacuo* ($10^{-2}$ mbar, 1.5 h) yielding [Sm(Tp)$_2${$\kappa^2$-(Cy)NCHN(Cy)}] **3-Sm** (9.1 mg, 11.6 μmol, 36%) (see **Figure S 73(b)**).

Compound **3-Sm** has poor solubilities in toluene and hexane.

$^1H$ NMR ($d_6$-benzene): $\delta$ -0.50 to 4.00 (22H, m, Cy-**H**) 4.62 (br s, FWHM = 44.3 Hz, Tp-C**H**) 5.78 (6H, s, Tp-C**H**) 7.16 (2H, very br m, overlapped with the $d_6$-benzene resonance, Tp-B**H**) 8.39 (6H, s, Tp-C**H**) 15.26 (1H, s, (Cy)NC**H**N(Cy)) ppm; $^{11}B$ NMR ($d_6$-benzene): $\delta$ 2.19 (s, Tp-**B**) ppm.

## A8 NMR-scale reactions between [Ln(Tp)$_2$($\mu$-H)$_2$Al(H)(N")] 2-Ln (Ln = Y, Sm, Dy, Yb) with excess $^i$PrN=C=N$^i$Pr (DIC)

To four separate JY NMR tubes containing $d_6$-benzene (0.5 mL) solutions/suspension of [Ln(Tp)$_2$($\mu$-H)$_2$Al(H)(N")] (colourless solution for Y: 5.4 mg, 7.7 μmol; turbid suspension for Sm: 1.5 mg, 2.0 μmol; colourless solution for Dy: 4.8 mg, 6.2 μmol; pale pink solution for Yb: 4.8 mg, 6.1 μmol), the colourless liquid $^i$PrN=C=N$^i$Pr ($^i$Pr = CH(CH$_3$)$_2$) (10.0 μL, 8.1 mg, 63.9 μmol, *ca* 8.3-32.0 eq considering all four cases above) was added by a micropipette to each of the four JY NMR tubes and the resultant colourless solutions (Ln = Y, Dy), turbid suspension (Ln = Sm) and yellow solution (Ln = Yb) were analysed *via* multinuclear NMR (for Y see **Figure S 76** to **Figure S 78**, for Sm see **Figure S 79** to **Figure S 81**, for Dy see **Figure S 82** to **Figure S 84**, for Yb see **Figure S 85** to **Figure S 87**).

# A9 NMR-scale reactions between [Ln(Tp)$_2$($\mu$-H)$_2$Al(H)(N")] 2-Ln (Ln = Y, Sm, Dy, Yb) with benzophenone (Ph$_2$C=O)

## A9.1 NMR-scale reactions between [Ln(Tp)$_2$($\mu$-H)$_2$Al(H)(N")] 2-Ln (Ln = Y, Yb) with sequential addition of one, two, and three equivalents of benzophenone

Ln = Y: A JY NMR tube was charged with a dry mixture of the white powder [Y(Tp)$_2$($\mu$-H)$_2$Al(H)(N")] (6.6 mg, 9.4 μmol, 1.0 eq) and colourless crystalline benzophenone (2.0 mg, 11.0 μmol, 1.2 eq), to which $d_6$-benzene (0.5 mL) was added and the resultant colourless solution was analysed *via* multinuclear NMR. Subsequently the second equivalent of benzophenone (2.0 mg, 11.0 μmol, 2.4 eq overall) was added by a spatula and the resultant colourless solution was analysed *via* multinuclear NMR. This was followed by the third equivalent of benzophenone (2.0 mg, 11.0 μmol, 3.6 eq overall) and the resultant pale yellow solution was analysed *via* multinuclear NMR (see **Figure S 88** to **Figure S 90** for all the data points arising from the addition of first, second, and third equivalents, respectively).

Ln = Yb: A JY NMR tube was charged with a dry mixture of the pale pink powder [Yb(Tp)$_2$($\mu$-H)$_2$Al(H)(N")] (7.0 mg, 8.9 μmol, 1.0 eq) and colourless crystalline benzophenone (2.0 mg, 11.0 μmol, 1.2 eq), to which $d_6$-benzene (0.5 mL) was added and the resultant colourless solution was analysed *via* multinuclear NMR. Subsequently the second equivalent of benzophenone (2.0 mg, 11.0 μmol, 2.4 eq overall) was added by a spatula and the resultant colourless solution was analysed *via* multinuclear NMR. This was followed by the third equivalent of benzophenone (1.5 mg, 8.2 μmol, 3.4 eq overall) and the resultant pale yellow solution was analysed *via* multinuclear NMR (see **Figure S 91** to **Figure S 93** for all the data points arising from the addition of first, second, and third equivalents, respectively).

## A9.2 NMR-scale reactions between [Ln(Tp)$_2$($\mu$-H)$_2$Al(H)(N")] 2-Ln (Ln = Sm, Dy) with direct addition of three equivalents of benzophenone

Ln = Sm: To a turbid $d_6$-benzene (0.5 mL) suspension of [Sm(Tp)$_2$($\mu$-H)$_2$Al(H)(N")] (2.3 mg, 3.0 μmol, 1.0 eq) in a JY NMR tube, the colourless crystalline benzophenone (2.0 mg, 11.0 μmol, 3.7 eq) was added by a spatula and the resultant pale yellow suspension was analysed *via* multinuclear NMR (see **Figure S 94** to **Figure S 96**). When the white solids settled, a yellow solution remained on top.

Ln = Dy: To a colourless $d_6$-benzene (0.5 mL) solution of [Dy(Tp)$_2$($\mu$-H)$_2$Al(H)(N")] (4.0 mg, 5.1 μmol, 1.0 eq) in a JY NMR tube, the colourless crystalline benzophenone (3.1 mg, 17.0 μmol, 3.3 eq) was added by a spatula and the resultant pale yellow solution was analysed *via* multinuclear NMR (see **Figure S 97** and **Figure S 98**). Note: No $^{11}$B resonances were observed.

## A10 Isolation of single-crystals of [{Al(N")(H)($\mu$-O(CHPh$_2$))}$_2$] 4-Al from reactions between [Ln(Tp)$_2$($\mu$-H)$_2$Al(H)(N")] 2-Ln (Ln = Y, Sm, Dy, Yb) with benzophenone (Ph$_2$C=O)

On multiple occasions, when working up the reactions of [Ln(Tp)$_2$($\mu$-H)$_2$Al(H)(N")] **2-Ln** with benzophenone in $d_6$-benzene, by extraction of the crude reaction mixture into hexane with filtration and subsequent cooling to -24 °C, the block-shaped colourless single crystals of [{Al(N")(H)($\mu$-O(CHPh$_2$))}$_2$] **4-Al** (**Figure S 116**) were isolated.

# B. Spectroscopic Data for Complexes

## B1 Nuclear magnetic resonance (NMR) data for isolated compounds

### B1.1 $Sm(OTf)_3$

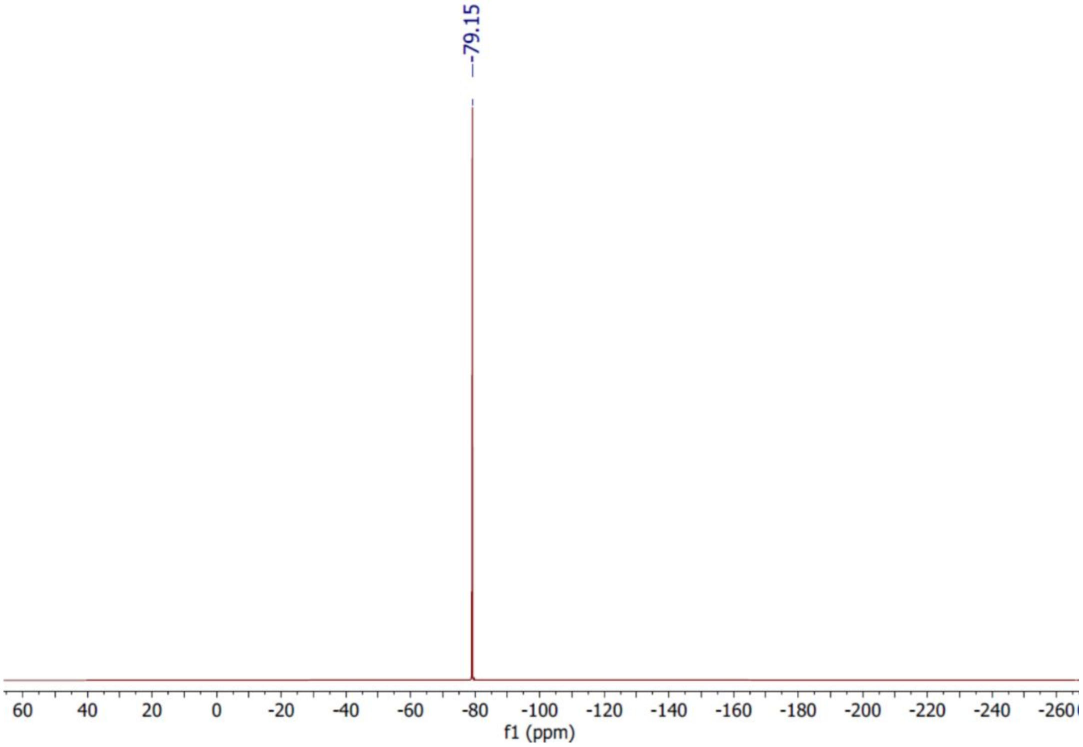


**Figure S 1.** $^{19}F$ NMR spectrum of $Sm(OTf)_3$, recorded in $d_3$-MeCN.

### B1.2 $[Sm(Tp)_2(OTf)]$ Sm-OTf

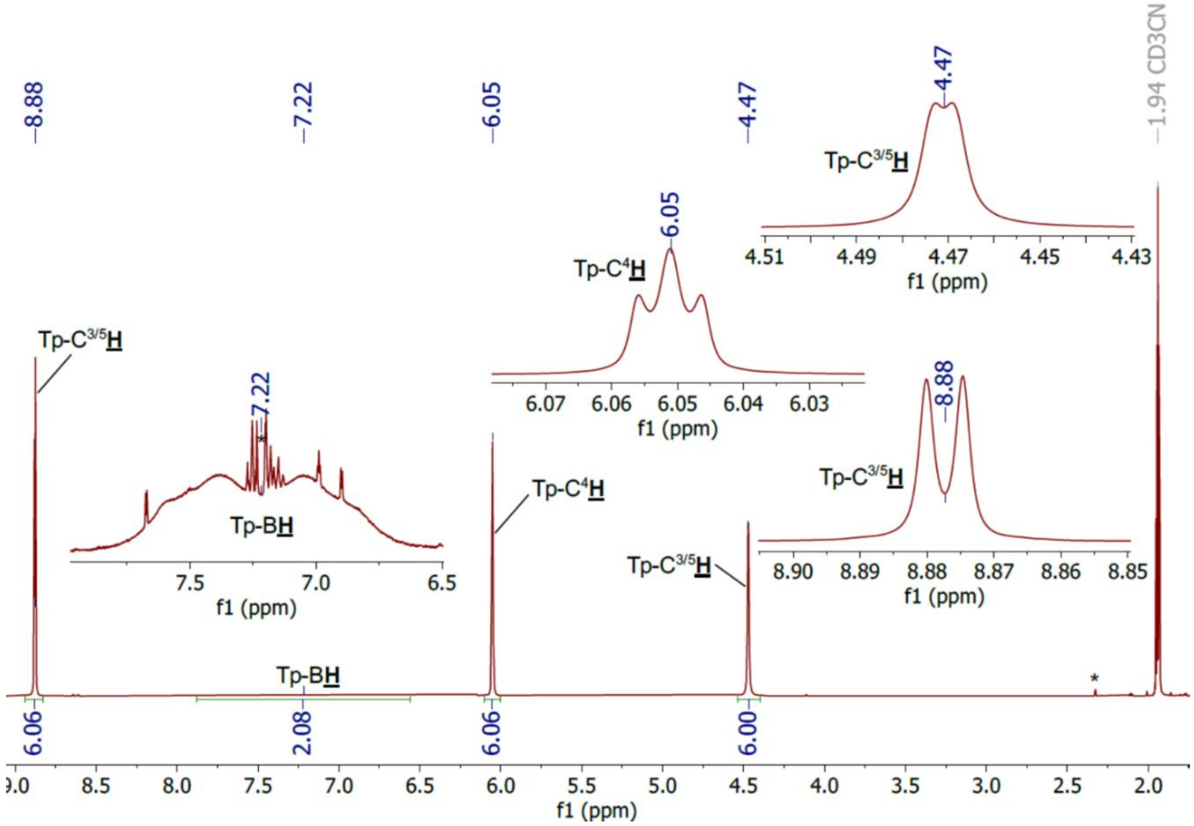


**Figure S 2.** $^{1}H$ NMR spectrum of $[Sm(Tp)_2(OTf)]$ **Sm-OTf**, recorded in $d_3$-MeCN. Residual toluene is denoted with *.

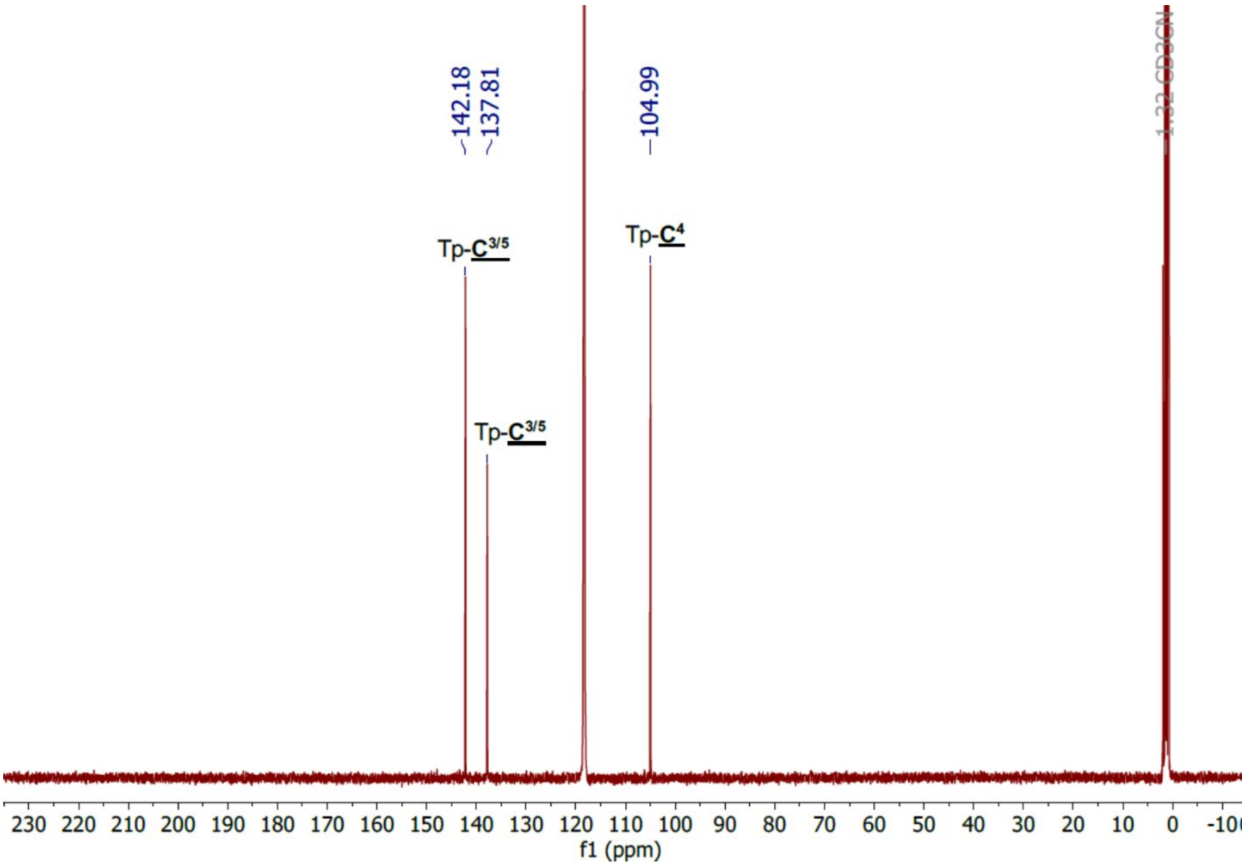


**Figure S 3.** $^{13}C\{^1H\}$ NMR spectrum of $[Sm(Tp)_2(OTf)]$ **Sm-OTf**, recorded in $d_3$-MeCN.

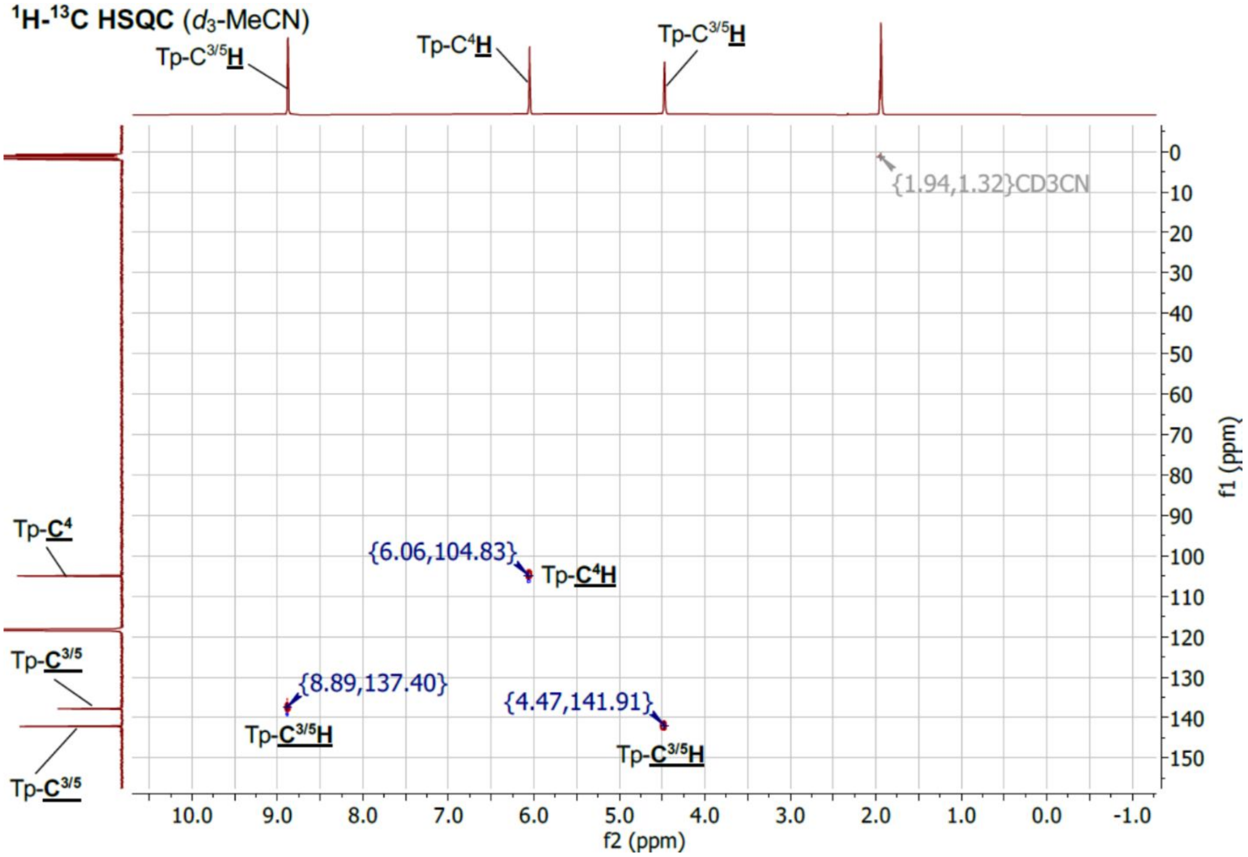


**Figure S 4.** $^1H$-$^{13}C$ HSQC NMR spectrum of $[Sm(Tp)_2(OTf)]$ **Sm-OTf**, recorded in $d_3$-MeCN.

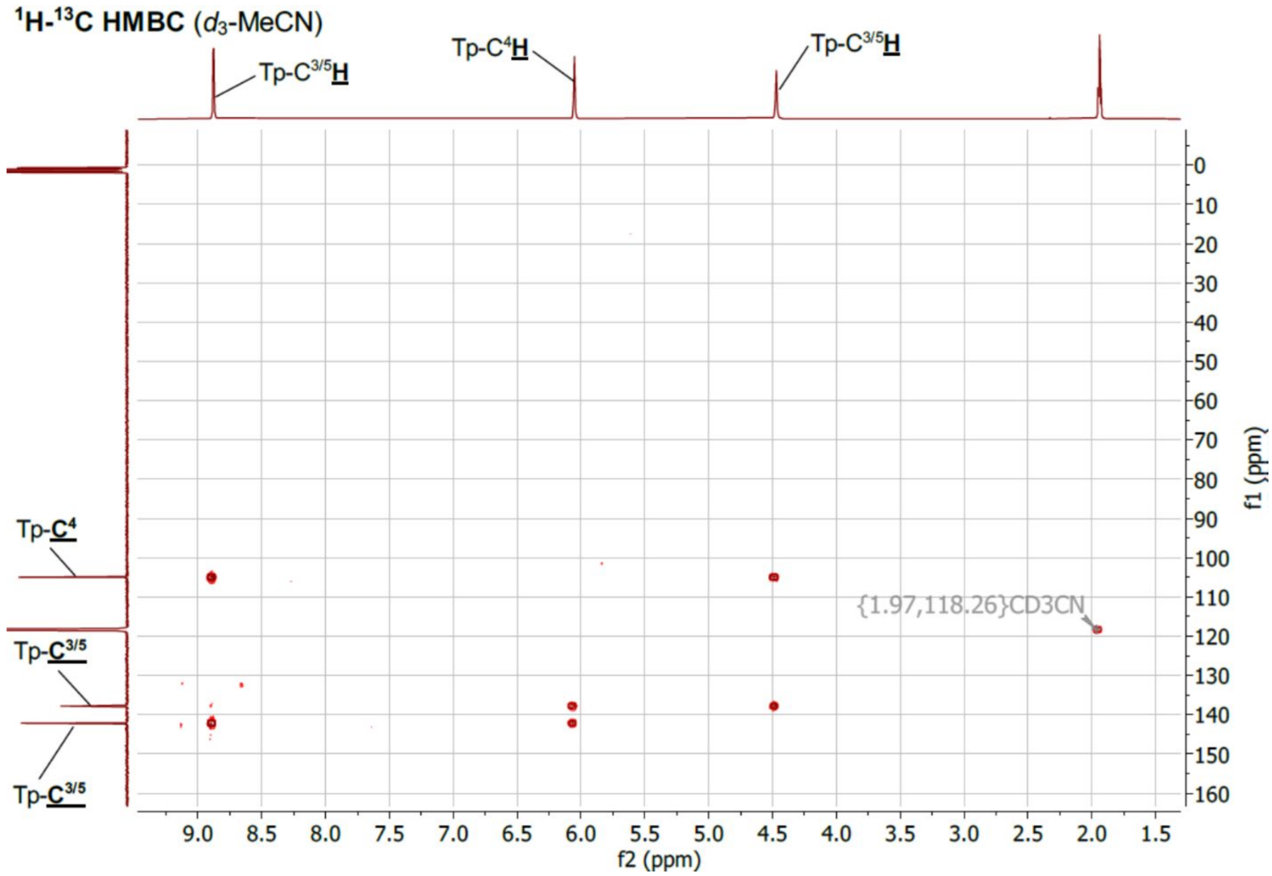


**Figure S 5.** $^{1}$H-$^{13}$C HMBC NMR spectrum of [Sm(Tp)$_2$(OTf)] **Sm-OTf**, recorded in $d_3$-MeCN.

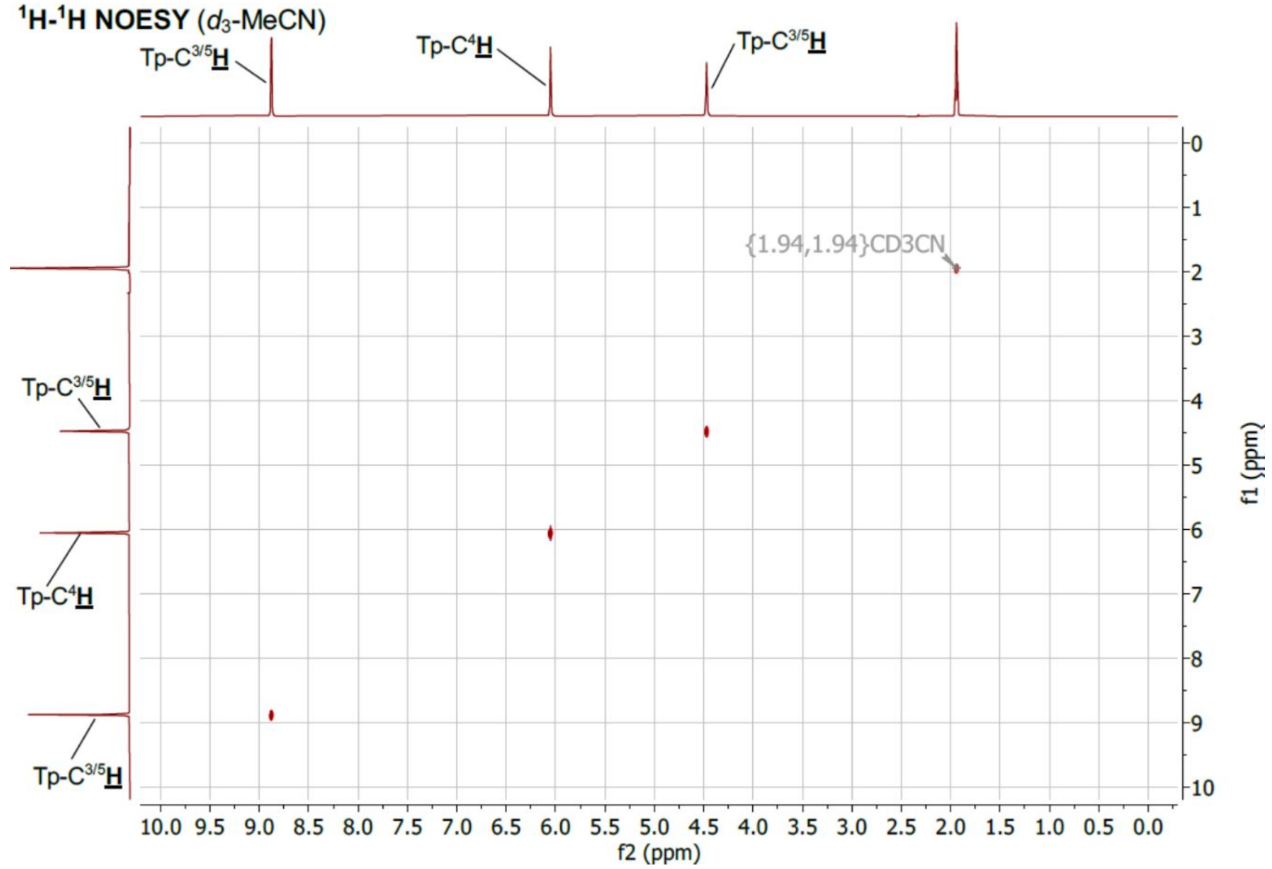


**Figure S 6.** $^{1}$H-$^{1}$H NOESY NMR spectrum of [Sm(Tp)$_2$(OTf)] **Sm-OTf**, recorded in $d_3$-MeCN.

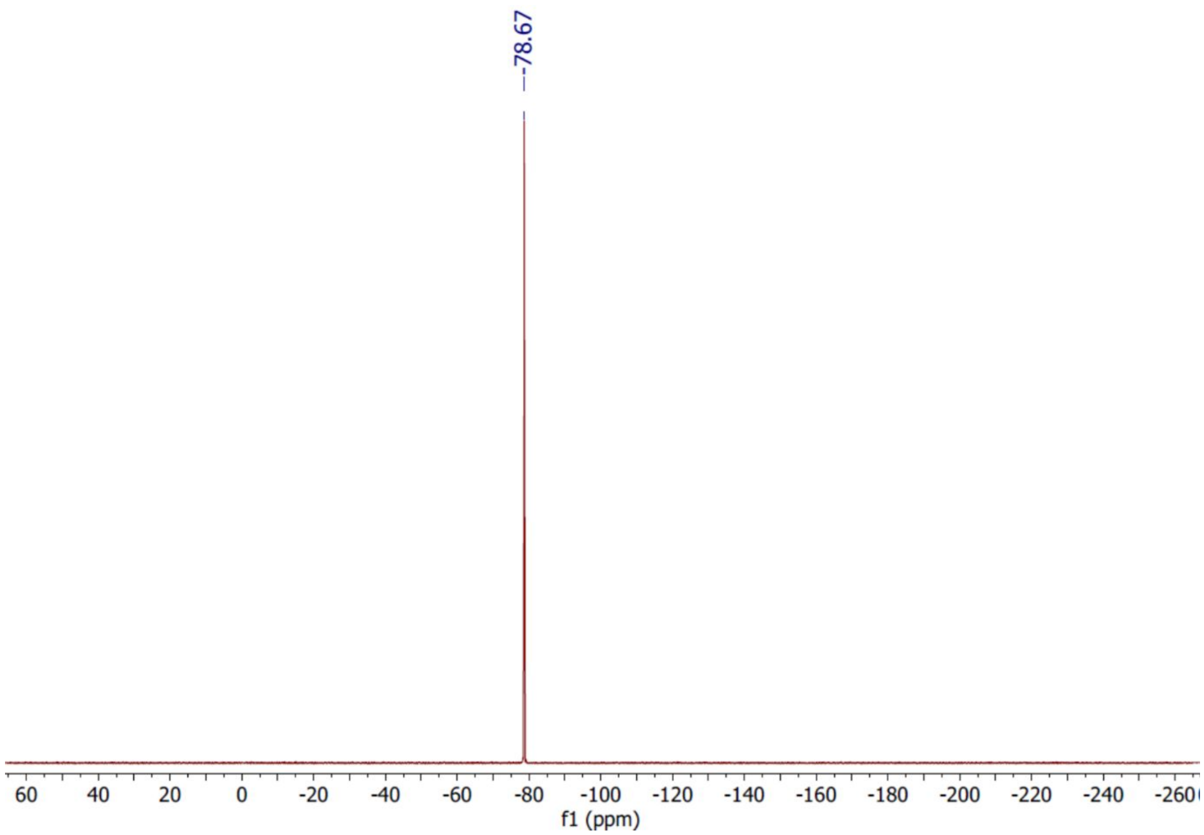


**Figure S 7.** $^{19}F$ NMR spectrum of $[Sm(Tp)_2(OTf)]$ **Sm-OTf**, recorded in $d_3$-MeCN.

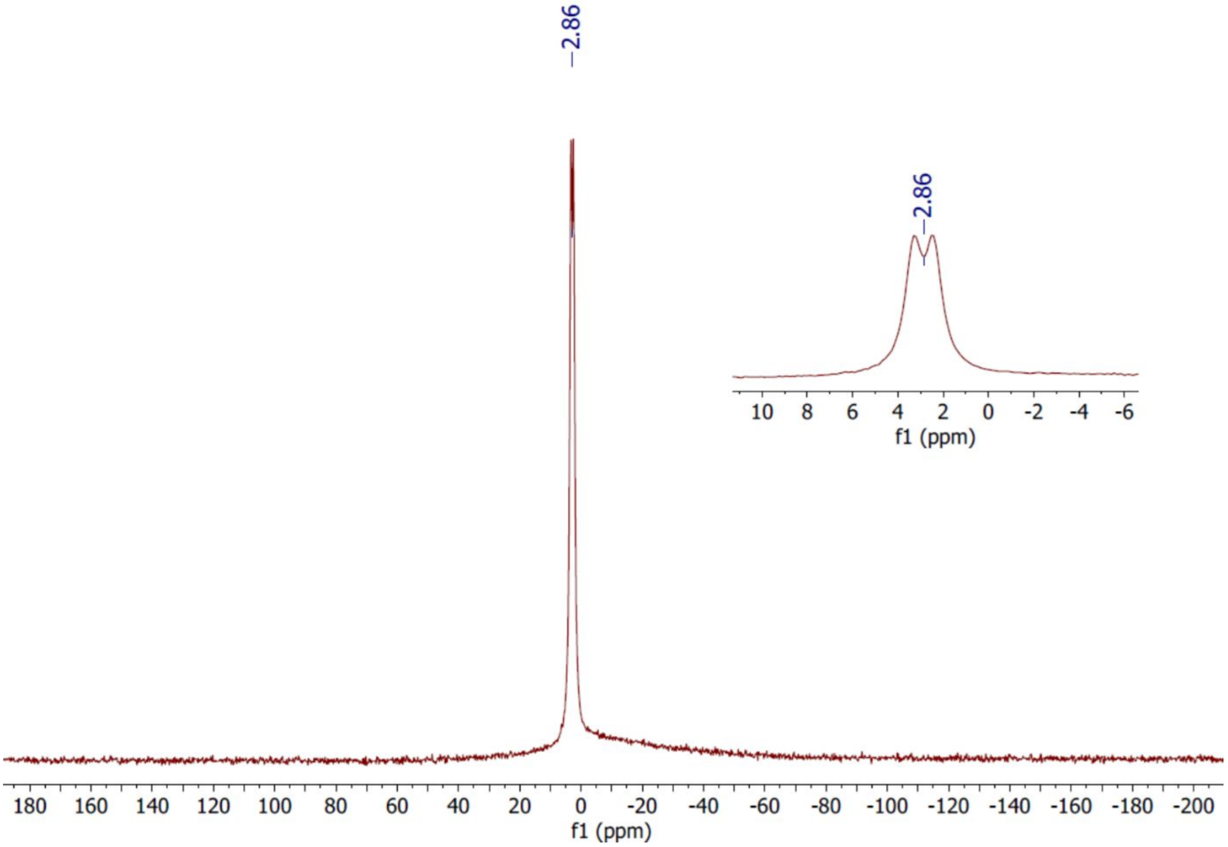


**Figure S 8.** $^{11}B$ NMR spectrum of $[Sm(Tp)_2(OTf)]$ **Sm-OTf**, recorded in $d_3$-MeCN.

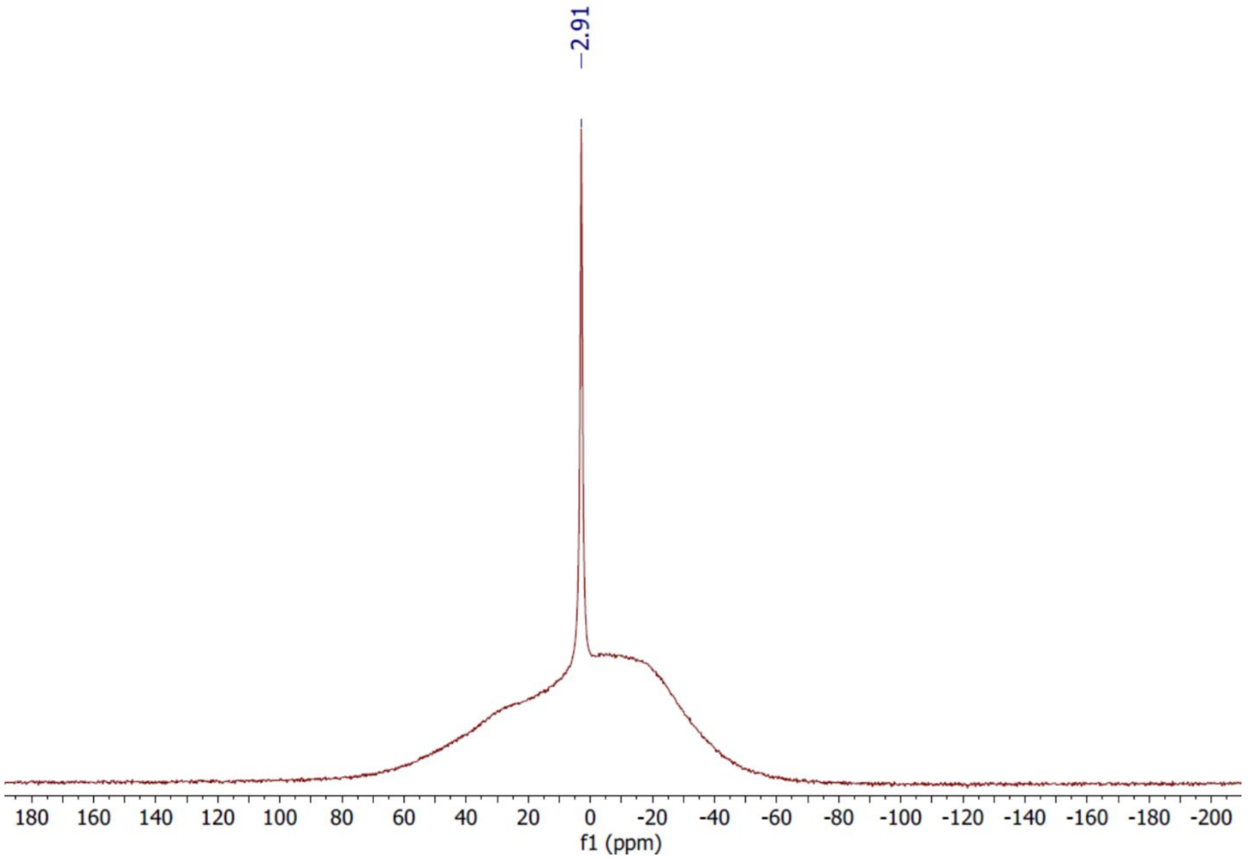


**Figure S 9.** $^{11}B\{^{1}H\}$ NMR spectrum of $[Sm(Tp)_2(OTf)]$ **Sm-OTf**, recorded in $d_3$-MeCN.

### B1.3 $[Sm(Tp)_2(N'')]$ 1-Sm

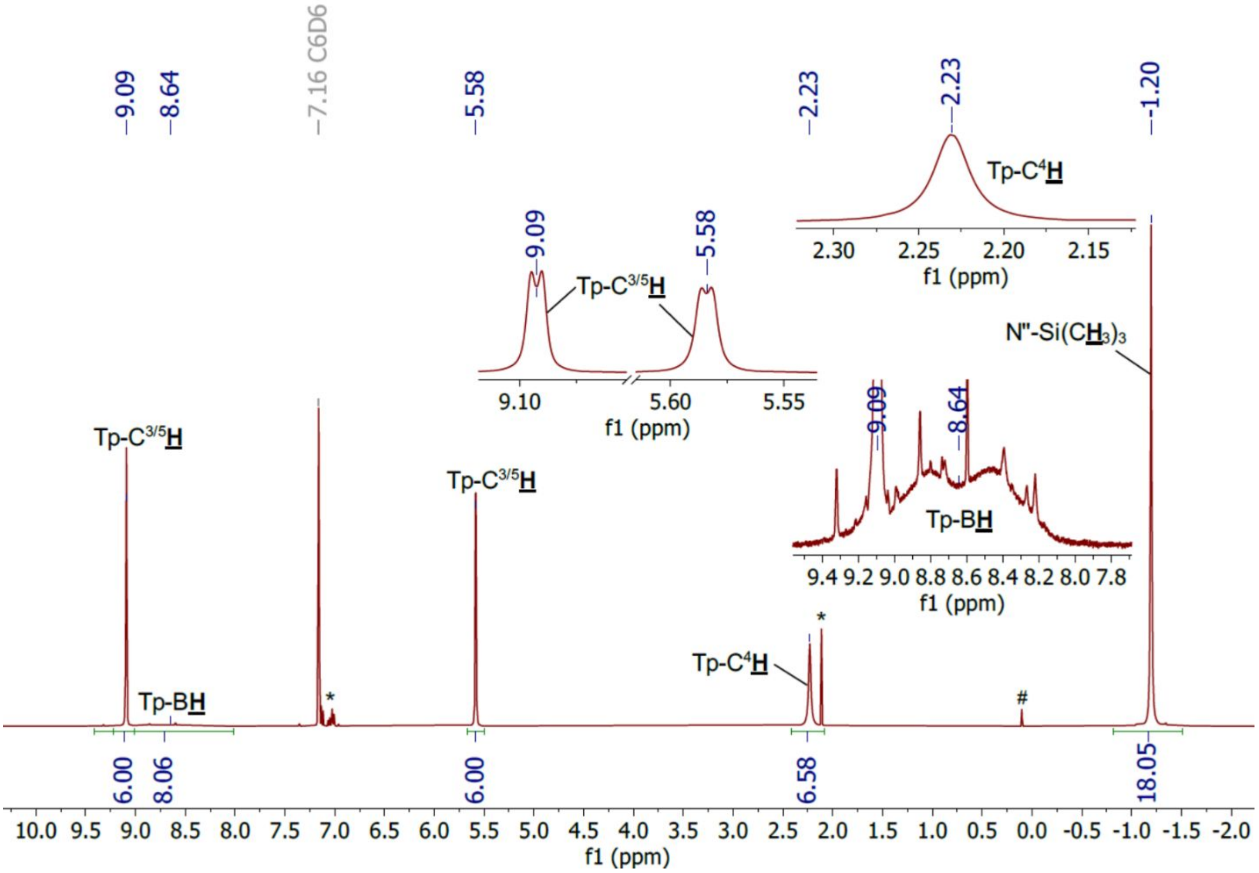


**Figure S 10.** $^{1}H$ NMR spectrum of $[Sm(Tp)_2(N'')]$ **1-Sm**, recorded in $d_6$-benzene. Residual toluene is denoted with * and minor HN'' impurity is denoted with #.

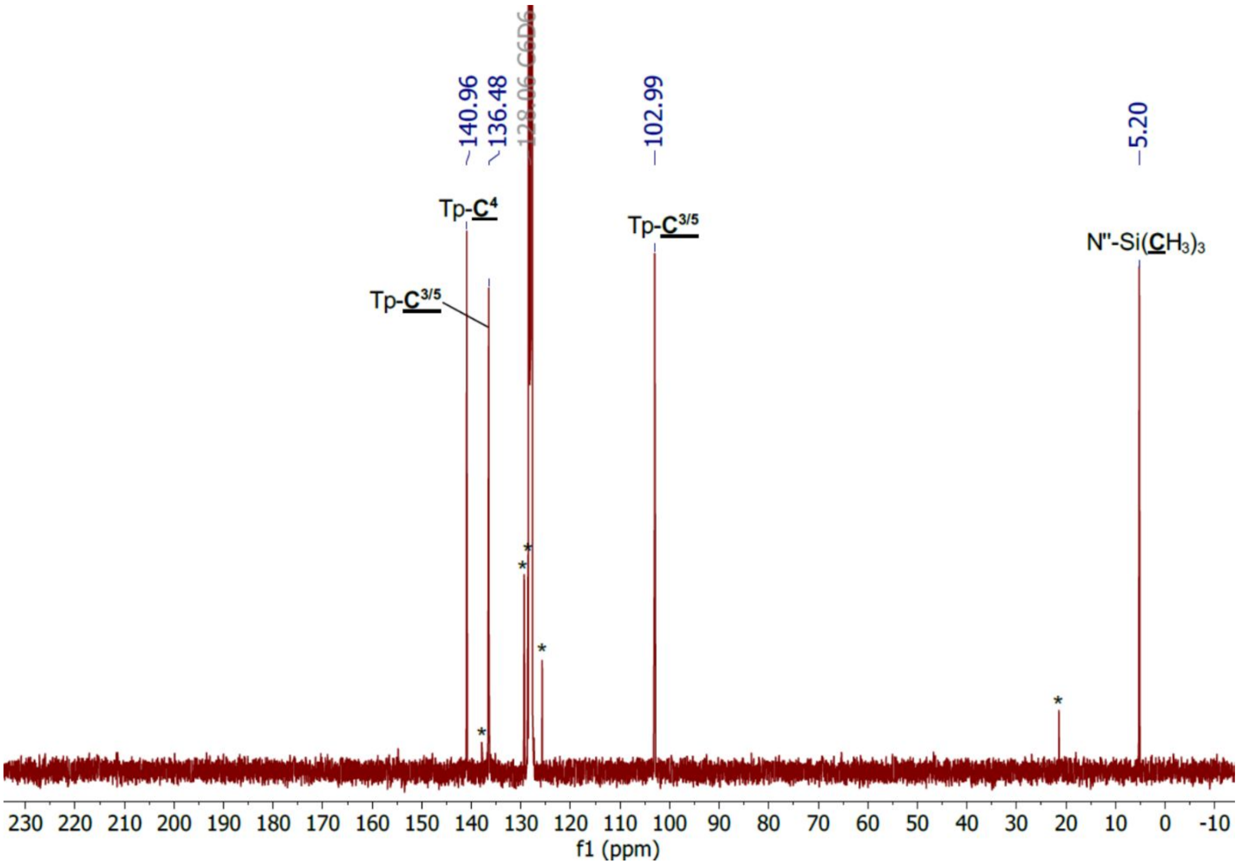


**Figure S 11.** $^{13}C\{^{1}H\}$ NMR spectrum of $[Sm(Tp)_2(N'')]$ **1-Sm**, recorded in $d_6$-benzene. Residual toluene is denoted with *.

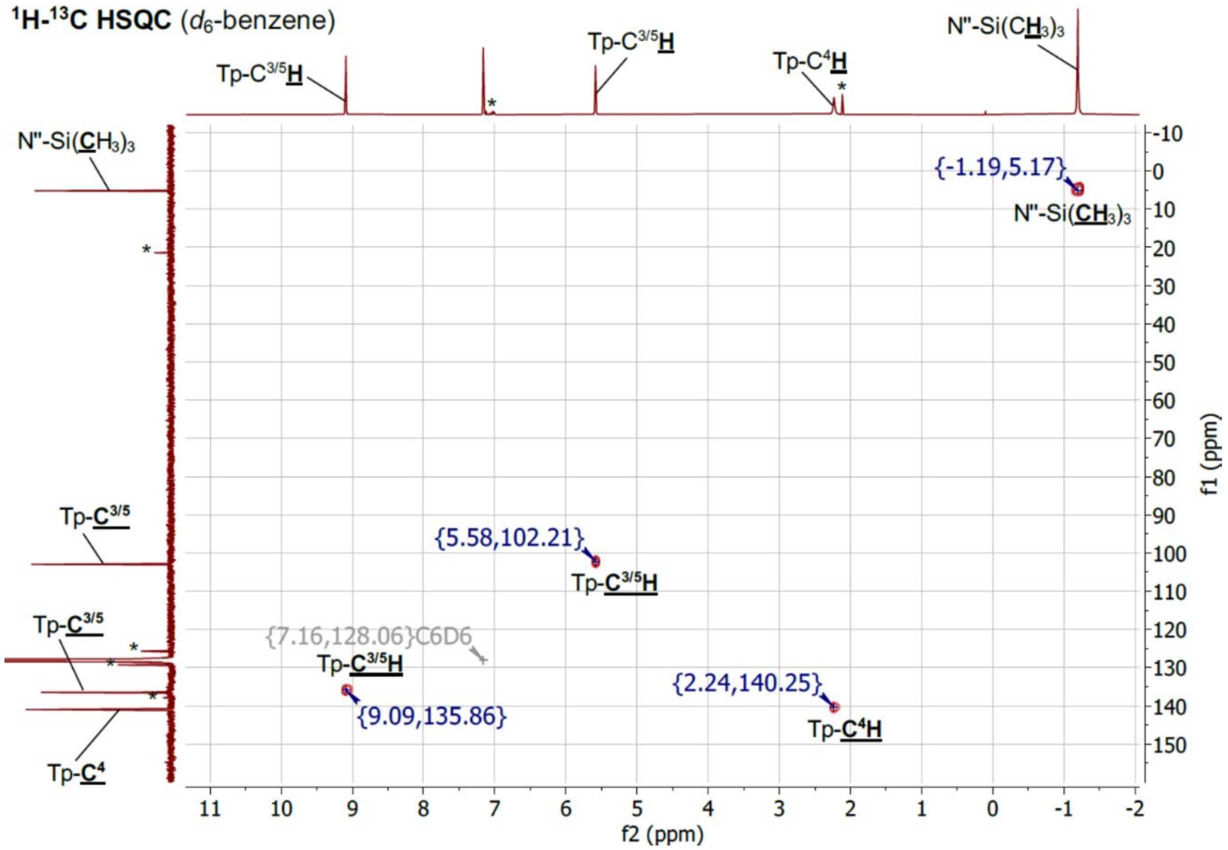


**Figure S 12.** $^{1}H$-$^{13}C$ HSQC spectrum of $[Sm(Tp)_2(N'')]$ **1-Sm**, recorded in $d_6$-benzene. Residual toluene is denoted with *.

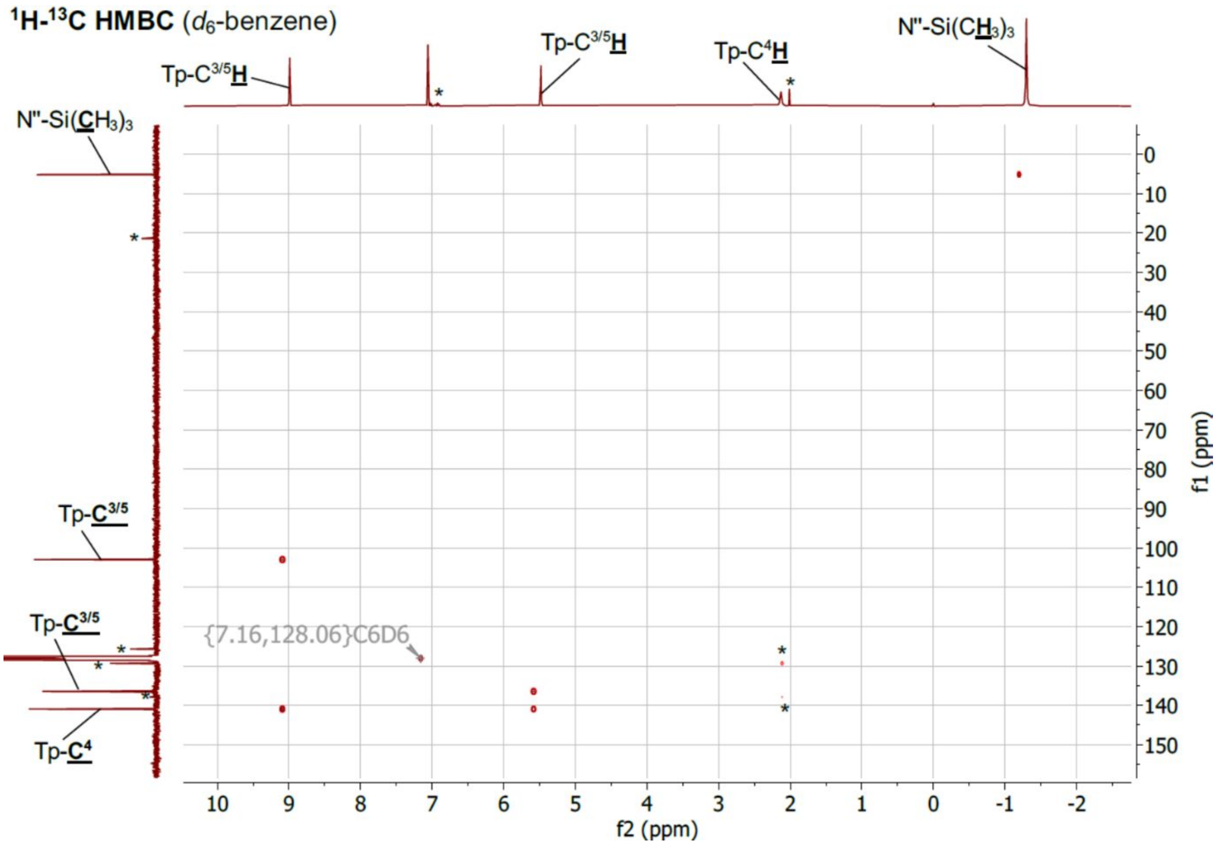


**Figure S 13.** $^1H$-$^{13}C$ HMBC spectrum of $[Sm(Tp)_2(N'')]$ **1-Sm**, recorded in $d_6$-benzene. Residual toluene and the corresponding cross-peaks for toluene are denoted with *.

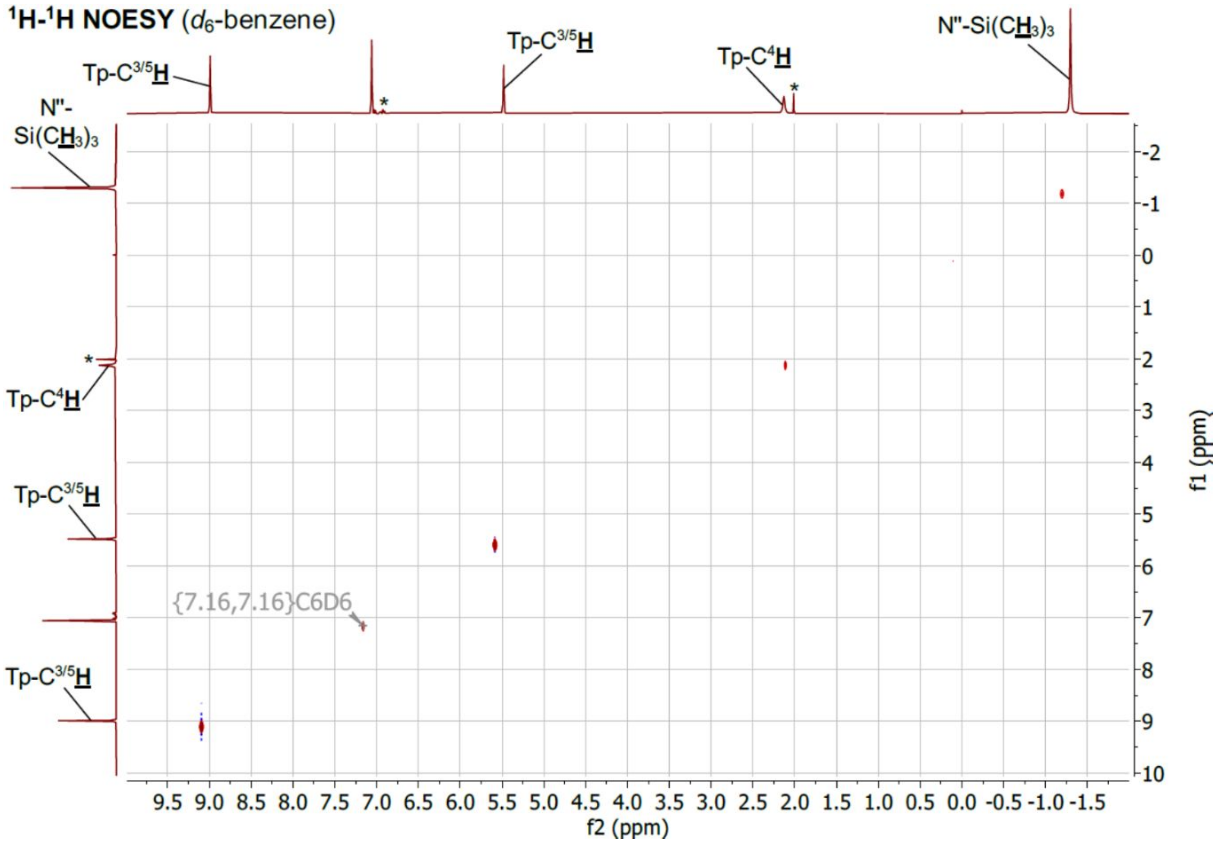


**Figure S 14.** $^1H$-$^1H$ NOESY spectrum of $[Sm(Tp)_2(N'')]$ **1-Sm**, recorded in $d_6$-benzene. Residual toluene is denoted with *.

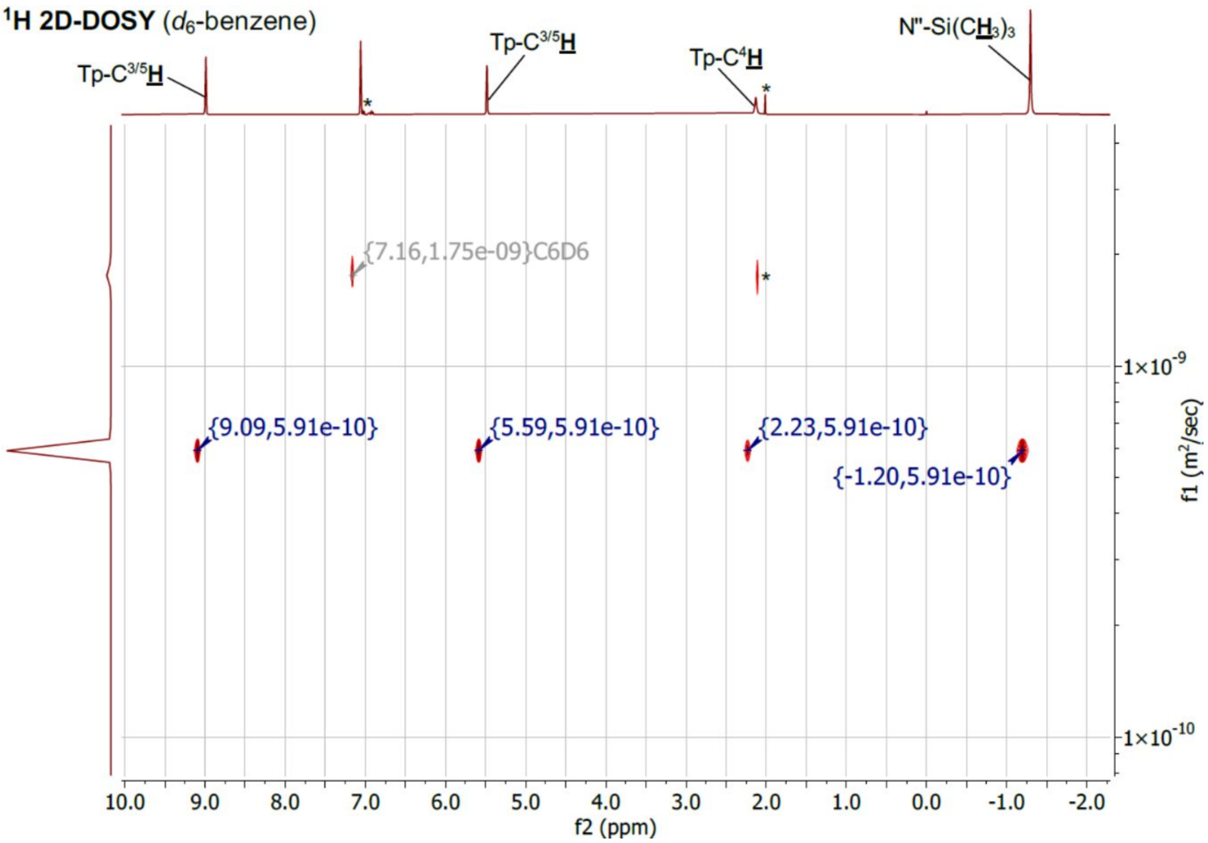


**Figure S 15.** $^{1}$H 2D-DOSY NMR spectrum of [Sm(Tp)$_2$(N'')] **1-Sm**, recorded in $d_6$-benzene. The horizontal scale shows $^{1}$H chemical shifts (ppm) and the vertical dimension the diffusion scale ($m^2s^{-1}$) with diffusion cross-peaks for **1-Sm** centred at 5.91 x $10^{-10}$ $m^2s^{-1}$. Residual toluene and the corresponding diffusion cross-peak for free toluene are denoted with *.

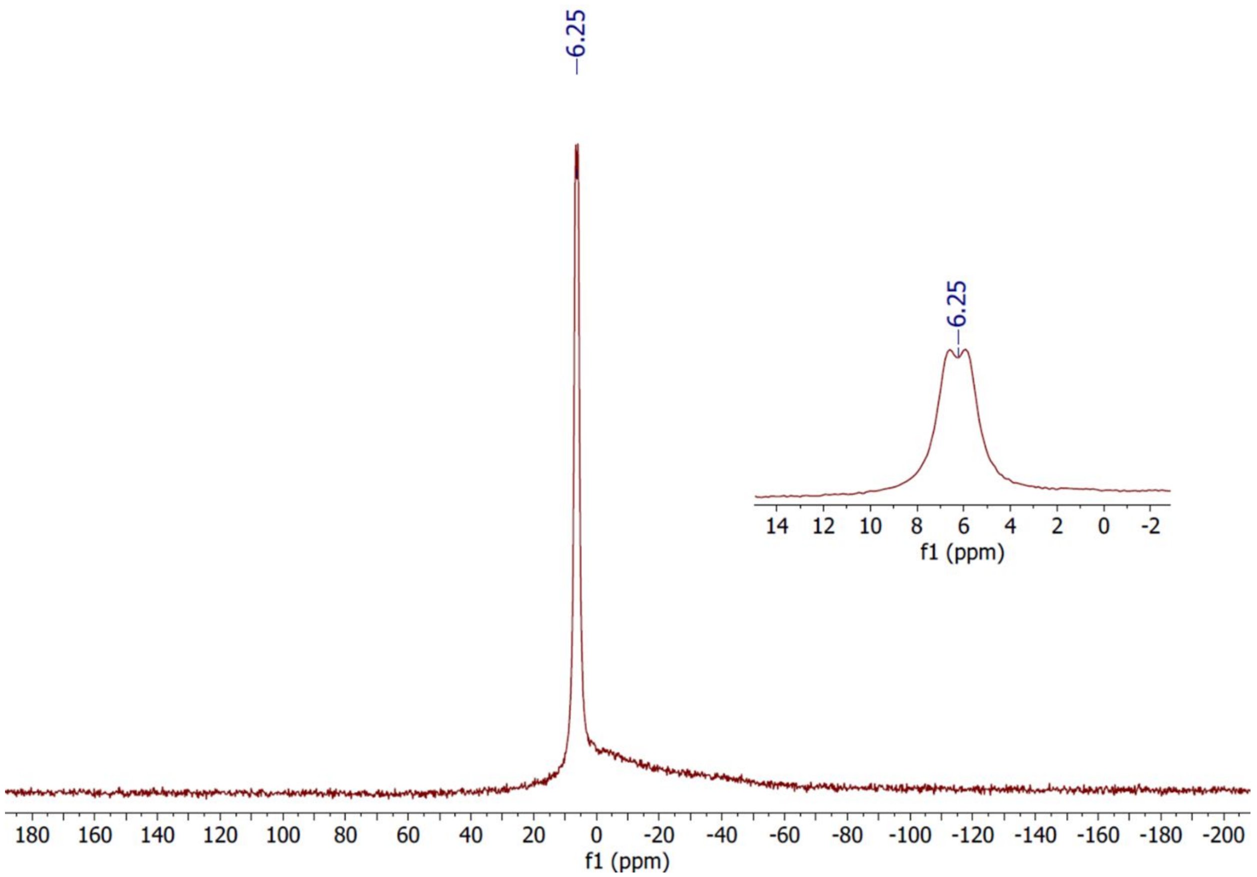


**Figure S 16.** $^{11}$B NMR spectrum of [Sm(Tp)$_2$(N'')] **1-Sm**, recorded in $d_6$-benzene.

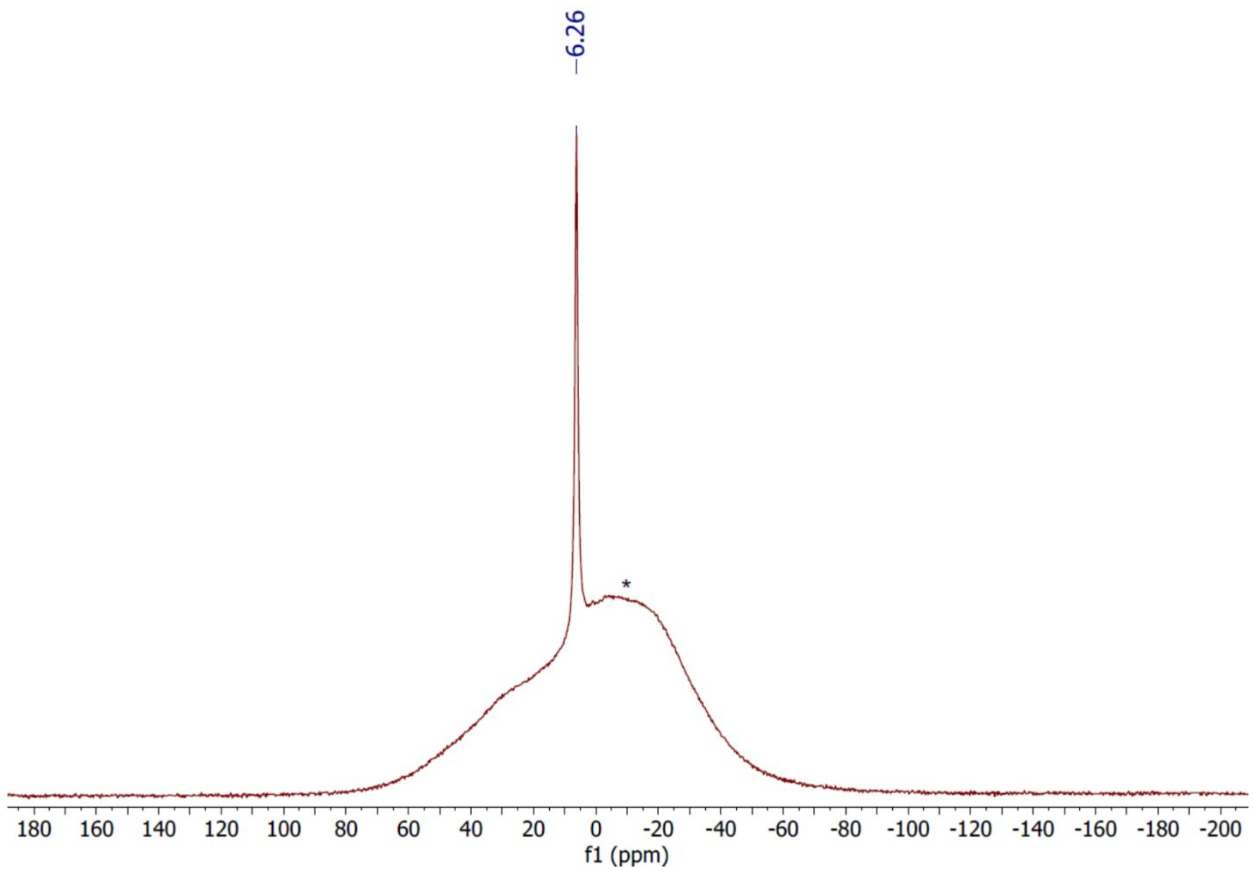


**Figure S 17.** $^{11}B\{^{1}H\}$ NMR spectrum of [Sm(Tp)$_2$(N")] **1-Sm**, recorded in $d_6$-benzene. Borosilicate glass is denoted with *.

### B1.4 $^{29}Si\{^{1}H\}$ INEPT NMR data for [Ln(Tp)$_2$(N")] 1-Ln (Ln = Y, Sm, Dy, Yb)

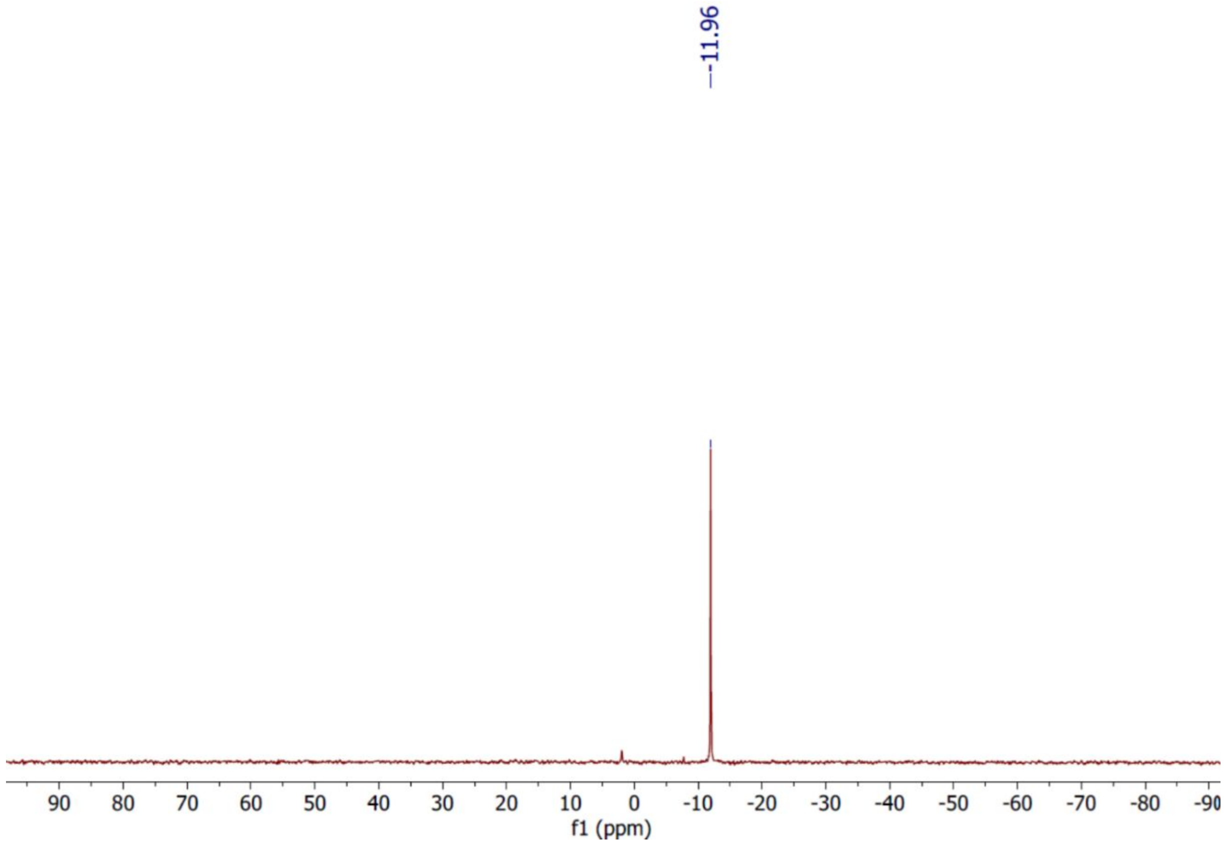


**Figure S 18.** $^{29}Si\{^{1}H\}$ INEPT NMR spectrum of [Y(Tp)$_2$(N")] **1-Y**, recorded in $d_6$-benzene.

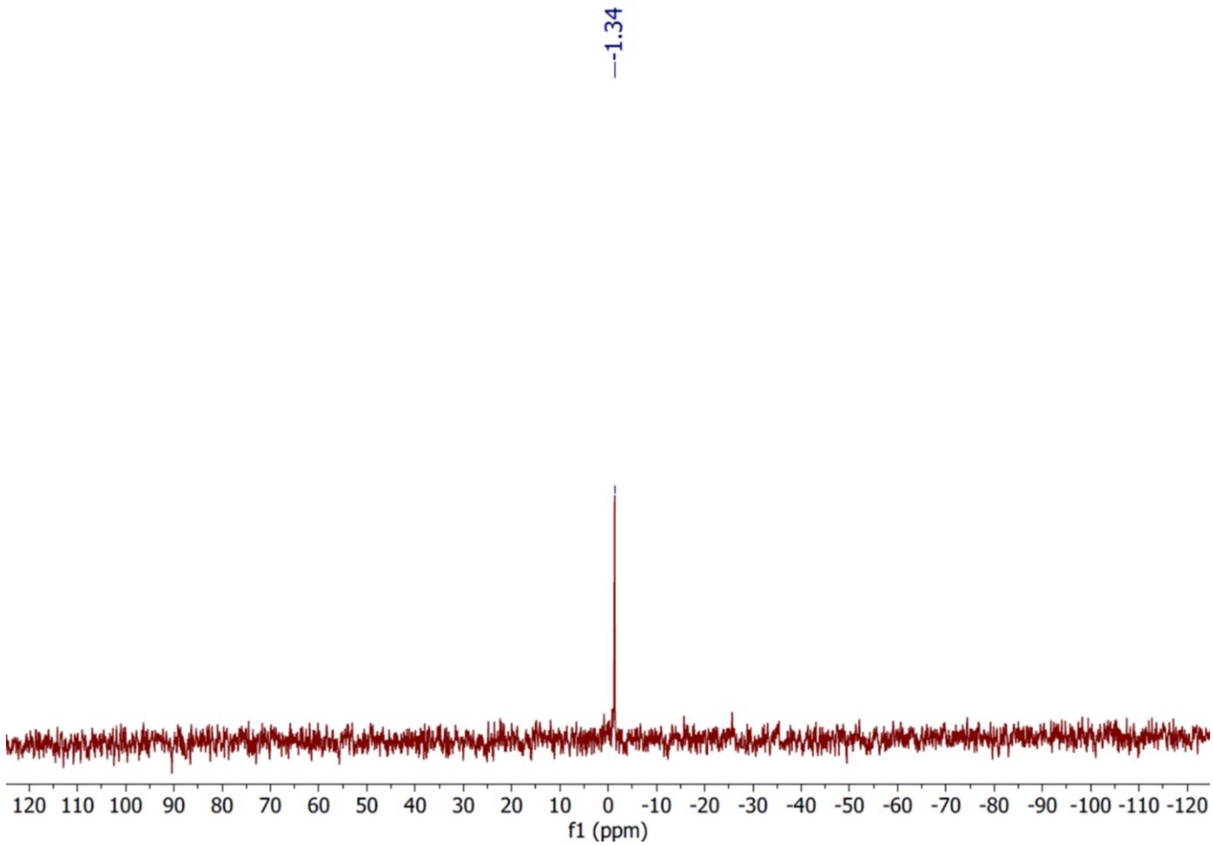


**Figure S 19.** $^{29}Si\{^{1}H\}$ INEPT NMR spectrum of $[Sm(Tp)_2(N'')]$ **1-Sm**, recorded in $d_6$-benzene.

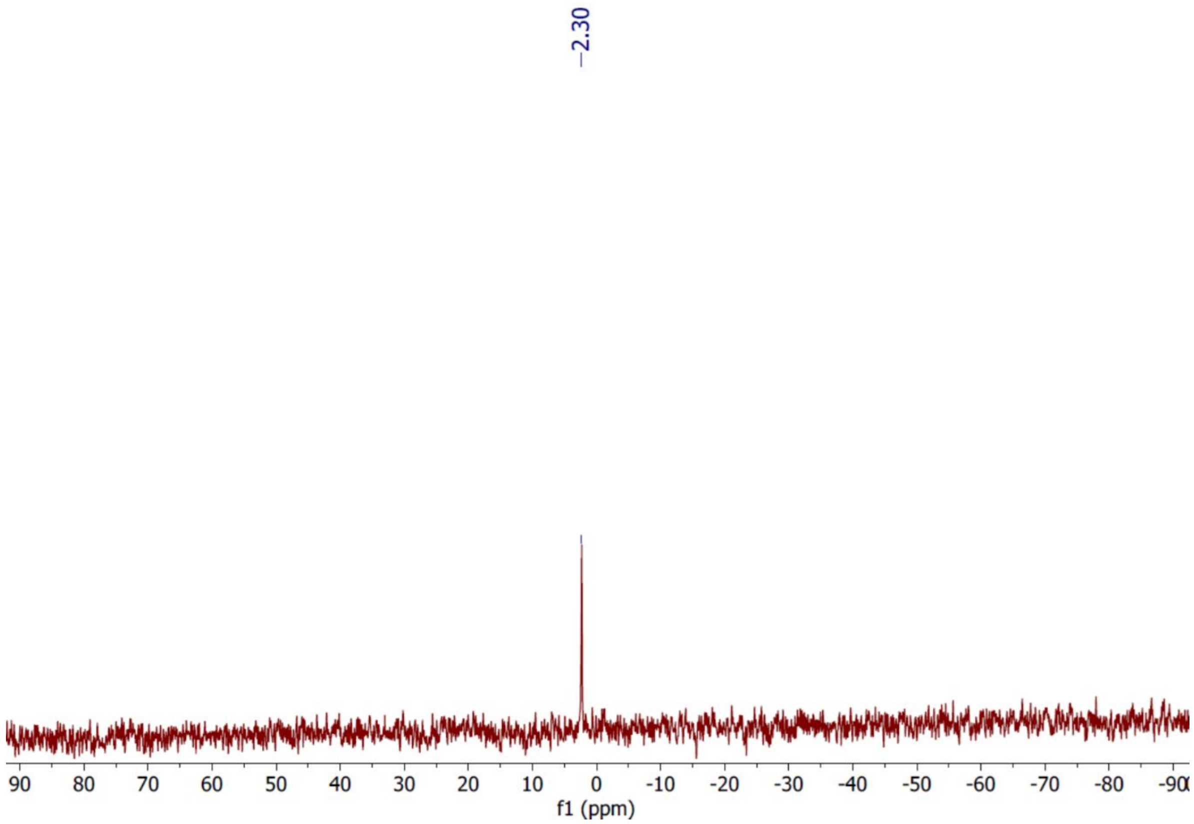


**Figure S 20.** $^{29}Si\{^{1}H\}$ INEPT NMR spectrum of $[Dy(Tp)_2(N'')]$ **1-Dy**, recorded in $d_6$-benzene.

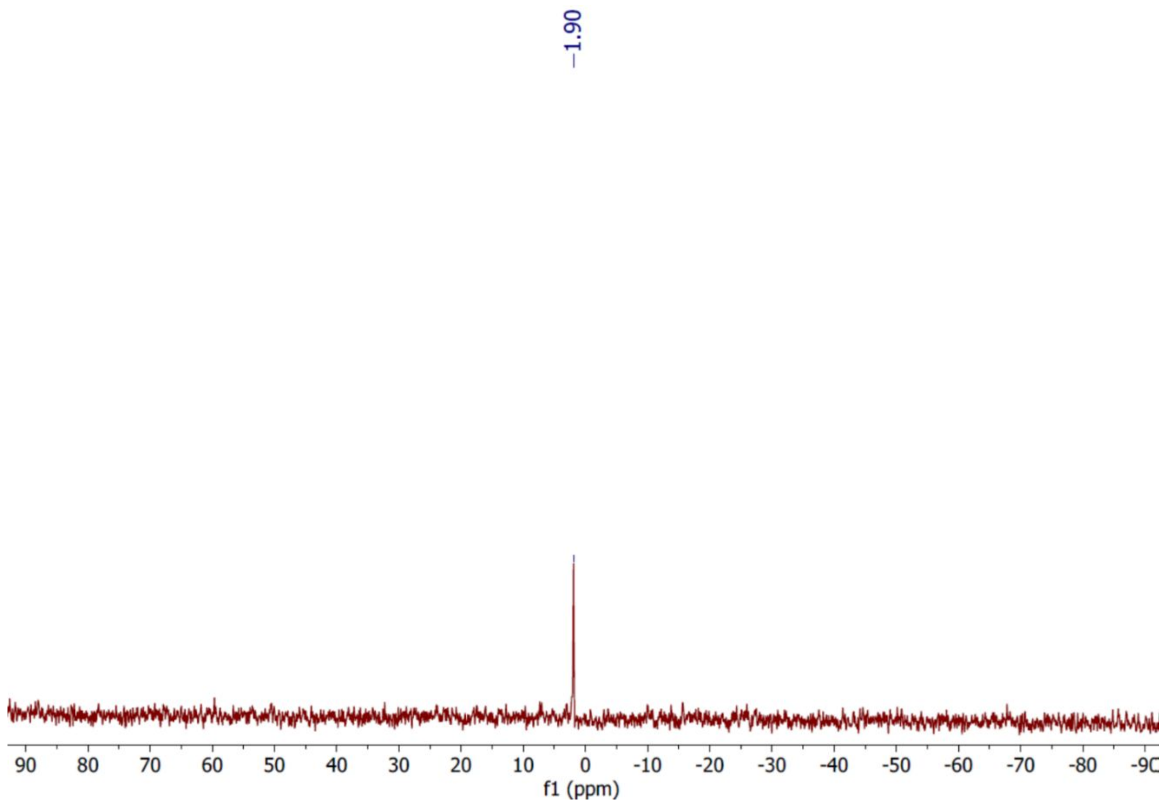


**Figure S 21.** $^{29}Si\{^{1}H\}$ INEPT NMR spectrum of [Yb(Tp)$_2$(N'')] **1-Yb**, recorded in $d_6$-benzene.

### B1.5 [Y(Tp)$_2$($\mu$-H)$_2$Al(H)(N'')] 2-Y

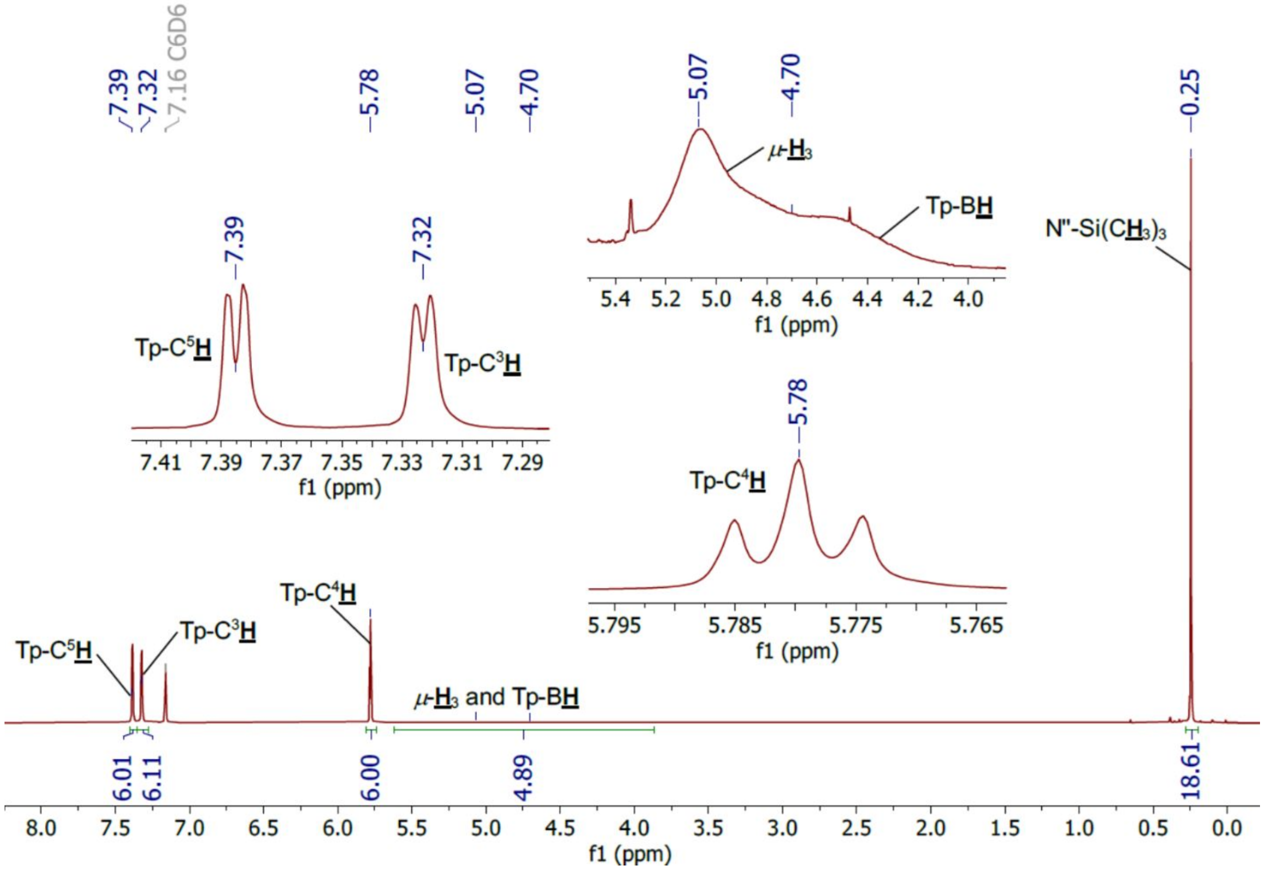


**Figure S 22.** $^{1}H$ NMR spectrum of [Y(Tp)$_2$($\mu$-H)$_2$Al(H)(N'')] **2-Y**, recorded in $d_6$-benzene.

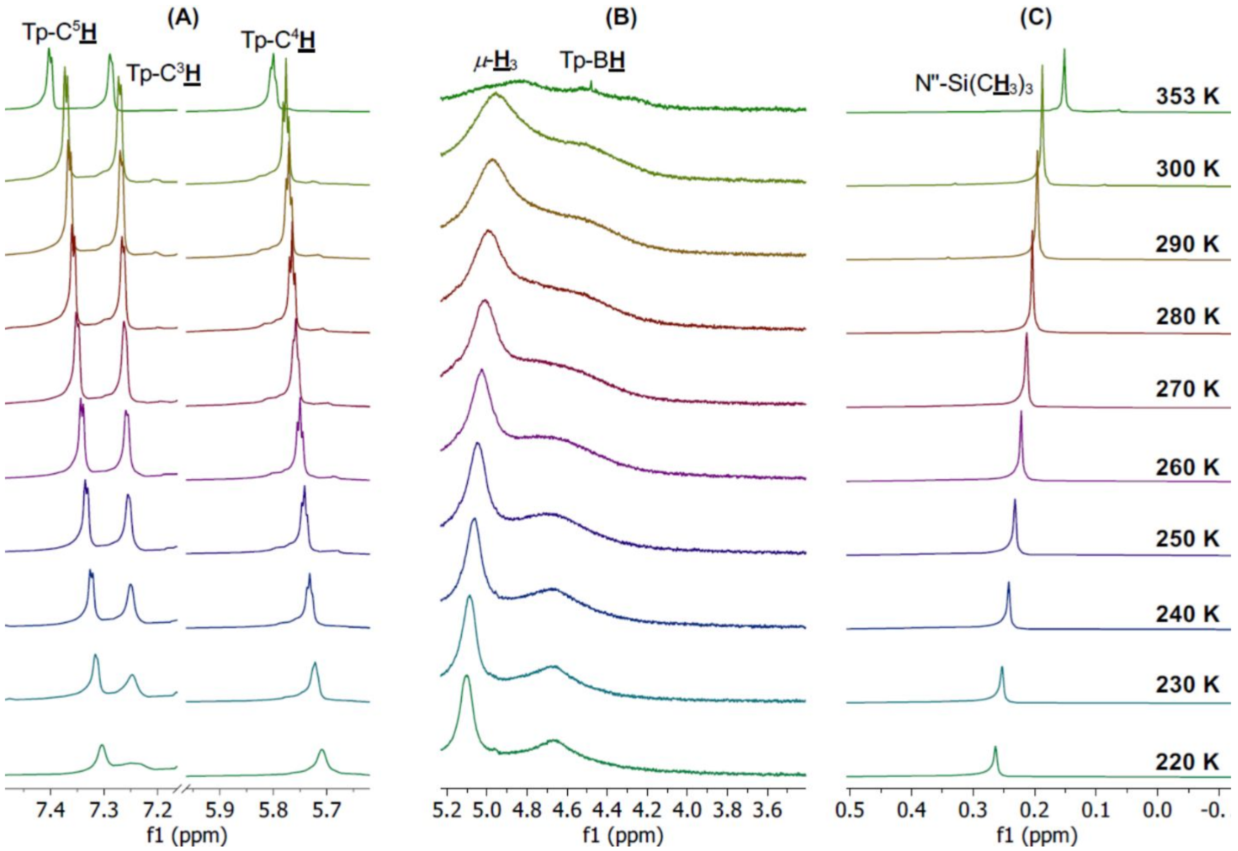


**Figure S 23.** Variable Temperature (VT) $^{1}$H NMR spectrum of $[Y(Tp)_2(\mu\text{-}H)_2Al(H)(N'')]$ **2-Y**, recorded in $d_8$-toluene, showing the regions of the pyrazolyl proton resonances (A), the hydride resonances (B), and the trimethylsilyl amide proton resonance (C) of **2-Y**.

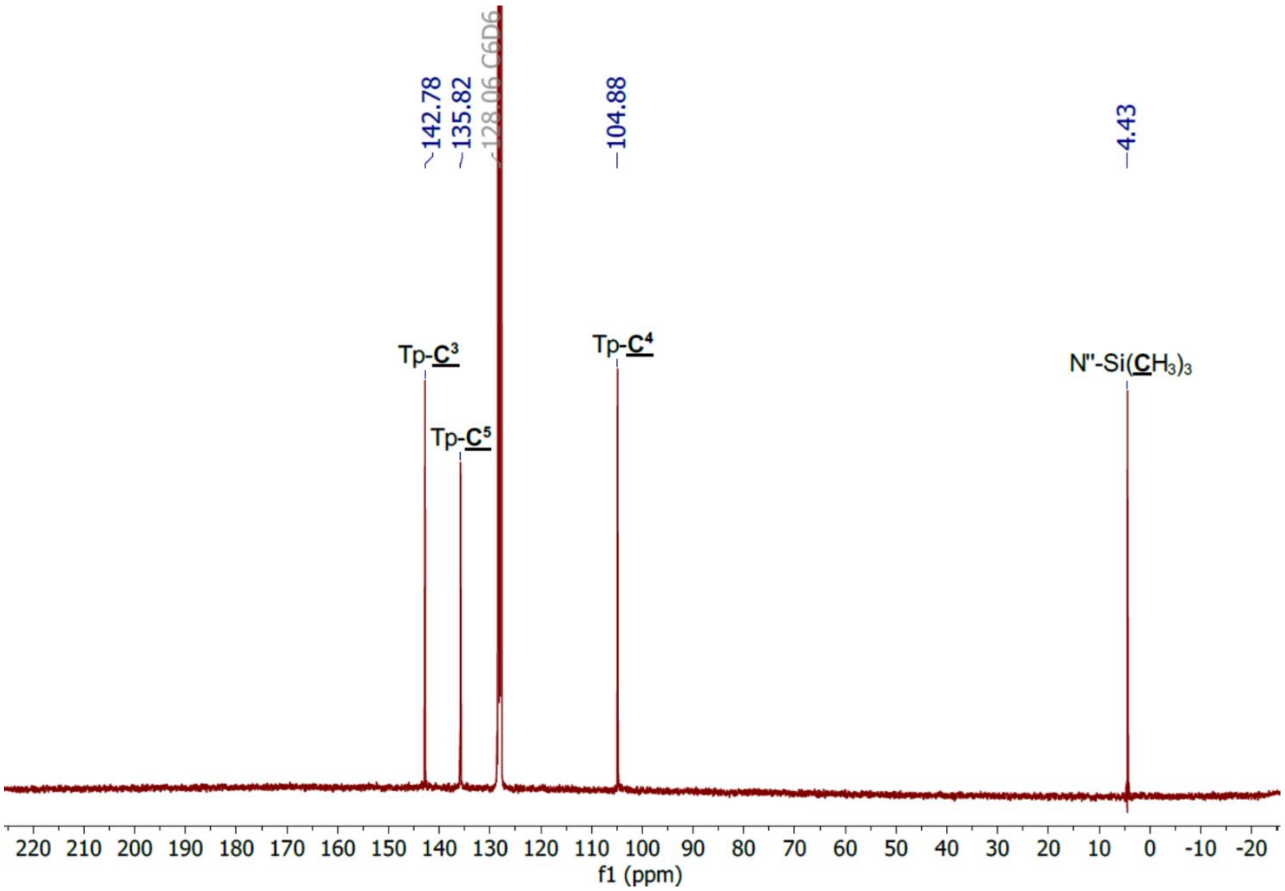


**Figure S 24.** $^{13}C\{^1H\}$ NMR spectrum of $[Y(Tp)_2(\mu\text{-}H)_2Al(H)(N'')]$ **2-Y**, recorded in $d_6$-benzene.

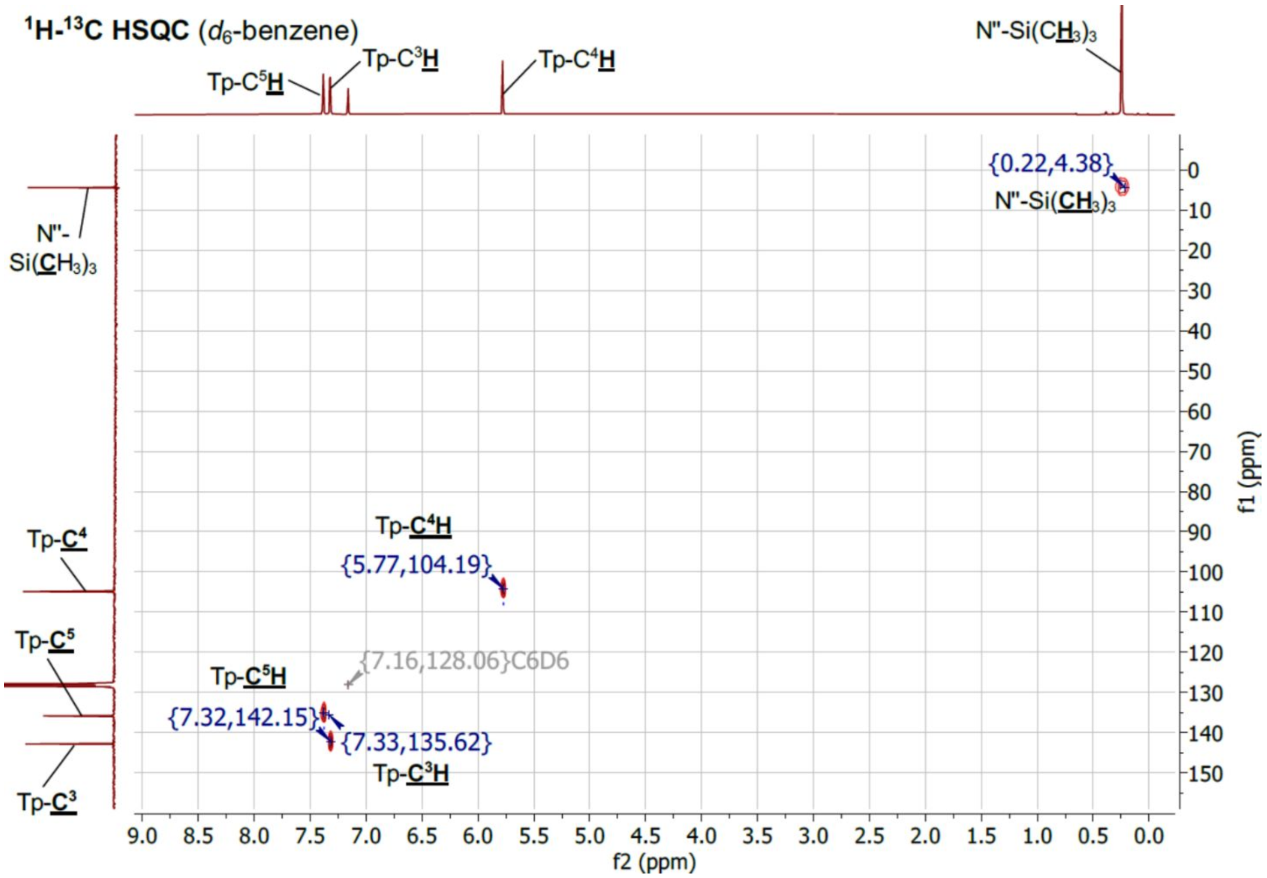


**Figure S 25.** $^1$H-$^{13}$C HSQC NMR spectrum of [Y(Tp)$_2$($\mu$-H)$_2$Al(H)(N'')] **2-Y**, recorded in $d_6$-benzene.

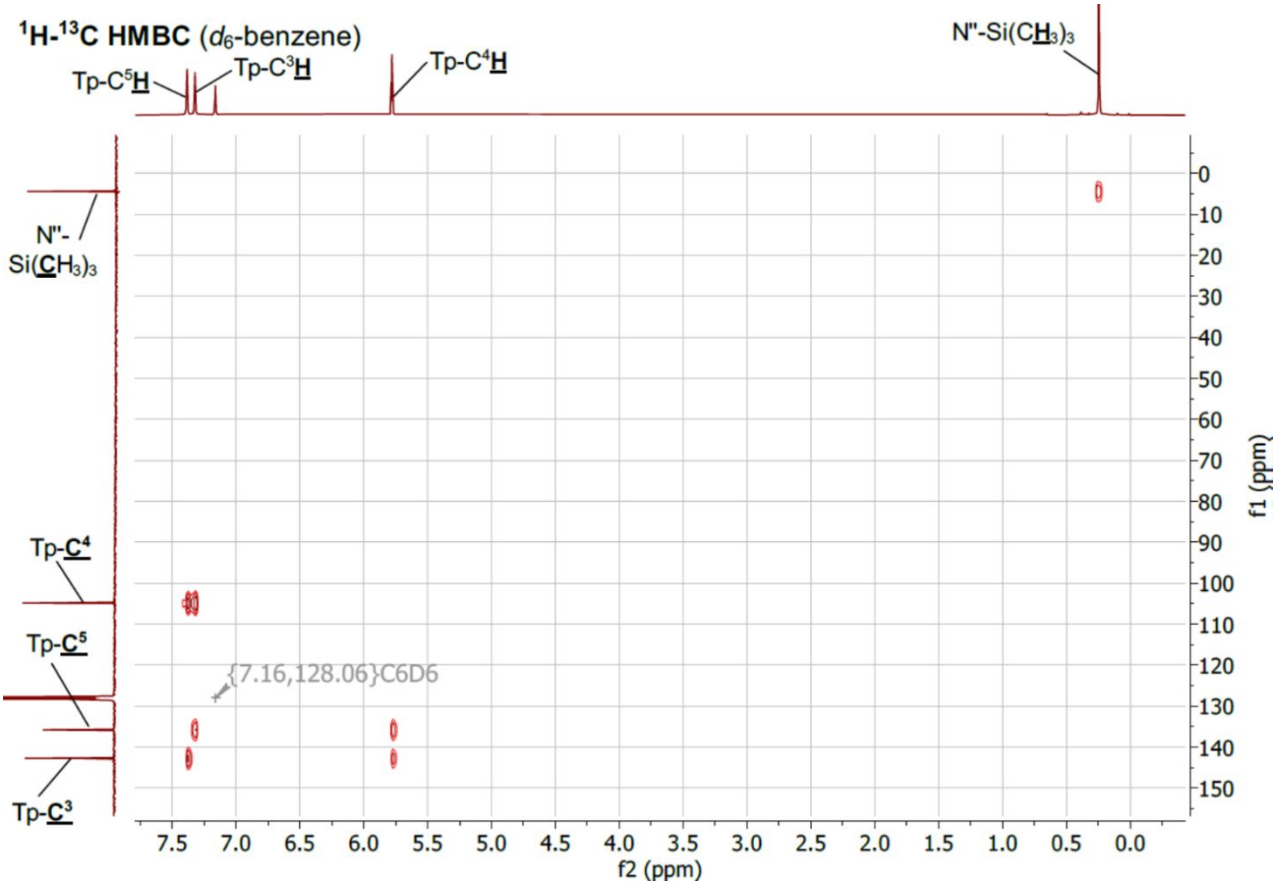


**Figure S 26.** $^1$H-$^{13}$C HMBC NMR spectrum of [Y(Tp)$_2$($\mu$-H)$_2$Al(H)(N'')] **2-Y**, recorded in $d_6$-benzene.

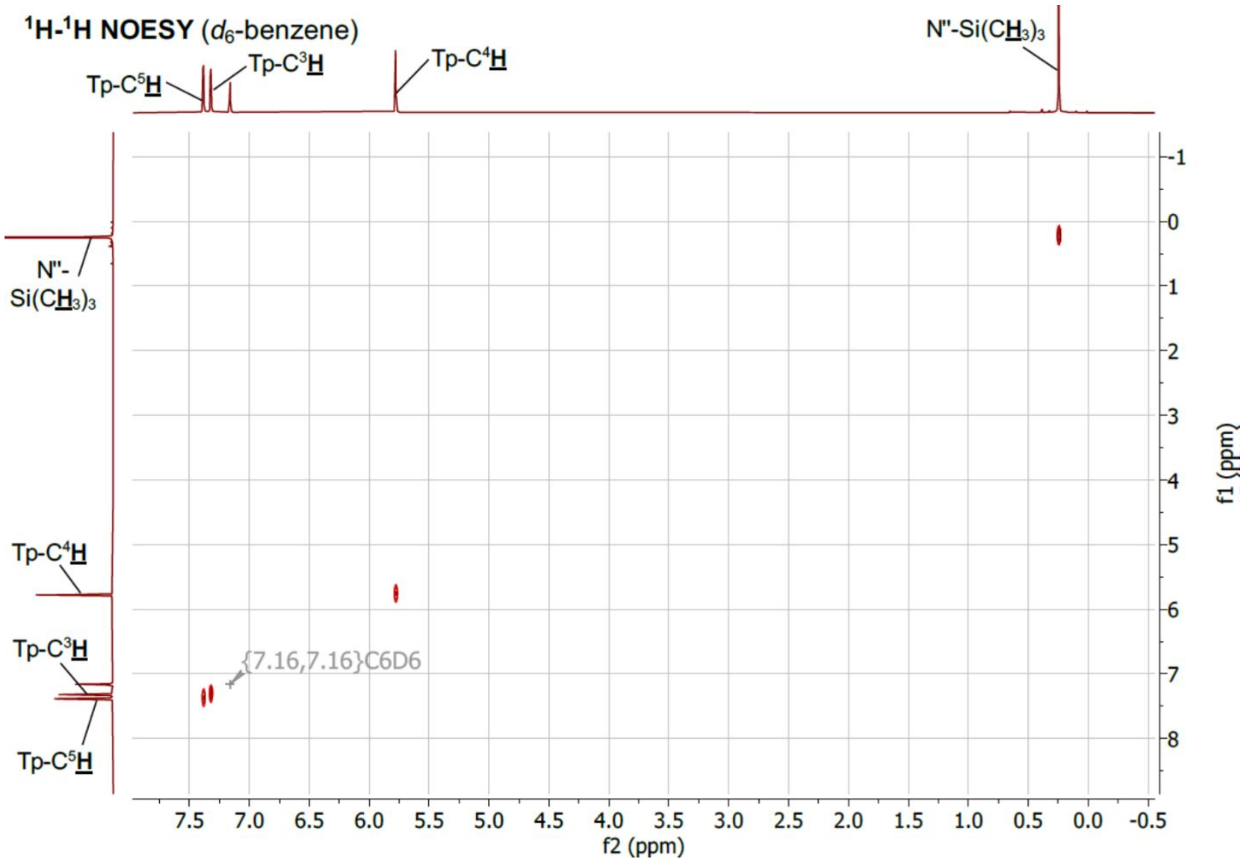


**Figure S 27.** $^{1}H$-$^{1}H$ NOESY NMR spectrum of $[Y(Tp)_2(\mu\text{-}H)_2Al(H)(N'')]$ **2-Y**, recorded in $d_6$-benzene.

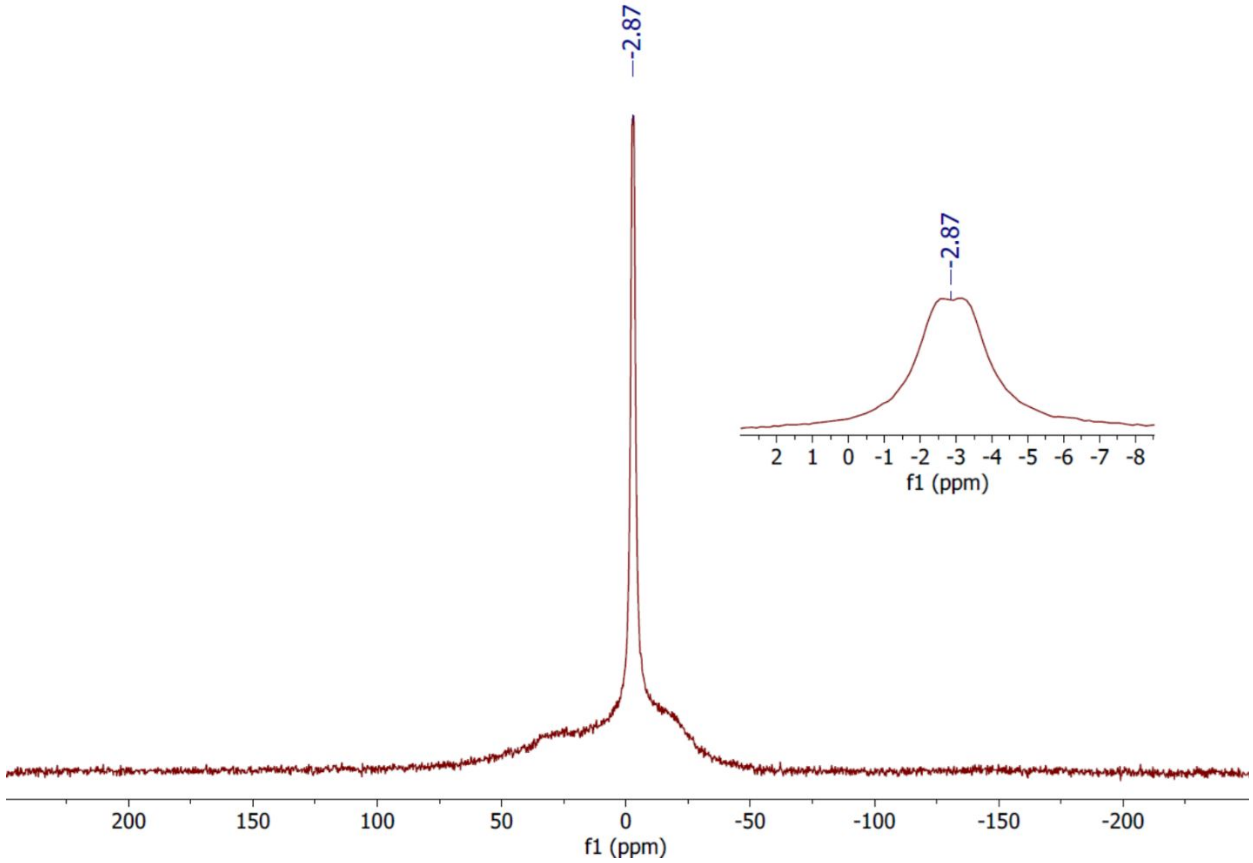


**Figure S 28.** $^{11}B$ NMR spectrum of $[Y(Tp)_2(\mu\text{-}H)_2Al(H)(N'')]$ **2-Y**, recorded in $d_6$-benzene.

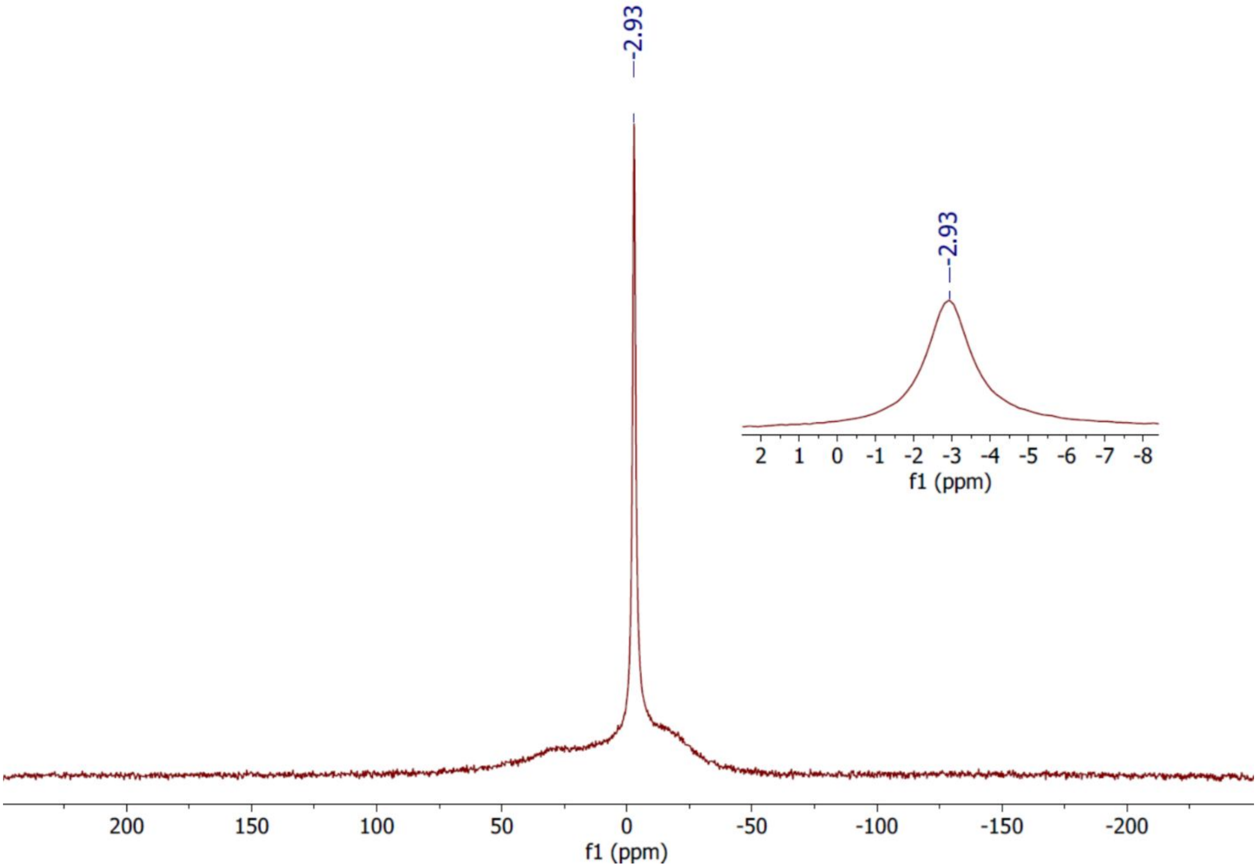


**Figure S 29.** $^{11}B\{^1H\}$ NMR spectrum of $[Y(Tp)_2(\mu\text{-}H)_2Al(H)(N'')]$ **2-Y**, recorded in $d_6$-benzene.

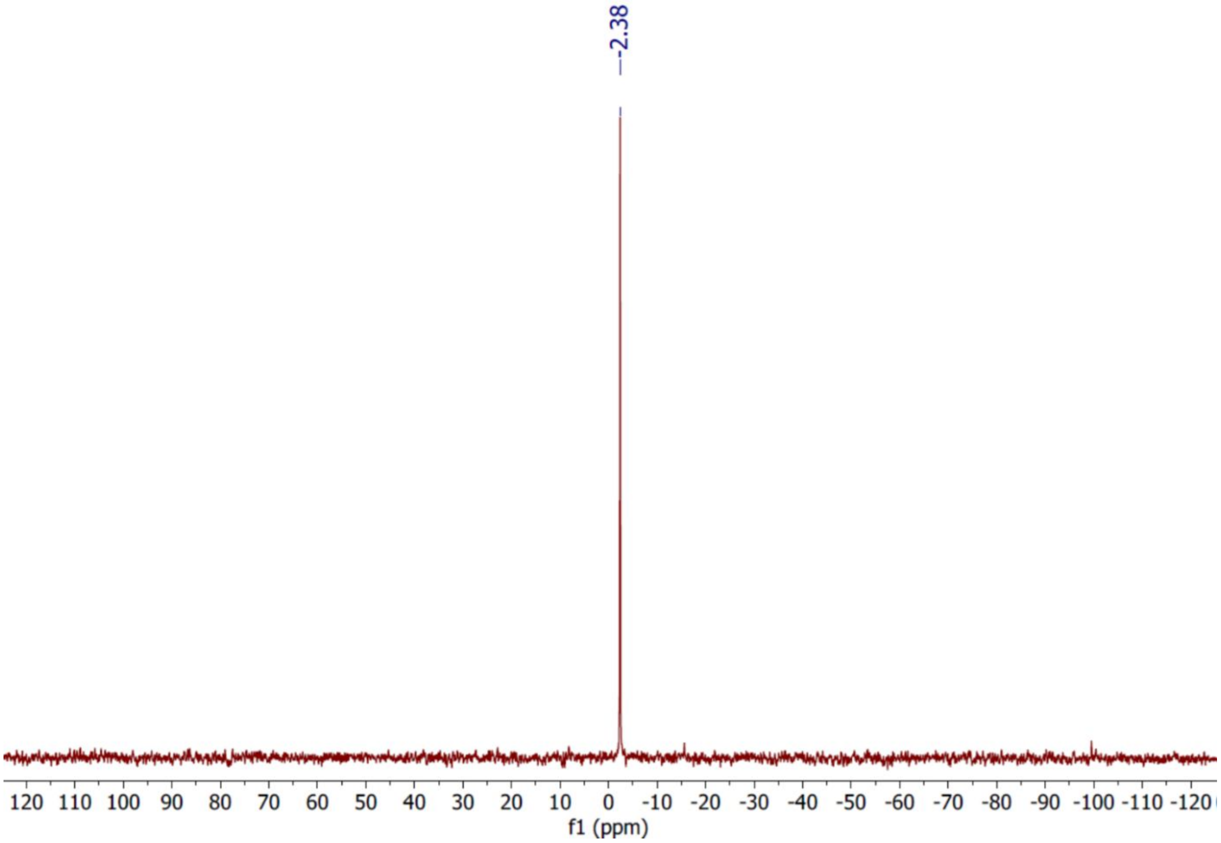


**Figure S 30.** $^{29}Si\{^1H\}$ INEPT NMR spectrum of $[Y(Tp)_2(\mu\text{-}H)_2Al(H)(N'')]$ **2-Y**, recorded in $d_6$-benzene.

### B1.6 [Sm(Tp)$_2$($\mu$-H)$_2$Al(H)(N'')] 2-Sm

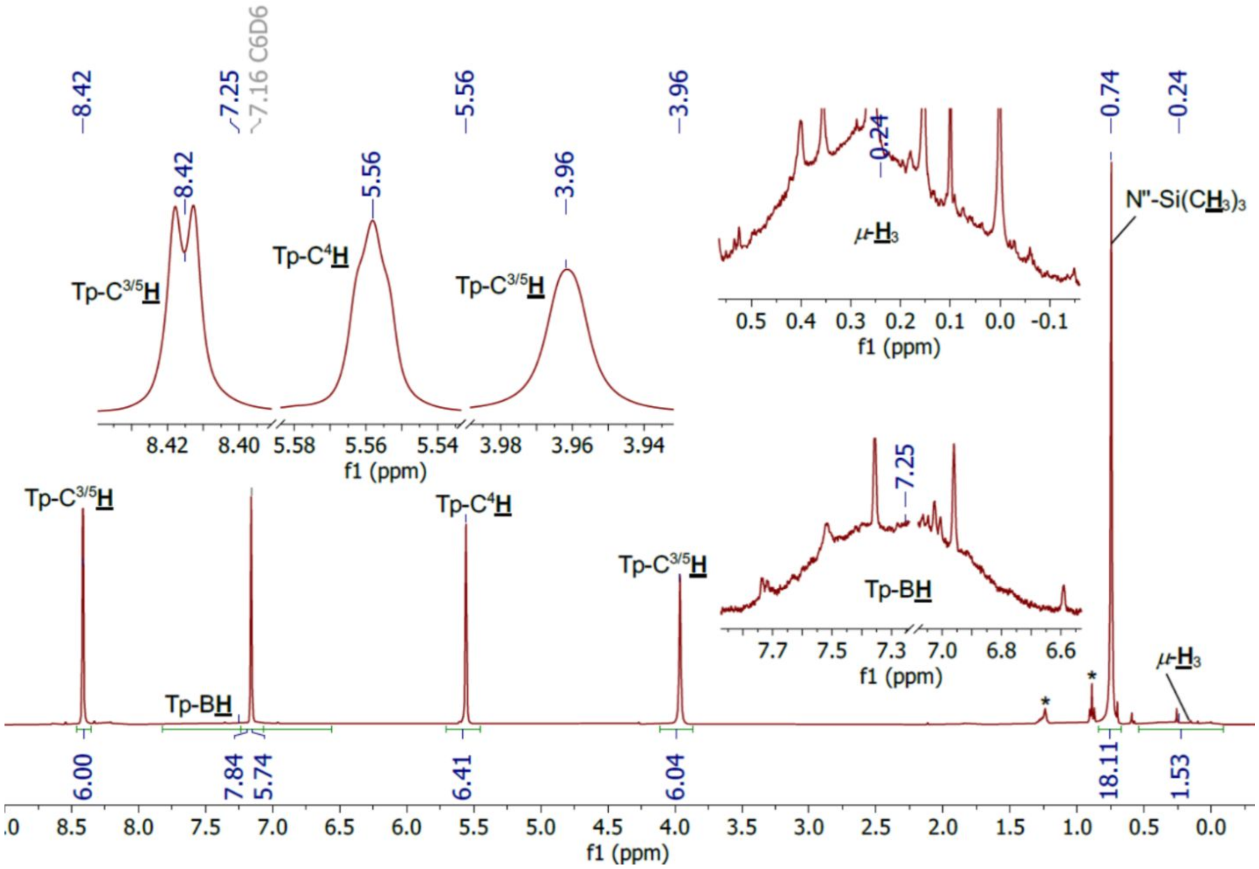


**Figure S 31.** $^1$H NMR spectrum of [Sm(Tp)$_2$($\mu$-H)$_2$Al(H)(N'')] **2-Sm**, recorded in $d_6$-benzene. Residual hexane is denoted with *.

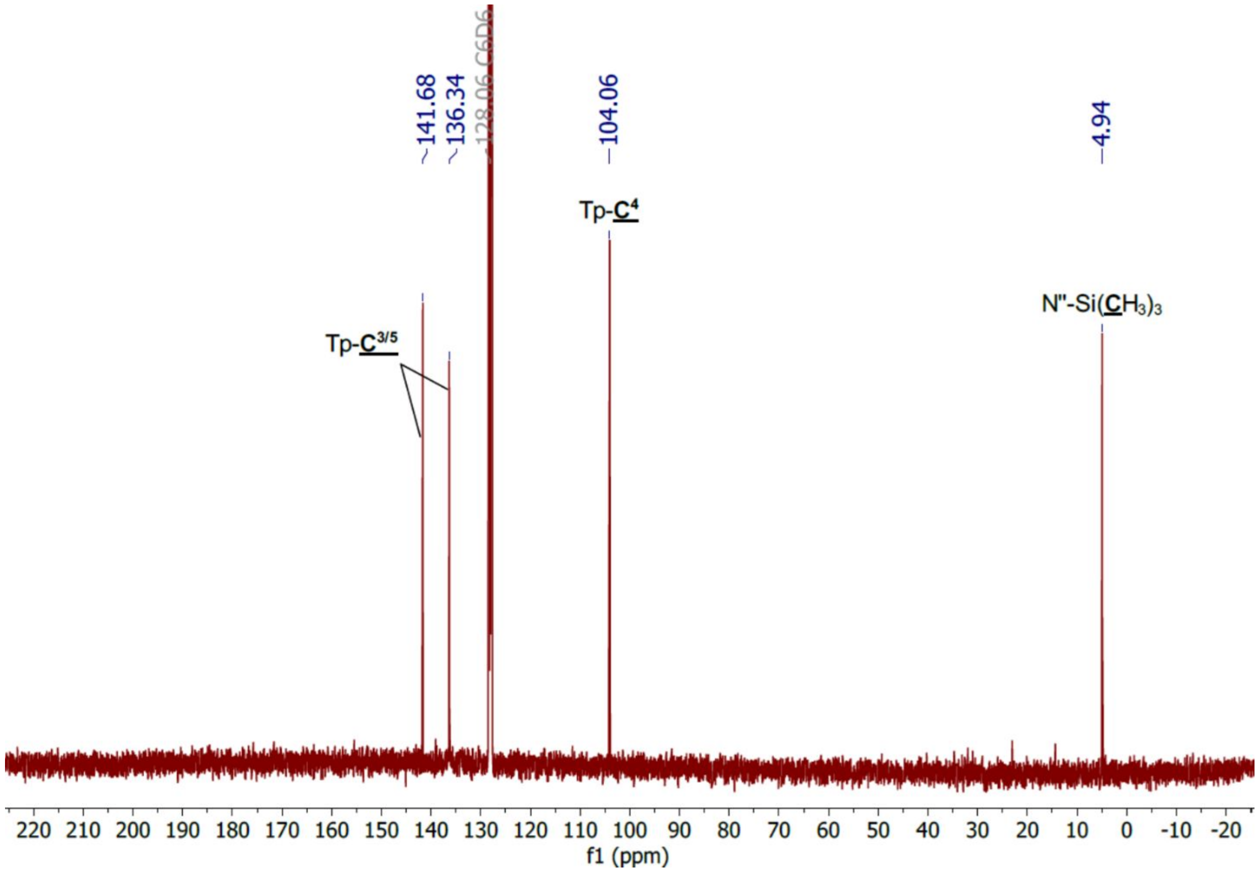


**Figure S 32.** $^{13}$C{$^1$H} NMR spectrum of [Sm(Tp)$_2$($\mu$-H)$_2$Al(H)(N'')] **2-Sm**, recorded in $d_6$-benzene.

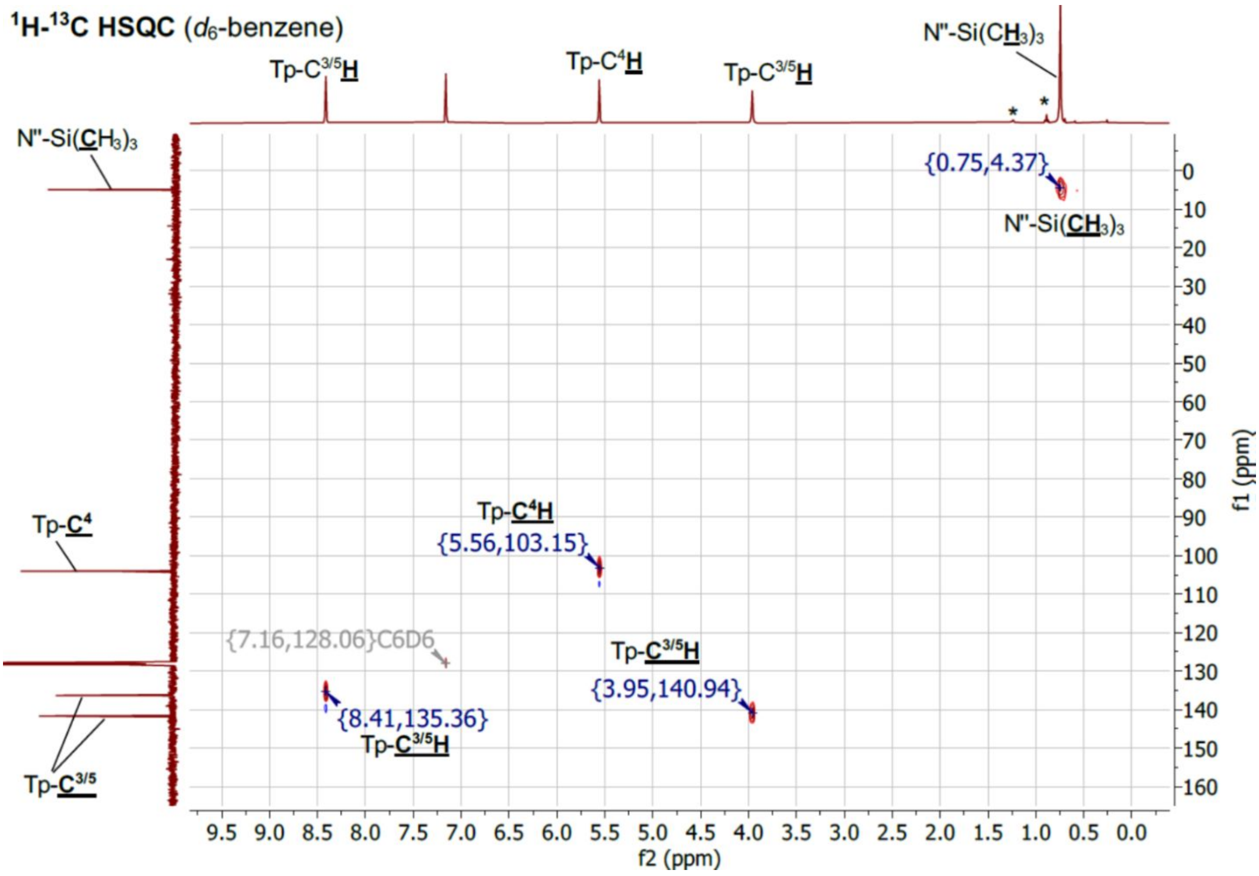


**Figure S 33.** $^{1}$H-$^{13}$C HSQC NMR spectrum of [Sm(Tp)$_2$($\mu$-H)$_2$Al(H)(N'')] **2-Sm**, recorded in $d_6$-benzene. Residual hexane is denoted with *.

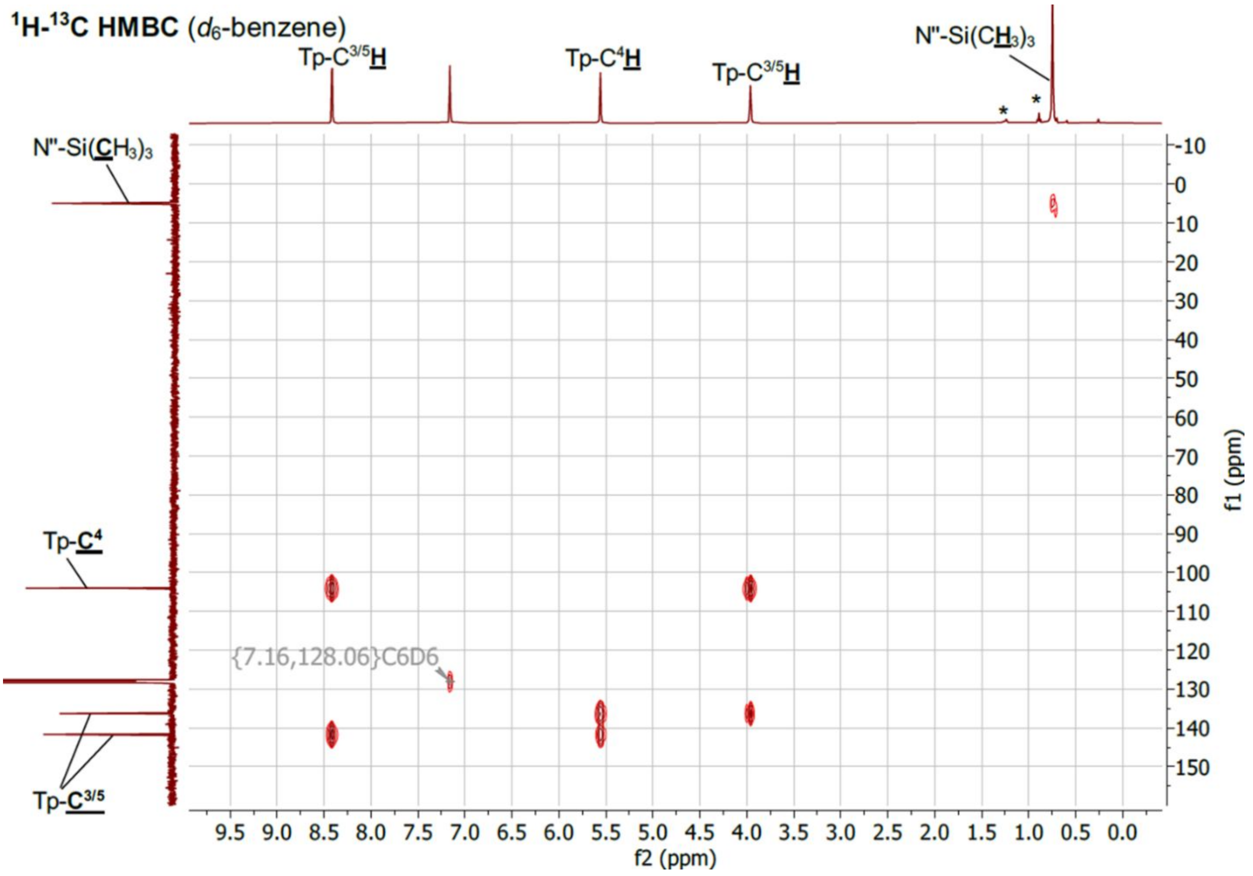


**Figure S 34.** $^{1}$H-$^{13}$C HMBC NMR spectrum of [Sm(Tp)$_2$($\mu$-H)$_2$Al(H)(N'')] **2-Sm**, recorded in $d_6$-benzene. Residual hexane is denoted with *.

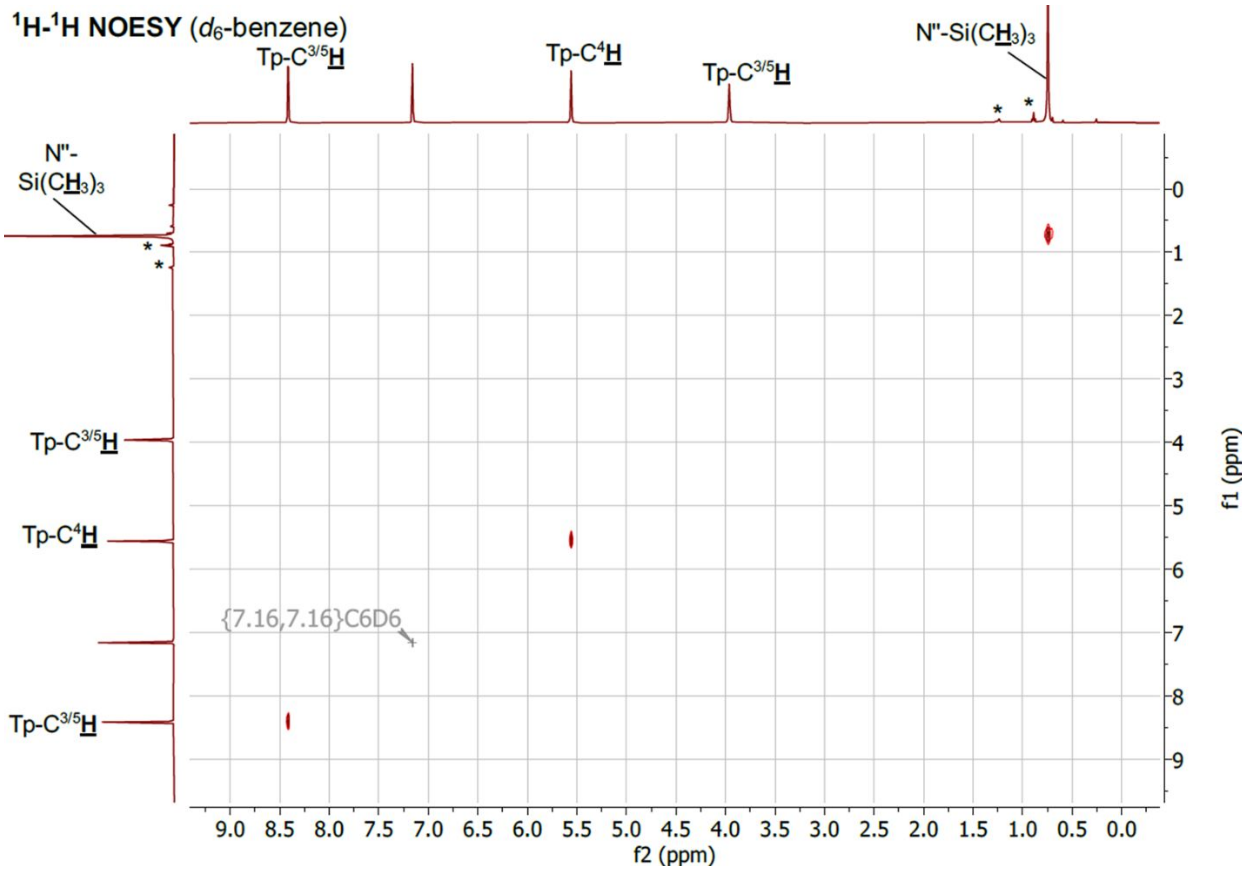


**Figure S 35.** $^{1}H$-$^{1}H$ NOESY NMR spectrum of [Sm(Tp)$_2$($\mu$-H)$_2$Al(H)(N")] **2-Sm**, recorded in $d_6$-benzene. Residual hexane is denoted with *.

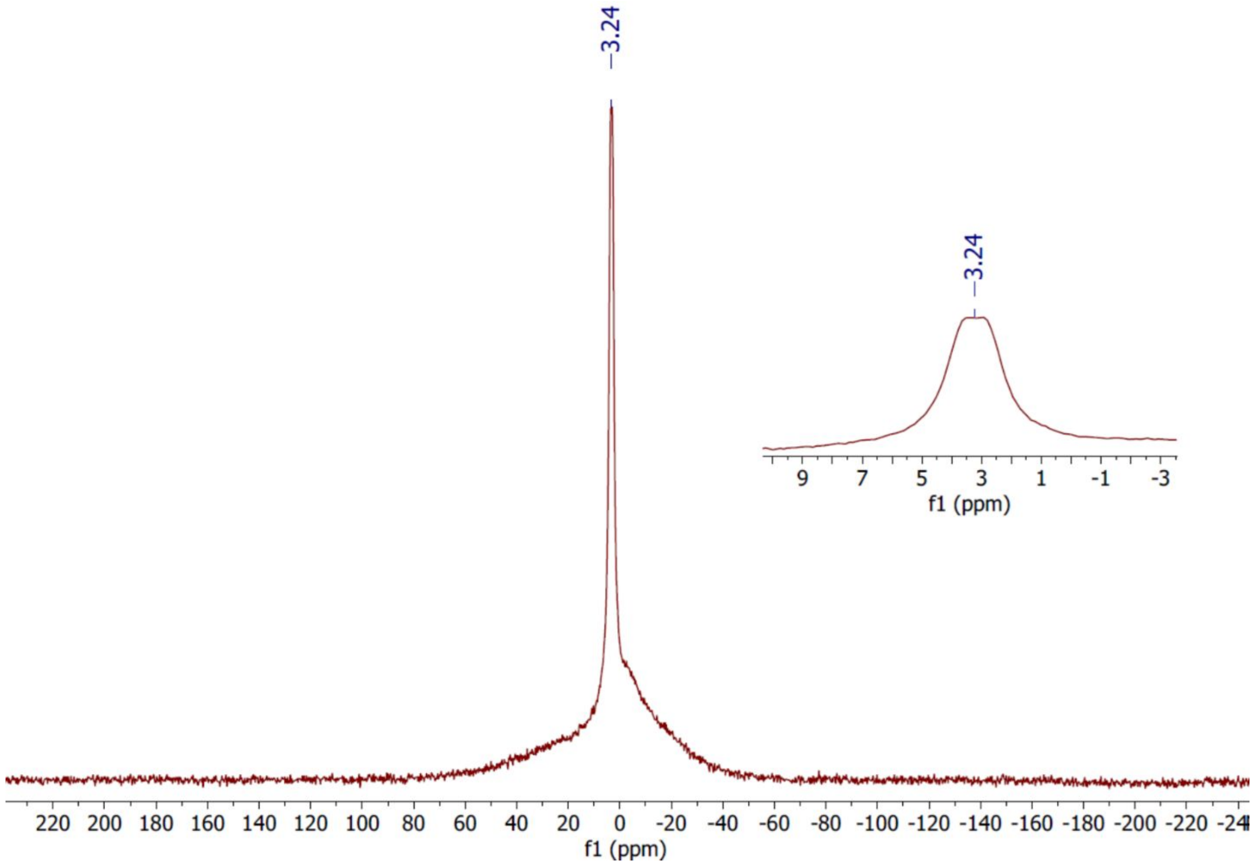


**Figure S 36.** $^{11}B$ NMR spectrum of [Sm(Tp)$_2$($\mu$-H)$_2$Al(H)(N")] **2-Sm**, recorded in $d_6$-benzene

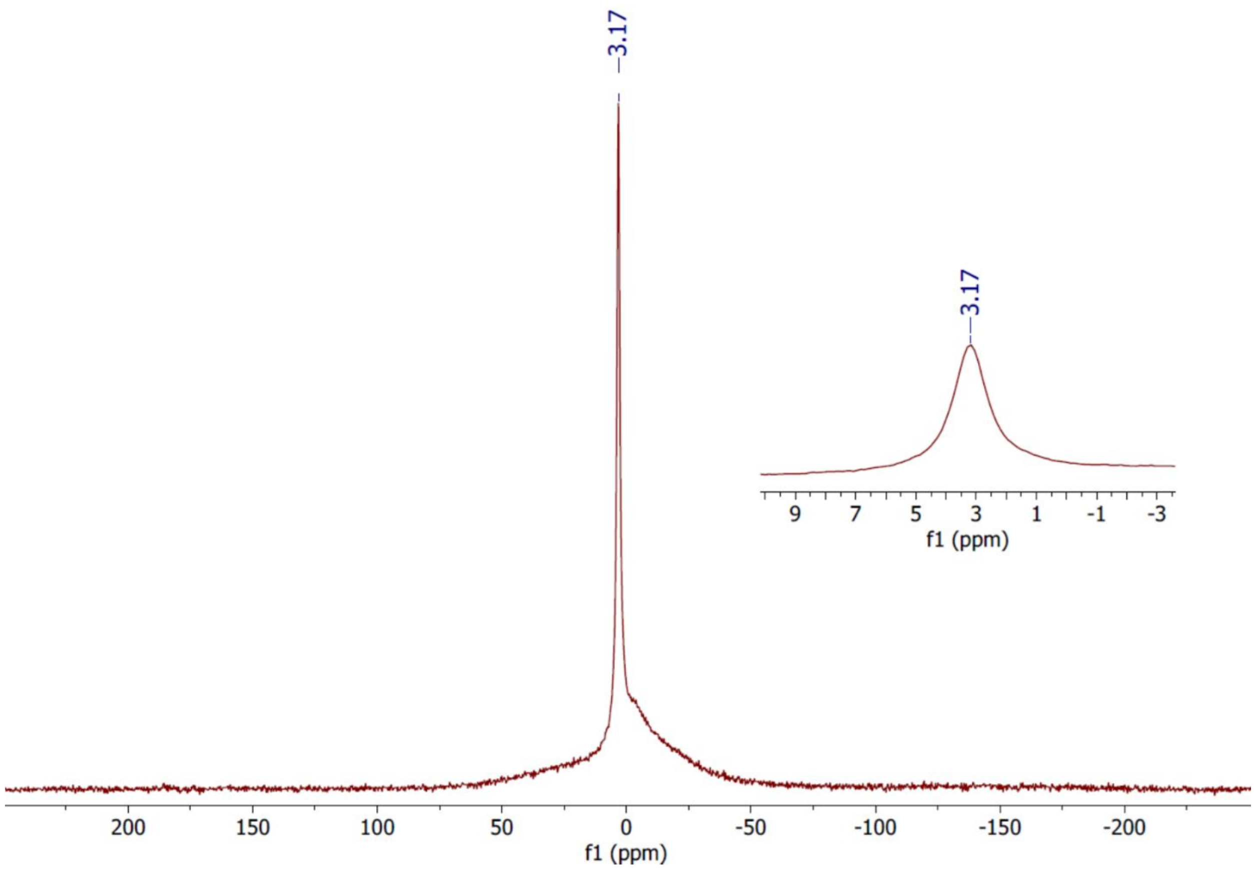


**Figure S 37.** $^{11}B\{^1H\}$ NMR spectrum of [Sm(Tp)$_2$($\mu$-H)$_2$Al(H)(N")] **2-Sm**, recorded in $d_6$-benzene

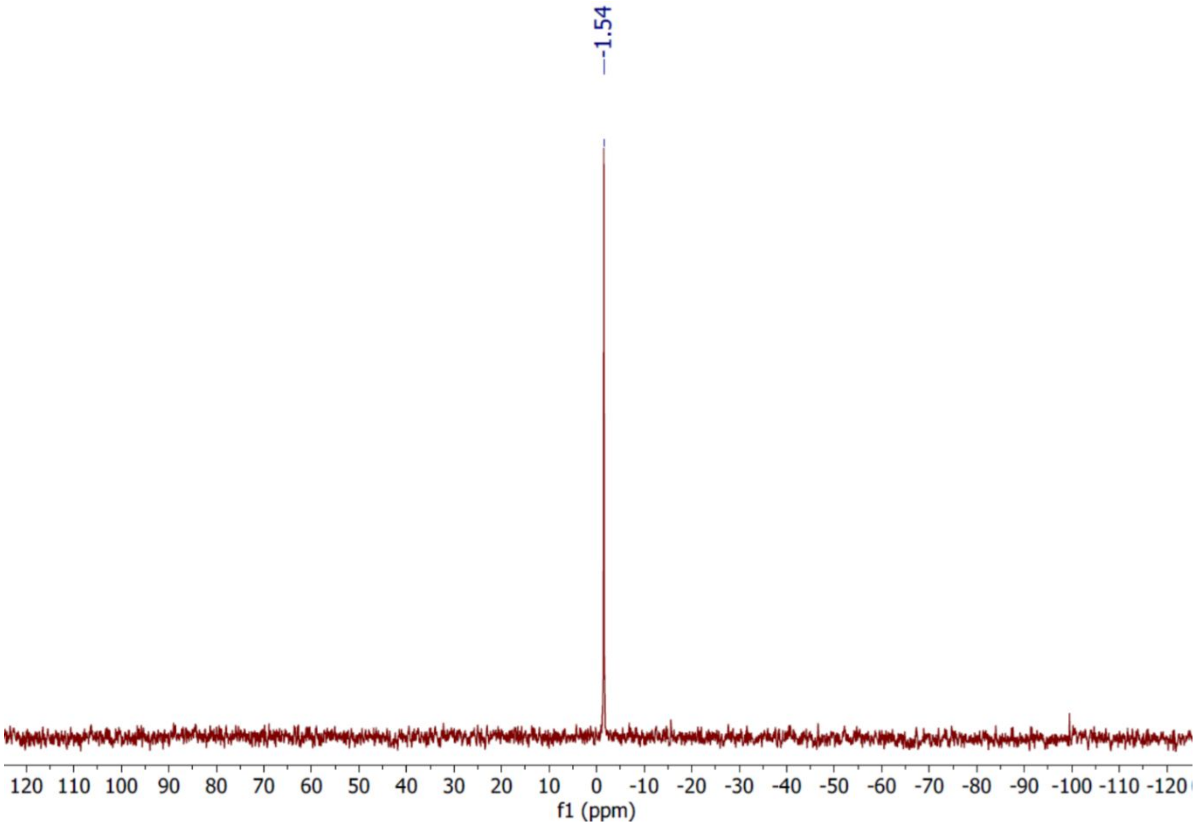


**Figure S 38.** $^{29}Si\{^1H\}$ INEPT NMR spectrum of [Sm(Tp)$_2$($\mu$-H)$_2$Al(H)(N")] **2-Sm**, recorded in $d_6$-benzene.

## B1.7 [Dy(Tp)$_2$($\mu$-H)$_2$Al(H)(N")] 2-Dy

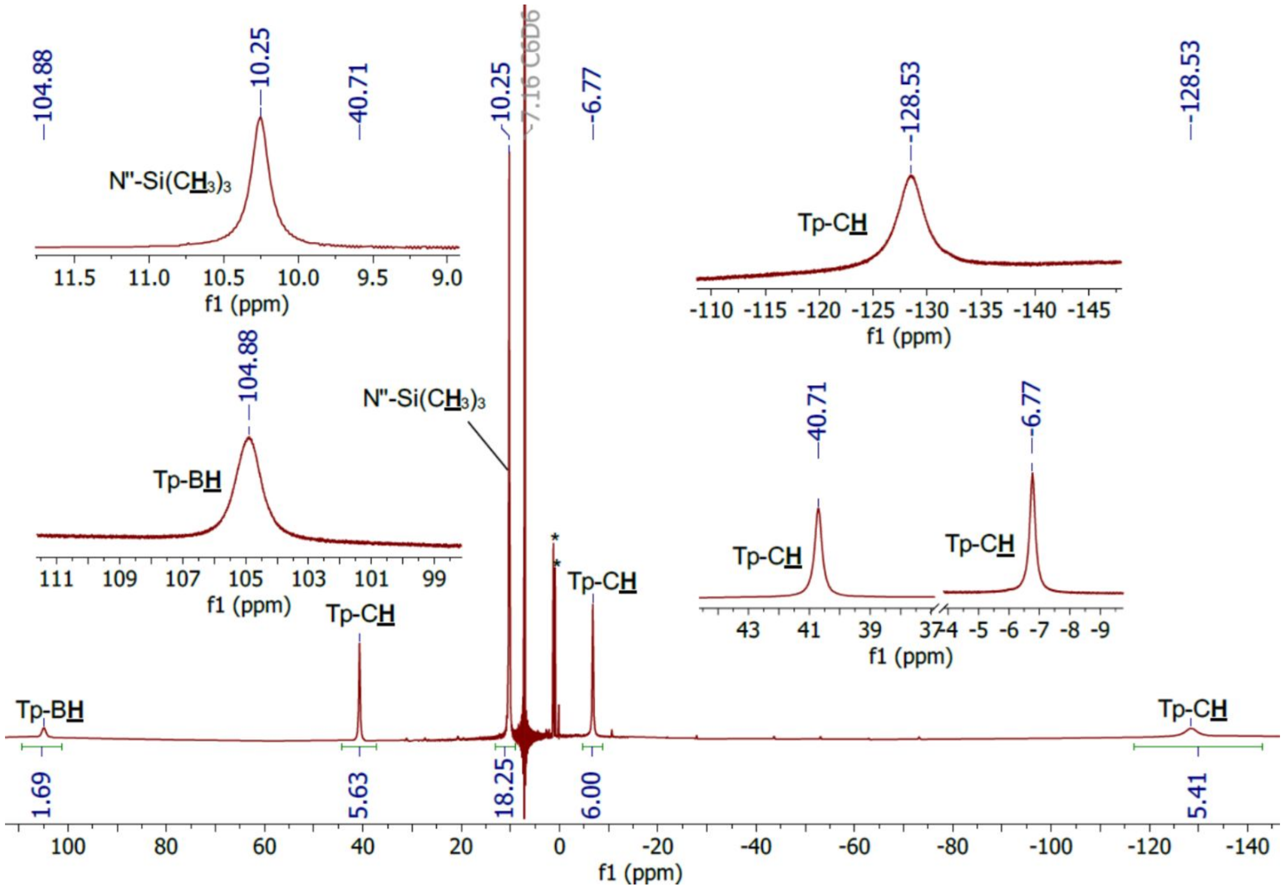


**Figure S 39.** $^{1}$H NMR spectrum of [Dy(Tp)$_2$($\mu$-H)$_2$Al(H)(N")] **1-Dy**, recorded in $d_6$-benzene. Residual hexane is denoted with *.

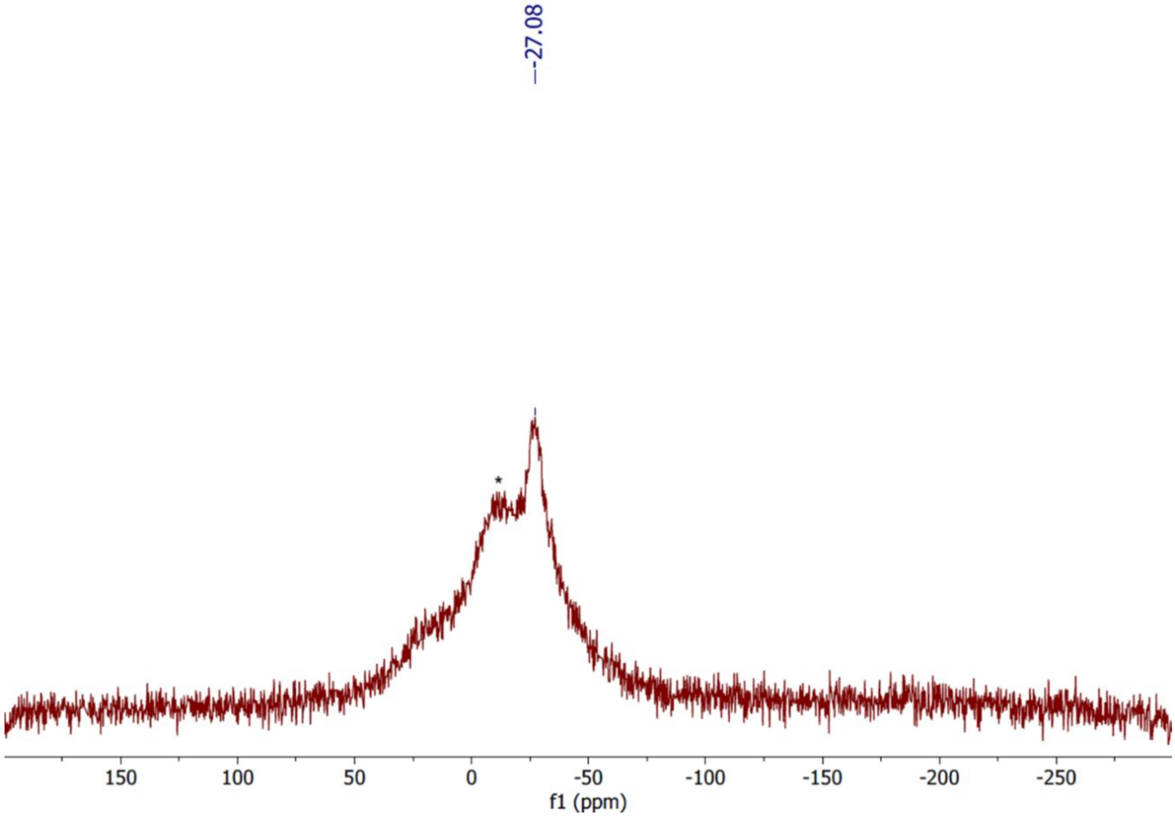


**Figure S 40.** $^{11}$B NMR spectrum of [Dy(Tp)$_2$($\mu$-H)$_2$Al(H)(N")] **1-Dy**, recorded in $d_6$-benzene. Borosilicate glass is denoted with *.

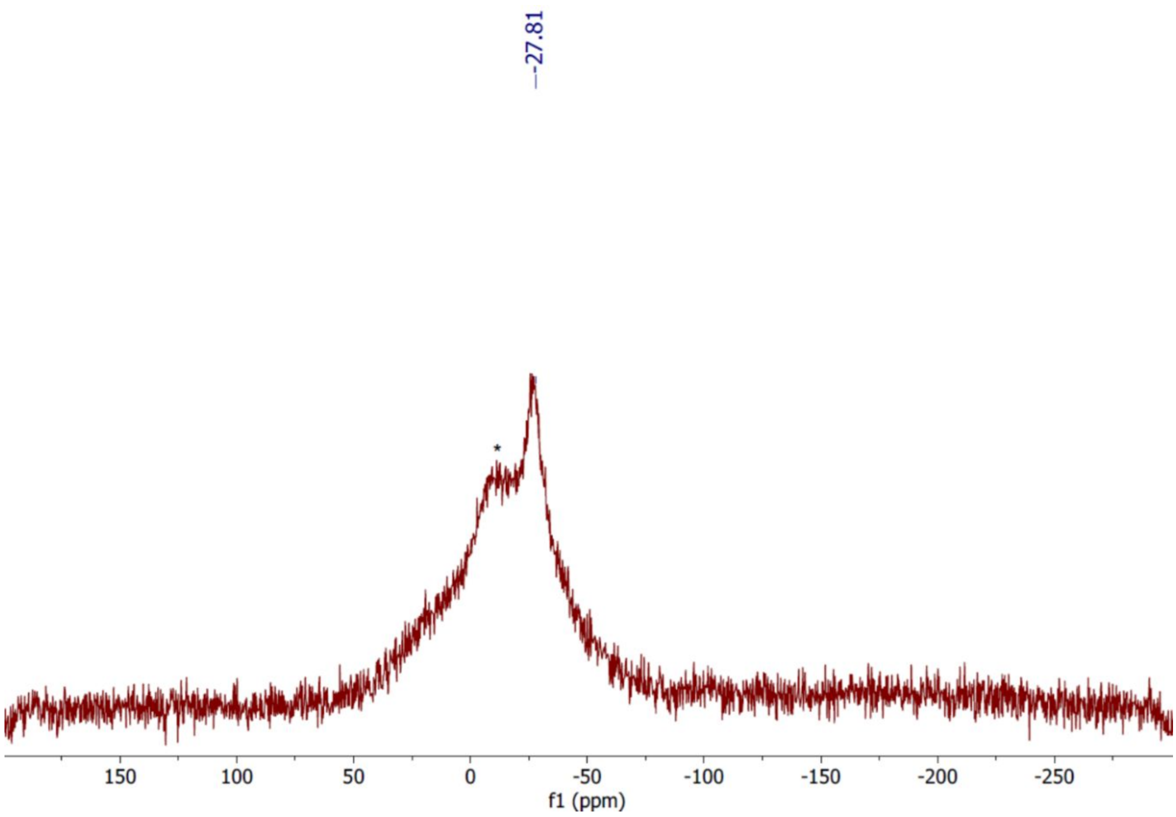


**Figure S 41.** $^{11}B\{^{1}H\}$ NMR spectrum of [Dy(Tp)$_2$($\mu$-H)$_2$Al(H)(N")] **1-Dy**, recorded in $d_6$-benzene. Borosilicate glass is denoted with *.

### B1.8 [Yb(Tp)$_2$($\mu$-H)$_2$Al(H)(N")] 2-Yb

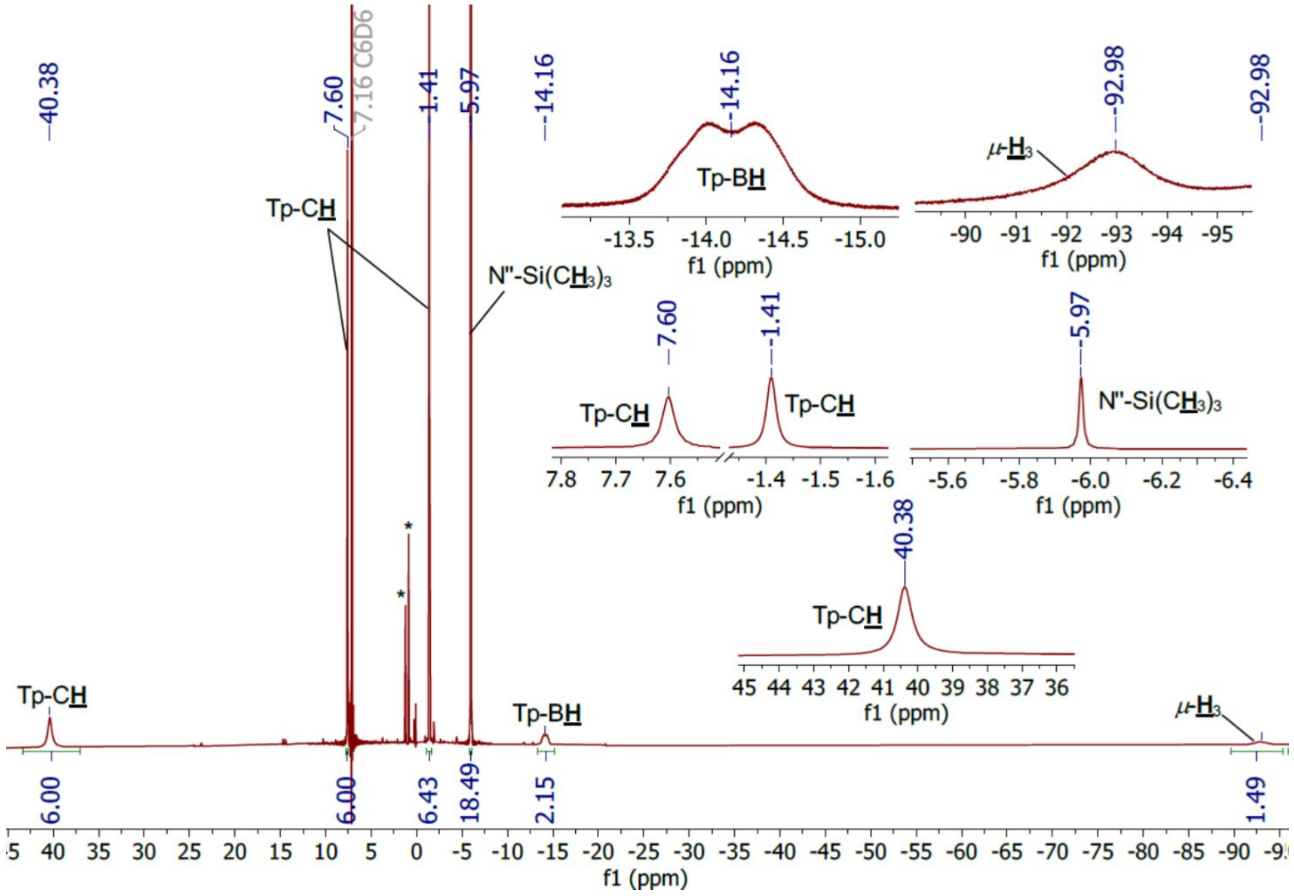


**Figure S 42.** $^{1}H$ NMR spectrum of [Yb(Tp)$_2$($\mu$-H)$_2$Al(H)(N")] **1-Yb**, recorded in $d_6$-benzene. Residual hexane is denoted with *.

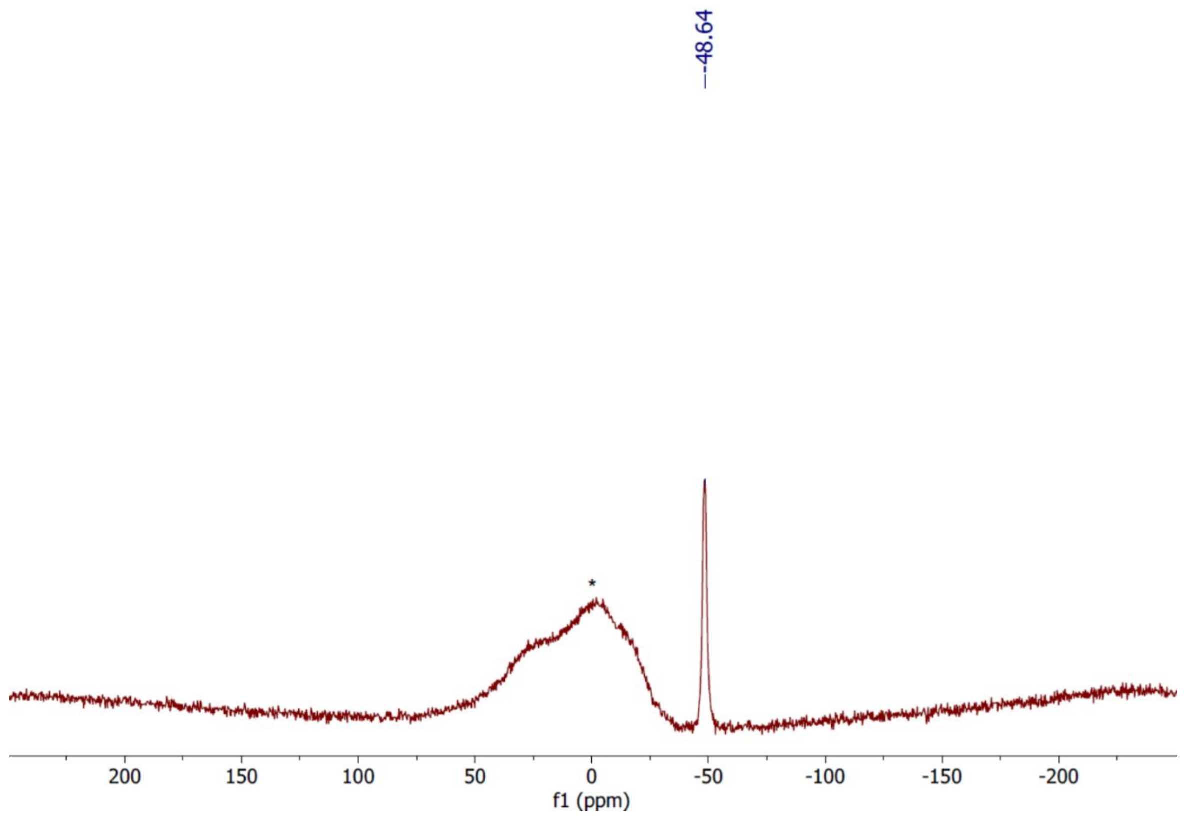


**Figure S 43.** $^{11}B$ NMR spectrum of $[Yb(Tp)_2(\mu\text{-}H)_2Al(H)(N'')]$ **1-Yb**, recorded in $d_6$-benzene. Borosilicate glass is denoted with *.

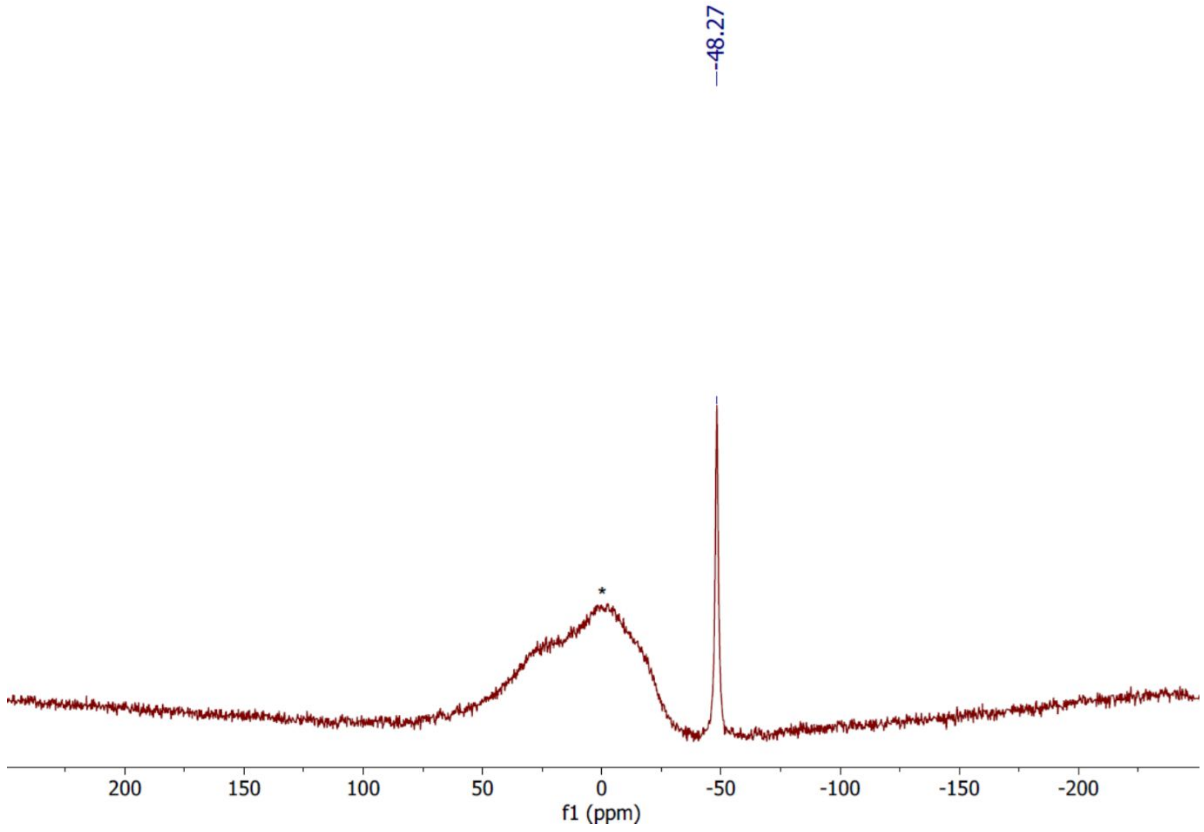


**Figure S 44.** $^{11}B\{^1H\}$ NMR spectrum of $[Yb(Tp)_2(\mu\text{-}H)_2Al(H)(N'')]$ **1-Yb**, recorded in $d_6$-benzene. Borosilicate glass is denoted with *.

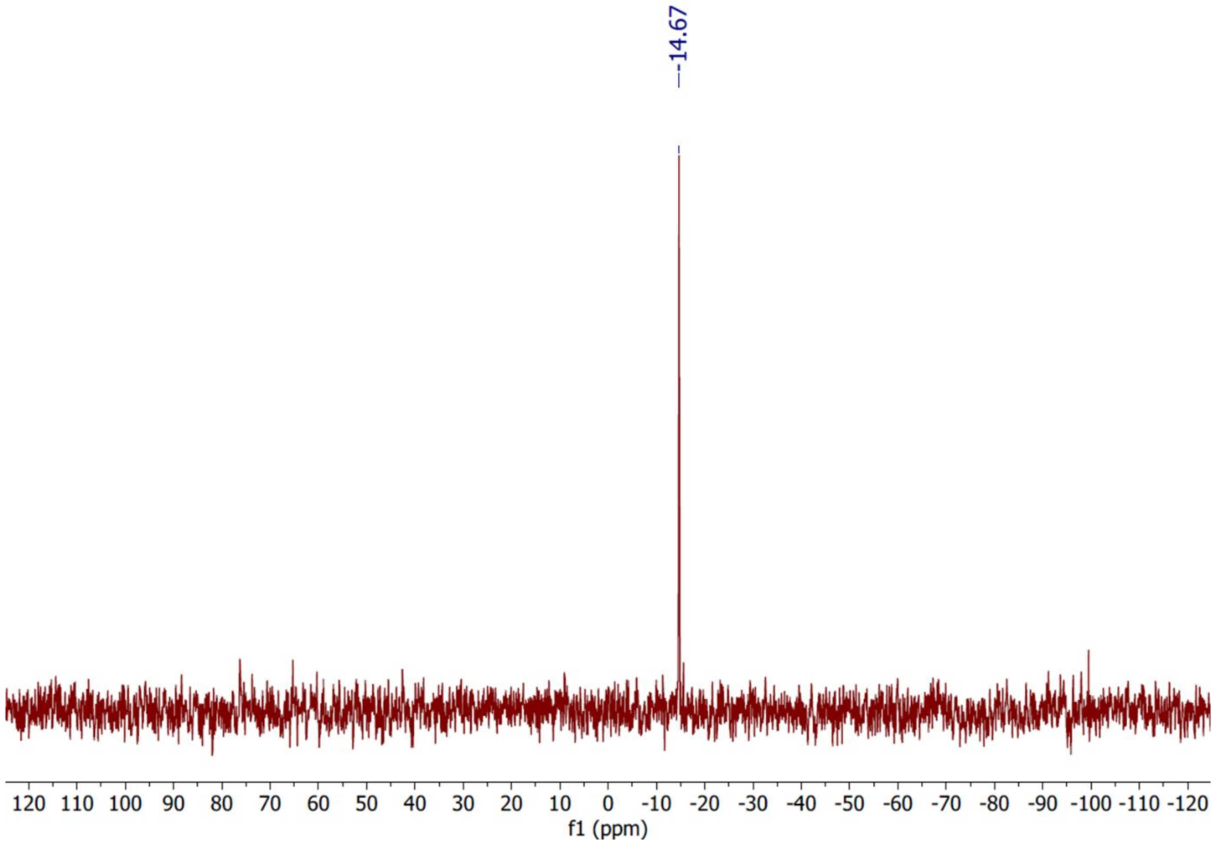


**Figure S 45.** $^{29}Si\{^{1}H\}$ INEPT NMR spectrum of $[Yb(Tp)_2(\mu\text{-}H)_2Al(H)(N'')]$ **1-Yb**, recorded in $d_6$-benzene.

### B1.9 [(IDipp)Al(N")(H)₂] Al-IDipp

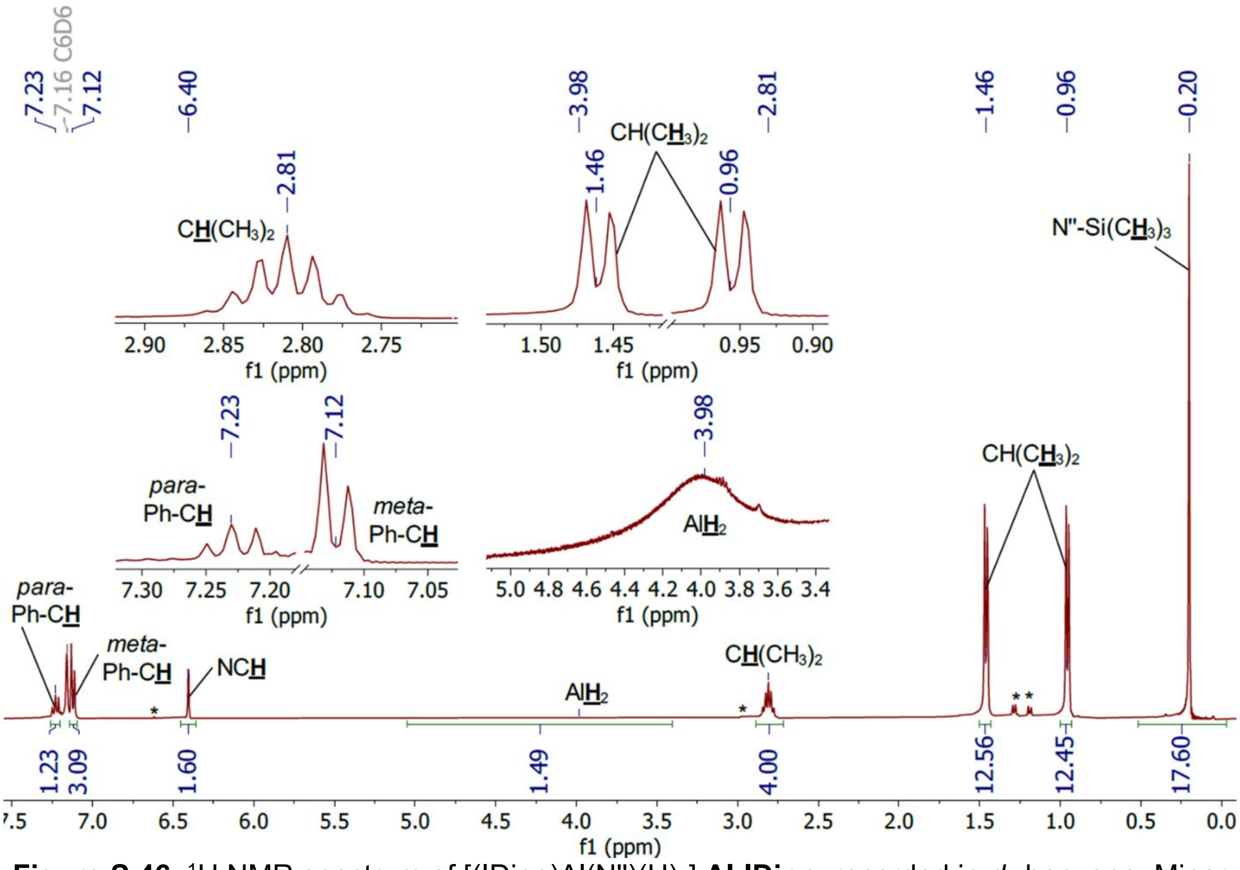


**Figure S 46.** $^{1}H$ NMR spectrum of $[(IDipp)Al(N'')(H)_2]$ **Al-IDipp**, recorded in $d_6$-benzene. Minor amount of free IDipp is denoted with *.

### B1.10 [Dy(Tp)$_2${$\kappa^2$-(Cy)NCHN(Cy)}] 3-Dy

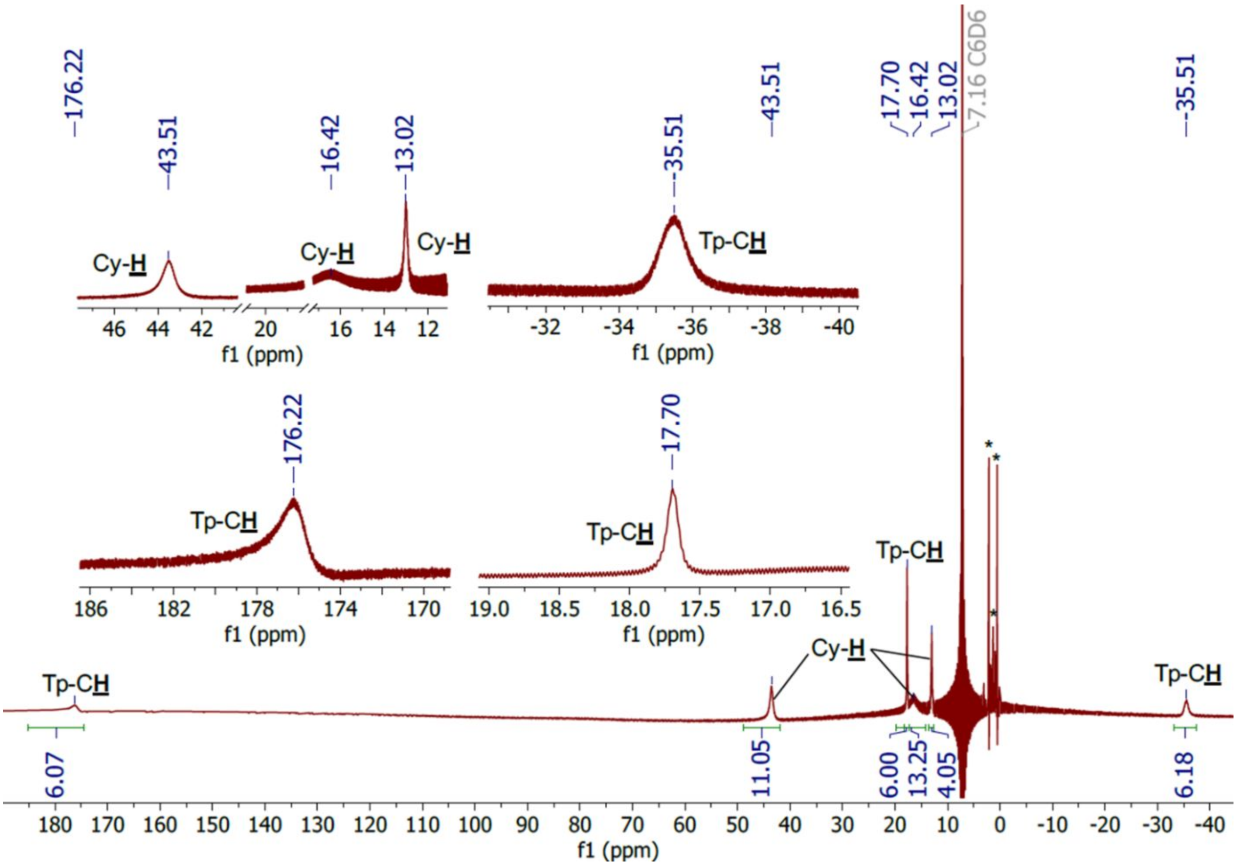


**Figure S 47.** $^1$H NMR spectrum of [Dy(Tp)$_2${$\kappa^2$-(Cy)NCHN(Cy)}] **3-Dy**, recorded in $d_6$-benzene. Minor byproduct [Al{$\kappa^2$-(Cy)NCHN(Cy)}$_2$(N'')] **3-Al** is denoted with *.

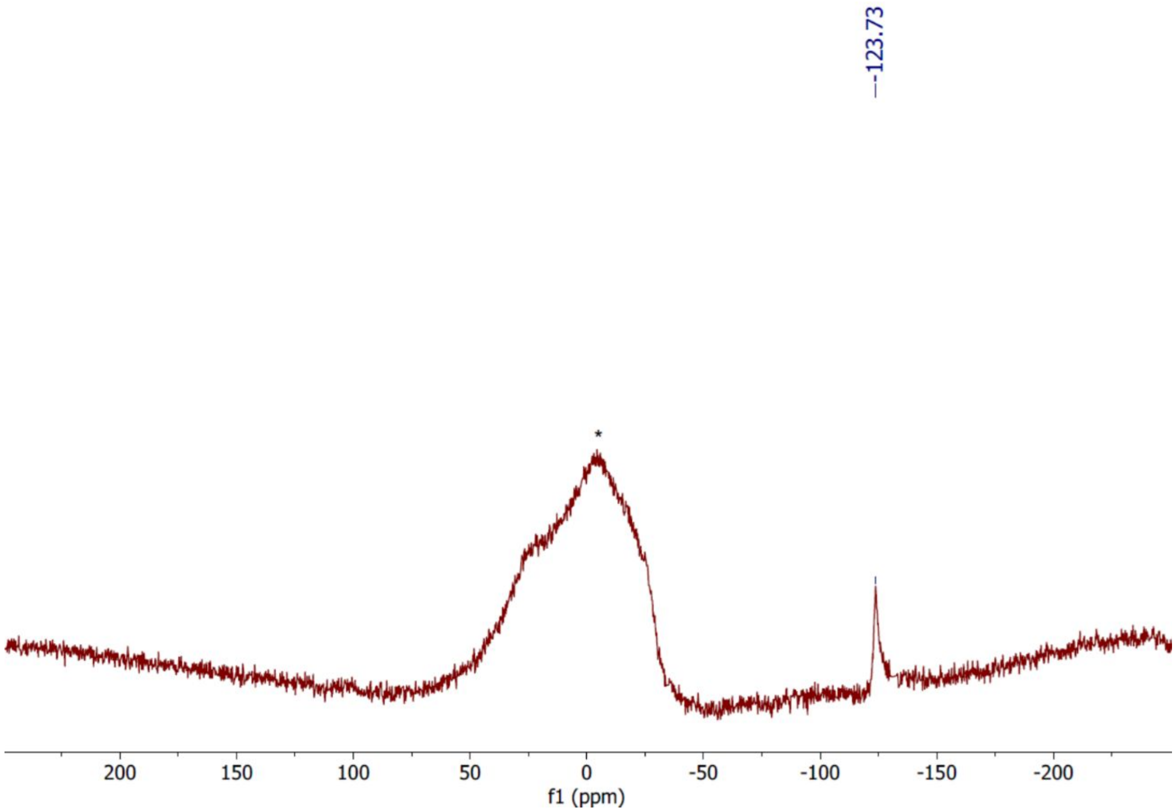


**Figure S 48.** $^{11}$B NMR spectrum of [Dy(Tp)$_2${$\kappa^2$-(Cy)NCHN(Cy)}] **3-Dy**, recorded in $d_6$-benzene. Borosilicate glass is denoted with *.

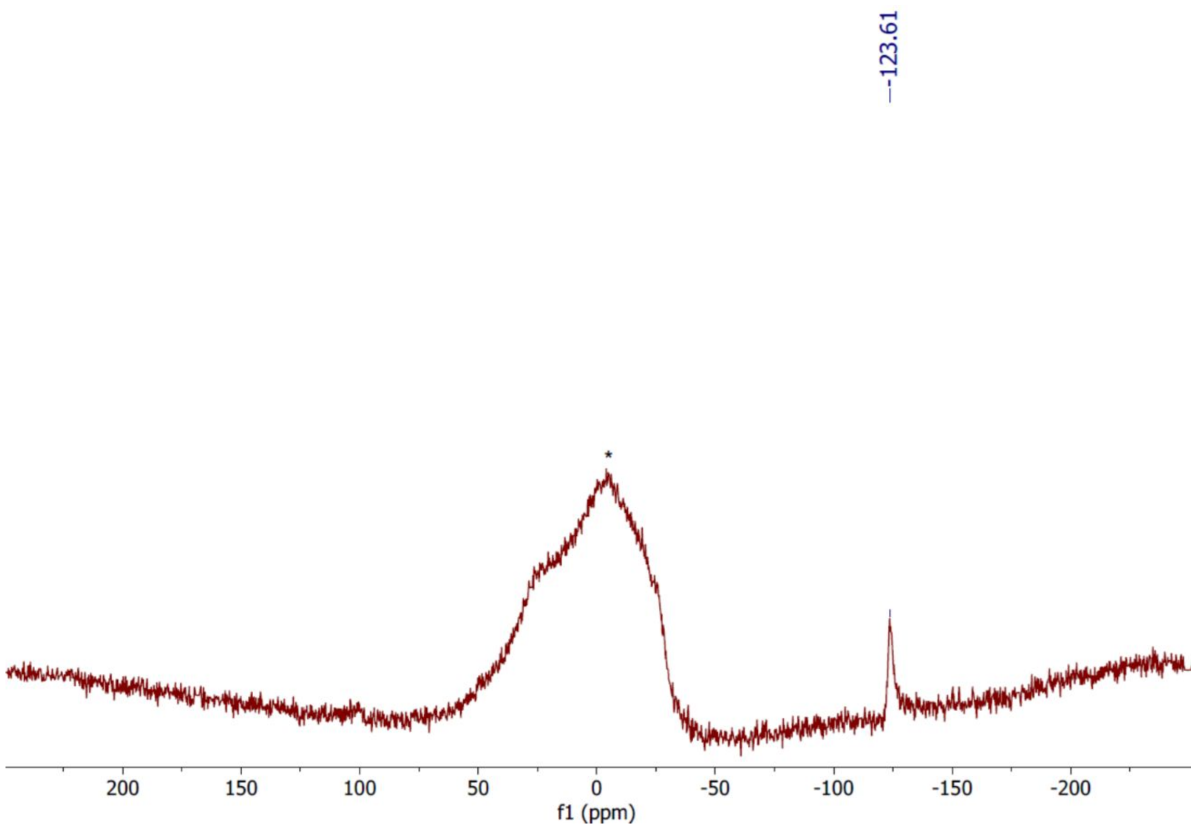


**Figure S 49.** $^{11}B\{^{1}H\}$ NMR spectrum of [Dy(Tp)$_2${$\kappa^2$-(Cy)NCHN(Cy)}] **3-Dy**, recorded in $d_6$-benzene. Borosilicate glass is denoted with *.

## B1.11 [Yb(Tp)$_2${$\kappa^2$-(Cy)NCHN(Cy)}] 3-Yb

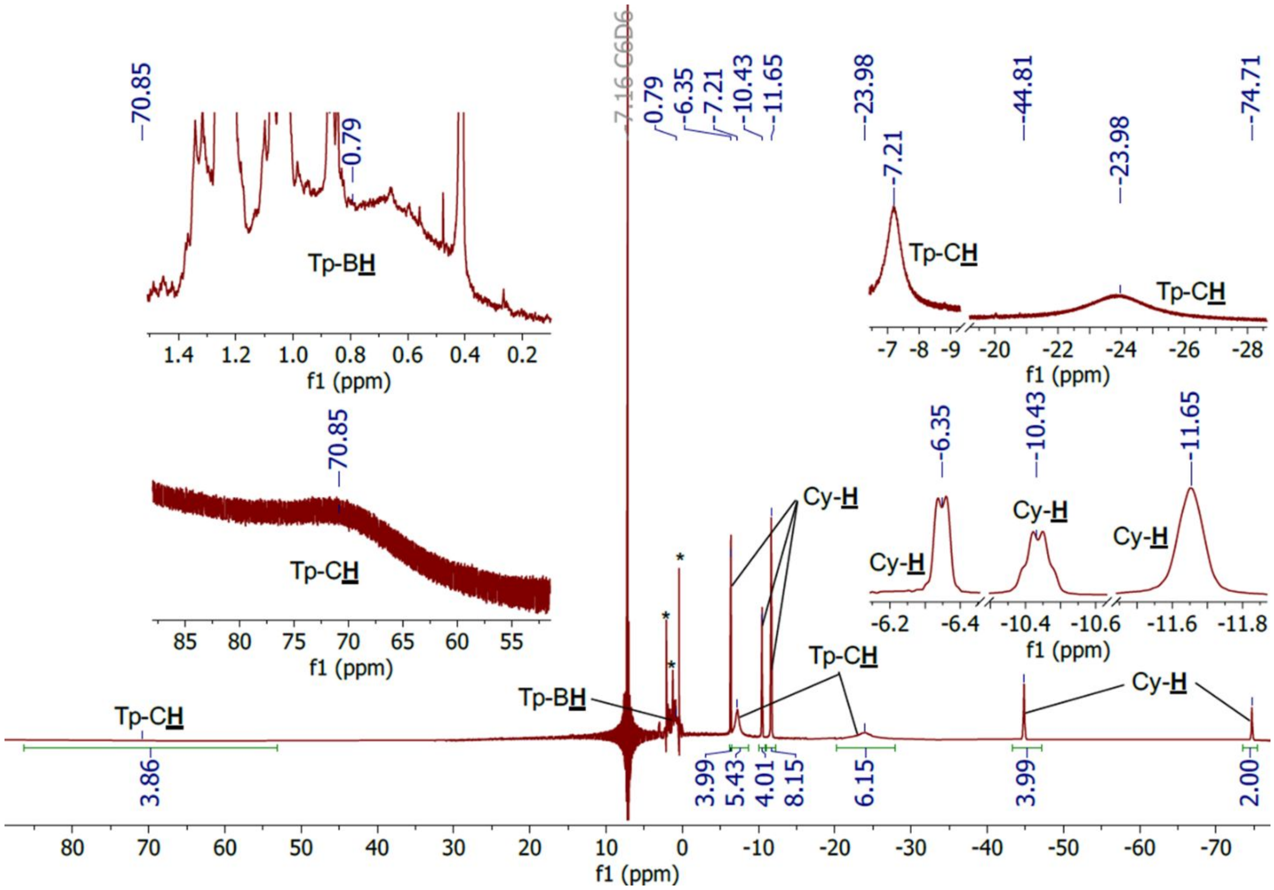


**Figure S 50.** $^{1}H$ NMR spectrum of [Yb(Tp)$_2${$\kappa^2$-(Cy)NCHN(Cy)}] **3-Yb**, recorded in $d_6$-benzene. Minor byproduct [Al{$\kappa^2$-(Cy)NCHN(Cy)}$_2$(N'')] **3-Al** is denoted with *.

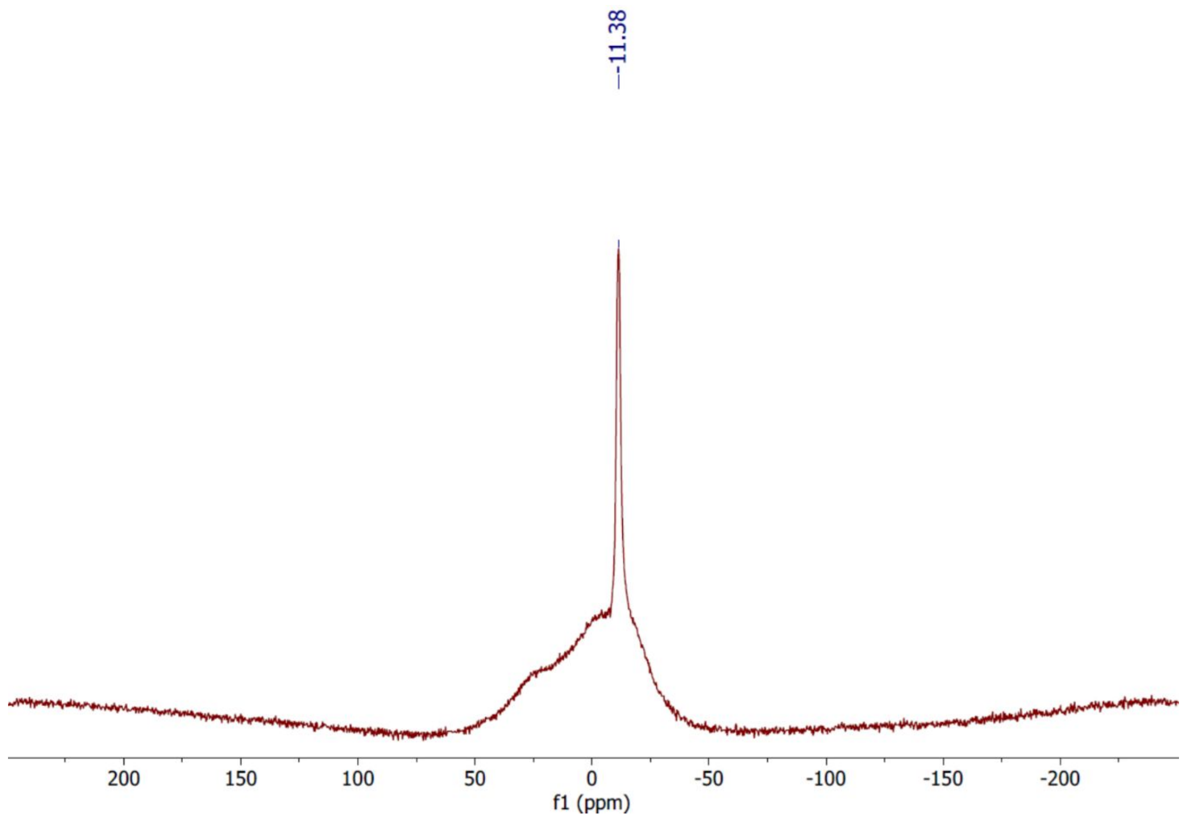


**Figure S 51.** $^{11}B$ NMR spectrum of $[Yb(Tp)_2\{\kappa^2\text{-}(Cy)NCHN(Cy)\}]$ **3-Yb**, recorded in $d_6$-benzene.

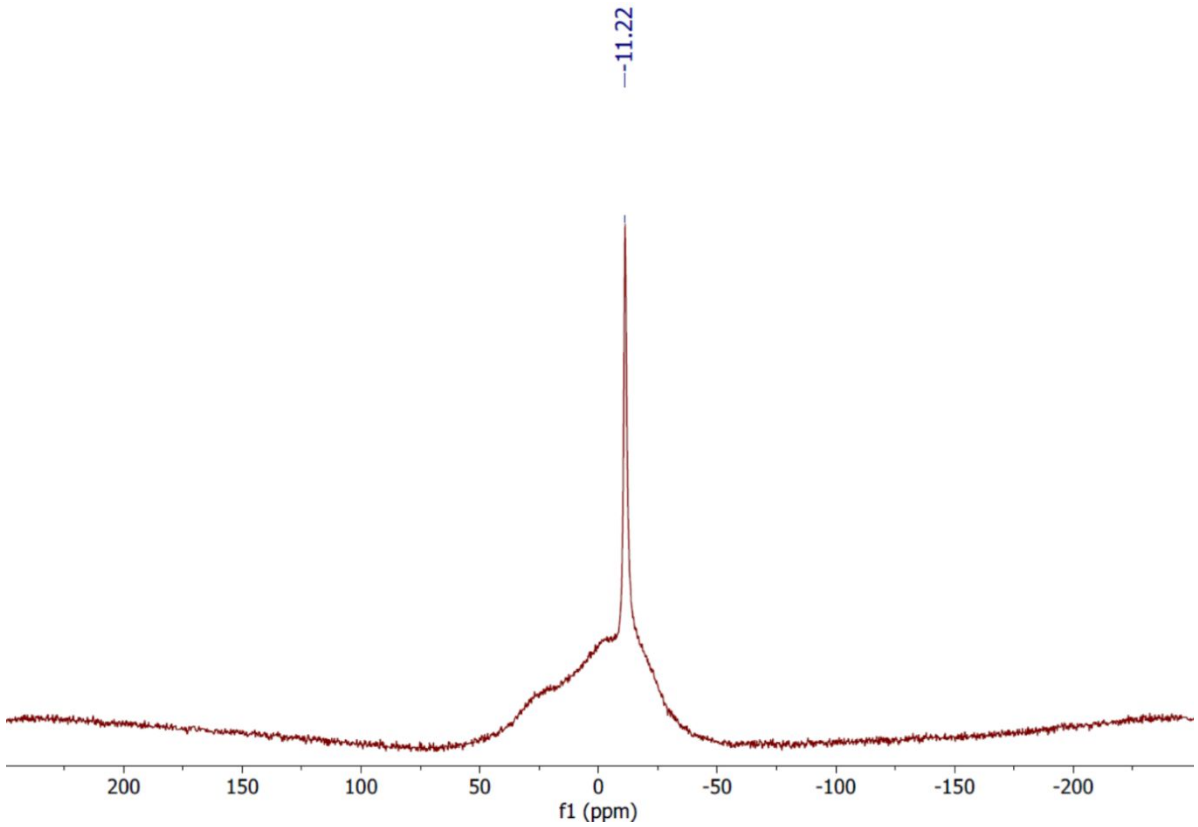


**Figure S 52.** $^{11}B\{^1H\}$ NMR spectrum of $[Yb(Tp)_2\{\kappa^2\text{-}(Cy)NCHN(Cy)\}]$ **3-Yb**, recorded in $d_6$-benzene.

## B2 NMR data for NMR-scale reactions

### B2.1 Heating [Y(Tp)$_2$($\mu$-H)$_2$Al(H)(N'')] 2-Y under dynamic vacuum at 80 °C

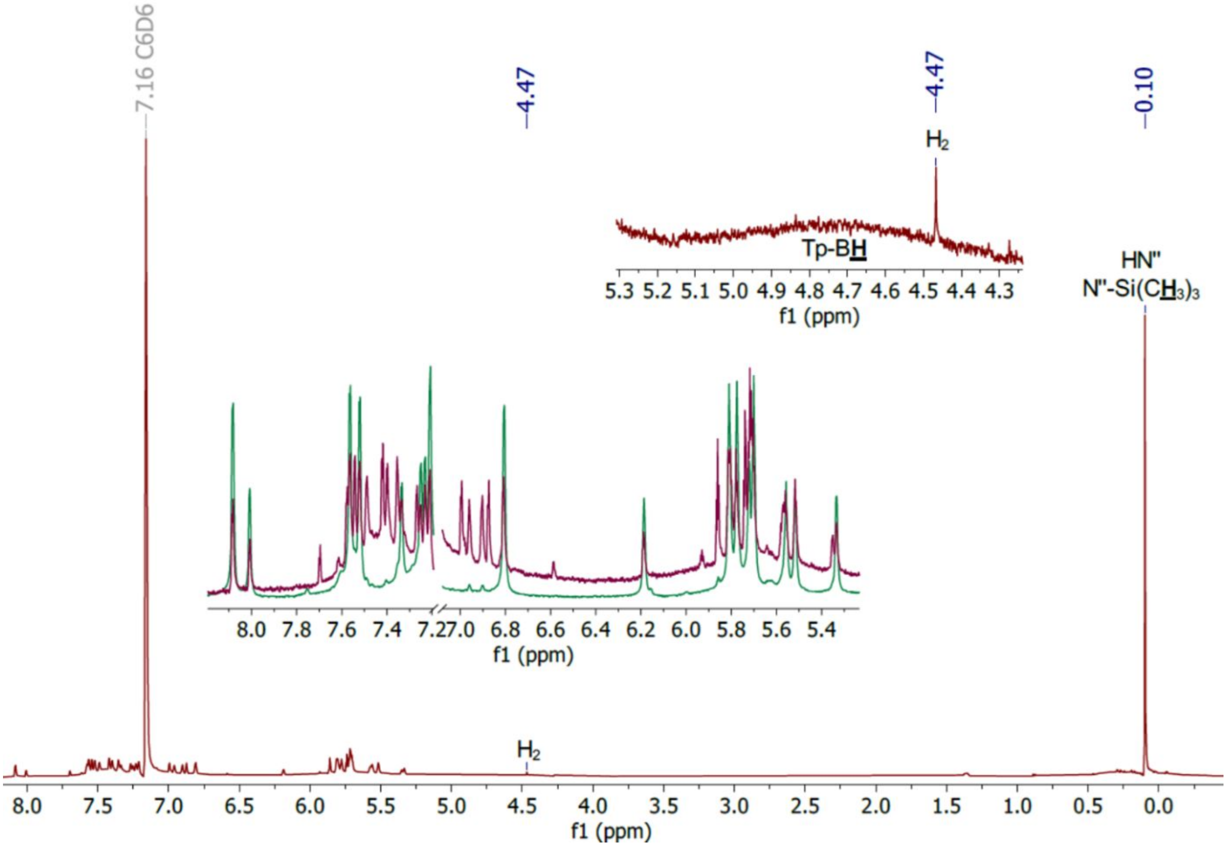


**Figure S 53.** $^{1}$H NMR spectrum in $d_6$-benzene, of the products obtained upon heating [Y(Tp)$_2$($\mu$-H)$_2$Al(H)(N'')] **2-Y** under dynamic vacuum at 80 °C, consistent with decomposition of **2-Y** resulting a complicated reaction mixture containing [Y(Tp)$_3$] (see overlay with green trace),[2] HN'', and $H_2$.

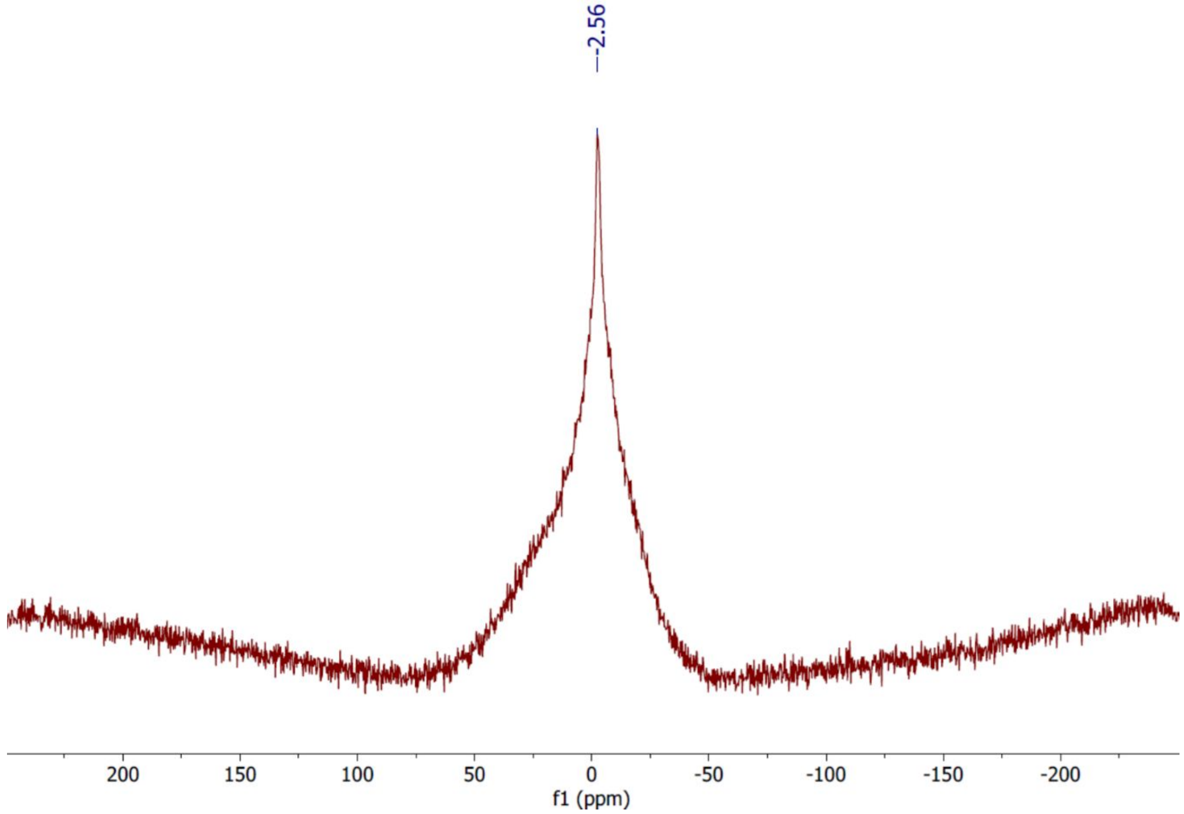


**Figure S 54.** $^{11}$B NMR spectrum in $d_6$-benzene, of the products obtained upon heating [Y(Tp)$_2$($\mu$-H)$_2$Al(H)(N'')] **2-Y** under dynamic vacuum at 80 °C consistent with decomposition of **2-Y**.

## B2.2 NMR-scale reaction between [Yb(Tp)$_2$($\mu$-H)$_2$Al(H)(N")] 2-Yb with IDipp

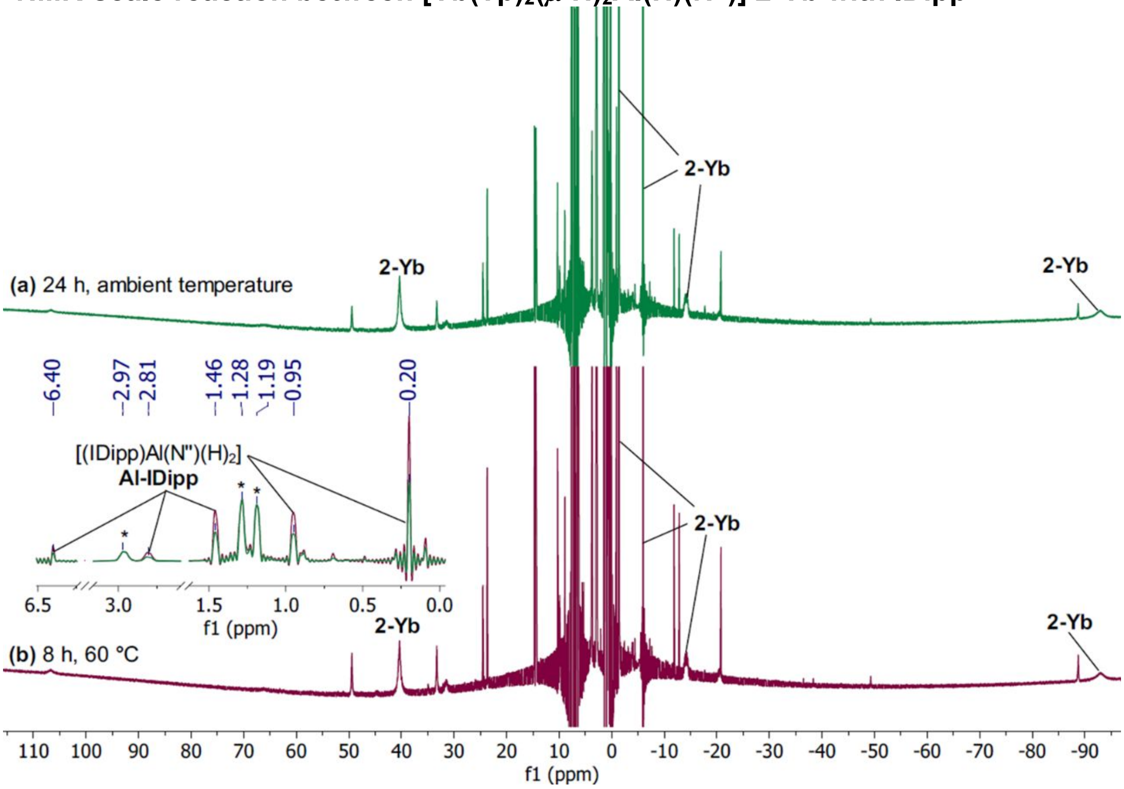


**Figure S 55.** $^{1}$H NMR spectra of the NMR-scale reaction between [Yb(Tp)$_2$($\mu$-H)$_2$Al(H)(N")] **2-Yb** with IDipp in $d_6$-benzene at t = 24 h at ambient temperature (a) and subsequent t = 8 h with heating at 60 °C (b), consistent with incomplete consumption of **2-Yb** and IDipp (denoted with *) and slow formation of [(IDipp)Al(N")(H)$_2$] **Al-IDipp** and [Yb(Tp)$_3$].[6]

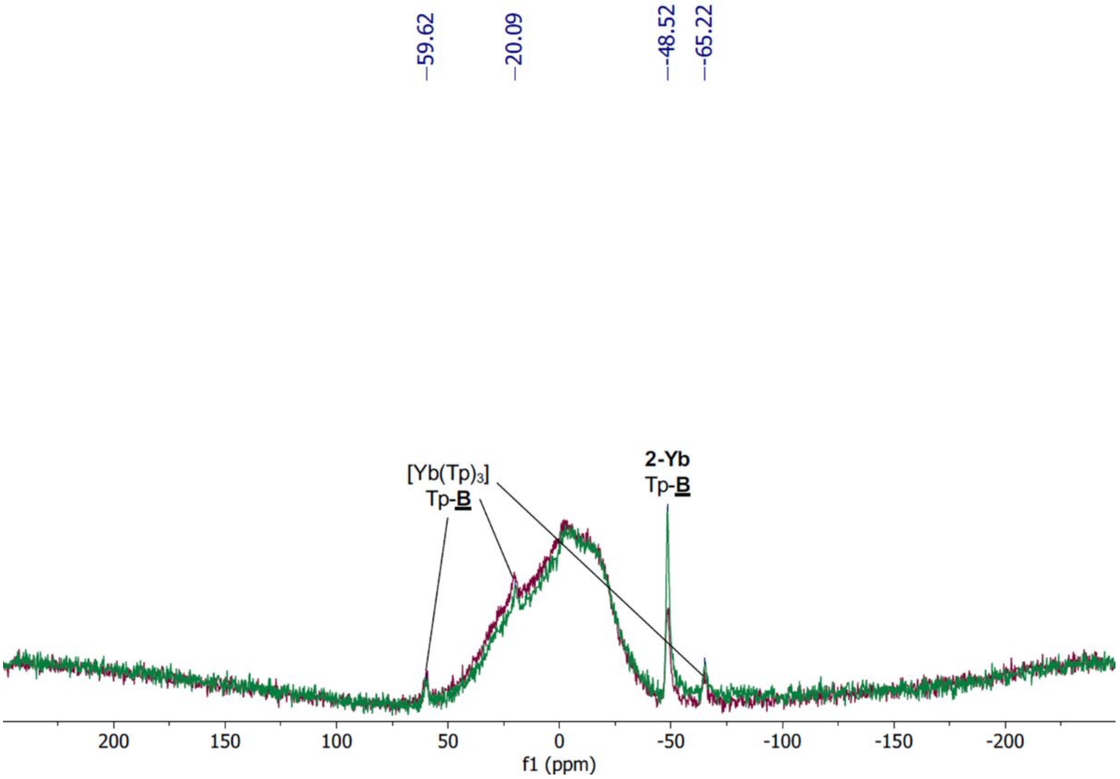


**Figure S 56.** $^{11}$B NMR spectra of the NMR-scale reaction between [Yb(Tp)$_2$($\mu$-H)$_2$Al(H)(N")] **2-Yb** with IDipp in $d_6$-benzene at t = 24 h at ambient temperature (green trace) and subsequent t = 8 h after heating at 60 °C (maroon trace), consistent with slow consumption of **2-Yb** and formation of [Yb(Tp)$_3$].[6]

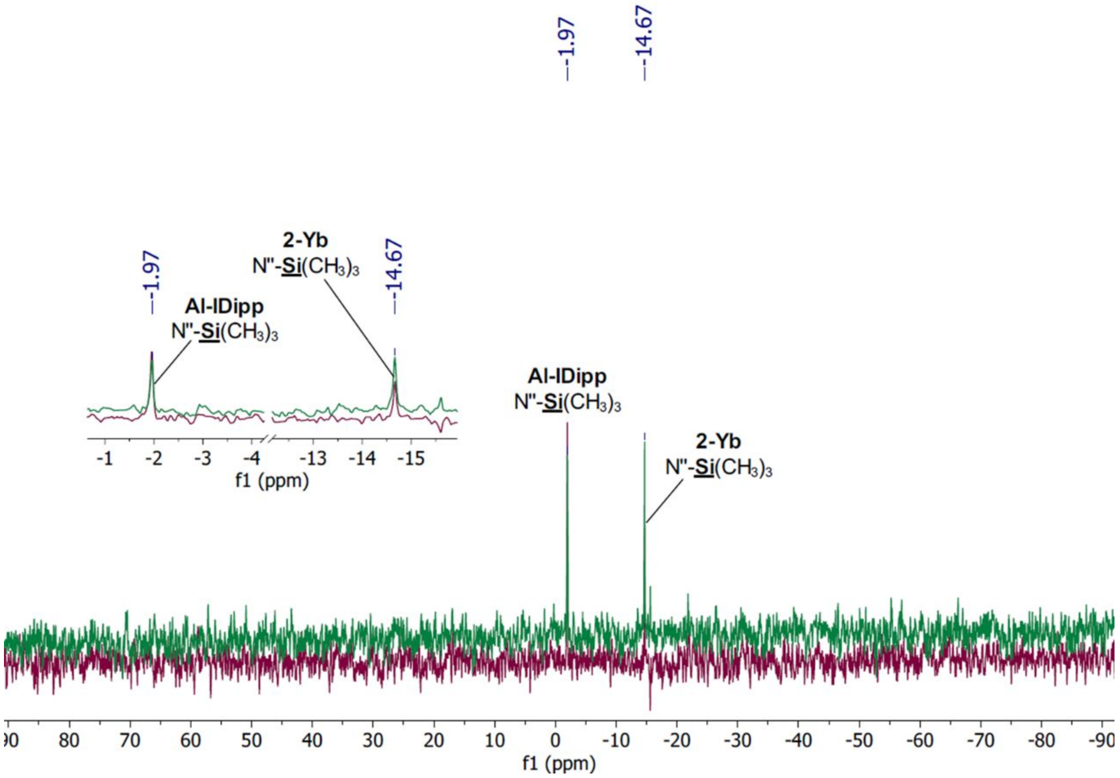


**Figure S 57.** $^{29}Si\{^{1}H\}$ INEPT NMR spectra of the NMR-scale reaction between $[Yb(Tp)_2(\mu\text{-}H)_2Al(H)(N'')]$ **2-Yb** with IDipp in $d_6$-benzene at t = 24 h at ambient temperature (a) and subsequent t = 8 h after heating at 60 °C (b), consistent with slow consumption of **2-Yb** and formation of $[(IDipp)Al(N'')(H)_2]$ **Al-IDipp**.

## B2.3 NMR-scale reaction between $[Me_3N•AlH_3]$ with K(N")

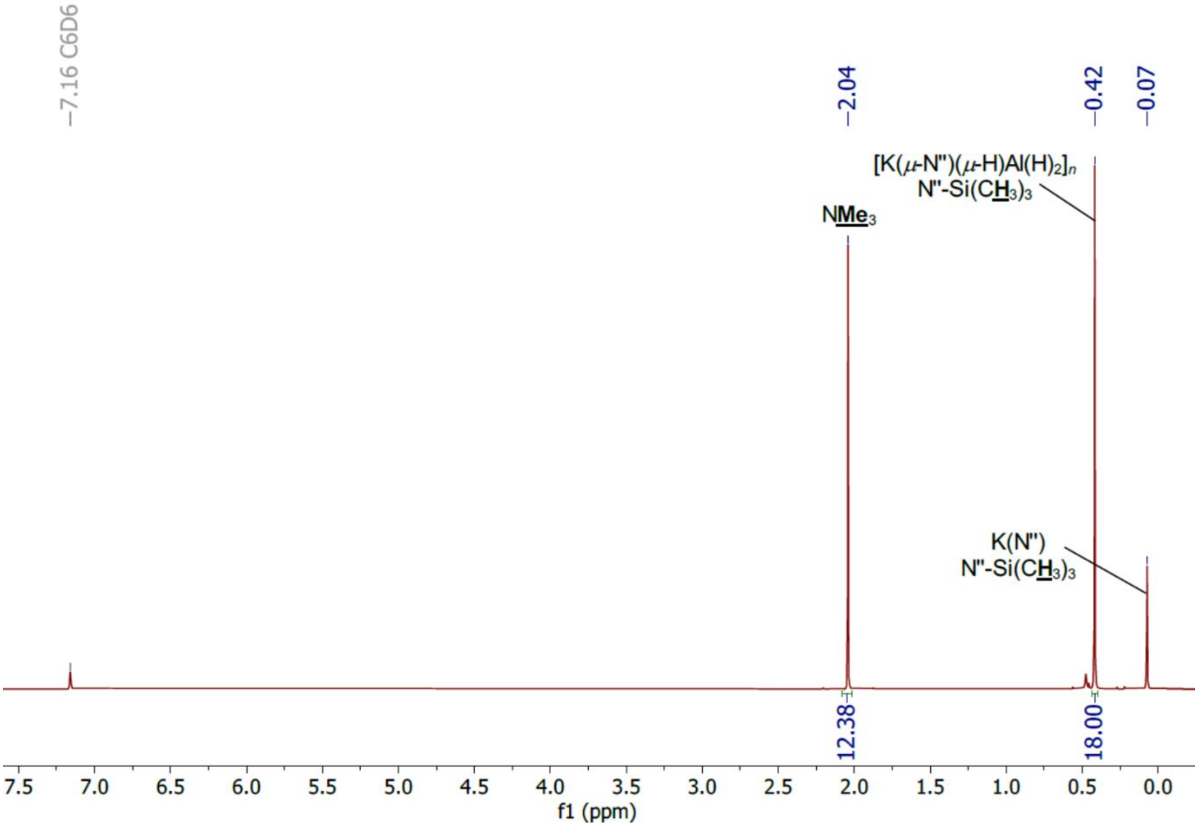


**Figure S 58.** $^{1}H$ NMR spectrum of the NMR-scale reaction between $[Me_3N•AlH_3]$ with K(N") in $d_6$-benzene, consistent with formation of partially soluble $[\{K(\mu\text{-}N'')Al(H)_3\}_n]$ and elimination of $NMe_3$ under ambient temperatures. Excess K(N") is also observed on the NMR scale.

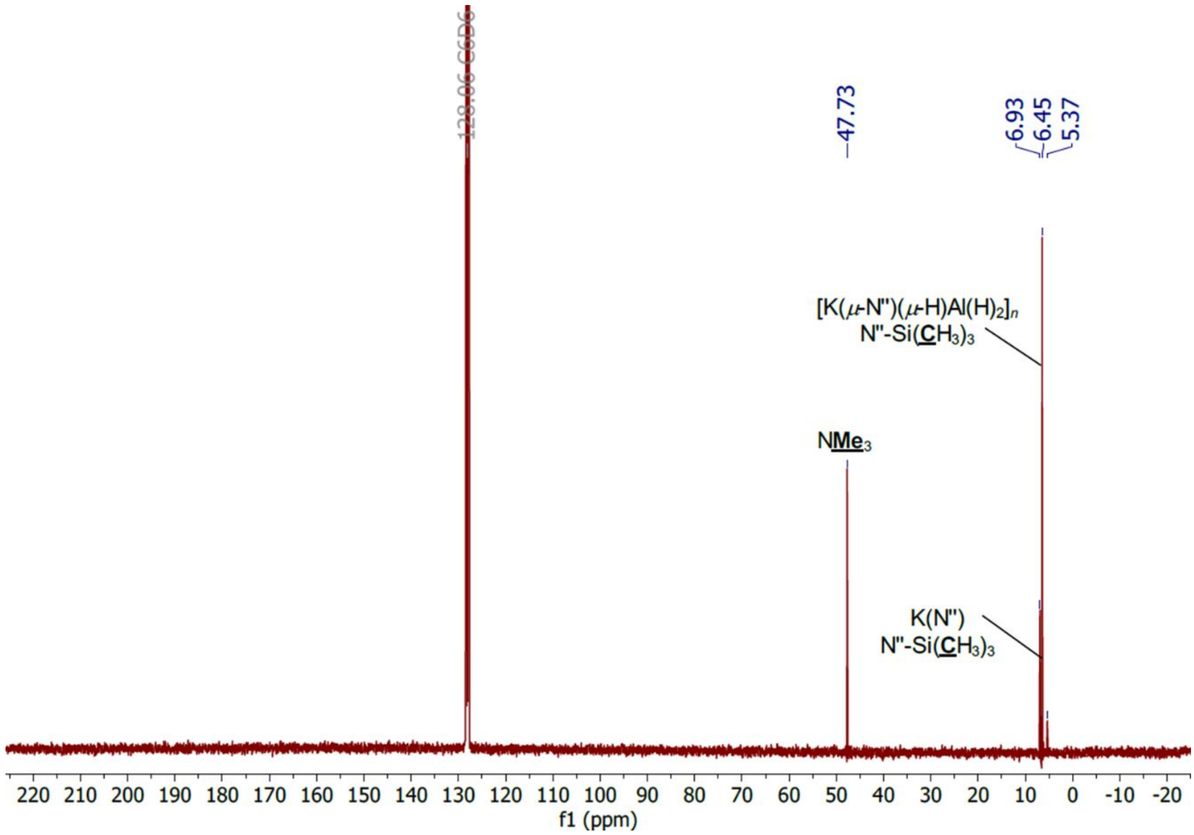


**Figure S 59.** $^{13}C\{^{1}H\}$ NMR spectrum of the NMR-scale reaction between [$Me_3N$•$AlH_3$] with K(N") in $d_6$-benzene, consistent with formation of partially soluble [{K($\mu$-N")Al(H)$_3$}$_n$] and elimination of $NMe_3$ under ambient temperatures. Excess K(N") is also observed on the NMR scale.

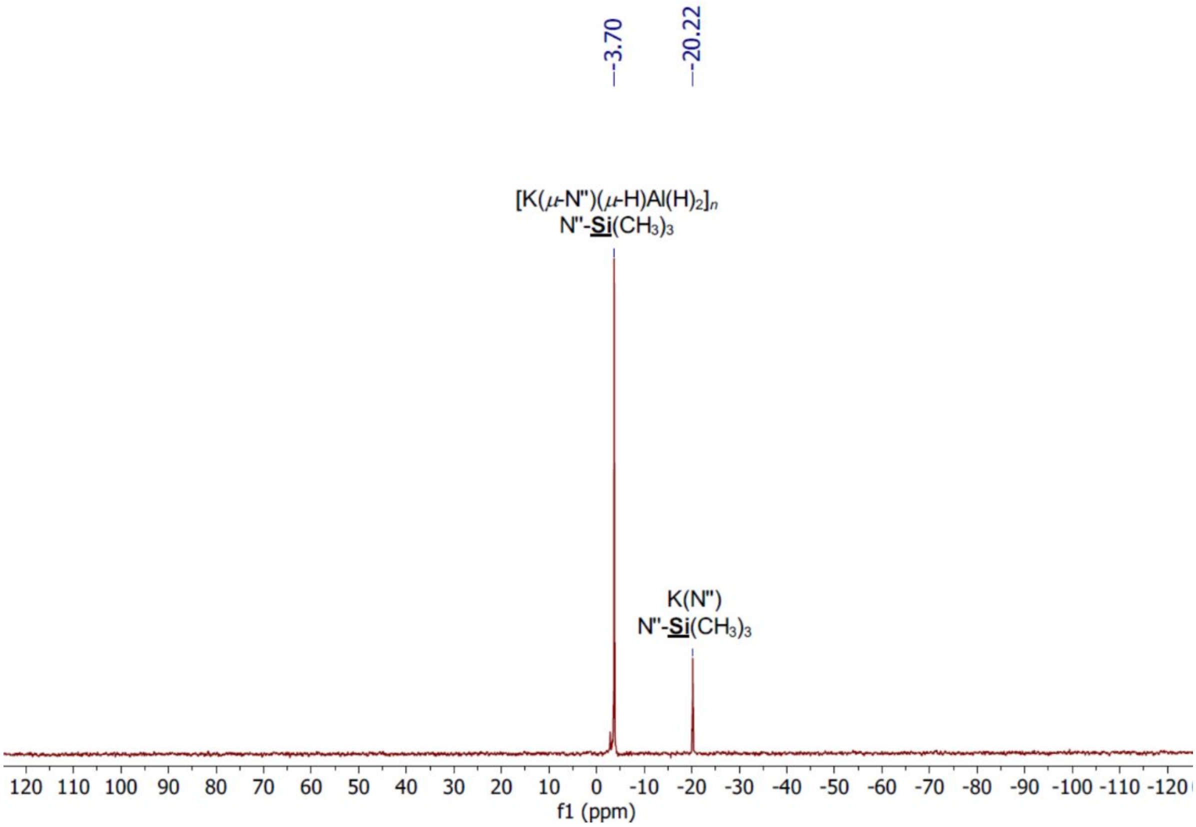


**Figure S 60.** $^{29}Si\{^{1}H\}$ INEPT NMR spectrum of the NMR-scale reaction between [$Me_3N$•$AlH_3$] with K(N") in $d_6$-benzene, consistent with formation of partially soluble [{K($\mu$-N")Al(H)$_3$}$_n$] under ambient temperatures. Excess K(N") is also observed on the NMR scale.

### B2.4 NMR-scale reaction between [Y(Tp)$_2$($\mu$-H)$_2$Al(H)(N")] 2-Y with K(N")

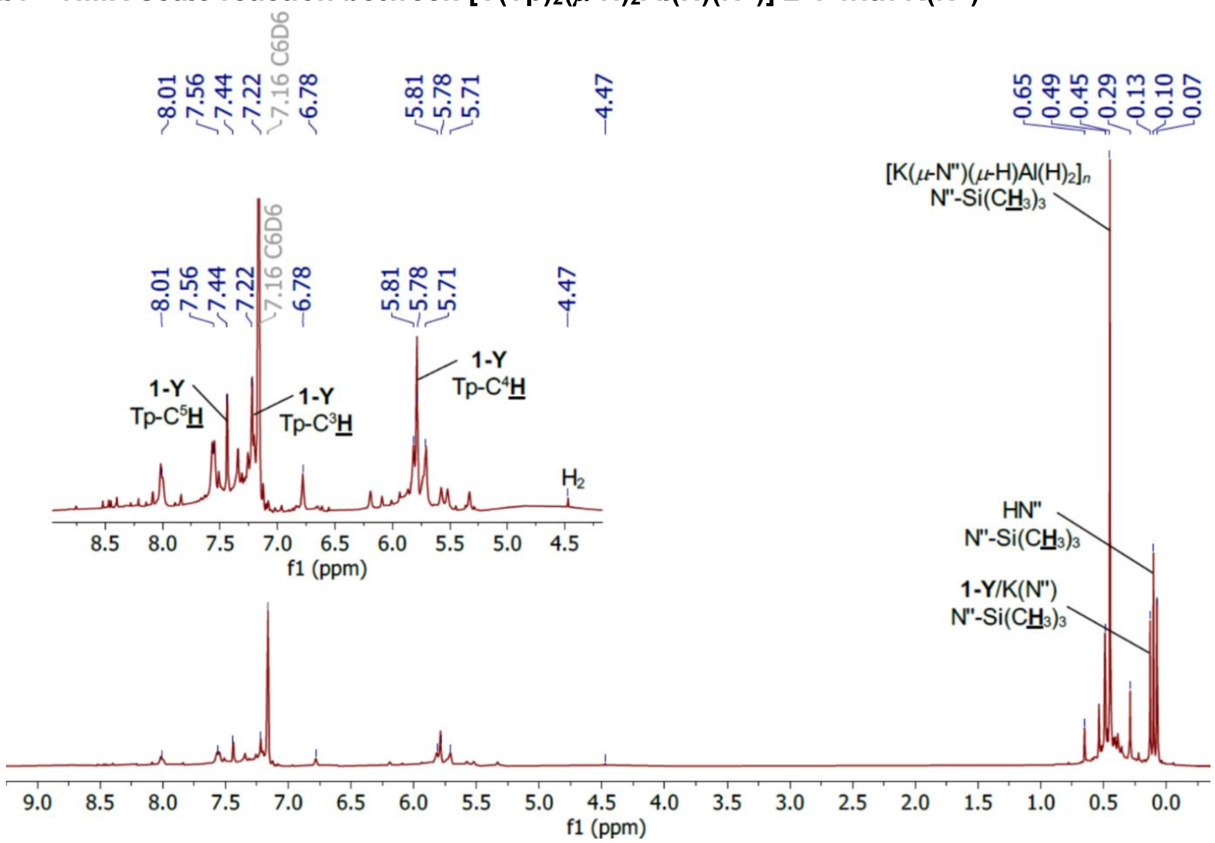


**Figure S 61.** $^1$H NMR spectrum of the NMR-scale reaction between [Y(Tp)$_2$($\mu$-H)$_2$Al(H)(N")] **2-Y** with K(N") in $d_6$-benzene, consistent with consumption of **2-Y** and formation of [K($\mu$-N")($\mu$-H)Al(H)$_2$]$_n$, and a complicated reaction mixture containing **1-Y**, [Y(Tp)$_3$],[2] K(N"), HN", and $H_2$ under ambient temperatures.

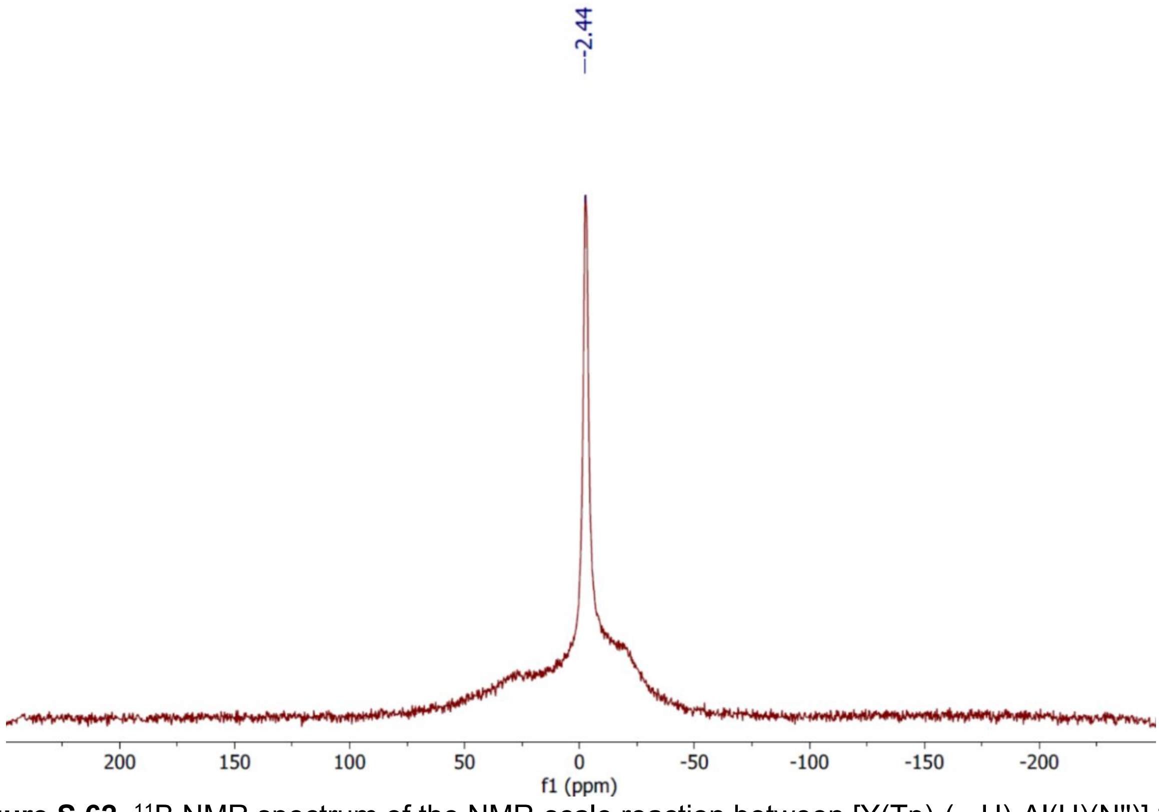


**Figure S 62.** $^{11}$B NMR spectrum of the NMR-scale reaction between [Y(Tp)$_2$($\mu$-H)$_2$Al(H)(N")] **2-Y** with K(N") in $d_6$-benzene, consistent with consumption of **2-Y** and formation of **1-Y** under ambient temperatures.

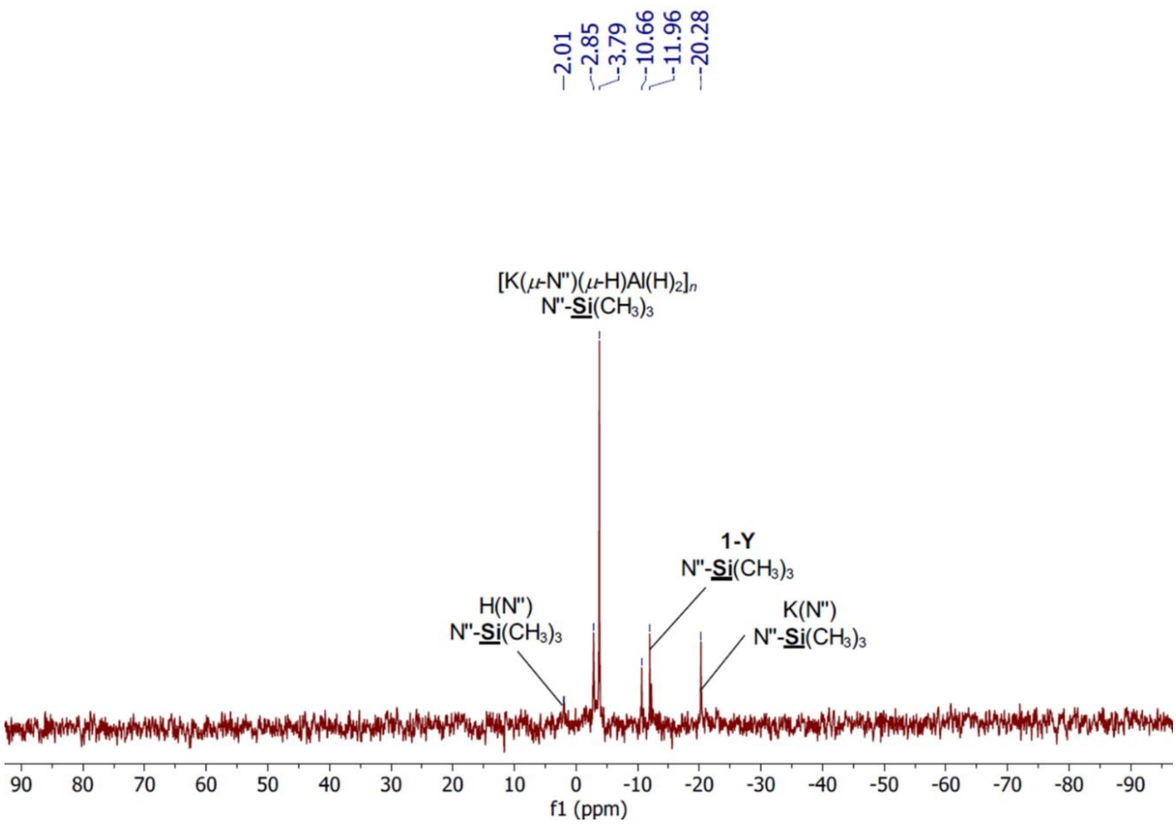


**Figure S 63.** $^{29}Si\{^1H\}$ INEPT NMR spectrum of the NMR-scale reaction between [Y(Tp)$_2$($\mu$-H)$_2$Al(H)(N")] **2-Y** with K(N") in $d_6$-benzene, consistent with consumption of **2-Y** and formation of [K($\mu$-N")($\mu$-H)Al(H)$_2$]$_n$, and a complicated reaction mixture containing **1-Y**, K(N"), and HN" under ambient temperatures.

### B2.5 NMR-scale reaction between [Sm(Tp)$_2$($\mu$-H)$_2$Al(H)(N")] 2-Sm with K(N")

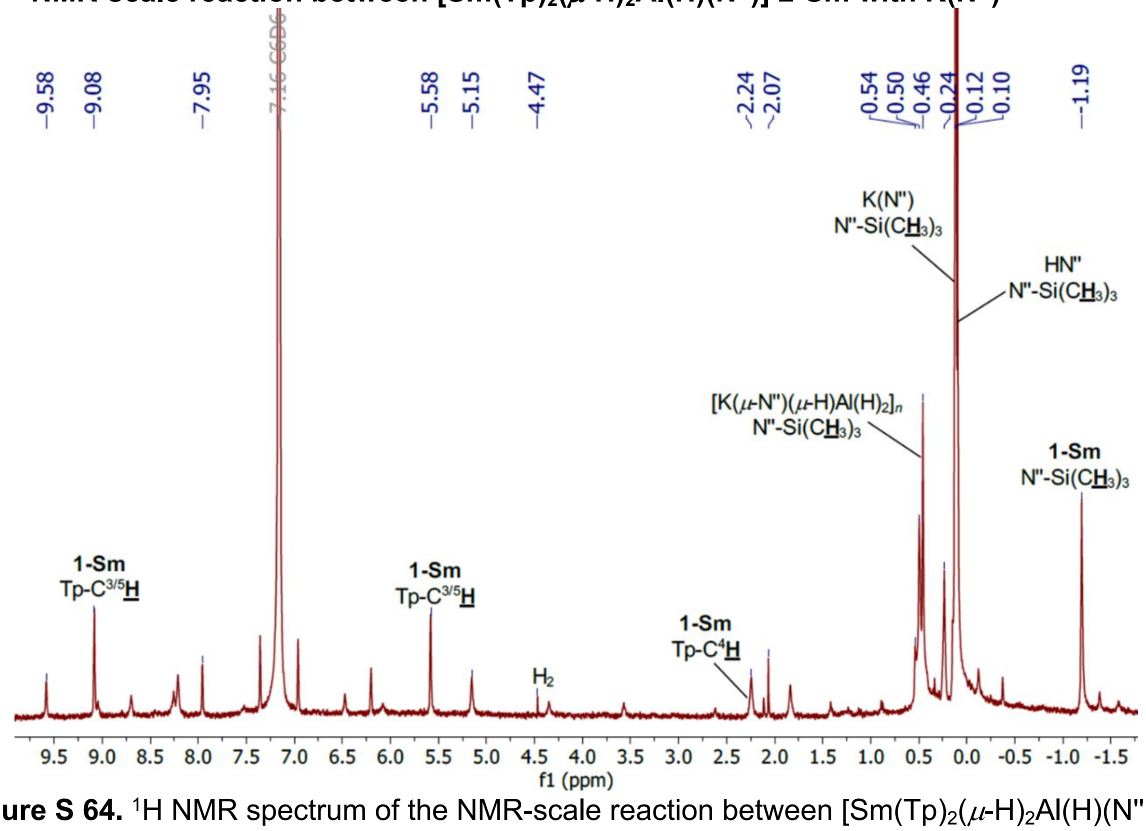


**Figure S 64.** $^1H$ NMR spectrum of the NMR-scale reaction between [Sm(Tp)$_2$($\mu$-H)$_2$Al(H)(N")] **2-Sm** with K(N") in $d_6$-benzene, consistent with consumption of **2-Sm** and formation of [K($\mu$-N")($\mu$-H)Al(H)$_2$]$_n$, and a complicated reaction mixture containing **1-Sm**, [Sm(Tp)$_3$],[7] K(N"), HN", and $H_2$ under ambient temperatures.

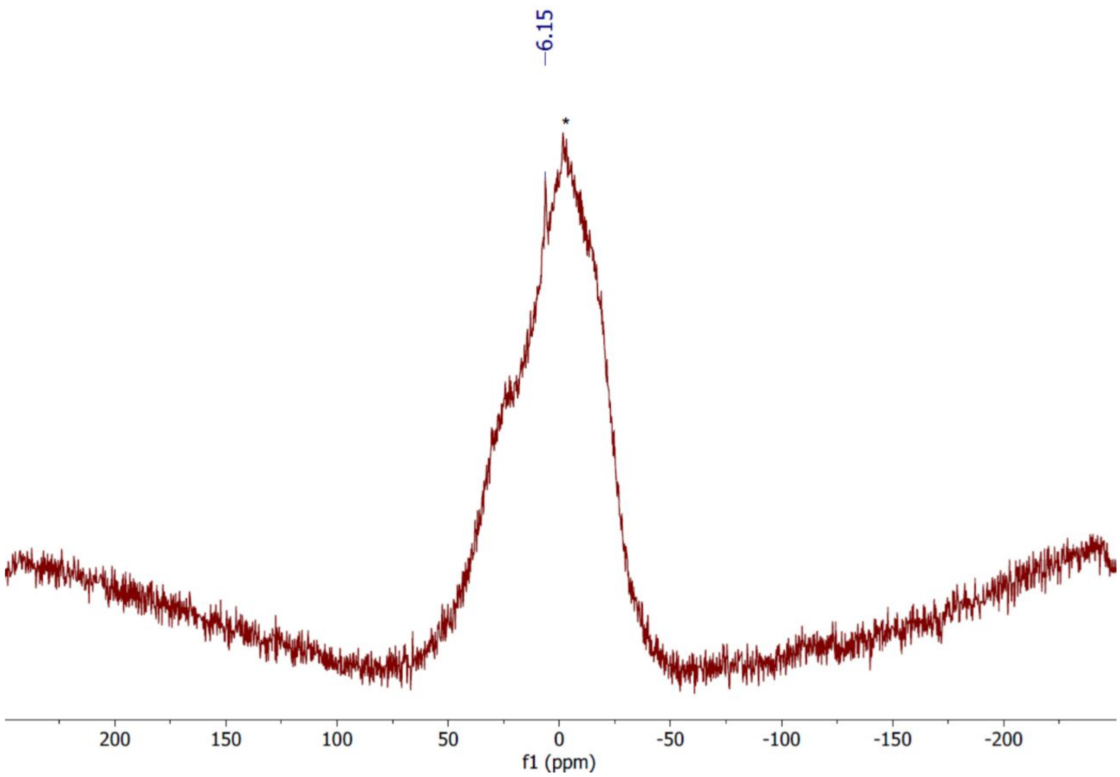


**Figure S 65.** $^{11}$B NMR spectrum of the NMR-scale reaction between $[Sm(Tp)_2(\mu\text{-}H)_2Al(H)(N'')]$ **2-Sm** with K(N'') in $d_6$-benzene, consistent with consumption of **2-Sm** and formation of **1-Sm** under ambient temperatures. Borosilicate glass is denoted with *.

### B2.6 NMR-scale reaction between $[Dy(Tp)_2(\mu\text{-}H)_2Al(H)(N'')]$ 2-Dy with K(N'')

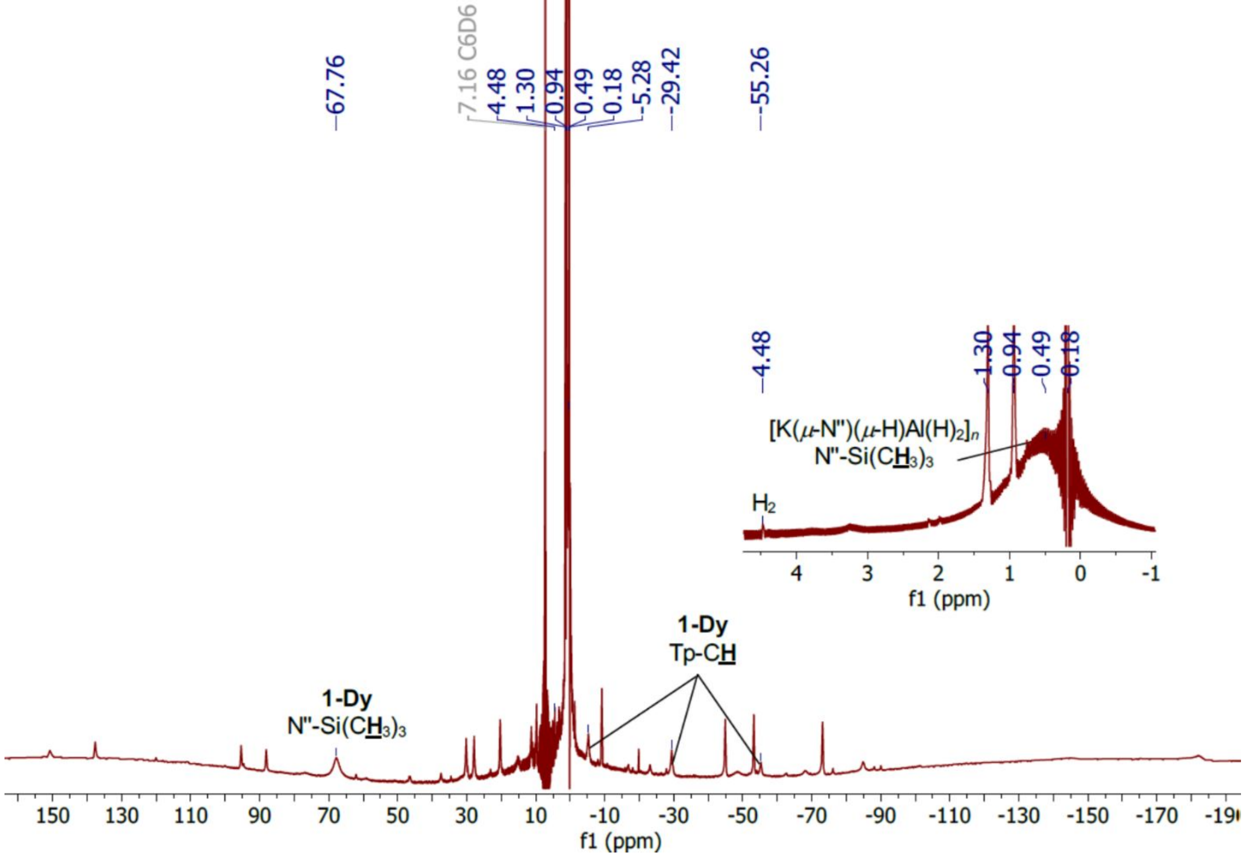


**Figure S 66.** $^{1}$H NMR spectrum of the NMR-scale reaction between $[Dy(Tp)_2(\mu\text{-}H)_2Al(H)(N'')]$ **2-Dy** with K(N'') in $d_6$-benzene, consistent with consumption of **2-Dy** and formation of $[K(\mu\text{-}N'')(\mu\text{-}H)Al(H)_2]_n$, and a complicated reaction mixture containing $[Dy(Tp)_3]$[7] and $H_2$ under ambient temperatures.

## B2.7 NMR-scale reaction between [Yb(Tp)$_2$($\mu$-H)$_2$Al(H)(N'')] 2-Yb with K(N'')

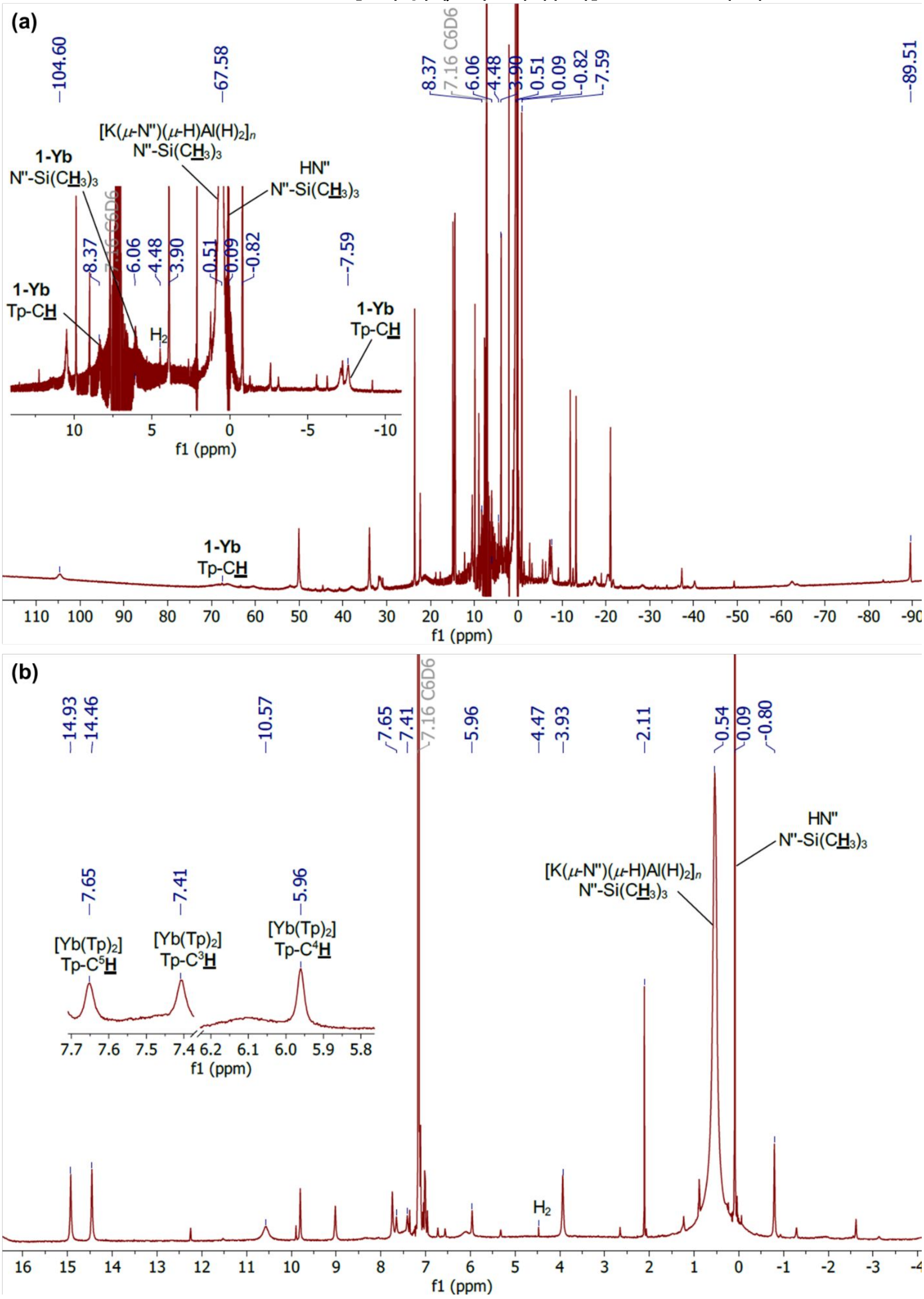


**Figure S 67.** $^1$H NMR spectra of the NMR-scale reaction between [Yb(Tp)$_2$($\mu$-H)$_2$Al(H)(N'')] **2-Yb** with K(N'') in $d_6$-benzene at t = 0.25 h (a) and t = 20 h (b), consistent with consumption of **2-Yb** and formation of [K($\mu$-N'')($\mu$-H)Al(H)$_2$]$_n$, **1-Yb**, [Yb(Tp)$_2$],[5] [Yb(Tp)$_3$],[6] HN'', and $H_2$ (gradual formation of [Yb(Tp)$_2$] and consumption of **1-Yb** observed within t = 20 h) under ambient temperatures.

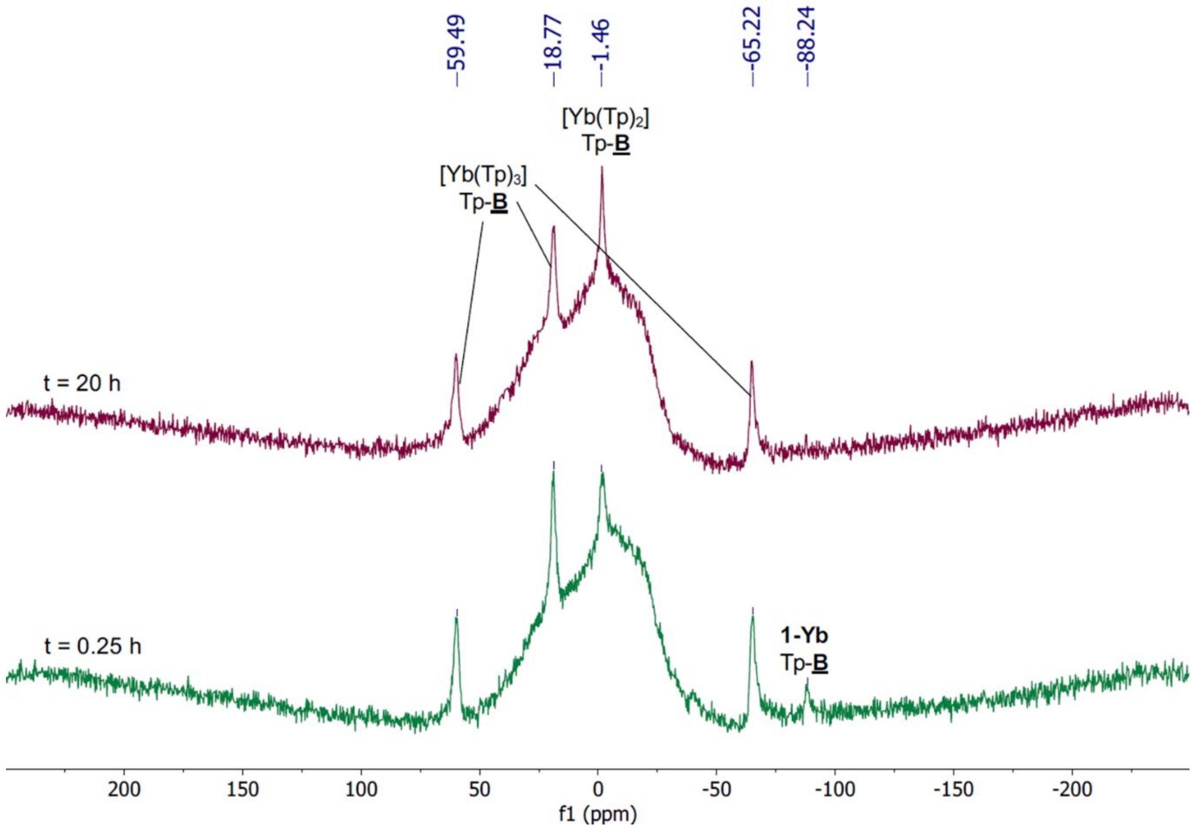


**Figure S 68.** $^{11}B$ NMR spectra of the NMR-scale reaction between [Yb(Tp)$_2$($\mu$-H)$_2$Al(H)(N'')] **2-Yb** with K(N'') in $d_6$-benzene at t = 0.25 h and t = 20 h, consistent with consumption of **2-Yb**, intermediate formation of **1-Yb** (t = 0.25 h) followed by consumption of **1-Yb** and gradual increase in the formation of [Yb(Tp)$_2$][5] (t = 20 h) and [Yb(Tp)$_3$][6] under ambient temperatures.

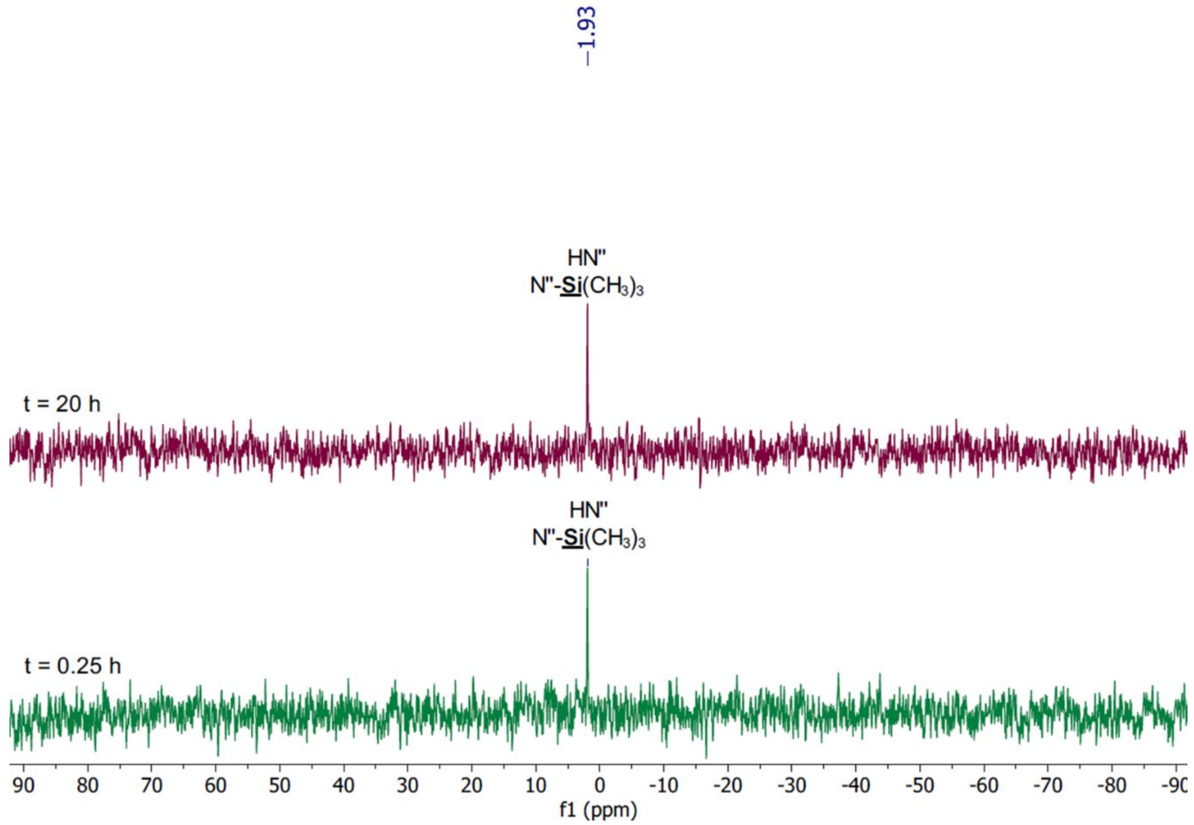


**Figure S 69.** $^{29}Si\{^1H\}$ INEPT NMR spectrum of the NMR-scale reaction between [Yb(Tp)$_2$($\mu$-H)$_2$Al(H)(N'')] **2-Yb** with K(N'') in $d_6$-benzene, consistent with consumption of **2-Yb** and formation of HN'' under ambient temperatures.

## B2.8 NMR-scale reaction between [Y(Tp)$_2$($\mu$-H)$_2$Al(H)(N'')] 2-Y with three equivalents of CyN=C=NCy (Cy = $C_6H_{11}$, DCC) and purified by washing [Al{$\kappa^2$-(Cy)NCHN(Cy)}$_2$(N'')] 3-Al away with hexane

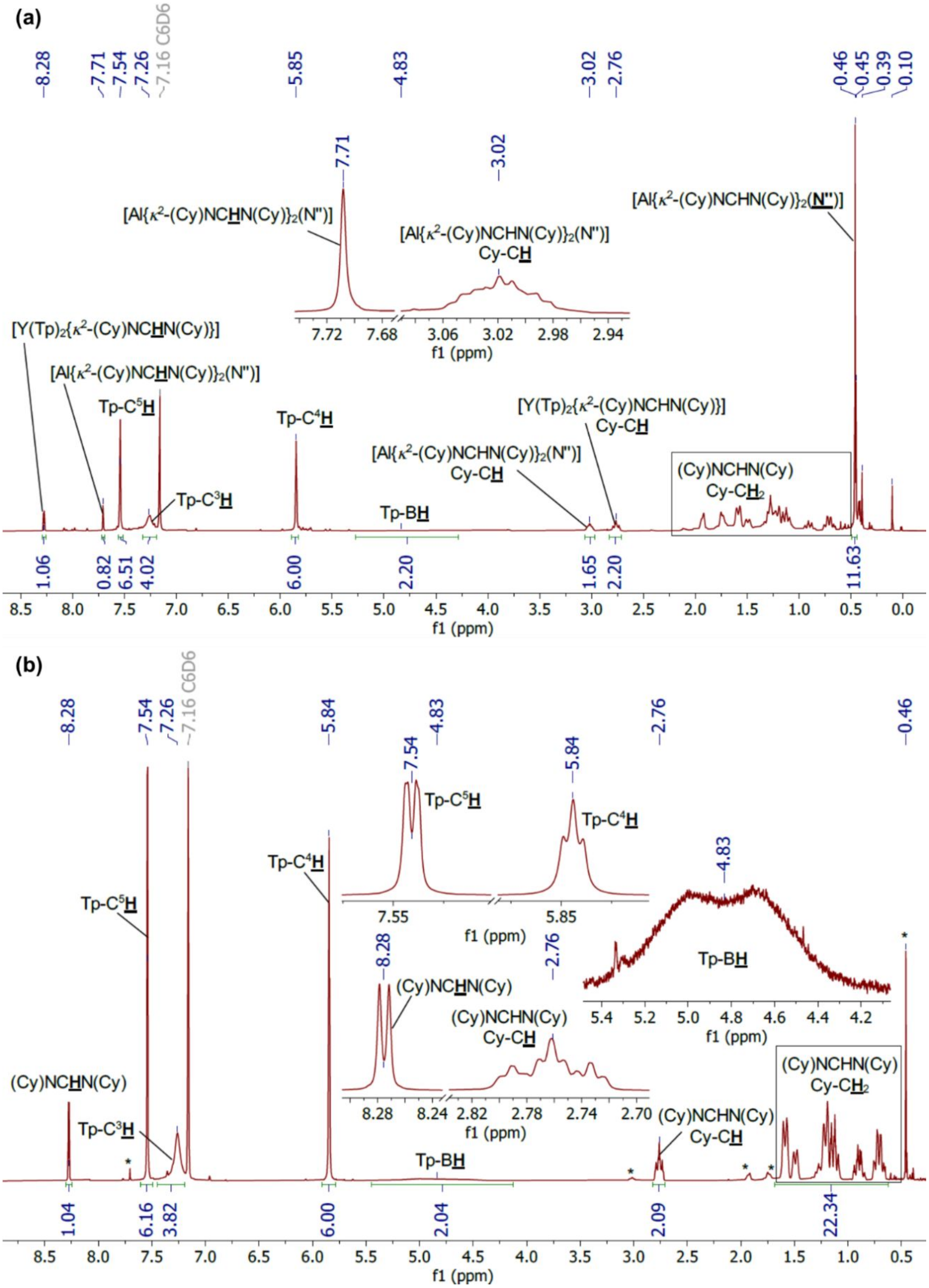


**Figure S 70.** $^1$H NMR spectrum of the NMR-scale reaction between [Y(Tp)$_2$($\mu$-H)$_2$Al(H)(N'')] **2-Y** with three equivalents of CyN=C=NCy in $d_6$-benzene (a), consistent with consumption of **2-Y** and formation of [Y(Tp)$_2${$\kappa^2$-(Cy)NCHN(Cy)}] **3-Y** and [Al{$\kappa^2$-(Cy)NCHN(Cy)}$_2$(N'')] **3-Al** under ambient temperatures. Solvent removed *in vacuo* from JY NMR tube and solids washed with hexane to remove away [Al{$\kappa^2$-(Cy)NCHN(Cy)}$_2$(N'')] **3-Al** (minor amounts denoted with *) and purify [Y(Tp)$_2${$\kappa^2$-(Cy)NCHN(Cy)}] **3-Y**, thus verifying no connections between the two products (b).

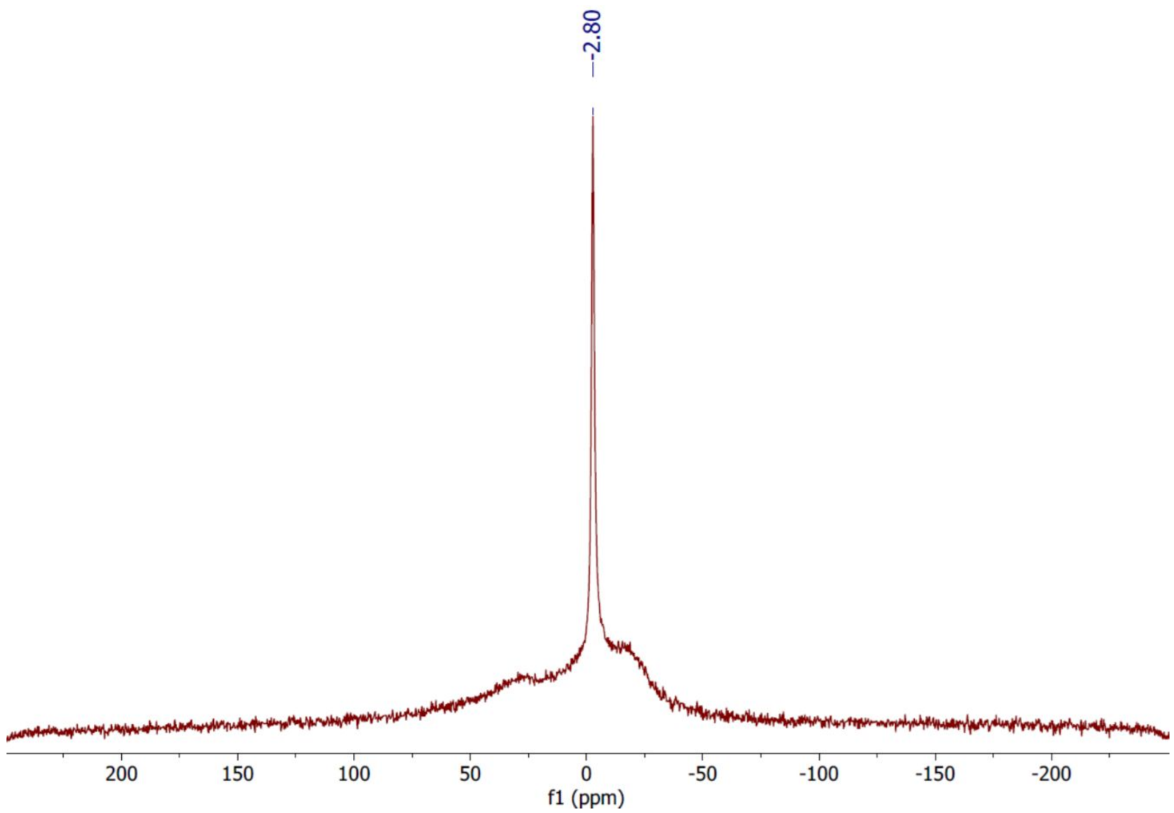


**Figure S 71.** $^{11}B$ NMR spectrum of the NMR-scale reaction between $[Y(Tp)_2(\mu\text{-}H)_2Al(H)(N'')]$ **2-Y** with three equivalents of CyN=C=NCy in $d_6$-benzene, consistent with consumption of **2-Y** and formation of $[Y(Tp)_2\{\kappa^2\text{-}(Cy)NCHN(Cy)\}]$ **3-Y** under ambient temperatures.

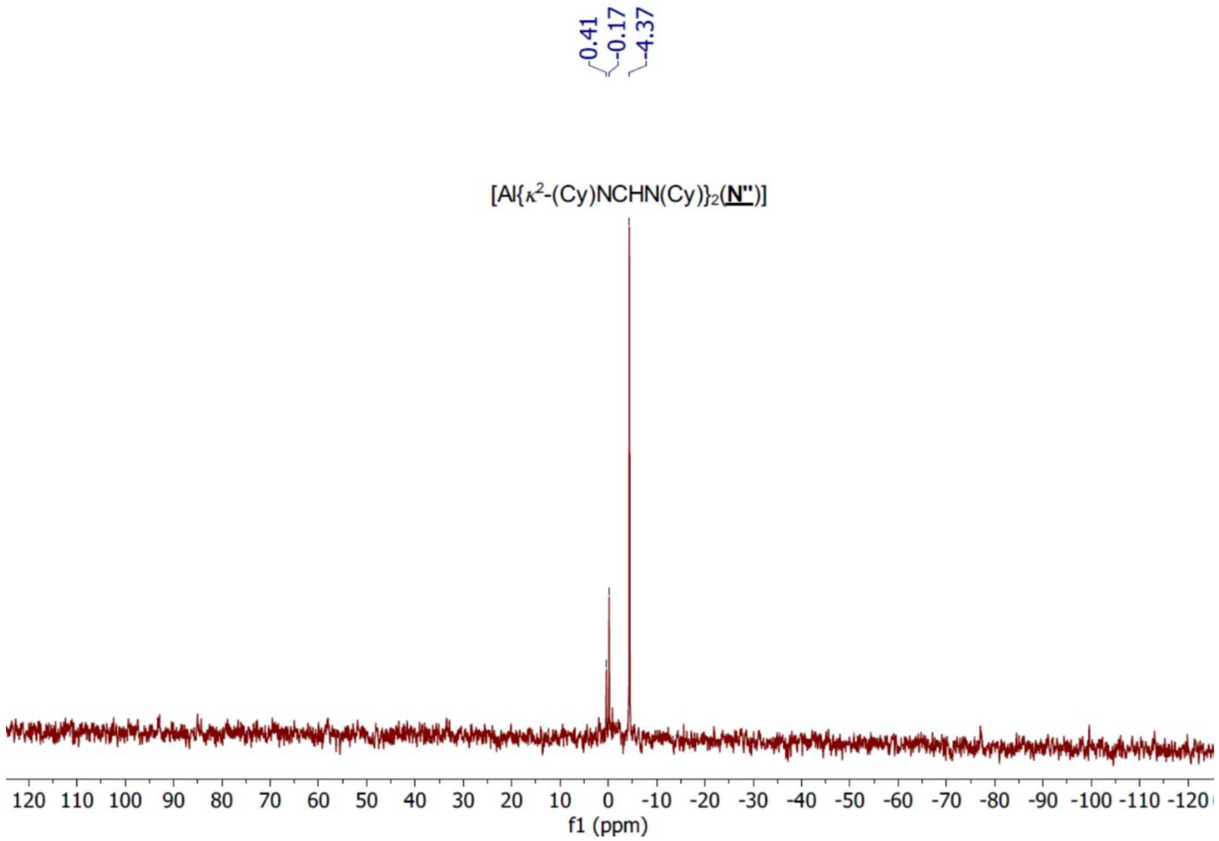


**Figure S 72.** $^{29}Si\{^1H\}$ INEPT NMR spectrum of the NMR-scale reaction between $[Y(Tp)_2(\mu\text{-}H)_2Al(H)(N'')]$ **2-Y** with three equivalents of CyN=C=NCy in $d_6$-benzene, consistent with consumption of **2-Y** and formation of $[Al\{\kappa^2\text{-}(Cy)NCHN(Cy)\}_2(N'')]$ **3-Al** and minor unidentified byproducts under ambient temperatures.

### B2.9 NMR-scale reaction between [Sm(Tp)$_2$($\mu$-H)$_2$Al(H)(N")] 2-Sm with three equivalents of CyN=C=NCy (Cy = $C_6H_{11}$, DCC) and purified by washing [Al{$\kappa^2$-(Cy)NCHN(Cy)}$_2$(N")] 3-Al away with hexane

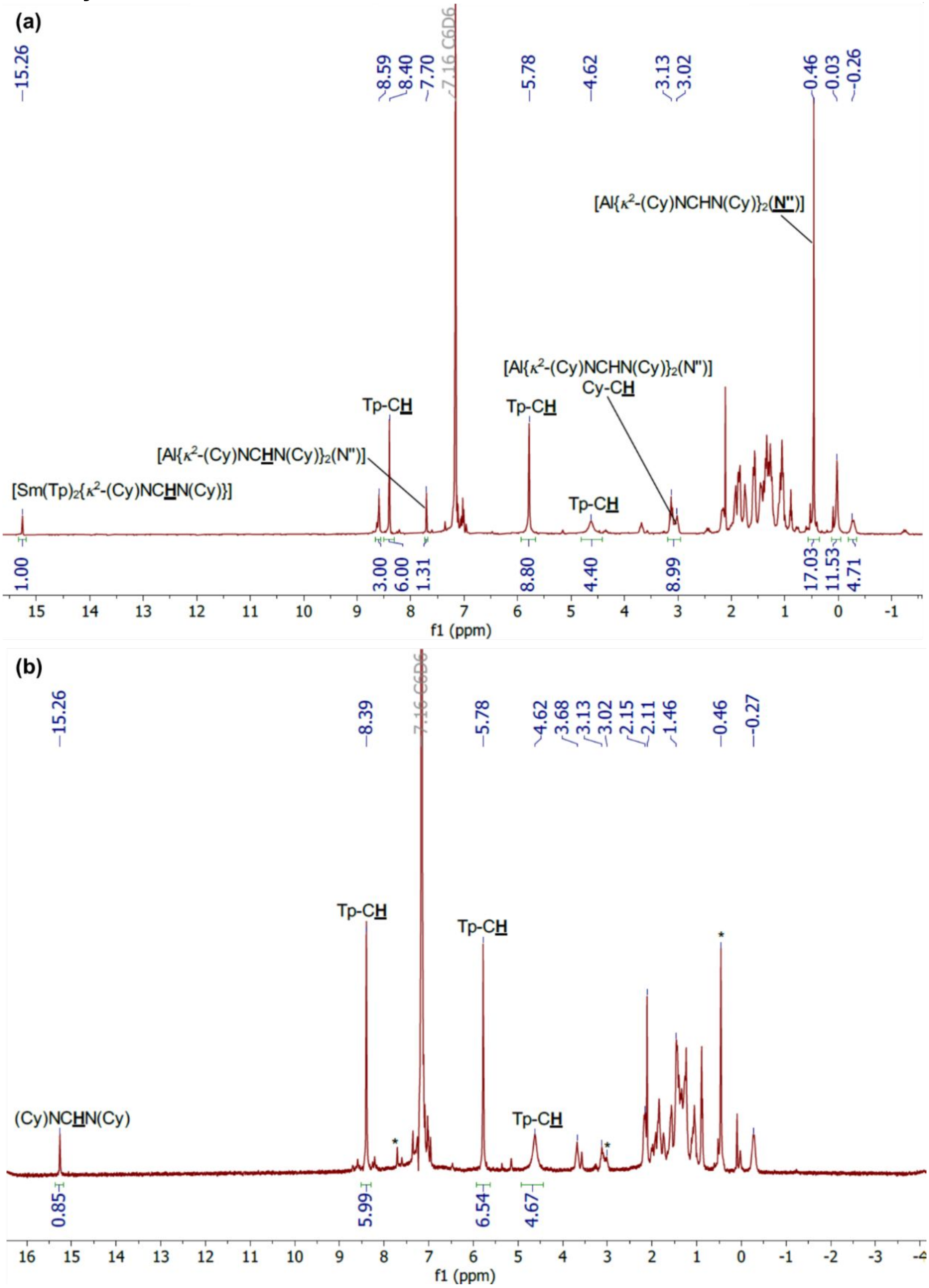


**Figure S 73.** $^1$H NMR spectrum of the NMR-scale reaction between [Sm(Tp)$_2$($\mu$-H)$_2$Al(H)(N")] **2-Sm** with three equivalents of CyN=C=NCy in $d_6$-benzene (a), consistent with consumption of **2-Sm** and formation of [Sm(Tp)$_2${$\kappa^2$-(Cy)NCHN(Cy)}] **3-Sm**, [Al{$\kappa^2$-(Cy)NCHN(Cy)}$_2$(N")] **3-Al** and unidentified byproducts under ambient temperatures. Solvent removed *in vacuo* from JY NMR tube and solids washed with hexane to remove away [Al{$\kappa^2$-(Cy)NCHN(Cy)}$_2$(N")] **3-Al** (minor amounts denoted with *) and purify sparingly soluble [Sm(Tp)$_2${$\kappa^2$-(Cy)NCHN(Cy)}] **3-Sm**, thus verifying no connections between the two products (b). In (a) and (b), the cyclohexyl ring protons of [Sm(Tp)$_2${$\kappa^2$-(Cy)NCHN(Cy)}] **3-Sm** were not assigned owing to being paramagnetically shifted.

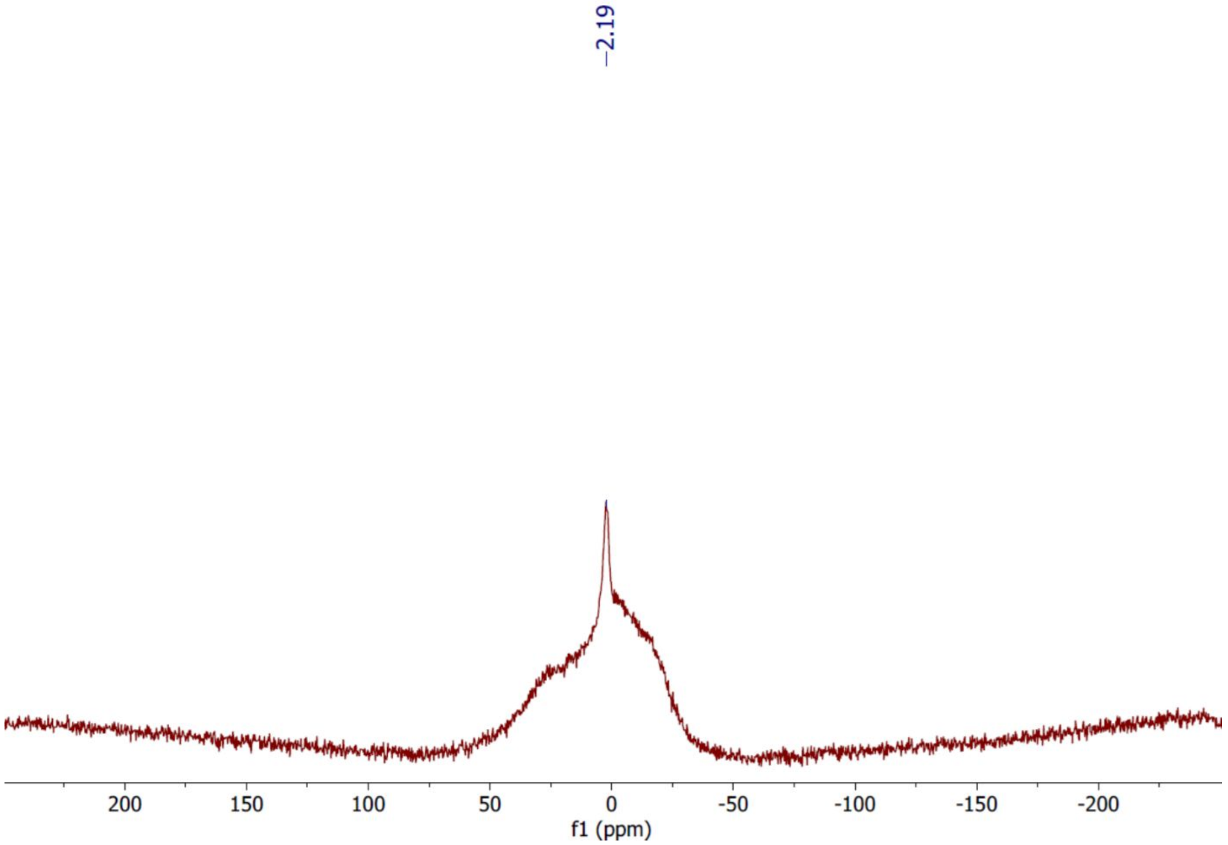


**Figure S 74.** $^{11}B$ NMR spectrum of the NMR-scale reaction between $[Sm(Tp)_2(\mu\text{-}H)_2Al(H)(N'')]$ **2-Sm** with three equivalents of CyN=C=NCy in $d_6$-benzene, consistent with consumption of **2-Sm** and formation of $[Sm(Tp)_2\{\kappa^2\text{-}(Cy)NCHN(Cy)\}]$ **3-Sm** under ambient temperatures.

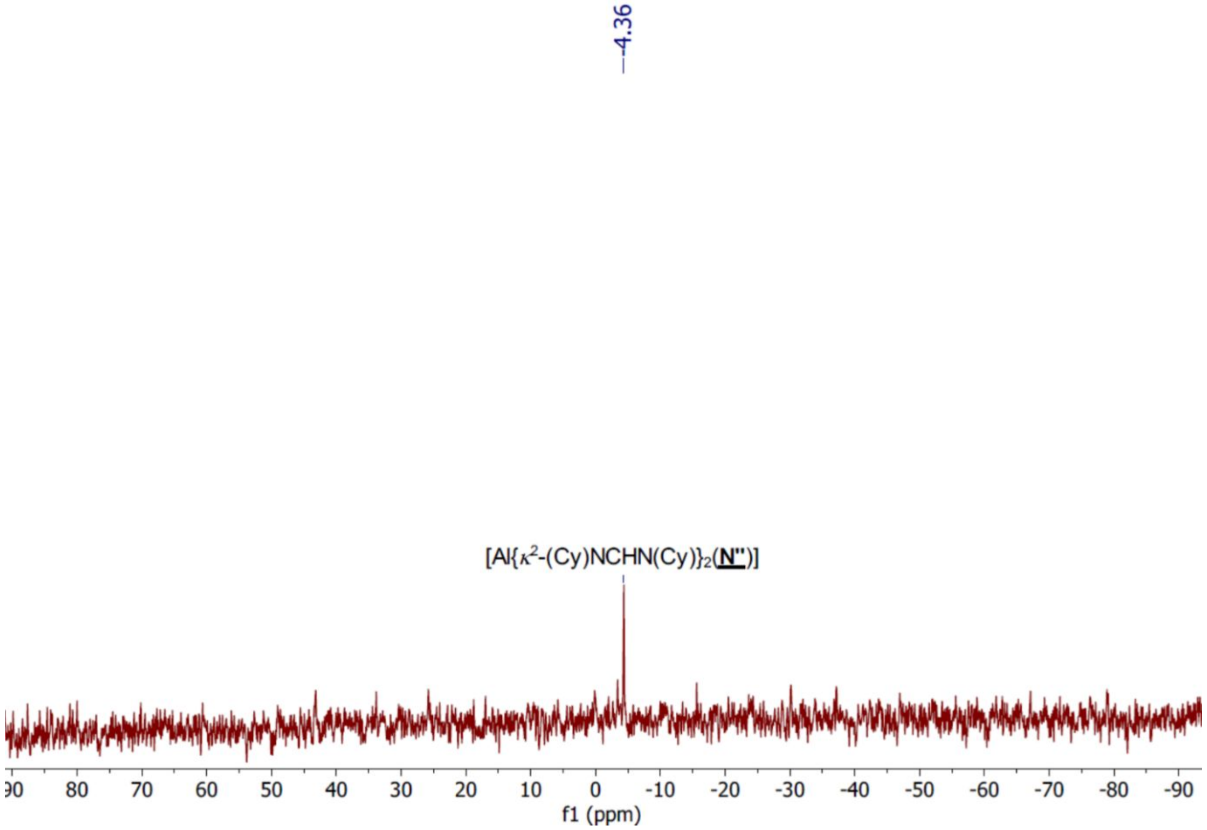


**Figure S 75.** $^{29}Si\{^1H\}$ INEPT NMR spectrum of the NMR-scale reaction between $[Sm(Tp)_2(\mu\text{-}H)_2Al(H)(N'')]$ **2-Sm** with three equivalents of CyN=C=NCy in $d_6$-benzene, consistent with consumption of **2-Sm** and formation of $[Al\{\kappa^2\text{-}(Cy)NCHN(Cy)\}_2(N'')]$ **3-Al** under ambient temperatures.

### B2.10 NMR-scale reaction between [Y(Tp)$_2$($\mu$-H)$_2$Al(H)(N")] 2-Y with excess $^i$PrN=C=N$^i$Pr (DIC)

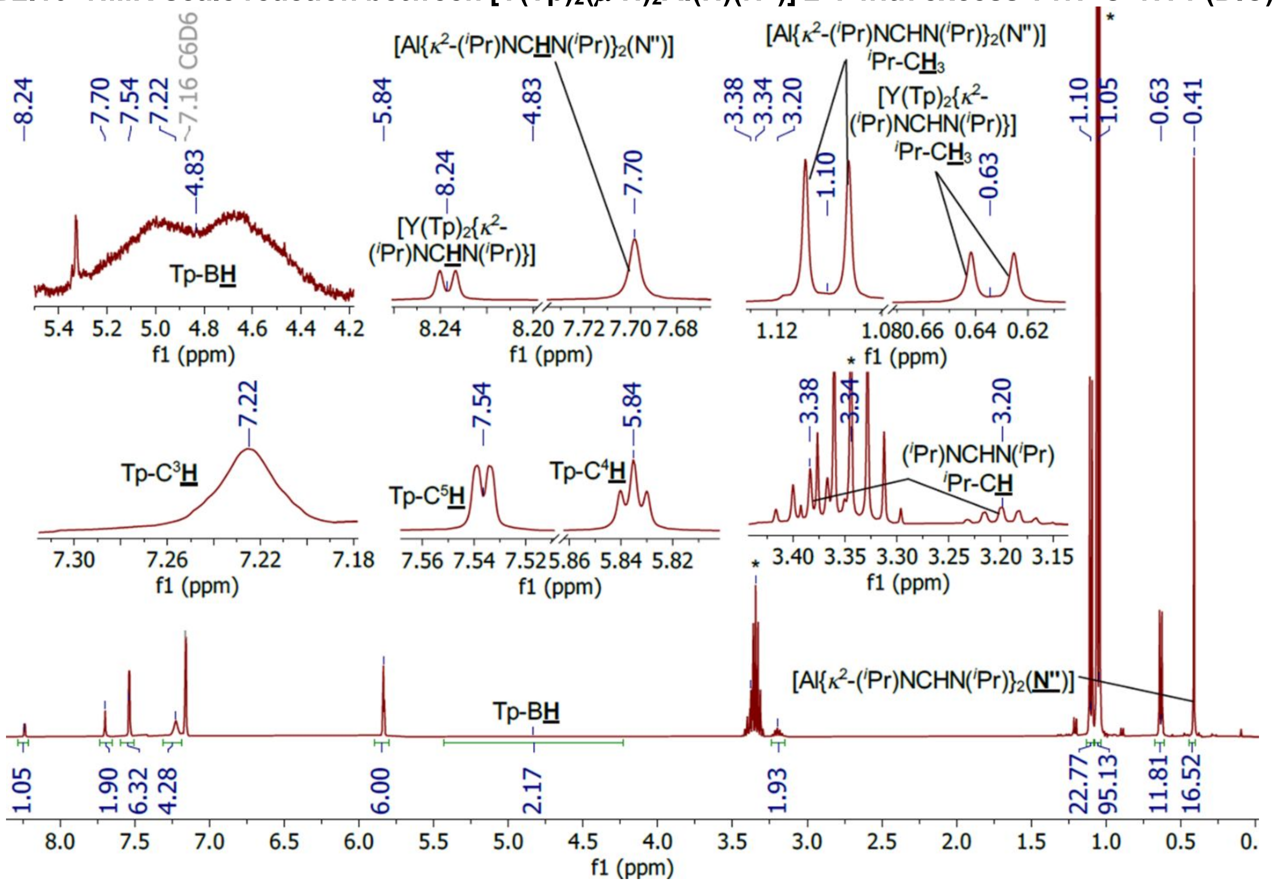


**Figure S 76.** $^{1}$H NMR spectrum of the NMR-scale reaction between [Y(Tp)$_2$($\mu$-H)$_2$Al(H)(N")] **2-Y** with 8.3 equivalents (denoted with *) of $^i$PrN=C=N$^i$Pr in $d_6$-benzene, consistent with consumption of **2-Y** and formation of [Y(Tp)$_2${$\kappa^2$-($^i$Pr)NCHN($^i$Pr)}] and [Al{$\kappa^2$-($^i$Pr)NCHN($^i$Pr)}$_2$(N")] **3-Al** under ambient temperatures. The $^i$Pr-C**H** resonances of the two products have not been assigned definitively.

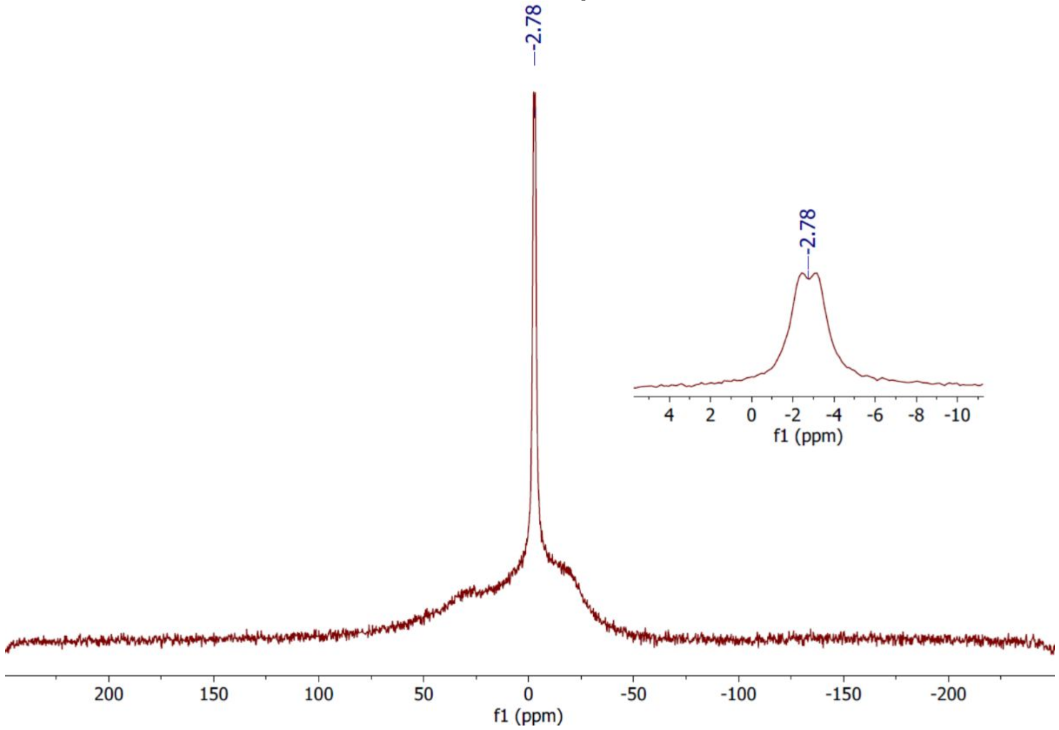


**Figure S 77.** $^{11}$B NMR spectrum of the NMR-scale reaction between [Y(Tp)$_2$($\mu$-H)$_2$Al(H)(N")] **2-Y** with 8.3 equivalents of $^i$PrN=C=N$^i$Pr in $d_6$-benzene, consistent with consumption of **2-Y** and formation of [Y(Tp)$_2${$\kappa^2$-($^i$Pr)NCHN($^i$Pr)}] under ambient temperatures.

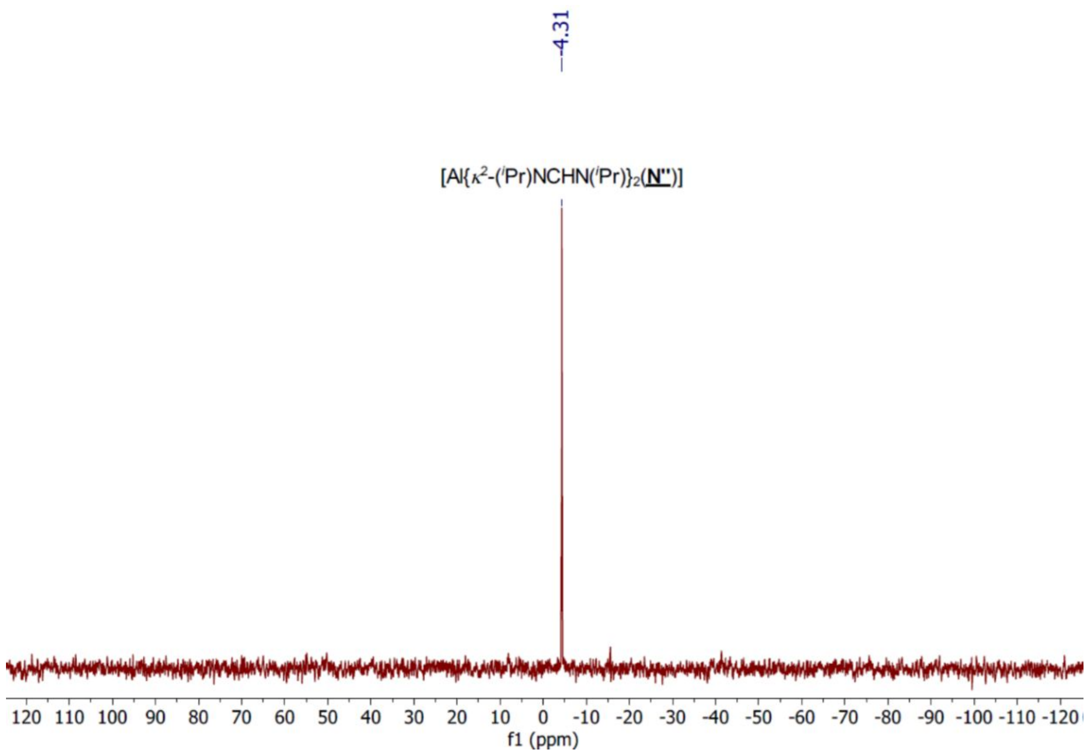


**Figure S 78.** $^{29}$Si{$^{1}$H} INEPT NMR spectrum of the NMR-scale reaction between [Y(Tp)$_2$($\mu$-H)$_2$Al(H)(N'')] **2-Y** with 8.3 equivalents of $^{i}$PrN=C=N$^{i}$Pr in $d_6$-benzene, consistent with consumption of **2-Y** and formation of [Al{$\kappa^2$-($^{i}$Pr)NCHN($^{i}$Pr)}$_2$(N'')] **3-Al** under ambient temperatures.

### B2.11 NMR-scale reaction between [Sm(Tp)$_2$($\mu$-H)$_2$Al(H)(N'')] 2-Sm with excess $^{i}$PrN=C=N$^{i}$Pr (DIC)

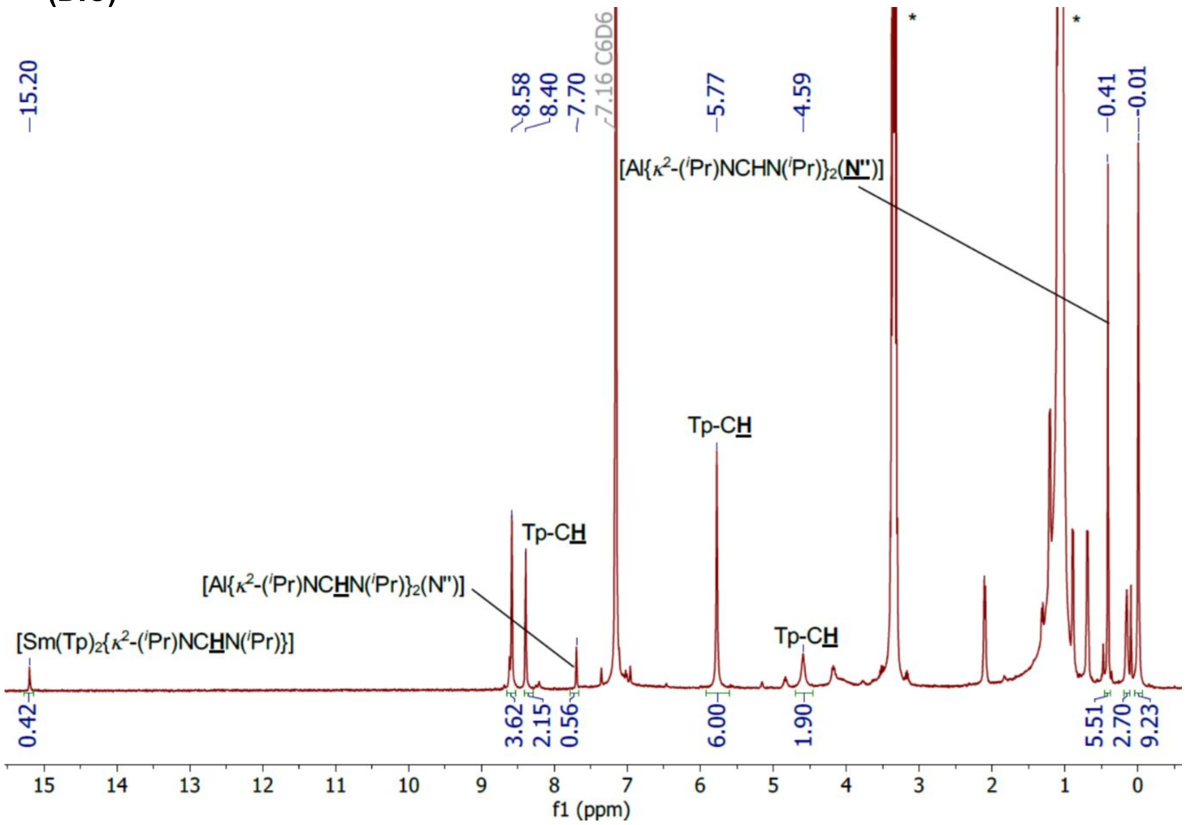


**Figure S 79.** $^{1}$H NMR spectrum of the NMR-scale reaction between [Sm(Tp)$_2$($\mu$-H)$_2$Al(H)(N'')] **2-Sm** with 32.0 equivalents (denoted with *) of $^{i}$PrN=C=N$^{i}$Pr in $d_6$-benzene, consistent with consumption of **2-Sm** and formation of [Sm(Tp)$_2${$\kappa^2$-($^{i}$Pr)NCHN($^{i}$Pr)}], [Al{$\kappa^2$-($^{i}$Pr)NCHN($^{i}$Pr)}$_2$(N'')] **3-Al** and unidentified byproducts under ambient temperatures. The isopropyl protons of [Sm(Tp)$_2${$\kappa^2$-($^{i}$Pr)NCHN($^{i}$Pr)}] were not assigned owing to being paramagnetically shifted.

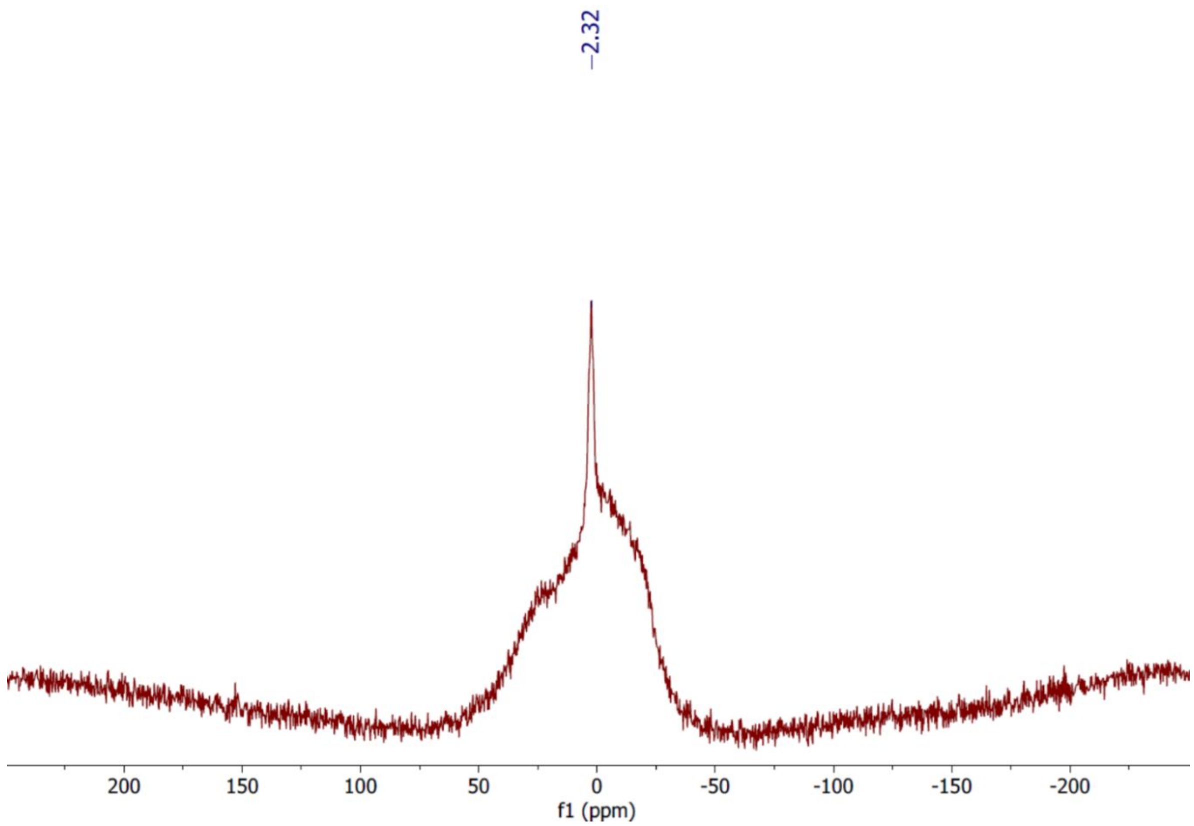


**Figure S 80.** $^{11}$B NMR spectrum of the NMR-scale reaction between [Sm(Tp)$_2$($\mu$-H)$_2$Al(H)(N")] **2-Sm** with 32.0 equivalents of $^i$PrN=C=N$^i$Pr in $d_6$-benzene, consistent with consumption of **2-Sm** and formation of [Sm(Tp)$_2${$\kappa^2$-($^i$Pr)NCHN($^i$Pr)}] under ambient temperatures.

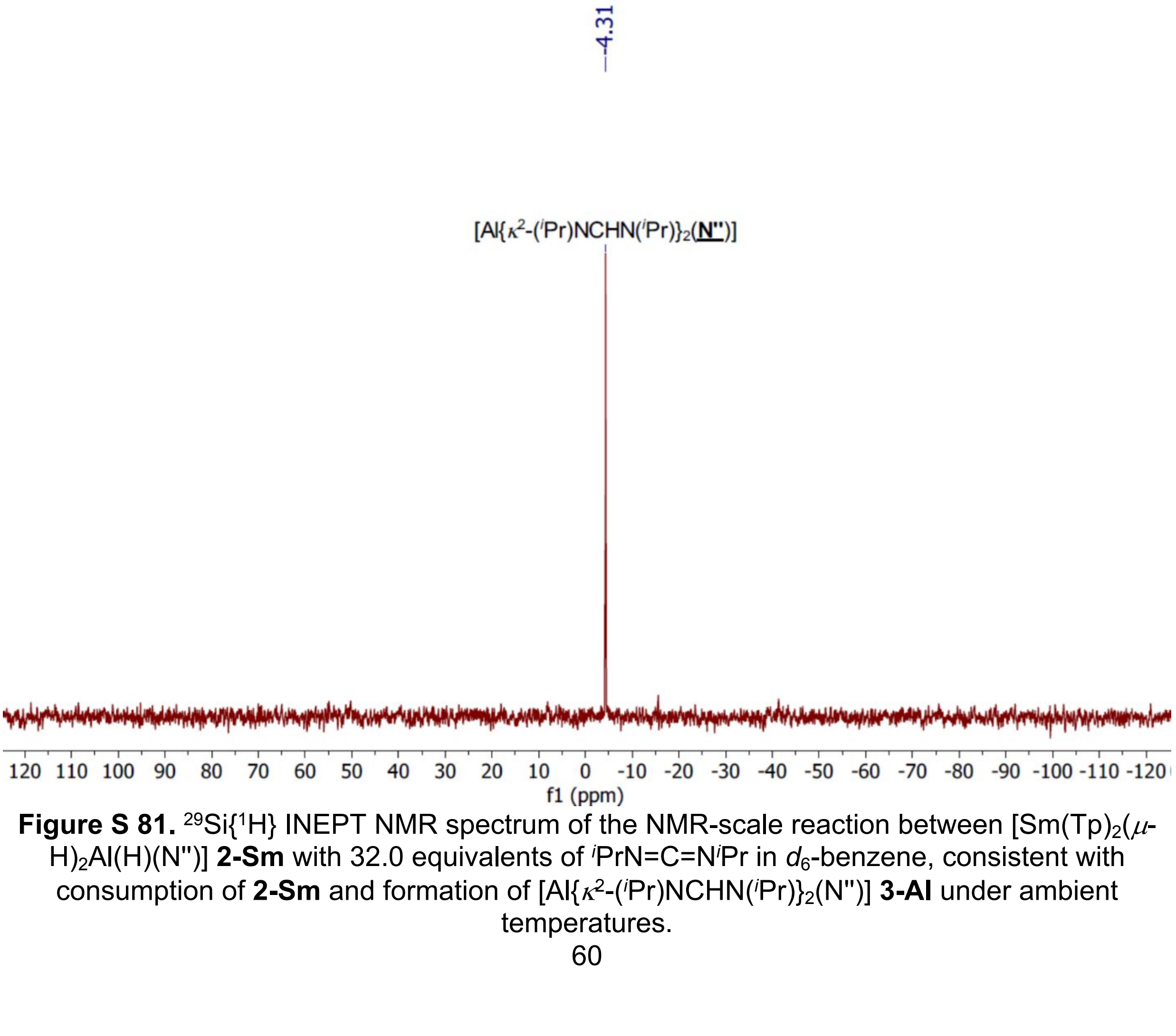


**Figure S 81.** $^{29}$Si{$^1$H} INEPT NMR spectrum of the NMR-scale reaction between [Sm(Tp)$_2$($\mu$-H)$_2$Al(H)(N")] **2-Sm** with 32.0 equivalents of $^i$PrN=C=N$^i$Pr in $d_6$-benzene, consistent with consumption of **2-Sm** and formation of [Al{$\kappa^2$-($^i$Pr)NCHN($^i$Pr)}$_2$(N")] **3-Al** under ambient temperatures.

**B2.12 NMR-scale reaction between [Dy(Tp)$_2$($\mu$-H)$_2$Al(H)(N")] 2-Dy with excess $^i$PrN=C=N$^i$Pr (DIC)**

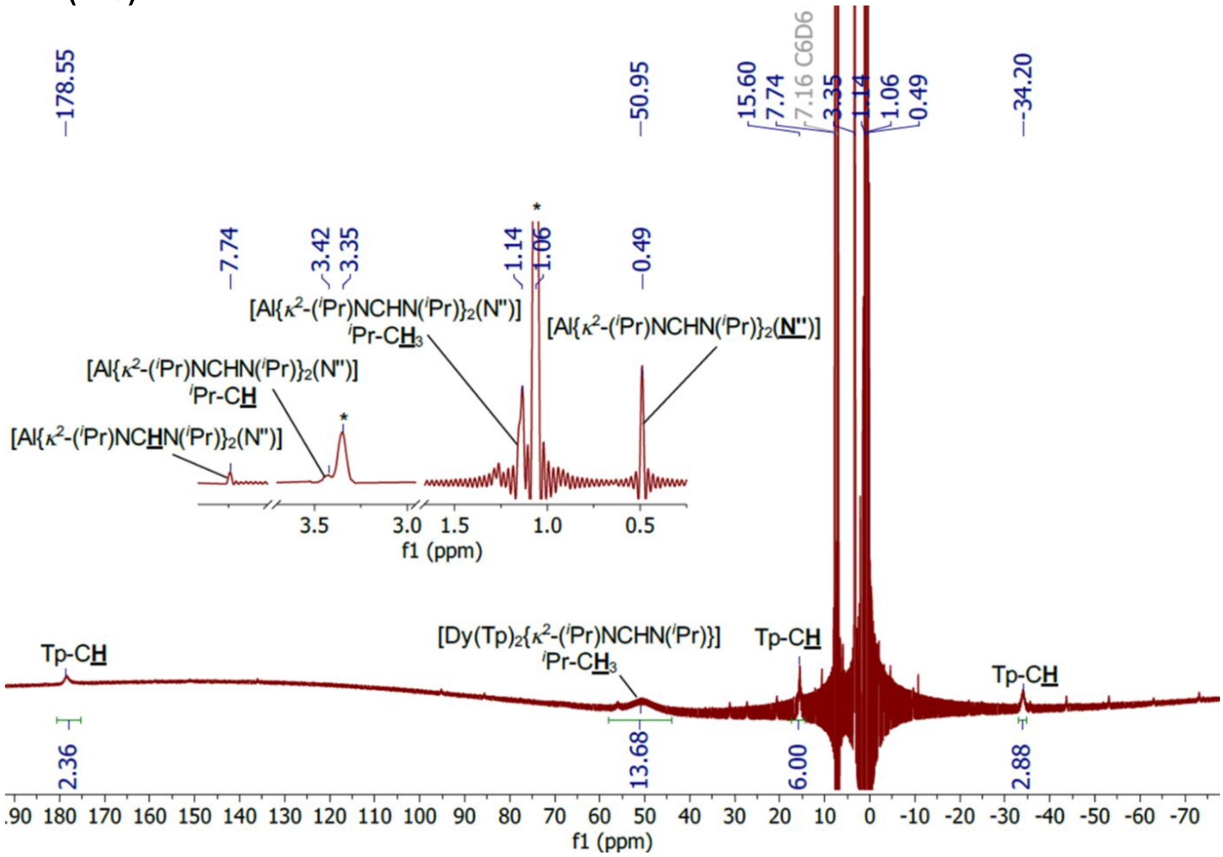


**Figure S 82.** $^1$H NMR spectrum of the NMR-scale reaction between [Dy(Tp)$_2$($\mu$-H)$_2$Al(H)(N")] **2-Dy** with 10.3 equivalents (denoted with *) of $^i$PrN=C=N$^i$Pr in $d_6$-benzene, consistent with consumption of **2-Dy** and formation of [Dy(Tp)$_2${$\kappa^2$-($^i$Pr)NCHN($^i$Pr)}] and [Al{$\kappa^2$-($^i$Pr)NCHN($^i$Pr)}$_2$(N")] **3-Al** under ambient temperatures. The [Dy(Tp)$_2${$\kappa^2$-( $^i$Pr)NC**H**N($^i$Pr)}] resonance was not observed similar to **3-Dy**.

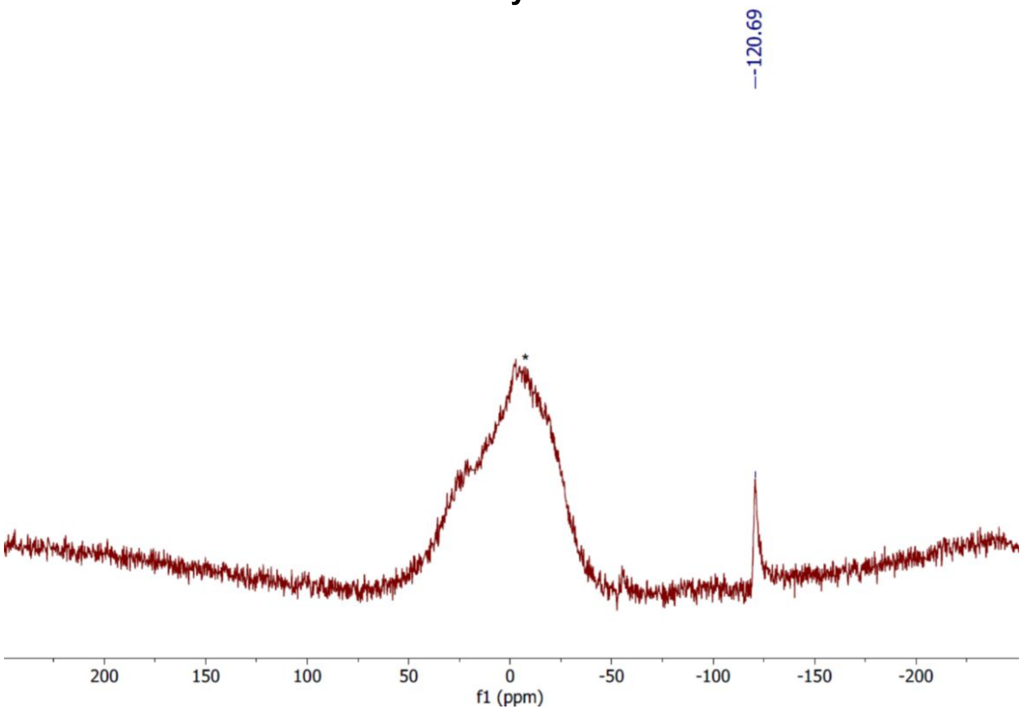


**Figure S 83.** $^{11}$B NMR spectrum of the NMR-scale reaction between [Dy(Tp)$_2$($\mu$-H)$_2$Al(H)(N")] **2-Dy** with 10.3 equivalents of $^i$PrN=C=N$^i$Pr in $d_6$-benzene, consistent with consumption of **2-Dy** and formation of [Dy(Tp)$_2${$\kappa^2$-($^i$Pr)NCHN($^i$Pr)}] under ambient temperatures. Borosilicate glass is denoted with *.

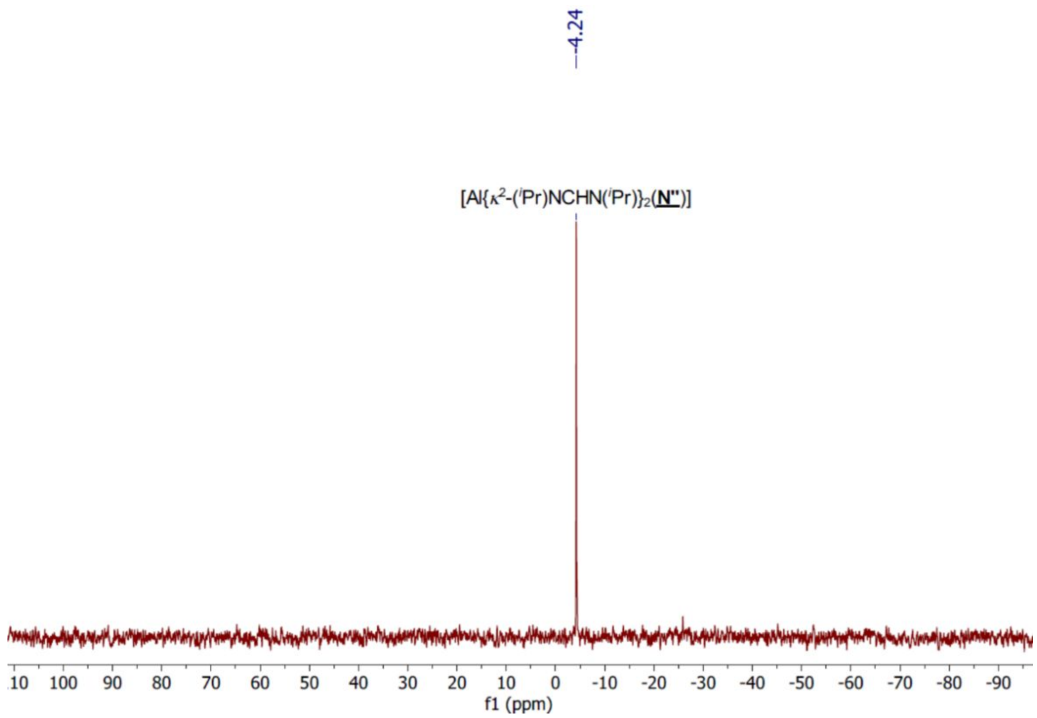


**Figure S 84.** $^{29}$Si{$^1$H} INEPT NMR spectrum of the NMR-scale reaction between [Dy(Tp)$_2$($\mu$-H)$_2$Al(H)(N")] **2-Dy** with 10.3 equivalents of $^i$PrN=C=N$^i$Pr in $d_6$-benzene, consistent with consumption of **2-Dy** and formation of [Al{$\kappa^2$-($^i$Pr)NCHN($^i$Pr)}$_2$(N")] **3-Al** under ambient temperatures.

### B2.13 NMR-scale reaction between [Yb(Tp)$_2$($\mu$-H)$_2$Al(H)(N")] 2-Yb with excess $^i$PrN=C=N$^i$Pr (DIC)

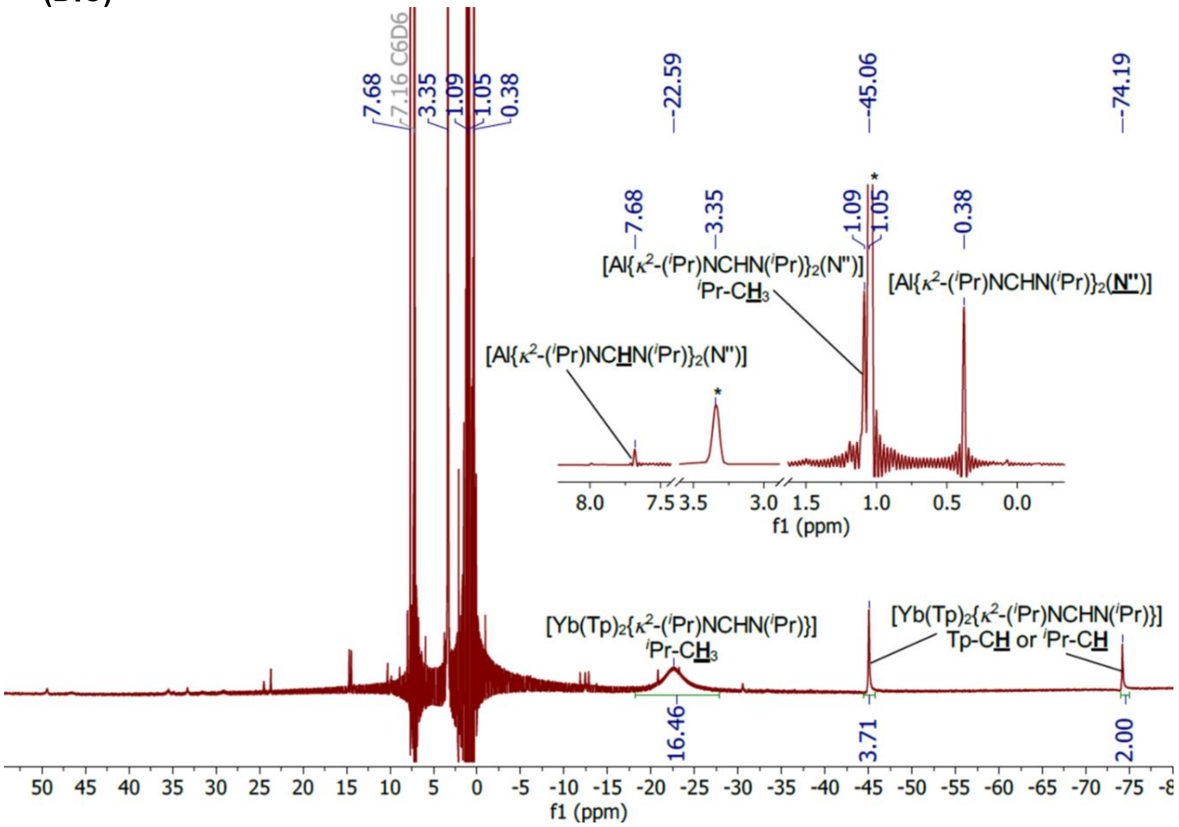


**Figure S 85.** $^1$H NMR spectrum of the NMR-scale reaction between [Yb(Tp)$_2$($\mu$-H)$_2$Al(H)(N")] **2-Yb** with 10.5 equivalents (denoted with *) of $^i$PrN=C=N$^i$Pr in $d_6$-benzene, consistent with consumption of **2-Yb** and formation of [Yb(Tp)$_2${$\kappa^2$-($^i$Pr)NCHN($^i$Pr)}] and [Al{$\kappa^2$-($^i$Pr)NCHN($^i$Pr)}$_2$(N")] **3-Al** under ambient temperatures. The [Yb(Tp)$_2${$\kappa^2$-( $^i$Pr)NC**H**N($^i$Pr)}] resonance was not observed similar to **3-Yb** and some of the Tp-pyrazolyl proton resonances were not observed.

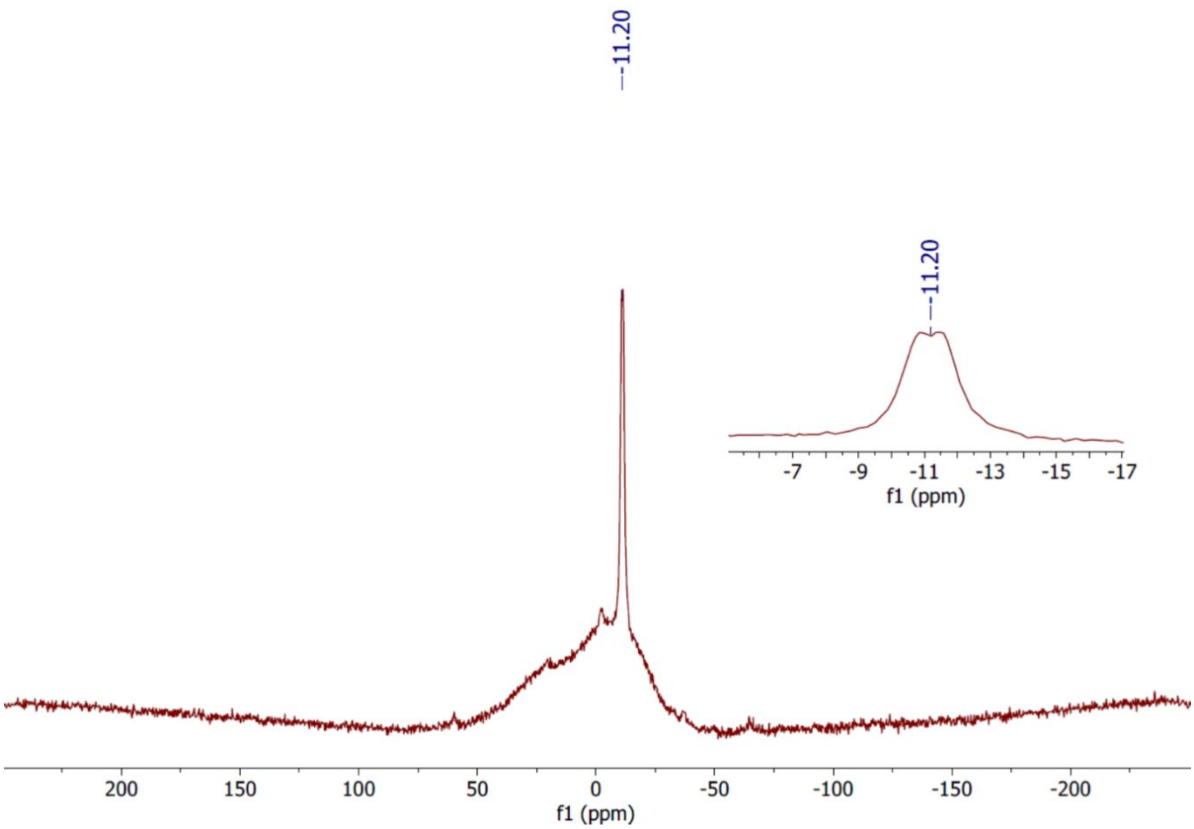


**Figure S 86.** $^{11}$B NMR spectrum of the NMR-scale reaction between [Yb(Tp)$_2$($\mu$-H)$_2$Al(H)(N")] **2-Yb** with 10.5 equivalents of $^i$PrN=C=N$^i$Pr in $d_6$-benzene, consistent with consumption of **2-Yb** and formation of [Yb(Tp)$_2${$\kappa^2$-($^i$Pr)NCHN($^i$Pr)}] and minor unidentified byproducts under ambient temperatures.

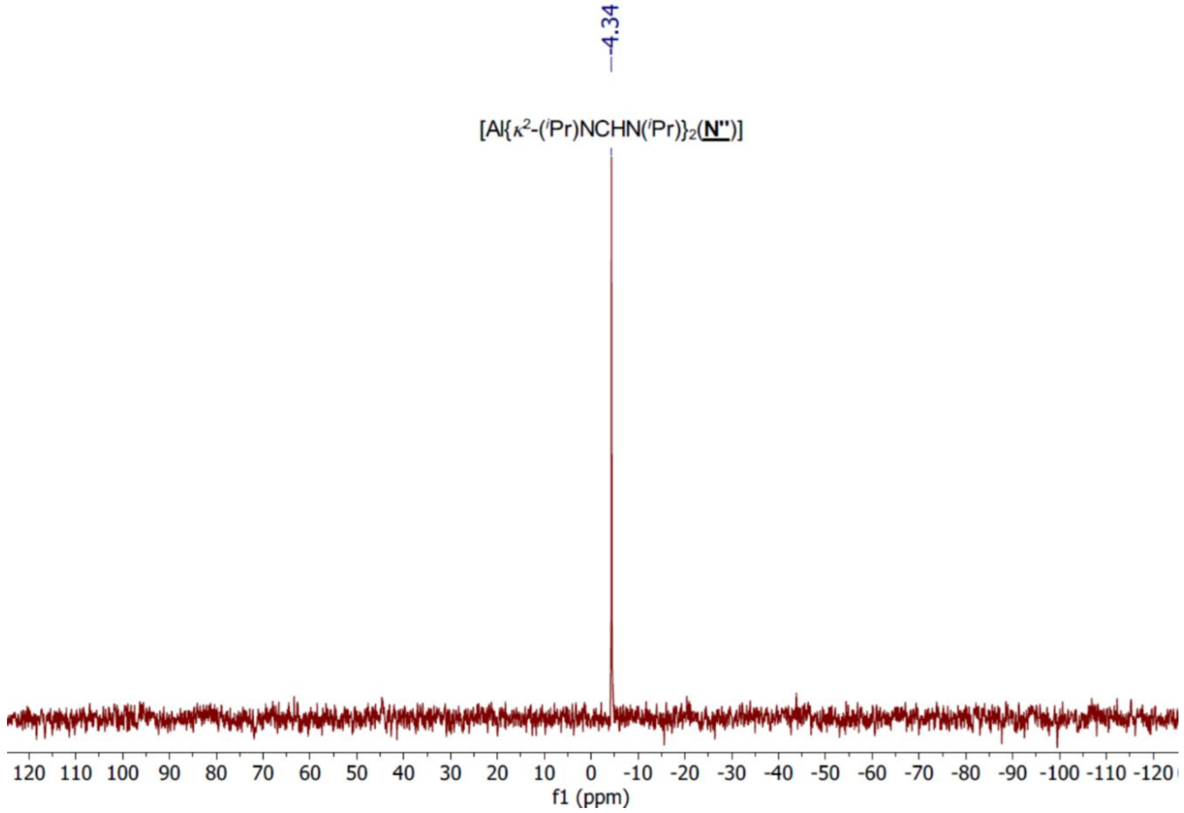


**Figure S 87.** $^{29}$Si{$^1$H} INEPT NMR spectrum of the NMR-scale reaction between [Yb(Tp)$_2$($\mu$-H)$_2$Al(H)(N")] **2-Yb** with 10.5 equivalents of $^i$PrN=C=N$^i$Pr in $d_6$-benzene, consistent with consumption of **2-Yb** and formation of [Al{$\kappa^2$-($^i$Pr)NCHN($^i$Pr)}$_2$(N")] **3-Al** under ambient temperatures.

### B2.14 NMR-scale reaction between [Y(Tp)$_2$($\mu$-H)$_2$Al(H)(N")] 2-Y with sequential addition of one, two, and three equivalents of benzophenone

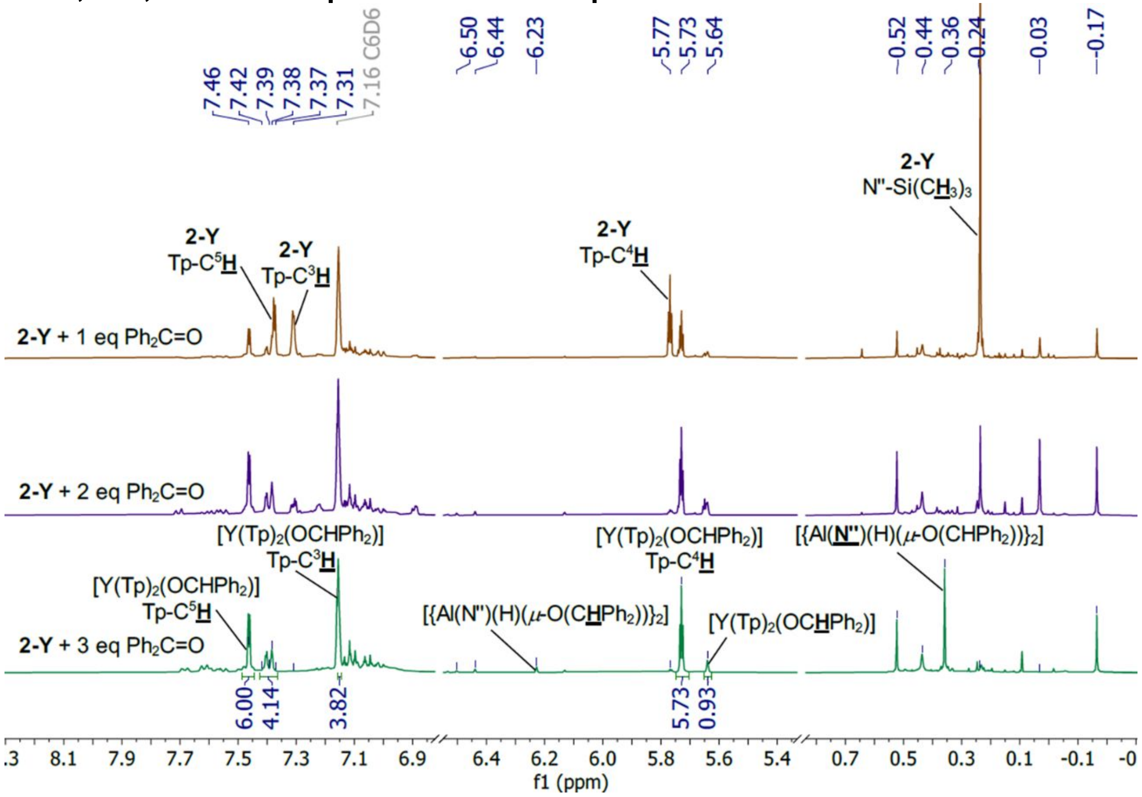


**Figure S 88.** $^{1}$H NMR spectra of the NMR-scale reaction between [Y(Tp)$_2$($\mu$-H)$_2$Al(H)(N")] **2-Y** with 1.2, 2.4, and 3.6 equivalents of benzophenone in $d_6$-benzene, consistent with gradual consumption of **2-Y**, formation of [Y(Tp)$_2$(OCHPh$_2$)], [{Al(N")(H)($\mu$-O(CHPh$_2$))}$_2$] **4-Al**, and unidentified Al byproducts under ambient temperatures. Complete consumption of **2-Y** and complete formation of [Y(Tp)$_2$(OCHPh$_2$)] and [{Al(N")(H)($\mu$-O(CHPh$_2$))}$_2$] **4-Al** are observed upon reaction of **2-Y** with three equivalents of benzophenone.

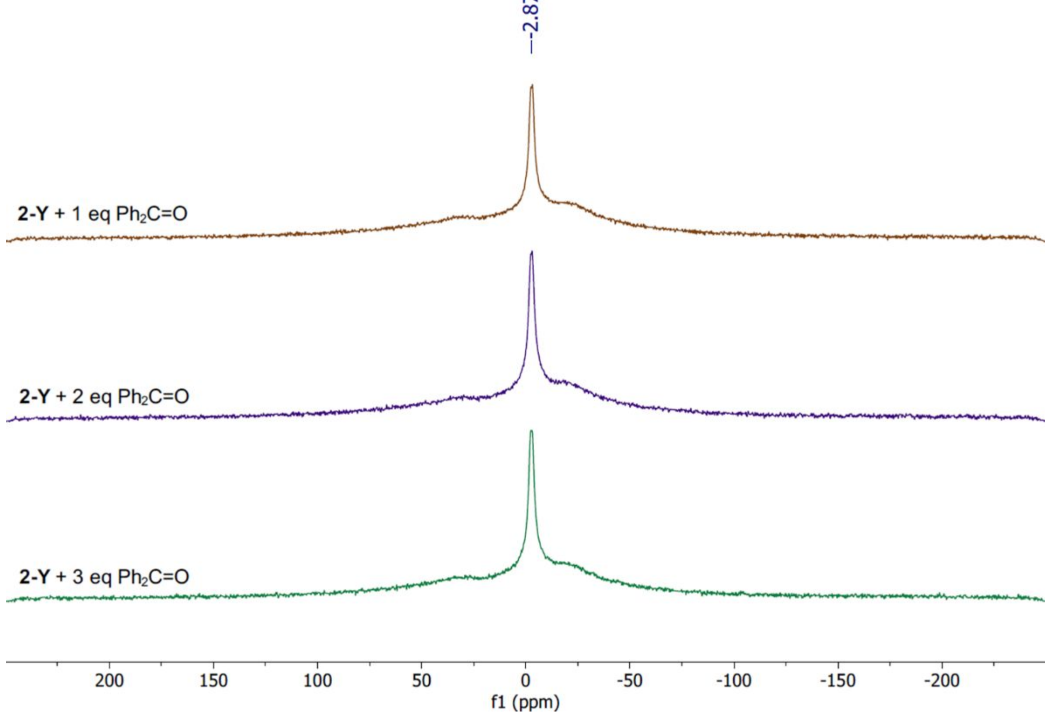


**Figure S 89.** $^{11}$B NMR spectra of the NMR-scale reaction between [Y(Tp)$_2$($\mu$-H)$_2$Al(H)(N")] **2-Y** with 1.2, 2.4, and 3.6 equivalents of benzophenone in $d_6$-benzene, consistent with gradual consumption of **2-Y** and formation of [Y(Tp)$_2$(OCHPh$_2$)] under ambient temperatures.

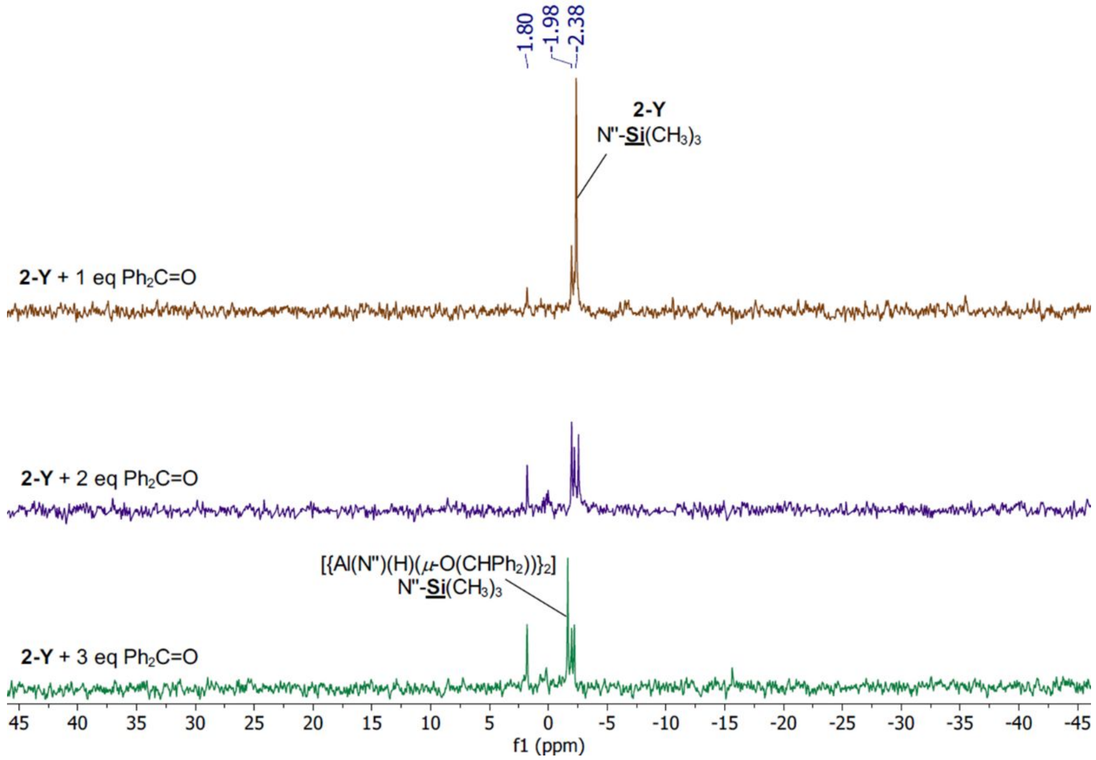


**Figure S 90.** $^{29}Si\{^{1}H\}$ INEPT NMR spectra of the NMR-scale reaction between $[Y(Tp)_2(\mu\text{-}H)_2Al(H)(N'')]$ **2-Y** with 1.2, 2.4, and 3.6 equivalents of benzophenone in $d_6$-benzene, consistent with gradual consumption of **2-Y** and formation of $[\{Al(N'')(H)(\mu\text{-}O(CHPh_2))\}_2]$ **4-Al** and unidentified Al byproducts under ambient temperatures. Complete consumption of **2-Y** is observed upon reaction of **2-Y** with three equivalents of benzophenone.

## B2.15 NMR-scale reaction between [Yb(Tp)$_2$($\mu$-H)$_2$Al(H)(N'')] 2-Yb with sequential addition of one, two, and three equivalents of benzophenone

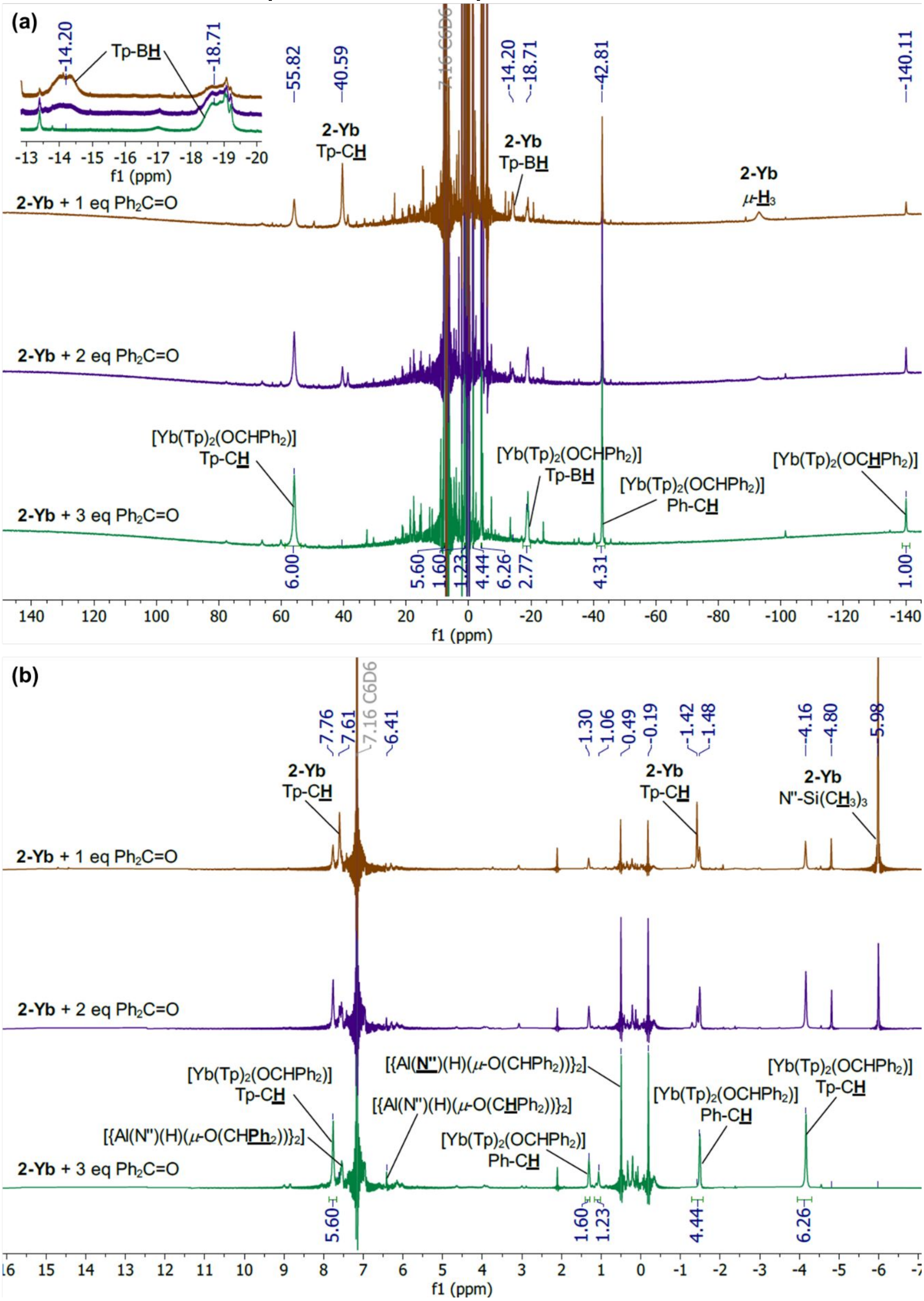


**Figure S 91.** $^{1}$H NMR spectra of the NMR-scale reaction between [Yb(Tp)$_2$($\mu$-H)$_2$Al(H)(N'')] **2-Yb** with 1.2, 2.4, and 3.4 equivalents of benzophenone in $d_6$-benzene, consistent with gradual consumption of **2-Yb**, formation of [Yb(Tp)$_2$(OCHPh$_2$)], [{Al(N'')(H)($\mu$-O(CHPh$_2$))}$_2$] **4-Al**, and unidentified Al byproducts under ambient temperatures. Complete consumption of **2-Yb** and complete formation of [Yb(Tp)$_2$(OCHPh$_2$)] and [{Al(N'')(H)($\mu$-O(CHPh$_2$))}$_2$] **4-Al** are observed upon reaction of **2-Yb** with three equivalents of benzophenone.

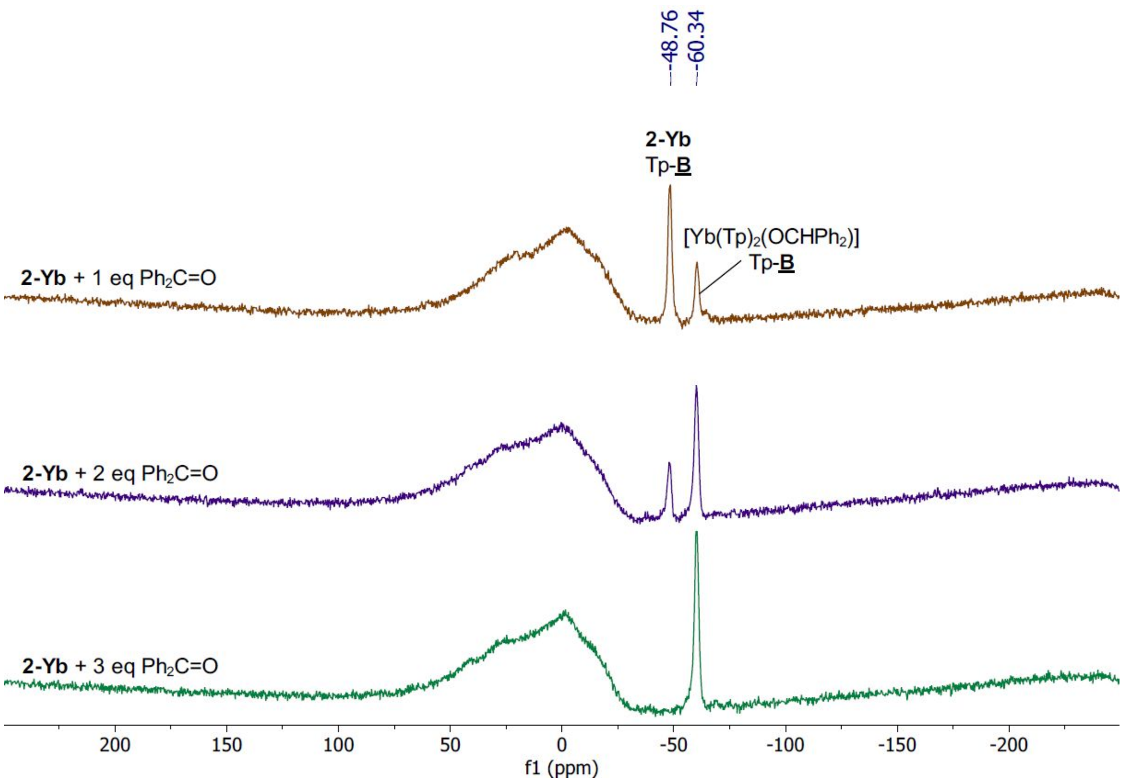


**Figure S 92.** $^{11}B$ NMR spectra of the NMR-scale reaction between [Yb(Tp)$_2$($\mu$-H)$_2$Al(H)(N")] **2-Yb** with 1.2, 2.4, and 3.4 equivalents of benzophenone in $d_6$-benzene, consistent with gradual consumption of **2-Yb** and formation of [Yb(Tp)$_2$(OCHPh$_2$)] under ambient temperatures. Complete consumption of **2-Yb** and complete formation of [Yb(Tp)$_2$(OCHPh$_2$)] are observed upon reaction of **2-Yb** with three equivalents of benzophenone.

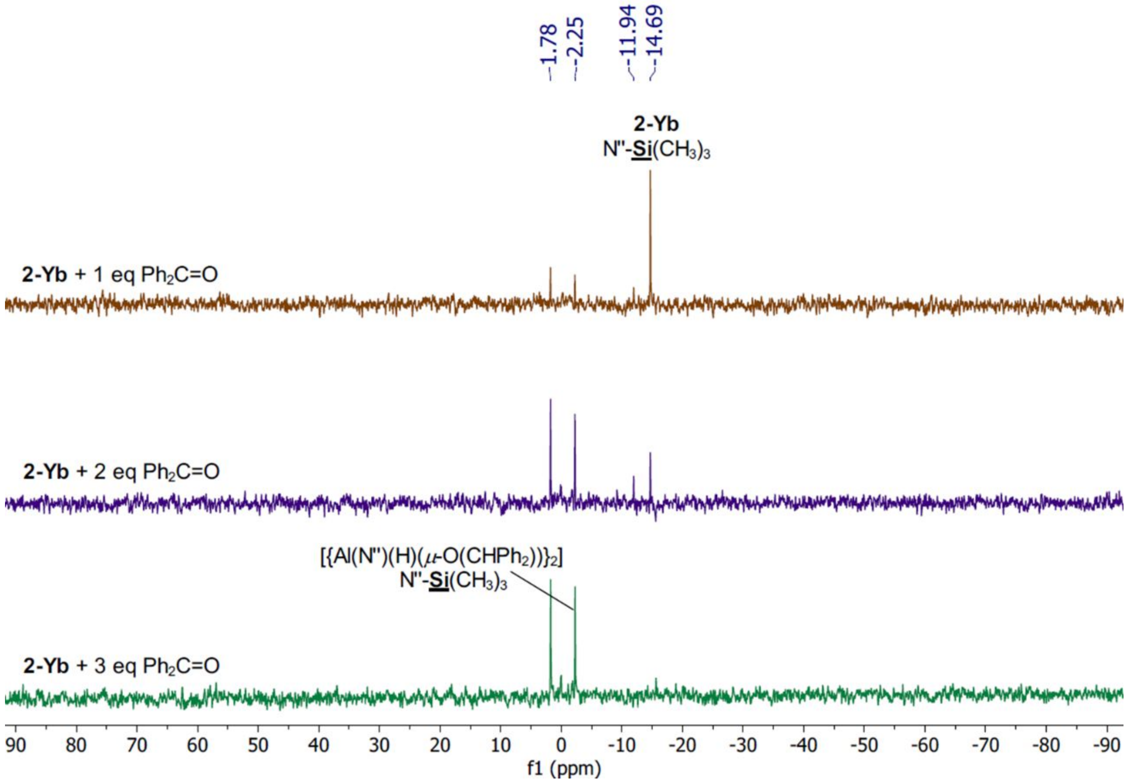


**Figure S 93.** $^{29}Si\{^1H\}$ INEPT NMR spectra of the NMR-scale reaction between [Yb(Tp)$_2$($\mu$-H)$_2$Al(H)(N")] **2-Yb** with 1.2, 2.4, and 3.4 equivalents of benzophenone in $d_6$-benzene, consistent with gradual consumption of **2-Yb** and formation of [{Al(N")(H)($\mu$-O(CHPh$_2$))}$_2$] **4-Al** and unidentified Al byproducts under ambient temperatures. Complete consumption of **2-Yb** is observed upon reaction of **2-Yb** with three equivalents of benzophenone.

### B2.16 NMR-scale reaction between [Sm(Tp)$_2$($\mu$-H)$_2$Al(H)(N")] 2-Sm with three equivalents of benzophenone

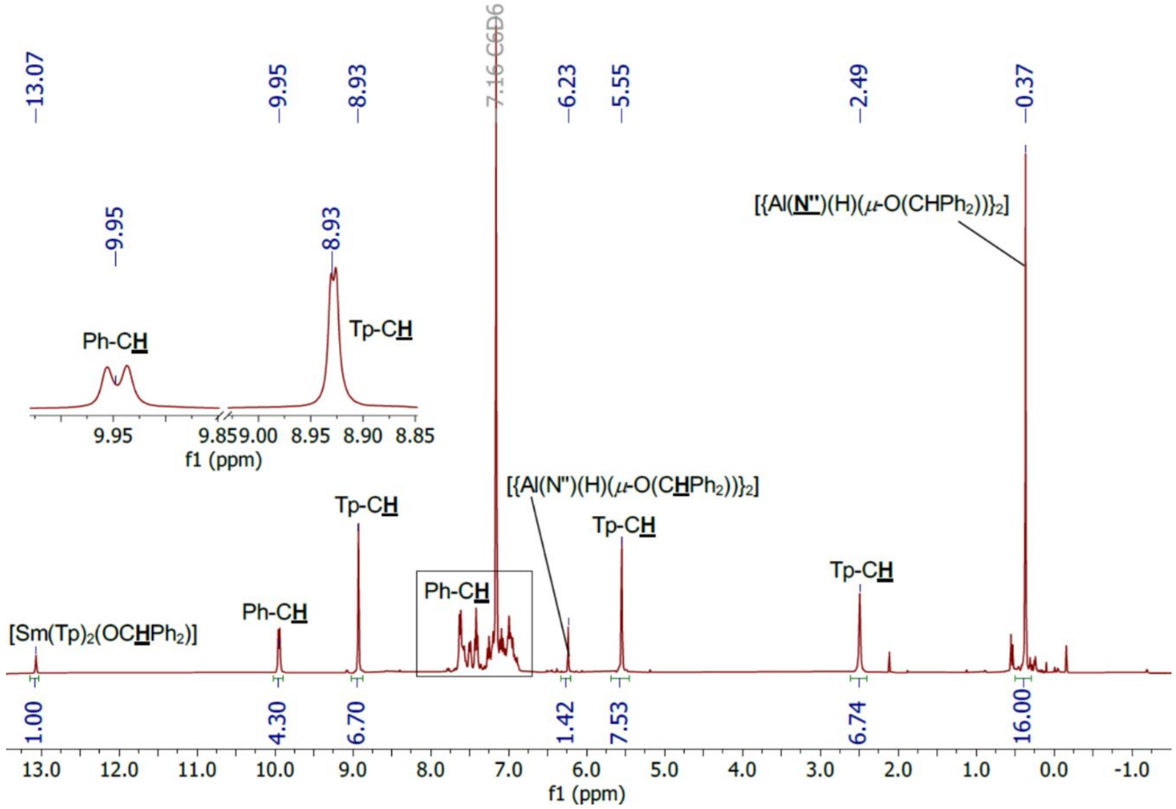


**Figure S 94.** $^{1}$H NMR spectrum of the NMR-scale reaction between [Sm(Tp)$_2$($\mu$-H)$_2$Al(H)(N")] **2-Sm** with 3.7 equivalents of benzophenone in $d_6$-benzene, consistent with consumption of **2-Sm**, formation of [Sm(Tp)$_2$(OCHPh$_2$)] and [{Al(N")(H)($\mu$-O(CHPh$_2$))}$_2$] **4-Al** under ambient temperatures.

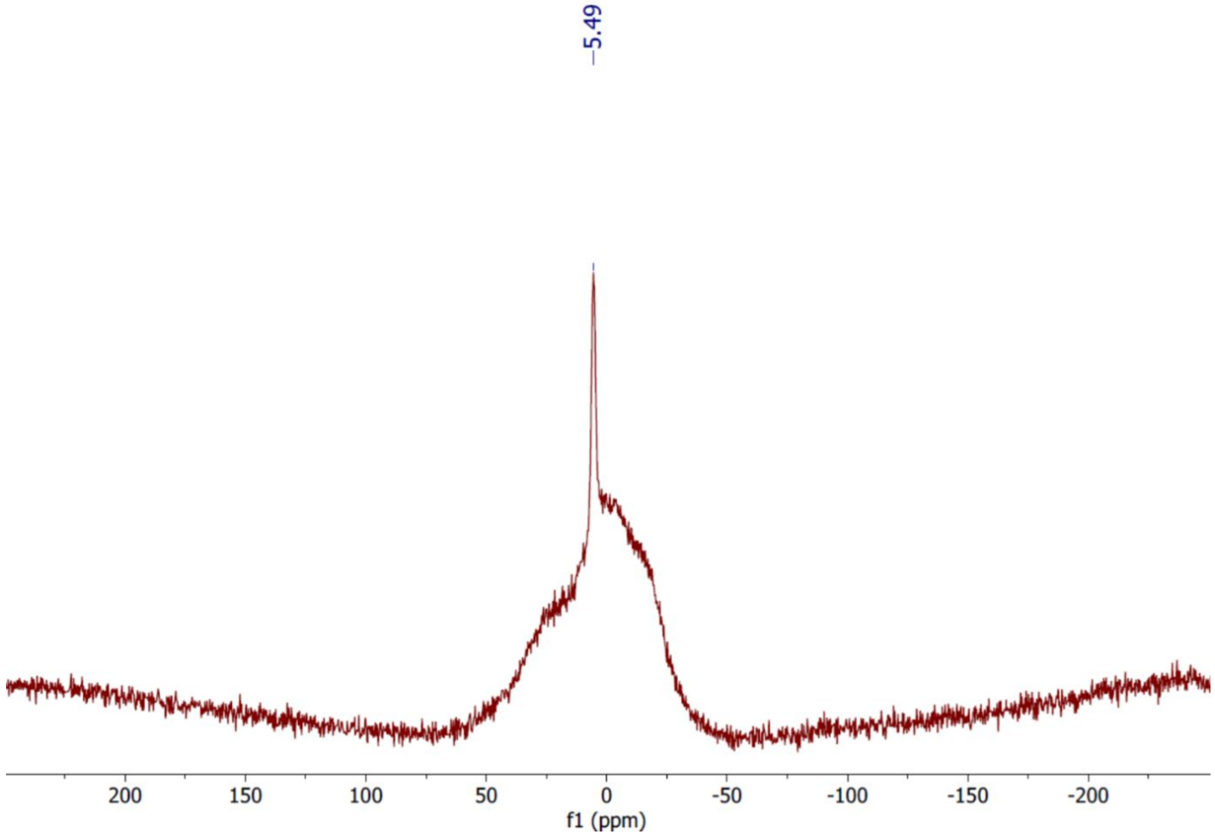


**Figure S 95.** $^{11}$B NMR spectrum of the NMR-scale reaction between [Sm(Tp)$_2$($\mu$-H)$_2$Al(H)(N")] **2-Sm** with 3.7 equivalents of benzophenone in $d_6$-benzene, consistent with consumption of **2-Sm** and formation of [Sm(Tp)$_2$(OCHPh$_2$)] under ambient temperatures.

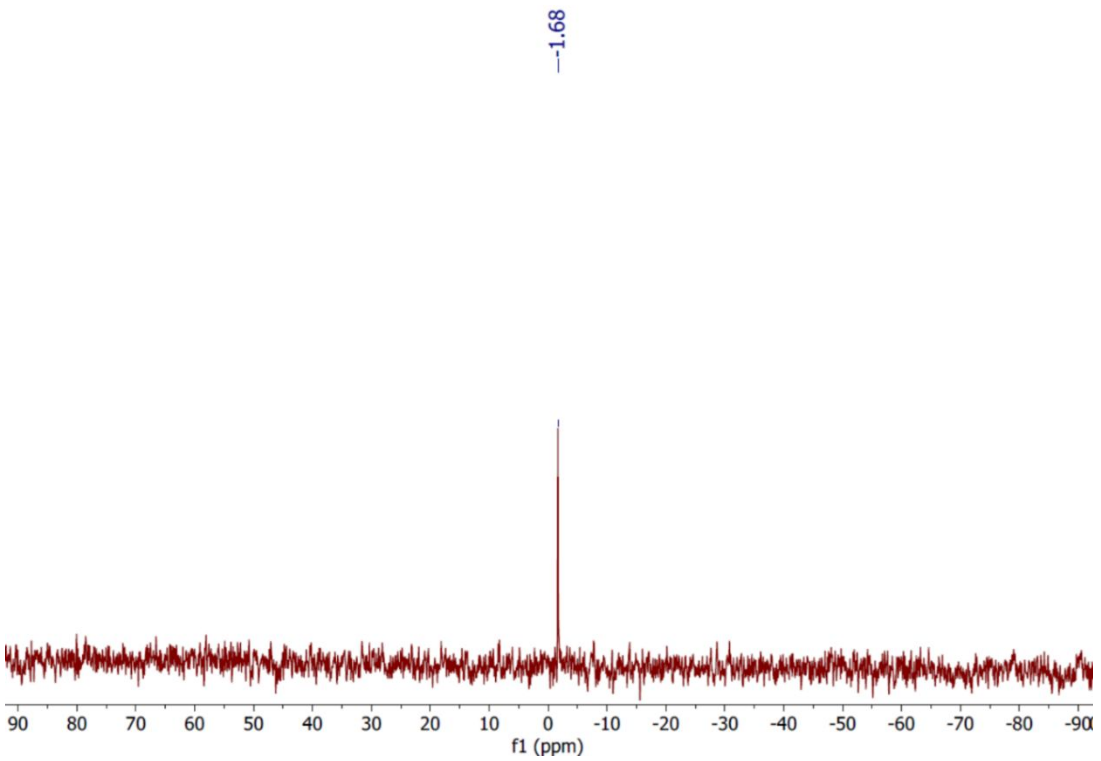


**Figure S 96.** $^{29}Si\{^1H\}$ INEPT NMR spectrum of the NMR-scale reaction between [Sm(Tp)$_2$($\mu$-H)$_2$Al(H)(N")] **2-Sm** with 3.7 equivalents of benzophenone in $d_6$-benzene, consistent with consumption of **2-Sm** and formation of [{Al(N")(H)($\mu$-O(CHPh$_2$))}$_2$] **4-Al** under ambient temperatures.

### B2.17 NMR-scale reaction between [Dy(Tp)$_2$($\mu$-H)$_2$Al(H)(N")] 2-Dy with three equivalents of benzophenone

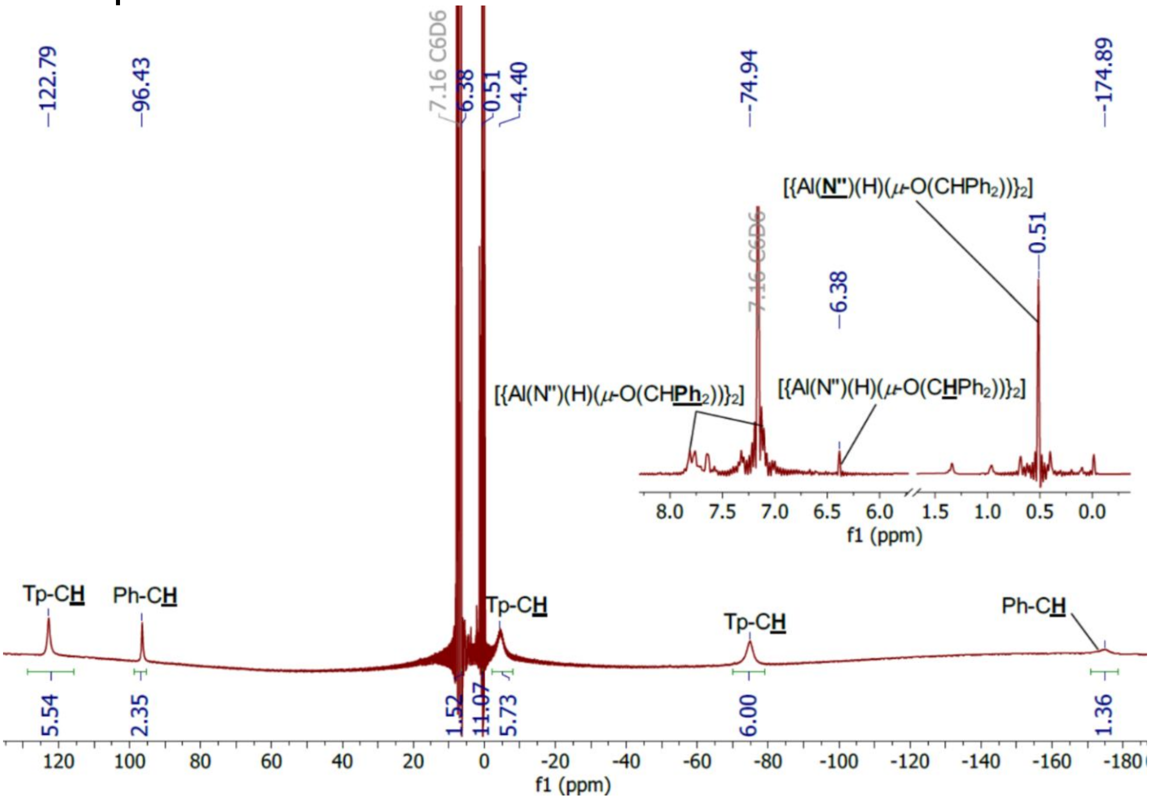


**Figure S 97.** $^1H$ NMR spectrum of the NMR-scale reaction between [Dy(Tp)$_2$($\mu$-H)$_2$Al(H)(N")] **2-Dy** with 3.3 equivalents of benzophenone in $d_6$-benzene, consistent with consumption of **2-Dy**, formation of [Dy(Tp)$_2$(OCHPh$_2$)] and [{Al(N")(H)($\mu$-O(CHPh$_2$))}$_2$] **4-Al** under ambient temperatures. The [Dy(Tp)$_2$(OC**H**Ph$_2$)] resonance was not observed.

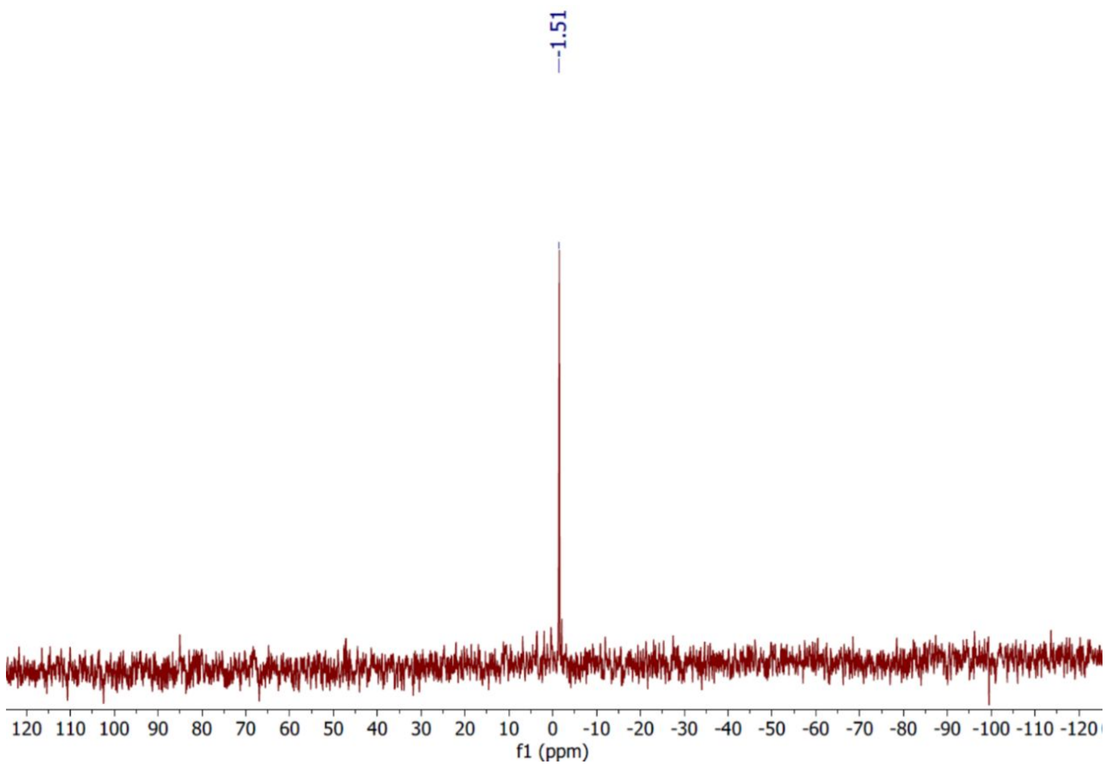


**Figure S 98.** $^{29}Si\{^{1}H\}$ INEPT NMR spectrum of the NMR-scale reaction between $[Dy(Tp)_2(\mu\text{-}H)_2Al(H)(N'')]$ **2-Dy** with 3.3 equivalents of benzophenone in $d_6$-benzene, consistent with formation of $[\{Al(N'')(H)(\mu\text{-}O(CHPh_2))\}_2]$ **4-Al** under ambient temperatures.

**Table S 1.** Complied multinuclear ($^{1}$H, $^{13}$C, $^{19}$F, $^{11}$B, $^{29}$Si) NMR data and assignments of the resonances of [Sm(Tp)$_2$(OTf)] **Sm-OTf**, [Ln(Tp)$_2$(N")] **1-Ln**, [Ln(Tp)$_2$($\mu$-H)$_2$Al(H)(N")] **2-Ln**, and [Ln(Tp)$_2${$\kappa^2$-(Cy)NCHN(Cy)}] **3-Ln** (Ln = Y, Sm, Dy, Yb) complexes.

| Complex | NMR solvent | NMR resonances of the respective nuclei (ppm) | | | | |
|---|---|---|---|---|---|---|
| | | $^{1}$H | | | | |
| | | Tp | | N"-C**H** | Cy-C**H** | $\mu$-**H**$_3$ |
| | | Tp-C**H** | Tp-B**H** | | | |
| **Sm-OTf** | *d*$_3$-MeCN | 4.47, 6.05, 8.88 | 7.22 | - | - | - |
| **1-Y**[1] | *d*$_6$-benzene | 5.78, 7.22, 7.43 | 4.70 | 0.13 | - | - |
| **1-Sm** | *d*$_6$-benzene | 2.23, 5.58, 9.09 | 8.64 | -1.20 | - | - |
| **1-Dy**[2] | *d*$_6$-benzene | -54.84, -30.12, -5.20 | -70.03 | 69.28 | - | - |
| **1-Yb**[1] | *d*$_6$-benzene | -7.76, 8.35, 69.17 | -28.84 | 5.86 | - | - |
| **2-Y** | *d*$_6$-benzene | 5.78, 7.32, 7.39 | 4.70 | 0.25 | - | 5.07 |
| **2-Sm** | *d*$_6$-benzene | 3.96, 5.56, 8.42 | 7.25 | 0.74 | - | 0.24 |
| **2-Dy** | *d*$_6$-benzene | -128.53, -6.77, 40.71 | 104.88 | 10.25 | - | - |
| **2-Yb** | *d*$_6$-benzene | -1.41, 7.60, 40.38 | -14.16 | -5.97 | - | -92.98 |
| **3-Y** | *d*$_6$-benzene | 5.84, 7.26, 7.54 | 4.83 | - | 0.63-1.68, 2.76, 8.28 | |
| **3-Sm** | *d*$_6$-benzene | 4.62, 5.78, 8.39 | 7.16 | - | -0.50 to 4.00, 15.26 | |
| **3-Dy** | *d*$_6$-benzene | -35.51, 17.70, 176.22 | - | - | 13.02, 16.42, 43.51 | - |
| **3-Yb** | *d*$_6$-benzene | -23.98, -7.21, 70.85 | 0.79 | - | -74.71, -44.81, -11.65, -10.43, -6.35 | - |

| Complex | NMR solvent | NMR resonances of the respective nuclei (ppm) | | | | |
|---|---|---|---|---|---|---|
| | | $^{11}$B | $^{13}$C | | $^{29}$Si | $^{19}$F |
| | | Tp-**B** | Tp-**C** | N"-**C** | N"-**Si** | OTf-C**F**$_3$ |
| **Sm-OTf** | *d*$_3$-MeCN | 2.86 | 105.0, 137.8, 142.2 | - | - | -78.67 |
| **1-Y**[1] | *d*$_6$-benzene | -2.96 | 104.5, 135.6, 142.7 | 6.8 | -11.96 | - |
| **1-Sm** | *d*$_6$-benzene | 6.25 | 103.0, 136.5, 141.0 | 5.2 | -1.34 | - |
| **1-Dy**[2] | *d*$_6$-benzene | -192.72 | - | - | 2.30 | - |
| **1-Yb**[1] | *d*$_6$-benzene | -88.39 | - | - | 1.90 | - |
| **2-Y** | *d*$_6$-benzene | -2.87 | 104.9, 135.8, 142.8 | 4.4 | -2.38 | - |
| **2-Sm** | *d*$_6$-benzene | 3.24 | 104.1, 136.3, 141.7 | 4.9 | -1.54 | - |
| **2-Dy** | *d*$_6$-benzene | -27.08 | - | - | - | - |
| **2-Yb** | *d*$_6$-benzene | -48.64 | - | - | -14.67 | - |
| **3-Y** | *d*$_6$-benzene | -2.80 | - | - | - | - |
| **3-Sm** | *d*$_6$-benzene | 2.19 | - | - | - | - |
| **3-Dy** | *d*$_6$-benzene | -123.73 | - | - | - | - |
| **3-Yb** | *d*$_6$-benzene | -11.38 | - | - | - | - |

## B3 Infrared (IR) data

### B3.1 ATR-IR data of $Sm(OTf)_3$, [$Sm(Tp)_2(OTf)$] Sm-OTf, and [$Sm(Tp)_2(N'')$] 1-Sm

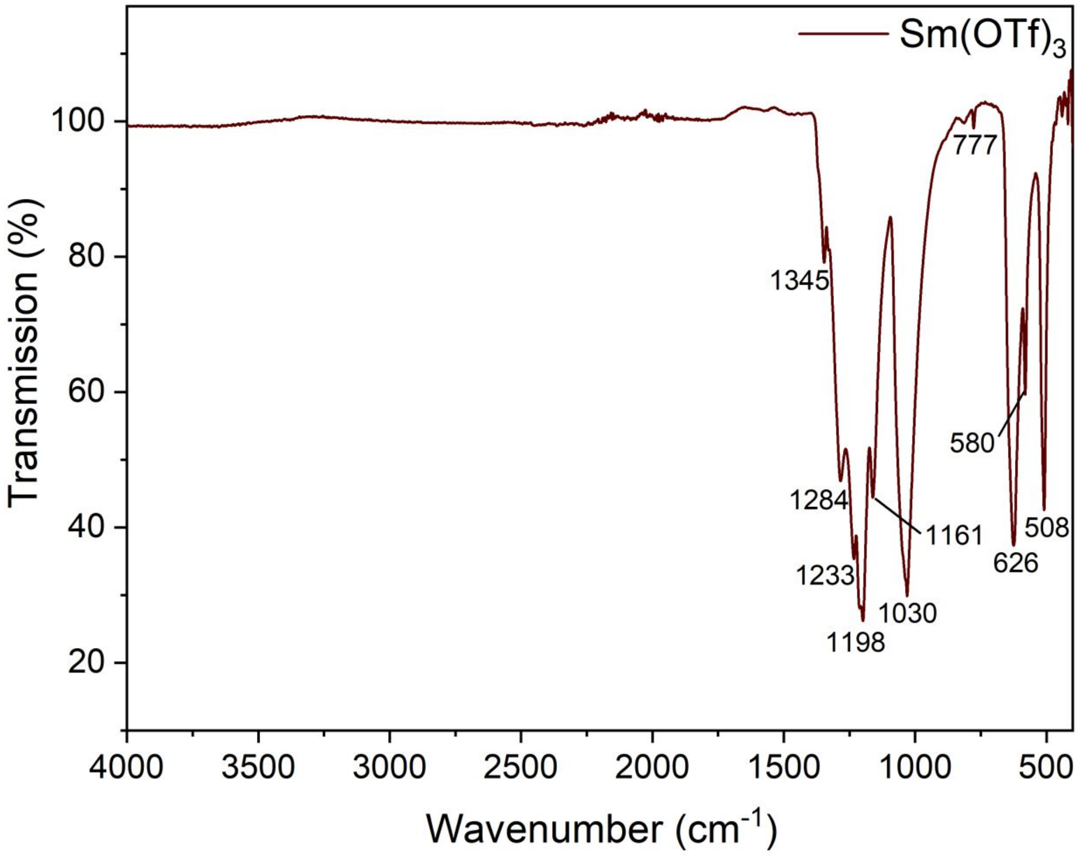


**Figure S 99.** ATR-IR spectrum of $Sm(OTf)_3$.

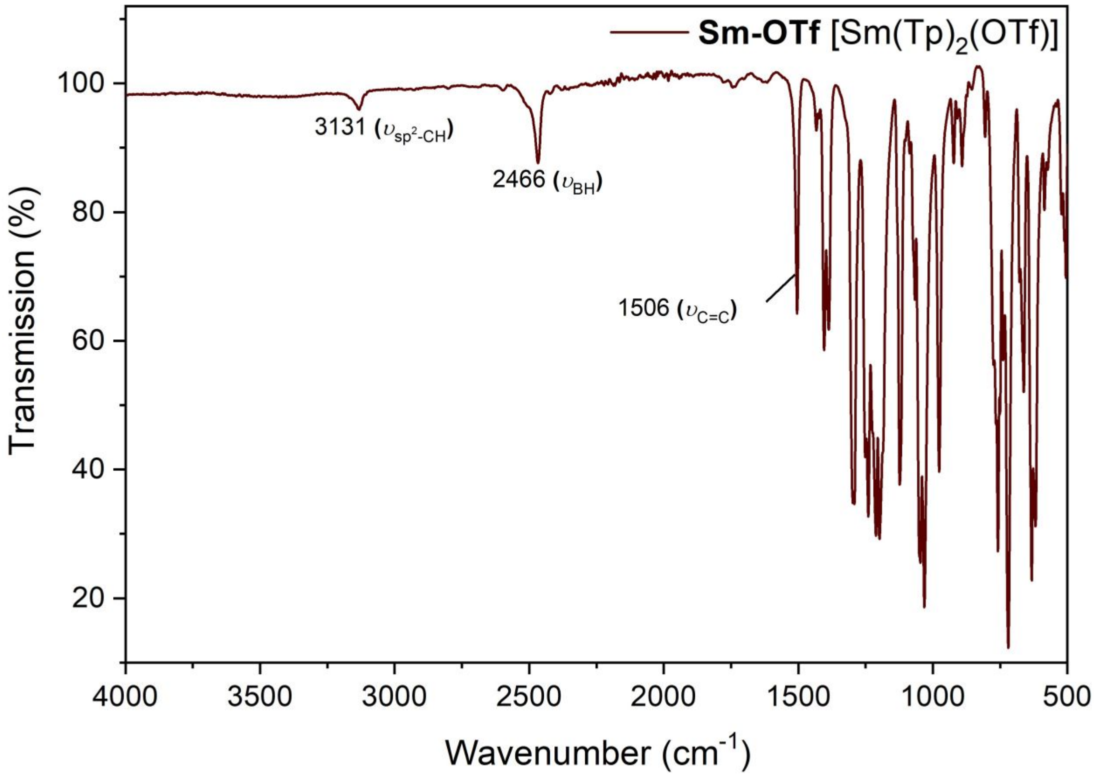


**Figure S 100.** ATR-IR spectrum of [$Sm(Tp)_2(OTf)$] **Sm-OTf**.

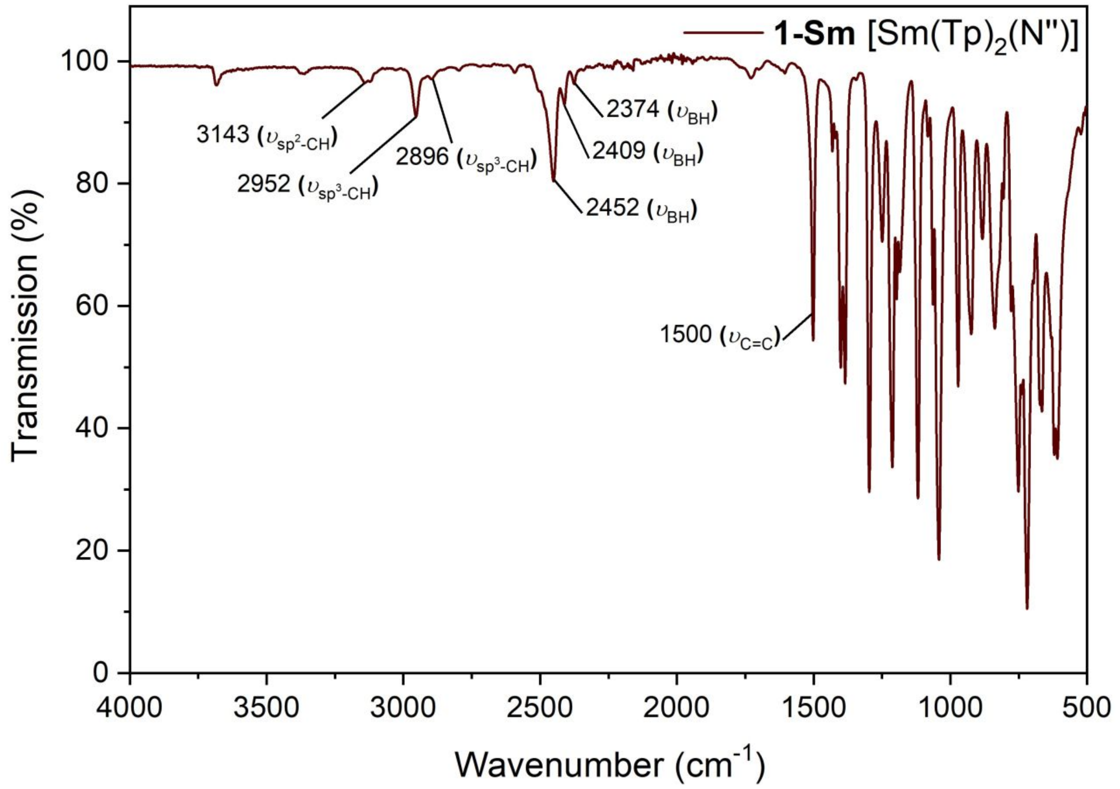


**Figure S 101.** ATR-IR spectrum of [Sm(Tp)$_2$(N")] **1-Sm**.

### B3.2 FTIR data of [Ln(Tp)$_2$($\mu$-H)$_2$Al(H)(N")] 2-Ln (Ln = Y, Sm, Dy, Yb)

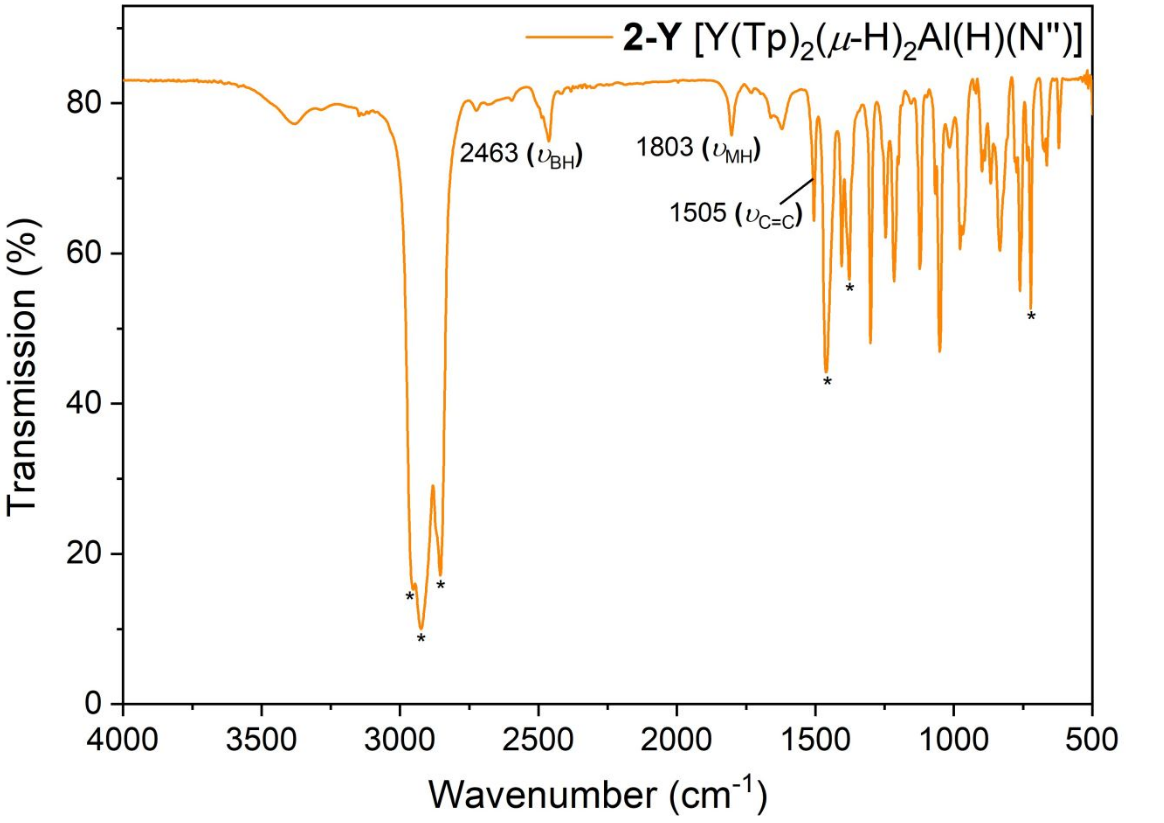


**Figure S 102.** FTIR (nujol mull between NaCl discs) spectrum of [Y(Tp)$_2$($\mu$-H)$_2$Al(H)(N")] **2-Y**. Absorptions due to nujol are denoted with *.

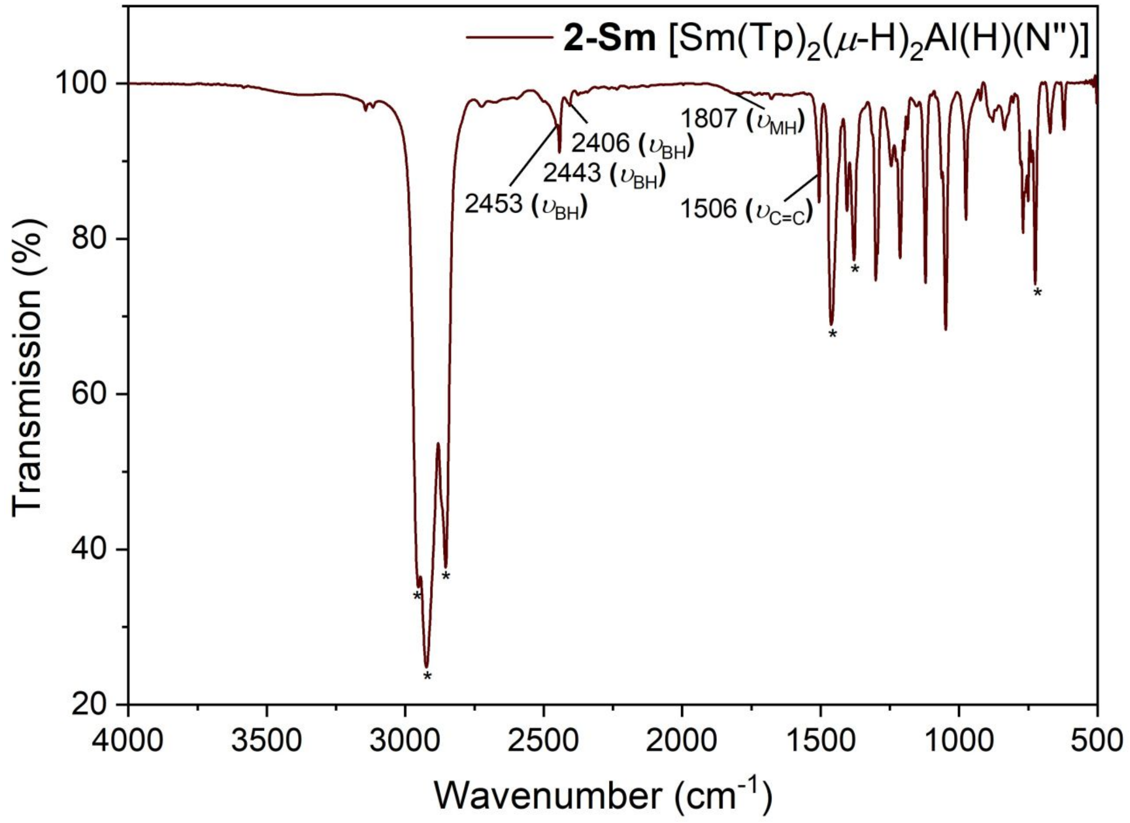


**Figure S 103.** FTIR (nujol mull between NaCl discs) spectrum of [Sm(Tp)$_2$($\mu$-H)$_2$Al(H)(N")] **2-Sm**. Absorptions due to nujol are denoted with *.

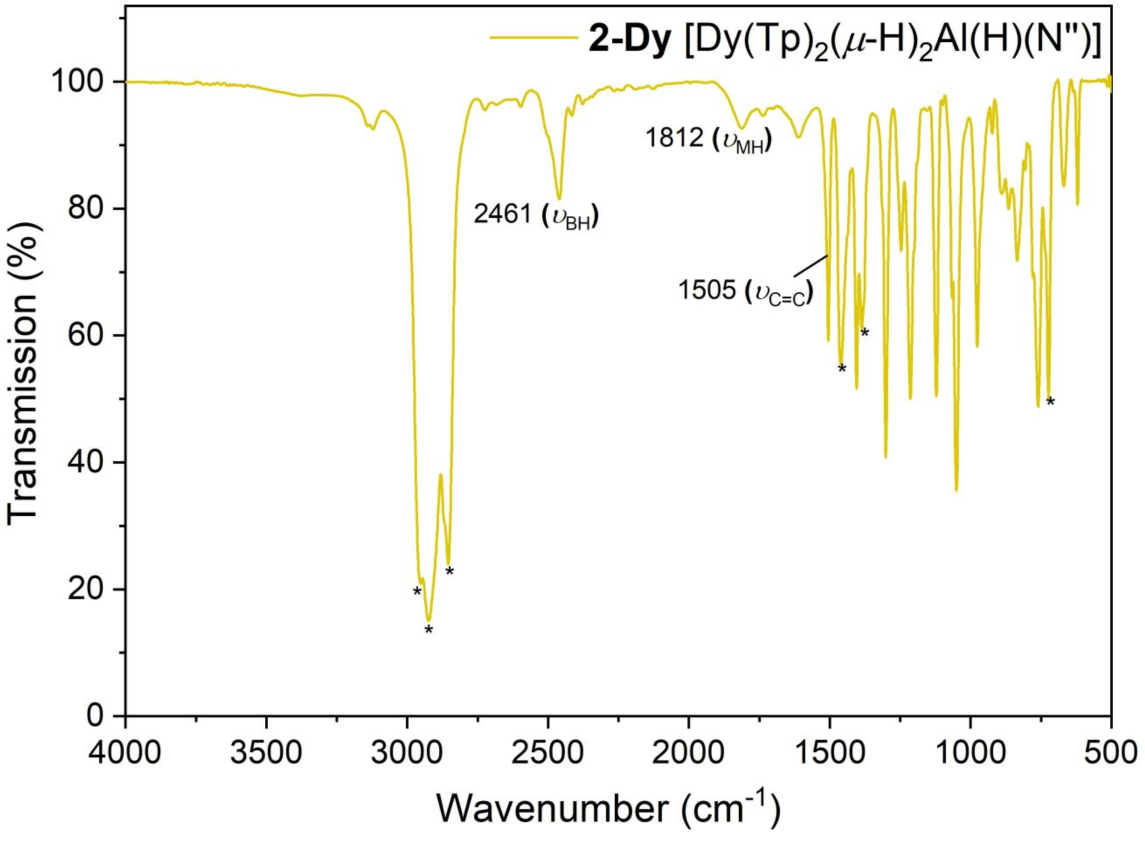


**Figure S 104.** FTIR (nujol mull between NaCl discs) spectrum of [Dy(Tp)$_2$($\mu$-H)$_2$Al(H)(N")] **2-Dy**. Absorptions due to nujol are denoted with *.

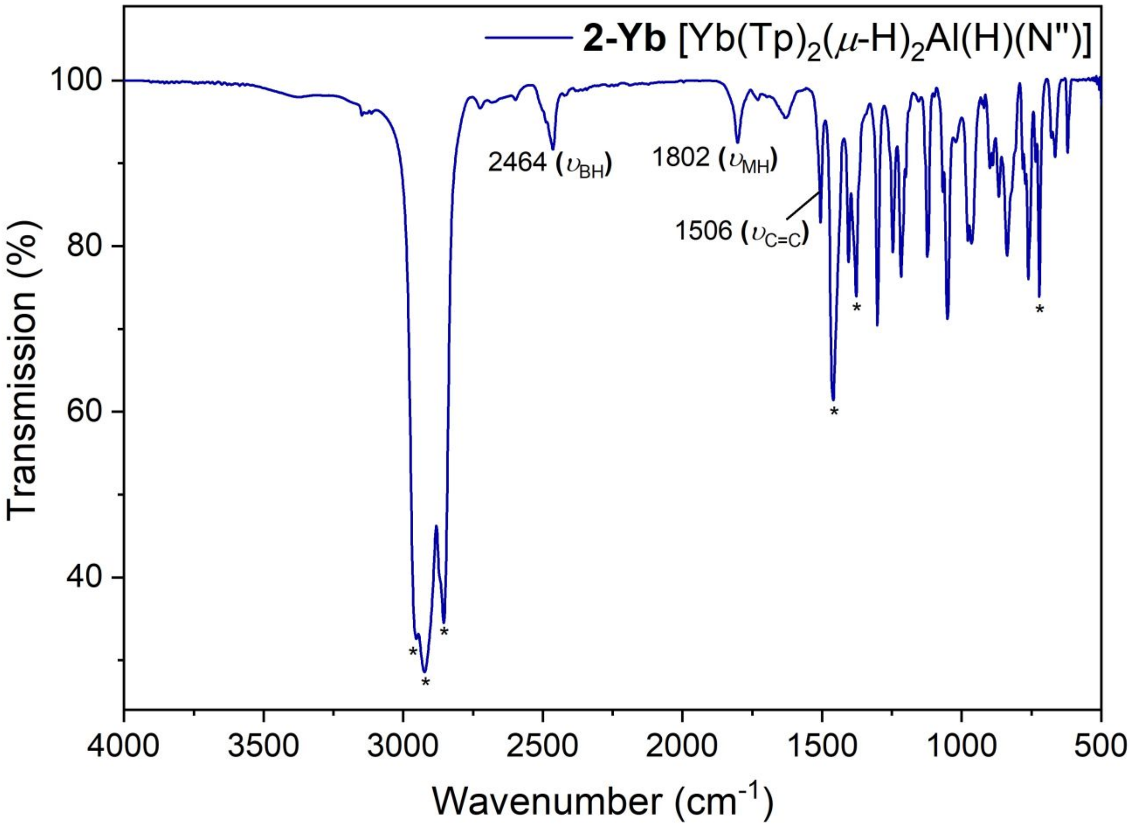


**Figure S 105.** FTIR (nujol mull between NaCl discs) spectrum of [Yb(Tp)$_2$($\mu$-H)$_2$Al(H)(N")] **2-Yb**. Absorptions due to nujol are denoted with *.

### B3.3 FTIR data of [Ln(Tp)$_2${$\kappa^2$-(Cy)NCHN(Cy)}] 3-Ln (Ln = Dy, Yb)

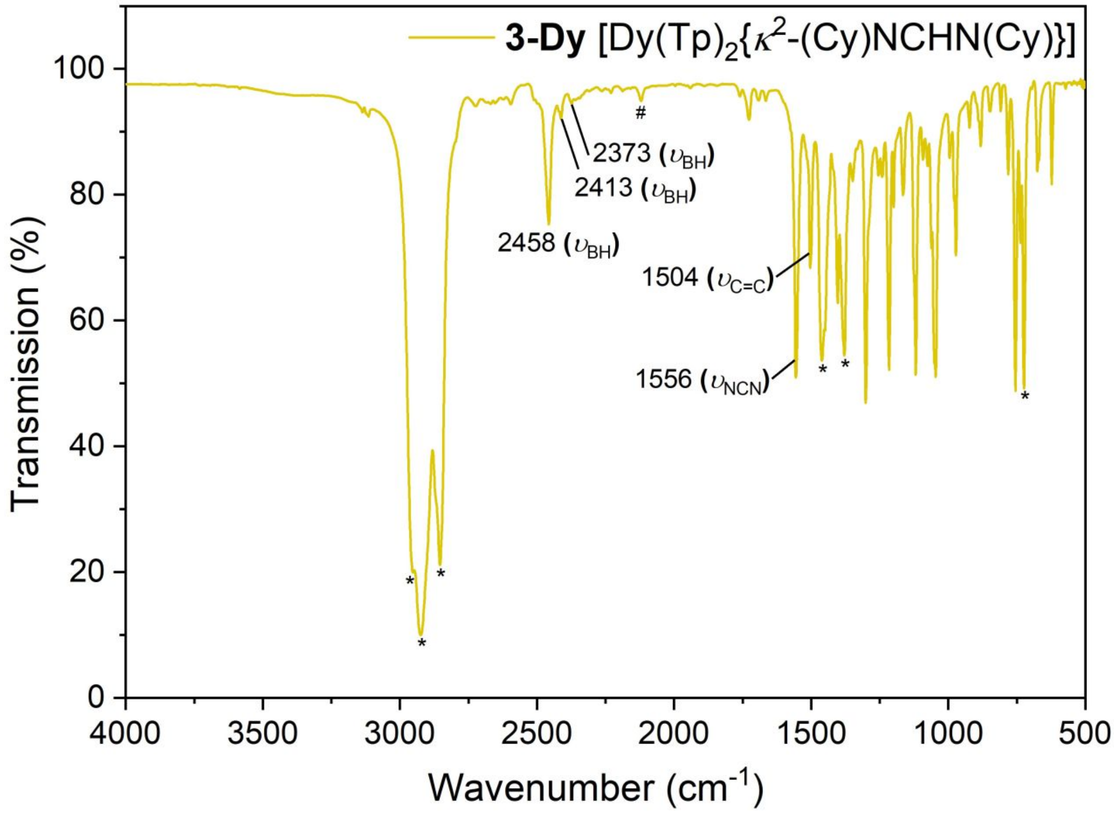


**Figure S 106.** FTIR (nujol mull between NaCl discs) spectrum of [Dy(Tp)$_2${$\kappa^2$-(Cy)NCHN(Cy)}] **3-Dy**. Absorptions due to nujol are denoted with * and absorption at 2120 cm$^{-1}$ ($\upsilon_{NCN}$ of DCC) due to minor adventitious decomposition of **3-Dy** is denoted with $^{\#}$.

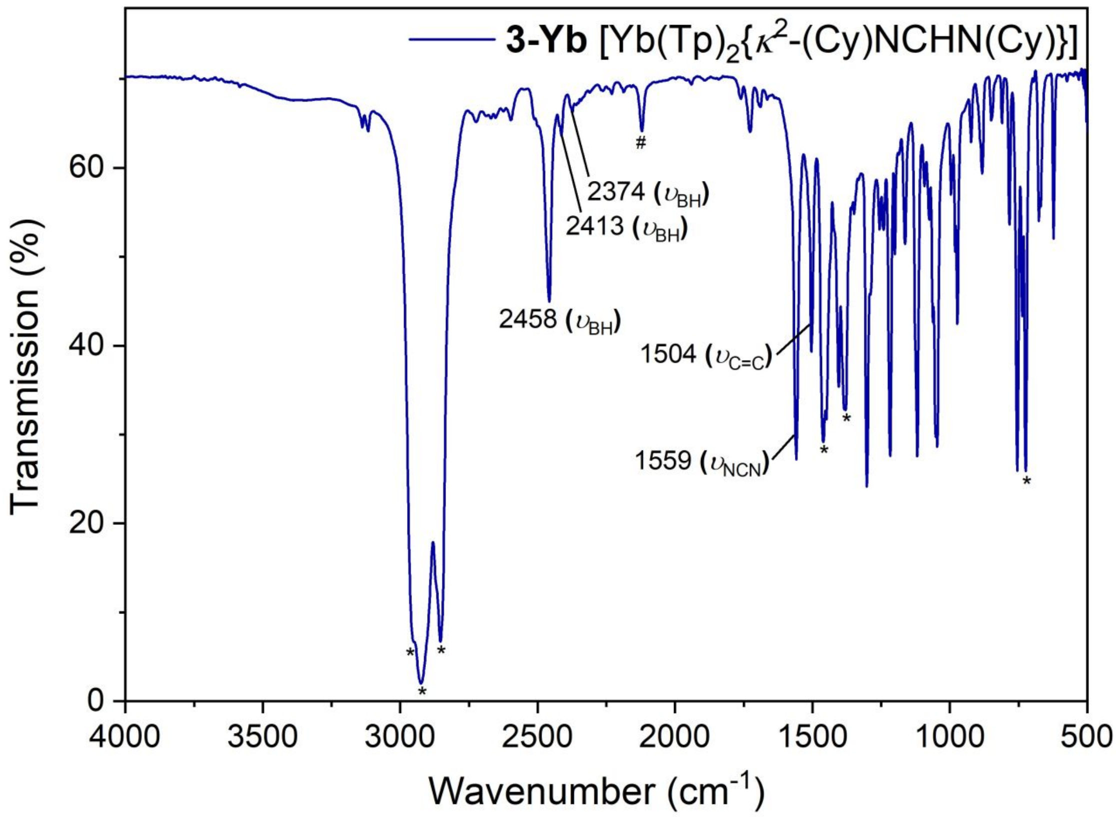


**Figure S 107.** FTIR (nujol mull between NaCl discs) spectrum of [Yb(Tp)$_2${$\kappa^2$-(Cy)NCHN(Cy)}] **3-Yb**. Absorptions due to nujol are denoted with * and absorption at 2120 cm$^{-1}$ ($\upsilon_{NCN}$ of DCC) due to minor adventitious decomposition of **3-Yb** is denoted with $^{\#}$.

## B4 Single-crystal X-Ray diffraction data

**Table S 2**. Selected metrics for the [Ln(Tp)$_2$(OTf)(THF)] **Ln-OTf** (Ln = Sm, Gd, Dy, Yb), [Ln(Tp)$_2$(N'')] **1-Ln** (Ln = Y, Sm, Dy, Yb), [Ln(Tp)$_2$($\mu$-H)$_2$Al(H)(N'')] **2-Ln** (Ln = Y, Dy, Yb), and [Ln(Tp)$_2${$\kappa^2$-(Cy)NCHN(Cy)}] **3-Ln** (Ln = Dy, Yb) complexes.

| Complexes **Ln-OTf** and **1-Ln** | Bond metrics associated with the respective ligands | | | | |
|---|---|---|---|---|---|
| | **Tp** | | **OTf** | **THF** | **N''** |
| | Bond lengths (Å) | Bond angles (°) | Bond lengths (Å) | | |
| | Ln–N(Tp) | B(Tp)–Ln–B(Tp) | Ln–O(OTf) | Ln–O(THF) | Ln–N(N'') |
| **Sm-OTf** | 2.512(2)-2.583(2) | 132.6 | 2.385(2) | 2.5298(19) | - |
| **Gd-OTf**[1] | 2.449(11)-2.592(7) | 127.3 | 2.374(8) | 2.477(9) | - |
| **Dy-OTf**[2] | 2.430(2)-2.520(2) | 130.43 | 2.350(2) | 2.567(2) | - |
| **Yb-OTf**[1] | 2.365(6)-2.489(6) | 126.3-127.8 | 2.296(5) | 2.582(5) | - |
| **1-Y**[1] | 2.3848(19)-2.554(2) | 130.0 | - | - | 2.338(2) |
| **1-Sm** | 2.488(4)-2.603(4) | 126.0 | - | - | 2.377(4) |
| **1-Dy**[2] | 2.393(13)-2.578(12) | 129.7 | - | - | 2.338(11) |
| **1-Yb**[1] | 2.3510(13)-2.5232(13) | 129.9 | - | - | 2.2956(12) |

| Complexes **2-Ln** | Bond metrics associated with the respective ligands | | | | | | |
|---|---|---|---|---|---|---|---|
| | **Tp** | | **Al(H)$_3$(N'')** | | | | |
| | Bond lengths (Å) | Bond angles (°) | Bond lengths (Å) | | | Bond angles (°) | |
| | Ln–N(Tp) | B(Tp)–Ln–B(Tp) | M–H | | Al–N(N'') | H($\mu$-H)–M–HA($\mu$-H) | |
| | | | Ln | Al | | Ln | Al |
| **2-Y** | 2.3882(15)-2.5042(15) | 136.1 | Y1–H 2.31(2), Y1–HA 2.37(2) | Al1–H 1.66(2), Al1–HA 1.59(2), Al1–HB 1.49(3) | 1.8433(15) | 58.9(8) | 90.2(12) |
| **2-Dy** | 2.4011(18)-2.5108(18) | 136.0 | Dy1–H 2.35(3), Dy1–HA 2.36(3) | Al1–H 1.60(3), Al1–HA 1.56(3), Al1–HB 1.51(3) | 1.8430(19) | 56.8(9) | 90.5(14) |
| **2-Yb** | 2.348(2)-2.467(2) | 136.3 | Yb1–H 2.39(4), Yb1–HA 2.25(4) | Al1–H 1.66(4), Al1–HA 1.59(4), Al1–HB 1.62(4) | 1.844(3) | 60.1(14) | 91(2) |

| Complexes **3-Ln** | Bond metrics associated with the respective ligands | | | | | |
|---|---|---|---|---|---|---|
| | **Tp** | | **(Cy)NCHN(Cy)** | | | |
| | Bond lengths (Å) | Bond angles (°) | Bond lengths (Å) | | Bond angles (°) | |
| | Ln–N(Tp) | B(Tp)–Ln–B(Tp) | Ln–N7 or Ln–N7' | C10–N7 or C10–N7' | N7–Ln–N7' | N7–C10–N7' |
| **3-Dy** | 2.414(9)-2.521(9) | 132.3 | 2.376(8) | 1.326(14) | 57.4(5) | 118.6(15) |
| **3-Yb** | 2.408(4)-2.515(3) | 132.6 | 2.373(3) | 1.315(5) | 57.3(2) | 119.8(6) |

**Table S 3.** Crystallographic data table for complexes [Sm(Tp)$_2$(OTf)(THF)] **Sm-OTf**, [Sm(Tp)$_2$(N")] **1-Sm**, [Ln(Tp)$_2$($\mu$-H)$_2$Al(H)(N")] **2-Ln** (Ln = Y, Dy, Yb), [Ln(Tp)$_2${$\kappa^2$-(Cy)NCHN(Cy)}] **3-Ln** (Ln = Dy, Yb), [(IDipp)Al(N")(H)$_2$] **Al-IDipp**, and [{Al(N")(H)($\mu$-O(CHPh$_2$))}$_2$] **4-Al** complexes.

| **CCDC No.** | **2313687** | **2313688** | **2313689** |
|---|---|---|---|
| **Parameter** | **Sm-OTf** | **1-Sm** | **2-Y** |
| Formula | $C_{23}H_{28}B_2F_3N_{12}O_4SSm$ | $C_{24}H_{38}B_2N_{13}Si_2Sm$ | $C_{24}H_{41}AlB_2N_{13}Si_2Y$ |
| *F*w | 797.60 | 736.82 | 705.39 |
| Colour and shape | Lath, colourless | Plate, colourless | Block, colourless |
| Dimensions (mm) | 0.21 × 0.07 × 0.02 | 0.16 × 0.06 × 0.02 | 0.20 × 0.11 × 0.08 |
| Crystal System | Monoclinic | Monoclinic | Monoclinic |
| Space group | $P2_1/n$ | $P2_1/n$ | $P2_1/n$ |
| *a* (Å) | 12.8192(9) | 10.6729(7) | 13.5131(1) |
| *b* (Å) | 19.9394(13) | 23.8746(15) | 14.5943(1) |
| *c* (Å) | 12.8857(10) | 13.1692(8) | 18.2962(2) |
| $\alpha$ (°) | 90 | 90 | 90 |
| $\beta$ (°) | 102.564(3) | 100.422(2) | 104.461(1) |
| $\gamma$ (°) | 90 | 90 | 90 |
| *V* (Å$^3$) | 3214.8(4) | 3300.3(4) | 3493.95(5) |
| *Z* | 4 | 4 | 4 |
| $D_x$ (Mg m$^{-3}$) | 1.648 | 1.483 | 1.341 |
| No. of reflections measured | 38925 | 34102 | 25476 |
| No. of independent reflections | 7970 | 6767 | 6972 |
| No. of reflections with $I > 2\sigma(I)$ | 6632 | 5519 | 6420 |
| $R_{int}$ | 0.0569 | 0.0588 | 0.0395 |
| $R_1$ ($I > 2\sigma$)[a] | 0.0312 | 0.0467 | 0.0292 |
| $wR_2(F^2)$[b] | 0.0754 | 0.1070 | 0.0785 |
| *GoF*[c] | 1.049 | 1.104 | 1.052 |

| **CCDC No.** | **2313690** | **2313691** | **2313692** |
|---|---|---|---|
| **Parameter** | **2-Dy** | **2-Yb** | **3-Dy** |
| Formula | $C_{24}H_{41}AlB_2N_{13}Si_2Dy$ | $C_{24}H_{41}AlB_2N_{13}Si_2Yb$ | $C_{31}H_{43}B_2N_{14}Dy$ |
| *F*w | 778.98 | 789.52 | 795.91 |
| Colour and shape | Block, colourless | Block, colourless | Block, yellow |
| Dimensions (mm) | 0.29 × 0.12 × 0.08 | 0.10 × 0.06 × 0.03 | 0.29 × 0.20 × 0.06 |
| Crystal System | Monoclinic | Monoclinic | Trigonal |
| Space group | $P2_1/n$ | $P2_1/n$ | $P3_221$ |
| *a* (Å) | 13.4960(1) | 13.4843(1) | 9.8603(1) |
| *b* (Å) | 14.5771(1) | 14.5826(1) | 9.8603(1) |
| *c* (Å) | 18.3075(1) | 18.2409(2) | 30.7190(2) |
| $\alpha$ (°) | 90 | 90 | 90 |
| $\beta$ (°) | 104.559(1) | 104.349(1) | 90 |
| $\gamma$ (°) | 90 | 90 | 120 |
| *V* (Å$^3$) | 3486.03(4) | 3474.93(5) | 2586.53(6) |
| *Z* | 4 | 4 | 3 |
| $D_x$ (Mg m$^{-3}$) | 1.484 | 1.509 | 1.533 |
| No. of reflections measured | 37288 | 71008 | 28519* |
| No. of independent reflections | 6953 | 6946 | 28519* |
| No. of reflections with $I > 2\sigma(I)$ | 6601 | 6386 | 28426 |

| | | | |
|---|---|---|---|
| $R_{int}$ | 0.0427 | 0.0847 | 0.0600* |
| $R_1$ ($I > 2\sigma$)[a] | 0.0247 | 0.0333 | 0.0482 |
| $wR_2(F^2)$[b] | 0.0624 | 0.0888 | 0.1277 |
| *GoF*[c] | 1.030 | 1.052 | 1.060 |
| **CCDC No.** | **2313693** | **2313694** | **2313695** |
| **Parameter** | **3-Yb** | **Al-IDipp** | **4-Al** |
| Formula | $C_{31}H_{43}B_2N_{14}Yb$ | $C_{33}H_{56}AlN_3Si_2$•$C_6H_{14}$ | $C_{38}H_{60}Al_2N_2O_2Si_4$ |
| *F*w | 806.45 | 664.14 | 743.20 |
| Colour and shape | Block, yellow | Block, colourless | Block, colourless |
| Dimensions (mm) | 0.20 × 0.12 × 0.06 | 0.32 × 0.18 × 0.06 | 0.15 × 0.10 × 0.04 |
| Crystal System | Trigonal | Monoclinic | Triclinic |
| Space group | $P3_221$ | $P2_1/c$ | $P\bar{1}$ |
| *a* (Å) | 9.8605(1) | 12.4686(6) | 9.1536(2) |
| *b* (Å) | 9.8605(1) | 14.0568(7) | 12.4748(3) |
| *c* (Å) | 30.7296(3) | 25.3326(12) | 20.0086(3) |
| $\alpha$ (°) | 90 | 90 | 80.228(2) |
| $\beta$ (°) | 90 | 101.907(2) | 88.074(2) |
| $\gamma$ (°) | 120 | 90 | 74.123(2) |
| $V$ (Å$^3$) | 2587.53(6) | 4344.5(4) | 2165.52(8) |
| *Z* | 3 | 4 | 2 |
| $D_x$ (Mg m$^{-3}$) | 1.553 | 1.015 | 1.140 |
| No. of reflections measured | 33881 | 81656 | 43762 |
| No. of independent reflections | 3435 | 10778 | 8650 |
| No. of reflections with $I > 2\sigma(I)$ | 3430 | 7825 | 7291 |
| $R_{int}$ | 0.0618 | 0.0726 | 0.0491 |
| $R_1$ ($I > 2\sigma$)[a] | 0.0255 | 0.0508 | 0.0453 |
| $wR_2(F^2)$[b] | 0.0617 | 0.1429 | 0.1265 |
| *GoF*[c] | 1.131 | 1.004 | 1.080 |

[a] $R_1 = \Sigma(||F_0| - |F_c||) / \Sigma|F_0|$, $F_0 > 4\sigma(F_0)$.
[b] $wR_2 = \{\Sigma[w(F_0^2 - F_c^2)^2 / \Sigma[w(F_0^2)^2]\}^{1/2}$.
[c] $GOF = [\Sigma w (F_0^2 - F_c^2)^2 / (n_0 - n_p)]^{1/2}$.
* Twinned structure refined against hklf 5 formatted reflection file.

## B4.1 [Sm(Tp)$_2$(OTf)] Sm-OTf and [Sm(Tp)$_2$(N'')] 1-Sm

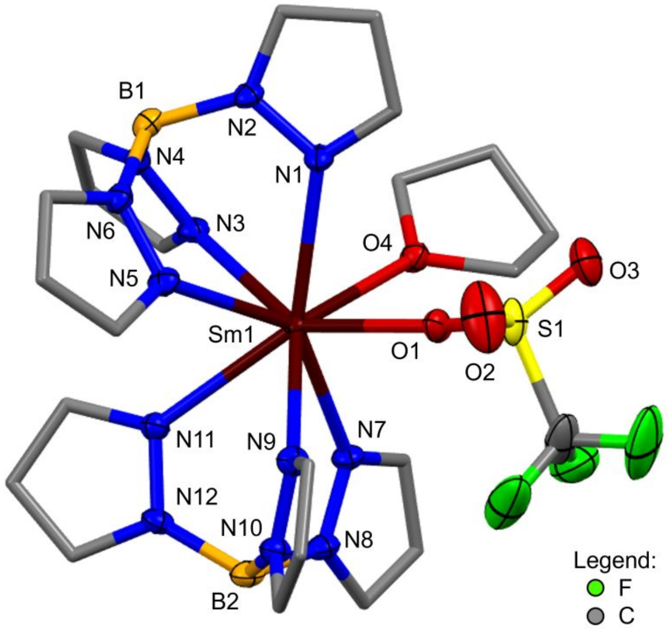


**Figure S 108.** Molecular structure of **Sm-OTf**.

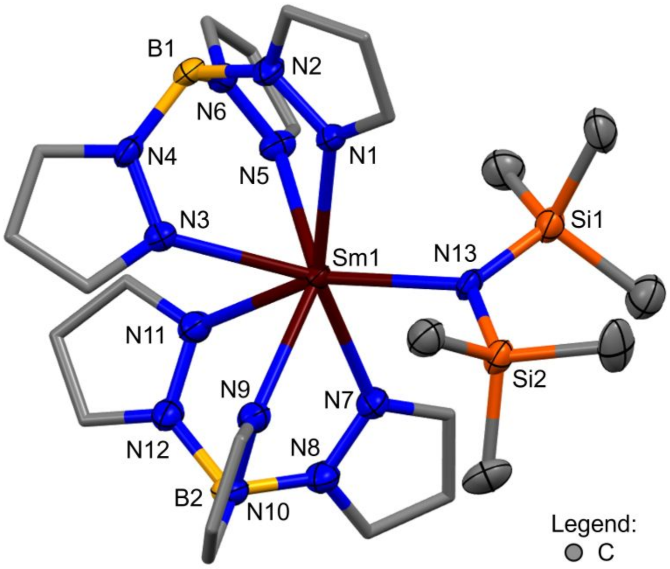


**Figure S 109.** Molecular structure of **1-Sm**.

## B4.2 [Ln(Tp)$_2$($\mu$-H)$_2$Al(H)(N'')] 2-Ln (Ln = Y, Dy, Yb)

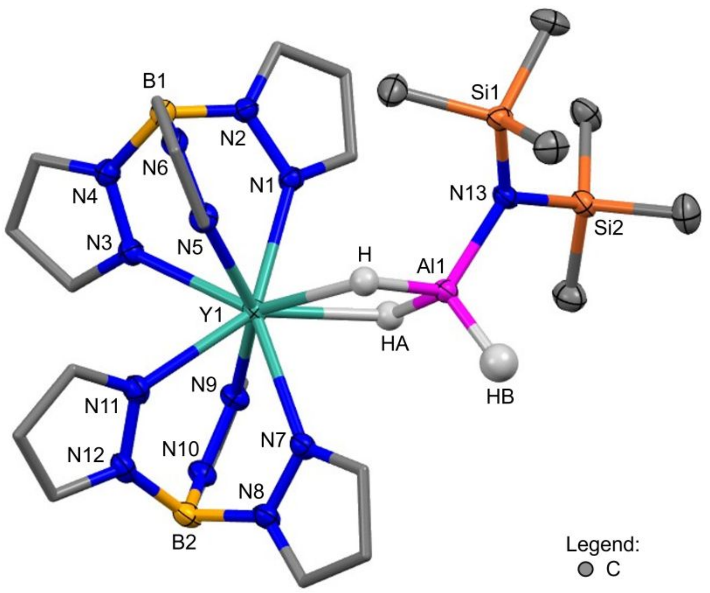


**Figure S 110.** Molecular structure of **2-Y**.

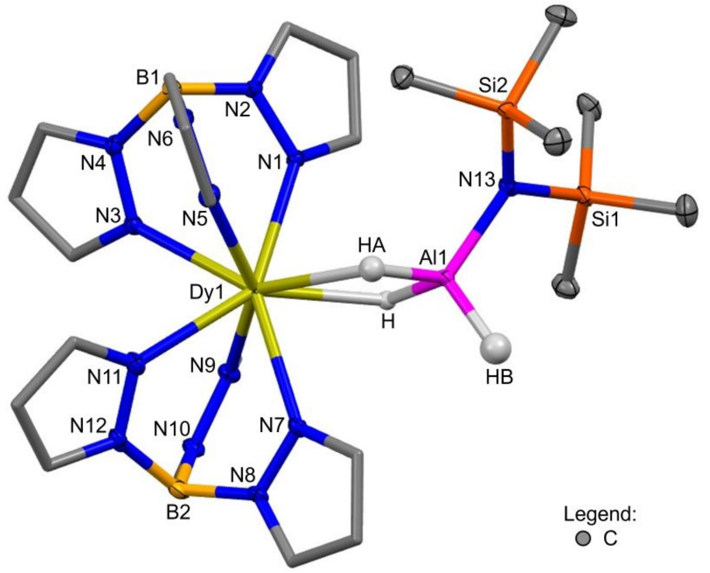


**Figure S 111.** Molecular structure of **2-Dy**.

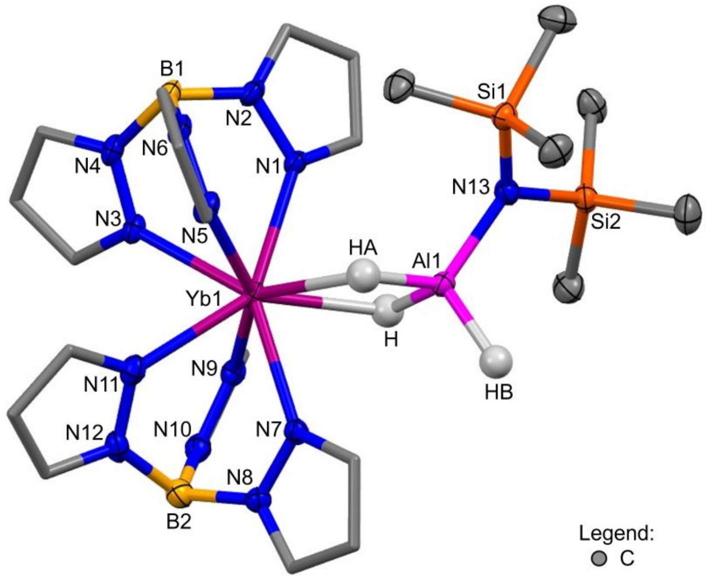


**Figure S 112.** Molecular structure of **2-Yb**.

### B4.3 [Ln(Tp)$_2${$\kappa^2$-(Cy)NCHN(Cy)}] 3-Ln (Ln = Dy, Yb)

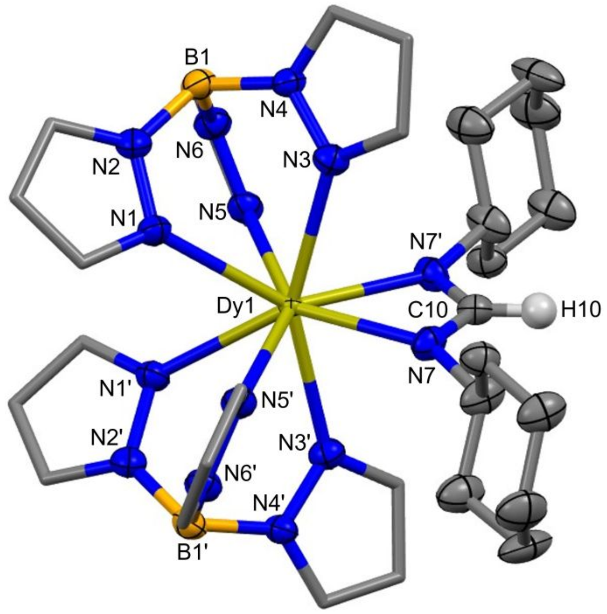


**Figure S 113.** Molecular structure of **3-Dy**. The molecule crystallises with the Dy lying on a 2-fold rotation axis and the asymmetric unit contains one Tp and half a DCC-H; 'prime' atom labels denote symmetry equivalent (1+x-y, 2-y, 4/3-z).

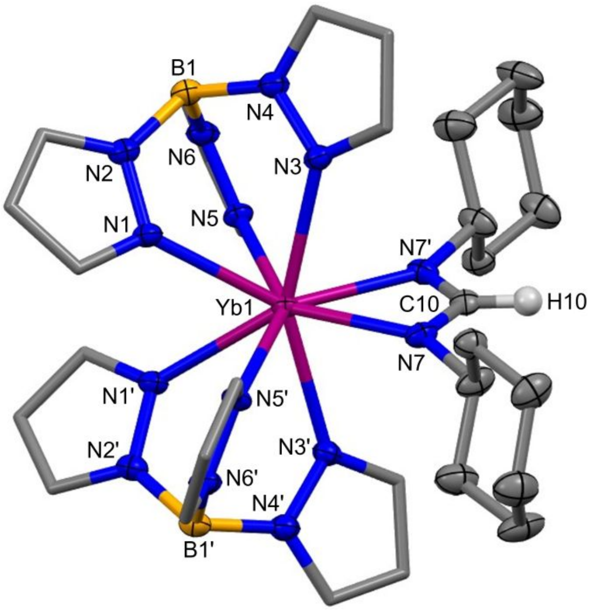


**Figure S 114.** Molecular structure of **3-Yb**. The molecule crystallises with the Yb lying on a 2-fold rotation axis and the asymmetric unit contains one Tp and half a DCC-H; 'prime' atom labels denote symmetry equivalent (1+x-y, 2-y, 4/3-z).

**B4.4 [(IDipp)Al(N'')(H)$_2$] Al-IDipp and [{Al(N'')(H)($\mu$-O(CHPh$_2$))}$_2$] 4-Al**

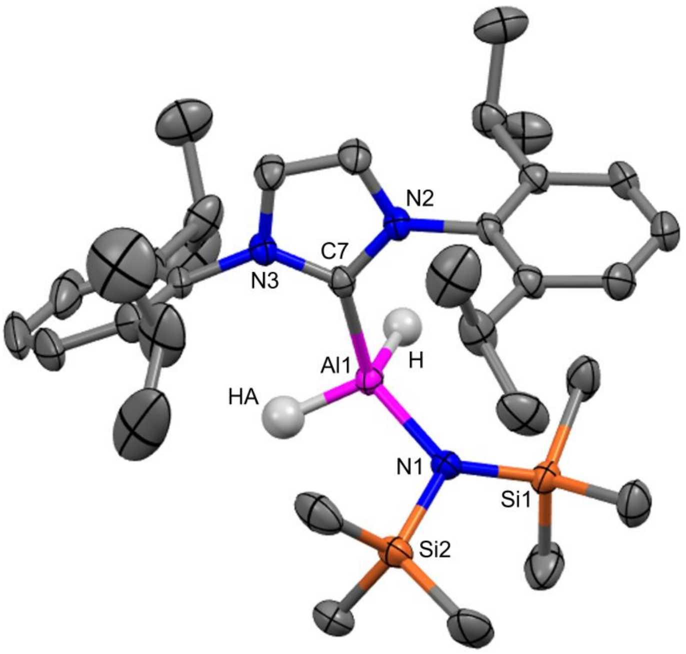


**Figure S 115.** Molecular structure of **Al-IDipp**. Selected bond distances: Al1–H 1.53(2) Å, Al1–HA 1.49(2) Å, Al1–N1 1.8664(14) Å, Al1–C7 2.0860(16) Å; selected bond angles: H–Al1–HA 112.8(11)°, N1–Al1–C7 117.81(6)°.

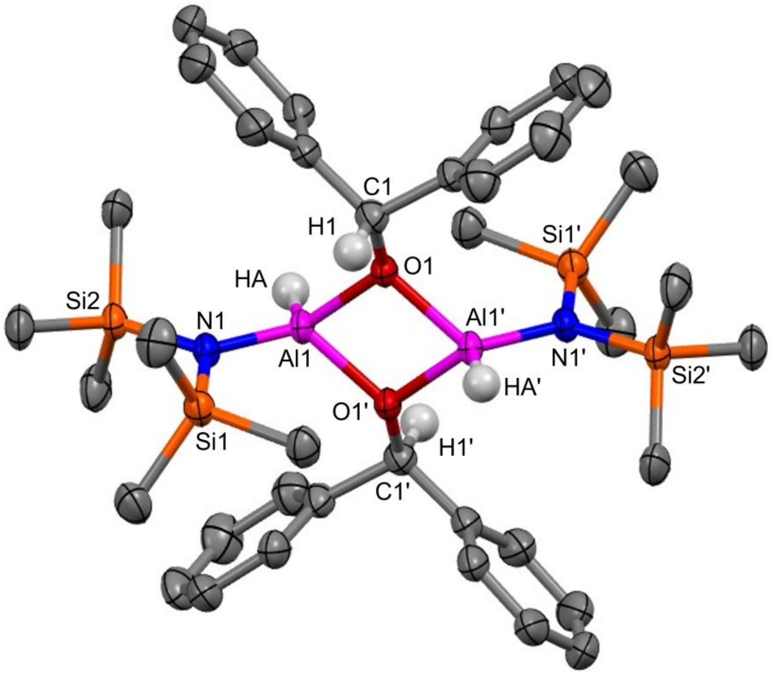


**Figure S 116.** Molecular structure of **4-Al**, showing one of the two crystallographically independent units. Selected bond distances: Al1–O1 1.8631(14) Å, Al1–O1' 1.8576(14) Å, Al1–N1 1.8097(18) Å, Al1–HA 1.57(3) Å, C1–O1 1.454(3) Å; selected bond angles: A1–O1–Al1' 99.66(7)°, O1–Al1–O1' 80.34(7)°; 'prime' atom labels denote symmetry equivalent (-x, 2-y, 1-z). Each crystallographically independent dimeric molecule crystallises across an inversion centre with one crystallographically independent Al site. The action of the inversion symmetry completes each dimer.

## C. Catalytic dehydrocoupling of dimethylaminoborane [$Me_2HN{\bullet}BH_3$] by [Ln(Tp)$_2$($\mu$-H)$_2$Al(H)(N")] 2-Ln (Ln = Y, Sm, Dy, Yb) at ambient temperature

### C1 Methodology for catalytic runs

*General procedure for catalytic reactions:* 10 mol% of the bimetallic precatalyst [Ln(Tp)$_2$($\mu$-H)$_2$Al(H)(N")] (**2-Ln**, Ln = Y, Sm, Dy, Yb; 2.9 $\mu$mol) and [$Me_2HN{\bullet}BH_3$] (28.9 $\mu$mol) were dissolved in 0.5 mL of $d_6$-benzene and added to a JY NMR tube. The catalytic reactions were kept at ambient temperature and $^1$H and $^{11}$B NMR were used to monitor the reaction over time until >80% consumption of [$Me_2HN{\bullet}BH_3$].

For ease in performing reactions in triplicate, a solution of each bimetallic precatalyst was prepared. **2-Ln** (Ln = Y, 6.0 mg; Sm, 6.5 mg; Dy, 6.6 mg; Yb, 6.7 mg; 8.47-8.51 mmol each) in $d_6$-benzene (1.5 mL) to achieve concentrations of 5.67 mmol L$^{-1}$. 0.5 mL of each solution was then used for each catalytic run.

*General procedure for control reactions:* 10 mol% of the starting component ([$Me_3N{\bullet}AlH_3$] or [Yb(Tp)$_2$(N")] **1-Yb**, 2.9 $\mu$mol) and [$Me_2HN{\bullet}BH_3$] (28.9 $\mu$mol) were dissolved in 0.5 mL of $d_6$-benzene and added to a JY NMR tube. The catalytic reactions were kept at ambient temperature for 18 hours, upon which time $^1$H and $^{11}$B NMR were used to assess the relative consumption of [$Me_2HN{\bullet}BH_3$].

### C2 Data for catalytic runs

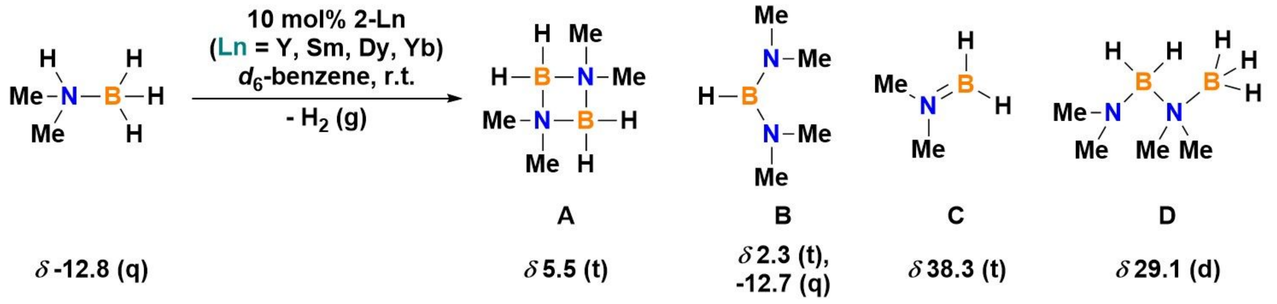


**Scheme 1.** Products of [$Me_2HN{\bullet}BH_3$] dehydrocoupling by [Ln(Tp)$_2$($\mu$-H)$_2$Al(H)(N")] **2-Ln** and their respective chemical shifts (in ppm) in the $^{11}$B NMR in $d_6$-benzene with multiplicities in brackets.

**Table S 4.** Catalytic dehydrocoupling reactions of [$Me_2HN{\bullet}BH_3$] (28.9 $\mu$mol) with 10 mol% [Ln(Tp)$_2$($\mu$-H)$_2$Al(H)(N")] **2-Ln** (Ln = Y, Sm, Dy, Yb), [Yb(Tp)$_2$(N")] **1-Yb**, and [$Me_3N{\bullet}AlH_3$] in $d_6$-benzene (0.5 mL). The table below is the average data of the catalytic reactions that were performed in triplicate.

| Catalyst | Time / hr | Conversion[a] / % | TOF[b] / hr$^{-1}$ | Product distribution[c] | | | | |
|---|---|---|---|---|---|---|---|---|
| | | | | A | B | C | D | Others |
| **2-Y** | 48 | 90 | 0.19 | 61 | 4 | 2 | 5 | 18 |
| **2-Sm** | 172 | 85 | 0.05 | 78 | 2 | - | 4 | 1 |
| **2-Dy** | 60 | 91 | 0.15 | 69 | 8 | 2 | 9 | 3 |
| **2-Yb** | 24 | 93 | 0.39 | 66 | 16 | 4 | 6 | 1 |
| **1-Yb** | 18 | >99 | 0.56 | 98 | - | 2 | - | - |
| [$Me_3N{\bullet}AlH_3$] | 18 | 0 | 0 | 0 | - | - | - | - |

Note: Owing to the poor solubility of **2-Sm** in $d_6$-benzene, the compound precipitated out of solution in all three catalytic experimental runs involving **2-Sm** and settled at the bottom of the JY NMR tube at *ca* 12 h and was analysed as such without agitating the NMR tubes.

[a]Values for the conversion (%) (or consumption of starting material [$Me_2HN$•$BH_3$]) at respective reaction progress times is calculated by subtracting the percentage of starting material from 100%.
[b]Turnover frequency or TOF ($h^{-1}$) is calculated for data points by utilising the formula TOF = Conversion/(Catalyst loading × time).
[c]Relative ratios of distribution of products (%) is calculated by dividing the integral of that respective product by the summation of the integrals of all the dehydrocoupled products and starting material [$Me_2HN$•$BH_3$] and expressed as a percentage (see example $^{11}B$ NMR data below in **Figure S 117** for **2-Y**, Exp 1, t = 48 h). Note that the integration of the Tp-**B** resonance is not considered when calculating the relative product distribution percentages in all cases (and therefore not integrated as also shown in **Figure S 117**).

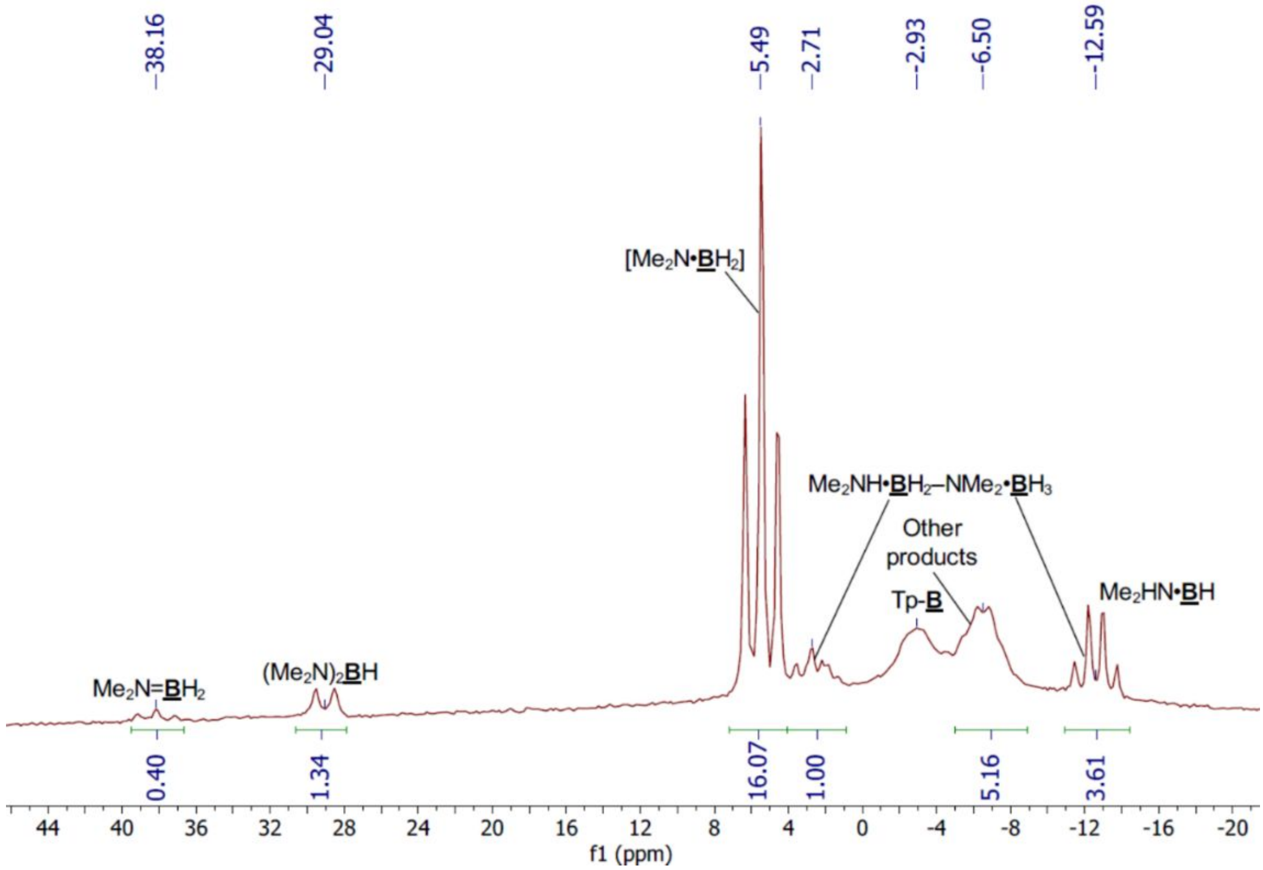


**Figure S 117.** Representative $^{11}B$ NMR spectrum showing the average product distribution of [$Me_2HN$•$BH_3$] dehydrocoupling by [Y(Tp)$_2$($\mu$-H)$_2$Al(H)(N")] **2-Y** (10 mol%) in $d_6$-benzene (Exp 1, t = 48 h).

## D. References


(1) Chowdhury, T.; Horsewill, S. J.; Wilson, C.; Farnaby, J. H. Heteroleptic lanthanide(III) complexes: synthetic utility and versatility of the unsubstituted *bis*-scorpionate ligand framework. *Aust. J. Chem.* **2022**, *75* (9), 660-675. DOI: 10.1071/CH21313.

(2) Chowdhury, T.; Wilson, C.; Maichle-Mössmer, C.; Anwander, R.; Farnaby, J. H. Lanthanide Amide Complexes Supported by the *Bis*-tris(pyrazolyl)borate Ligand Environment. *Eur. J. Inorg. Chem* **2024**, *27* (11), e202300731. DOI: 10.1002/ejic.202300731.

(3) (a) Ruff, J. K.; Hawthorne, M. F. The Amine Complexes of Aluminum Hydride. I. *J. Am. Chem. Soc.* **1960**, *82* (9), 2141-2144. DOI: 10.1021/ja01494a013;

(b) Murphy, F.; Kennedy, A. R.; Weetman, C. E. Synthesis and Structural Comparisons of NHC-Alanes. *Inorganics* **2022**, *11* (1), 13. DOI: 10.3390/inorganics11010013.

(4) (a) Jafarpour, L.; Stevens, E. D.; Nolan, S. P. A sterically demanding nucleophilic carbene: 1,3-*bis*(2,6-diisopropylphenyl)imidazol-2-ylidene). Thermochemistry and catalytic application in olefin metathesis. *J. Organomet. Chem.* **2000**, *606* (1), 49-54. DOI: 10.1016/S0022-328X(00)00260-6;

(b) Baker, R. J.; Davies, A. J.; Jones, C.; Kloth, M. Structural and spectroscopic studies of carbene and *N*-donor ligand complexes of Group 13 hydrides and halides. *J. Organomet. Chem.* **2002**, *656* (1), 203-210. DOI: 10.1016/S0022-328X(02)01592-9.

(5) Chowdhury, T.; Evans, M. J.; Coles, M. P.; Bailey, A. G.; Peveler, W. J.; Wilson, C.; Farnaby, J. H. Reduction chemistry yields stable and soluble divalent lanthanide tris(pyrazolyl)borate complexes. *Chem. Commun.* **2023**, *59* (15), 2134-2137, 10.1039/D2CC03189B. DOI: 10.1039/D2CC03189B.

(6) Stainer, M. V. R.; Takats, J. Solution structure of [Yb(Tp)$_3$]; rare example of solution rigid lanthanide complex. *J. Am. Chem. Soc.* **1983**, *105* (3), 410-415. DOI: 10.1021/ja00341a020.

(7) Apostolidis, C.; Rebizant, J.; Kanellakopulos, B.; Von Ammon, R.; Dornberger, E.; Müller, J.; Powietzka, B.; Nuber, B. Homoscorpionates (Tp complexes) of the trivalent 4f ions. The crystal and molecular structure of [Ln(Tp)$_3$] (Ln = Pr, Nd). *Polyhedron* **1997**, *16* (7), 1057-1068. DOI: 10.1016/s0277-5387(96)00391-9.
